# Mechanochemical synthesis of magnesium-based carbon nucleophiles in air and their use in organic synthesis

**DOI:** 10.1038/s41467-021-26962-w

**Published:** 2021-11-18

**Authors:** Rina Takahashi, Anqi Hu, Pan Gao, Yunpeng Gao, Yadong Pang, Tamae Seo, Julong Jiang, Satoshi Maeda, Hikaru Takaya, Koji Kubota, Hajime Ito

**Affiliations:** 1 Division of Applied Chemistry, Graduate School of Engineering, Hokkaido University, Sapporo, Hokkaido 060-8628 Japan; 2 Institute for Chemical Reaction Design and Discovery (WPI-ICReDD), Hokkaido University, Sapporo, Hokkaido 060-8628 Japan; 3 Department of Chemistry, Faculty of Science, Hokkaido University, Sapporo, Hokkaido 060-8628 Japan; 4 Institute for Chemical Research, Kyoto University, Gokasho, Uji, Kyoto, 611-0011 Japan; 5 Division of Photo-Molecular Science III/Advanced Molecular Science, Institute for Molecular Science, Myodaiji, Okazaki, Aichi 444-8585 Japan

**Keywords:** Synthetic chemistry methodology, Sustainability

## Abstract

Since the discovery of Grignard reagents in 1900, the nucleophilic addition of magnesium-based carbon nucleophiles to various electrophiles has become one of the most powerful, versatile, and well-established methods for the formation of carbon−carbon bonds in organic synthesis. Grignard reagents are typically prepared via reactions between organic halides and magnesium metal in a solvent. However, this method usually requires the use of dry organic solvents, long reaction times, strict control of the reaction temperature, and inert-gas-line techniques. Despite the utility of Grignard reagents, these requirements still represent major drawbacks from both an environmental and an economic perspective, and often cause reproducibility problems. Here, we report the general mechanochemical synthesis of magnesium-based carbon nucleophiles (Grignard reagents in paste form) in air using a ball milling technique. These nucleophiles can be used directly for one-pot nucleophilic addition reactions with various electrophiles and nickel-catalyzed cross-coupling reactions under solvent-free conditions.

## Abstract (web summary)

Grignard reagents have widespread utility in organic chemistry, but their preparation is limited by several drawbacks, such as the use of dry organic solvents and long reaction times. Here, the authors report a general mechanochemical synthesis of Grignard reagents in paste form in air, using a ball milling technique.

## Introduction

**Fig. 1:**
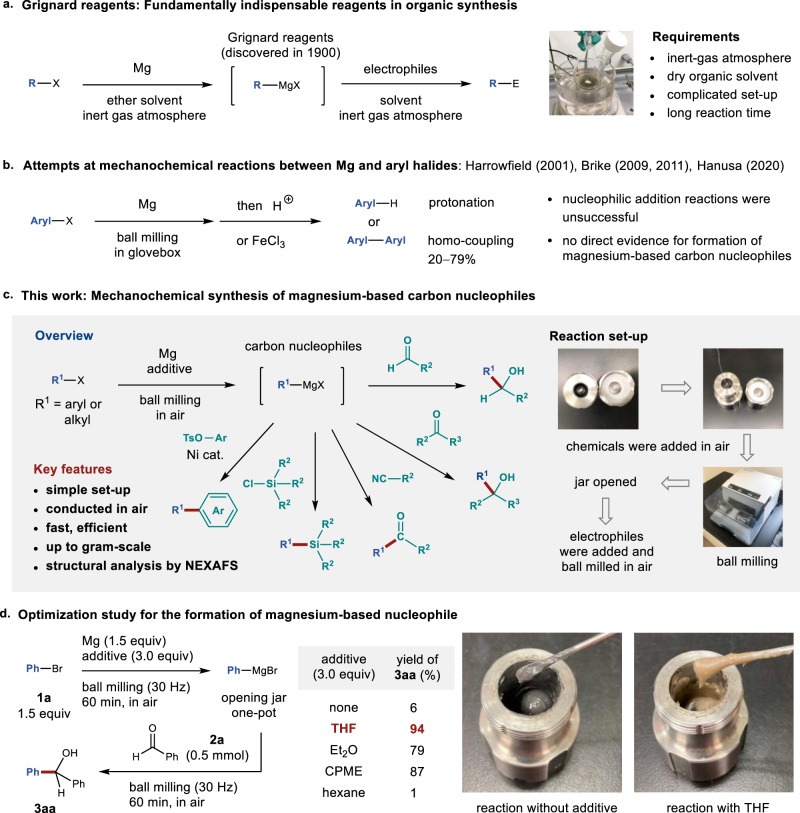
Synthesis of organomagnesium nucleophiles using mechanochemistry and their application to organic synthesis. **a** Conventional solution-based method for the preparation of Grignard reagents. **b** Previous attempts to mechanochemically synthesize organomagnesium nucleophiles. **c** Mechanochemical synthesis of organomagnesium nucleophiles and their application to organic synthesis. **d** Optimization study for the formation of magnesium-based nucleophiles using ball milling.

The discovery of what later became commonly known as the ‘Grignard reagents’ and their use as carbon nucleophiles were first reported in 1900 by Victor Grignard^1^. Since then, Grignard reagents have occupied an important place in organic chemistry, as they have been used to produce numerous synthetic intermediates and valuable functional molecules in the materials, pharmaceutical, food, polymer, and related chemical industries^2–5^. Thus, the development of efficient methods for their preparation has attracted considerable interest^6–8^. The direct insertion of magnesium metal into organic halides is one of the most established routes, as it is an atom-economical process with low toxicity (Fig. 1a)^9^. However, this method usually requires the use of dry organic solvents, long reaction times, strict control of the reaction temperature, and inert-gas-line techniques. Moreover, the surface of the magnesium metal may be covered with an unreactive oxide layer^10^, which requires a pre-activation process involving heating, ultrasound^11^, or microwave^12^ treatment, and/or the addition of activating reagents^13–15^. Unfortunately, these requirements still represent major drawbacks from both an environmental and an economic perspective, and often cause reproducibility problems for unreactive organic halides.

In this context, the use of ball milling techniques^16–24^ for the solvent-free preparation of Grignard reagents via reactions between aryl halides and magnesium metal has been studied by several research groups (Fig. 1b). In 2001, Harrowfield and co-workers first attempted mechanochemical reactions of 1-chloro- or 1-bromo-naphthalenes with magnesium metal in a glovebox^25^. Unfortunately, their subsequent one-pot nucleophilic addition to ketones resulted in complex product mixtures. As part of a search for dehalogenation reactions of harmful organic compounds, Birke and co-workers reported that the complete protonation of aryl chlorides could be achieved by milling them with magnesium and *n*-butyl amine in a glovebox^26^. More recently, Hanusa and co-workers reported the mechanochemical reactions of bromo- or fluoroarenes with magnesium metal in a glovebox^27^. Subsequent addition of FeCl_3_ to the reaction mixture gave the corresponding homo-coupling products in moderate to low yield. However, one-pot mechanochemical reactions with carbonyl electrophiles did not provide the corresponding nucleophilic addition products. More recently, Yang, Dai, and co-workers also reported magnesium-mediated reductive radical homo-coupling reactions of polyhaloarenes^28^. Although these pioneering studies are highly remarkable, neither successful examples of the use of magnesium-based carbon nucleophiles prepared by ball milling for the formation of carbon−carbon bonds with various electrophiles^29^, nor direct spectroscopic evidence for the formation of carbon−magnesium bonds under mechanochemical conditions have been reported so far.

Herein, we report that a mechanochemical approach using ball milling allows for a highly efficient, general, and robust method for the preparation of magnesium-based carbon nucleophiles in air and their application to various organic transformations under mechanochemical conditions (Fig. 1c). The key to the success of this protocol is the addition of small amounts of tetrahydrofuran (THF) or cyclopentyl methyl ether (CPME), which facilitates the formation of organomagnesium nucleophiles and their addition to electrophiles. The carbon nucleophiles thus formed can be used for various transformations including carbonyl addition, carbon−silicon bond formation, nickel-catalyzed Kumada–Tamao–Korriu cross-coupling reactions, and metal-catalyzed selective addition reactions to conjugated enones under mechanochemical conditions. Notably, the developed protocol does not require the use of inert gas or dry organic solvents, allowing the entire procedure to be conducted in air without necessitating any special precautions or synthetic techniques. We also succeeded in the preparation of an organomagnesium reagent from a poorly soluble aryl halide, and it should be noted here that this reagent cannot be synthesized via conventional solution-based protocols. Near edge, X-ray absorption fine structure (NEXAFS) spectroscopy is used to analyze the generation of the magnesium-based carbon nucleophiles under mechanochemical conditions. The present study thus provides a new platform centered on magnesium-based carbon nucleophiles, which has the potential to update modern organic synthesis with a more cost-effective and environmental-friendly procedure for Grignard reactions.

## Results

### Mechanochemical synthesis of magnesium-based carbon nucleophiles in air

We first attempted the synthesis of organomagnesium species from bromobenzene [**1a**, 1.5 equiv for benzaldehyde (**2a**)] and magnesium turnings [1.5 equiv for benzaldehyde (**2a**)] and a subsequent nucleophilic addition to benzaldehyde (**2a**) using a Retch MM400 mixer mill (5 mL stainless-steel milling jar with a 10 mm-diameter stainless-steel ball) (Fig. 1d). After the ball milling of **1a** and magnesium turnings for 60 min, the jar was opened in air, and a gray oil was obtained (Fig. 1d, left photograph). Visible magnesium metal grains or powder were not observed in the mixture. Then, **2a** was added to the jar and ball milling was continued for 60 min. The desired nucleophilic addition product (**3aa**) was obtained, albeit in only 6% NMR yield. Next, we attempted to improve the reactivity by using liquid ethers as additives in order to facilitate the formation of the organomagnesium nucleophiles and their addition to the electrophiles. The use of 2.0 equivalents of tetrahydrofuran (THF) relative to the amount of magnesium afforded a muddy light-orange mixture (Fig. 1d, right photograph). Then, this mixture was ball-milled with **2a**, which dramatically improved the yield of **3aa** to 94%. We also tested other liquid ethers, such as diethyl ether (Et_2_O) and cyclopentyl methyl ether (CPME), but the resulting yields were lower than that obtained using THF. The use of liquid additives that do not contain an oxygen atom, such as hexane, did not improve the yield of **3aa**. The protocol using THF as an additive was applied to the synthesis of magnesium-based carbon nucleophiles from iodobenzene (**1a′**) and chlorobenzene (**1a″**), and the desired alcohol **3aa** was obtained in good yields of 74 and 84%, respectively (for details, see the Supplementary Material). Notably, the reaction of **1a** and magnesium was also carried out on a gram scale in a 10 mL stainless-steel ball-milling jar with two 15 mm-diameter stainless-steel balls to afford **3aa** in 93% yield (1.03 g; for details, see the Supplementary Material). This result underscores the high practical utility of this protocol. Even when the reaction was carried out under a nitrogen atmosphere, the product yield was not improved. This indicates that air (oxygen and CO_2_) in the reaction vessel does not significantly affect the efficiency of this protocol.

### Substrate scope of the nucleophilic addition to aldehydes and ketones

**Fig. 2:**
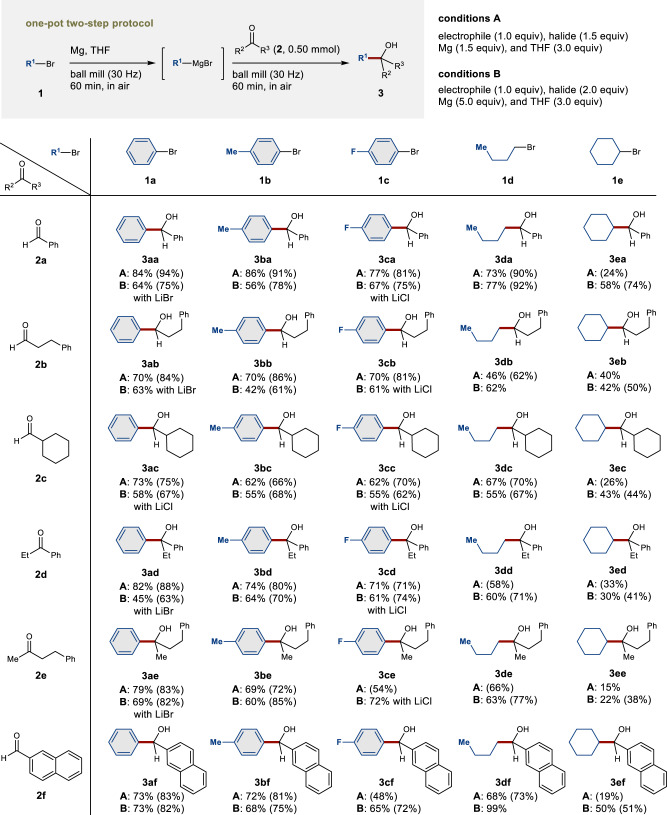
Scope of the mechanochemical synthesis of organomagnesium nucleophiles and their nucleophilic addition to aldehydes and ketones in a ball mill. A stainless-steel milling jar (5 mL) and stainless-steel ball (diameter: 10 mm) were used. Isolated yields are reported as percentages. Proton NMR integrated yields are shown in parentheses. For details, see the Supplementary Information.

With the optimized conditions (conditions A) in hand, we proceeded to investigate the substrate scope for the nucleophilic addition to aldehydes and ketones (Fig. 2). Our results show that this method is characterized by a broad substrate scope that encompasses aromatic and aliphatic bromides (**1a**−**1e**), aldehydes (**2a**−**2c** and **2f**), and ketones (**2d** and **2e**) to give the desired alcohols (**3aa**−**3ef**) in moderate to high yields. Furthermore, this method was also applicable to aryl halides that bear various substituents such as methoxy- and dimethylamino groups in *para*- and *ortho*-position (for details, see the Supplementary Material). To improve the yield of **3**, we also tested the use of an excess of magnesium (5.0 equiv) or organic halide (2.0 equiv) (conditions B). Although conditions B generally provided product yields comparable to those achieved using conditions A, conditions B improved the efficiency of the reactions using secondary alkyl bromide **1e**, affording the products **3ea**−**3ef** in moderate to good yields. The addition of lithium salts has slightly improved the yields of **3aa**−**3ae** and **3ca**−**3ce** (for details, the Supplementary Material). We also attempted the reactions using the liquid substrates in a test tube with efficient mixing by magnetic stirring under the optimized conditions, but these reactions resulted in poor or almost no product formation (for details, the Supplementary Material). These results suggest that strong mechanical agitation in the ball mill is crucial for the formation of the magnesium-based carbon nucleophiles^30^.

### Mechanochemical synthesis of magnesium-based carbon nucleophiles from solid aryl halides

**Fig. 3:**
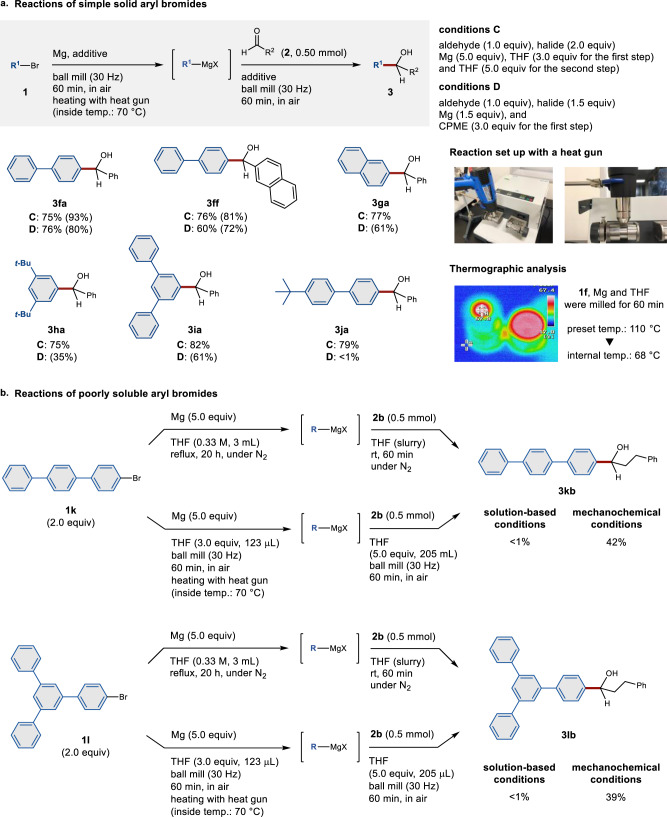
Synthesis of organomagnesium nucleophiles via a high-temperature ball-milling technique. A stainless-steel milling jar (5 mL) and stainless-steel ball (diameter: 10 mm) were used; for details, see the Supplementary Information. Isolated yields are reported as percentages. Proton NMR integrated yields are shown in parentheses. **a** Mechanochemical synthesis of magnesium-based carbon nucleophiles from solid aryl halides. **b** Mechanochemical conditions allowed the formation of magnesium-based carbon nucleophiles from poorly soluble aryl halides **1k** and **1l**, which are not readily applicable to the conventional solution-based protocol.

We found that attempts to react magnesium and solid aryl halides such as 4-bromobiphenyl (**1f**) for the subsequent nucleophilic addition to benzaldehyde (**2a**) resulted in almost no reaction under the optimized conditions (conditions A or B). Neither prolonging the reaction time nor using liquid additives improved the yield of the product (**3fa**). To promote the formation of magnesium-based nucleophiles from the solid aryl halides, we decided to carry out the reaction at higher temperature. In one of our previous studies, we revealed that external heating enables poorly soluble aryl halides to react in solid-state cross-coupling reactions^31^. In that case, external heating may help to weaken the intermolecular interactions of solid substrates, which would improve the mixing efficiency and promote chemical reactions. Specifically, we placed a commercially available, temperature-controllable heat gun directly above the ball-milling jar^31^. The mechanochemical reactions between magnesium and the solid aryl halides were conducted while applying hot air to the outside of the milling jar. We set the temperature of the heat gun to 110 °C in order to ensure an internal reaction temperature of 70 °C, which was confirmed by thermography immediately after opening the milling jar (Fig. 3a). The reaction between magnesium and **1f** in the presence of THF (3.0 equiv) was complete within 1 h and formed the desired nucleophilic product (**3fa**) in high yield (75%). In this procedure, THF (5.0 equiv) was added for the nucleophilic addition step, which improved the yield of **3fa**. These high-temperature ball-milling conditions (conditions C) were applied to various solid bromides (**1f**−**1j**) and afforded the corresponding alcohols (**3ff**−**3ja**) in high yield. The use of CPME instead of THF provided comparable yields of the addition products (conditions D).

Reactions of Mg metal with poorly soluble aryl halides under conventional solution-based conditions are often inefficient. For example, the reaction of poorly soluble 4-bromoterphenyl (**1k**) as a slurry under reflux conditions (309.2 mg of **1k** in 3 mL of THF, 0.33 M, for 20 h) did not afford any nucleophilic addition product (Fig. 3b). We also attempted using more dilute conditions (309.2 mg of **1k** in 15 mL of THF, 0.067 M, for 20 h), albeit that **3kb** was still not obtained. In contrast, the developed mechanochemical conditions provided the corresponding magnesium-based carbon nucleophile from **1k** via high-temperature ball milling within 1 h, and the desired nucleophilic addition product (**3kb**) was obtained in moderate yield (42%). Furthermore, poorly soluble **1l** was also reactive under the solid-state conditions and formed the desired addition product (**3lb**) in 39% yield, while **3lb** was not obtained under solution-based conditions. These results demonstrate the potential of this mechanochemical protocol as an operationally simple and efficient route to synthesize magnesium-based carbon nucleophiles from poorly soluble substrates that are incompatible with conventional solution-based conditions.

### Applications of mechanochemically generated organomagnesium nucleophiles to various organic transformations

**Fig. 4:**
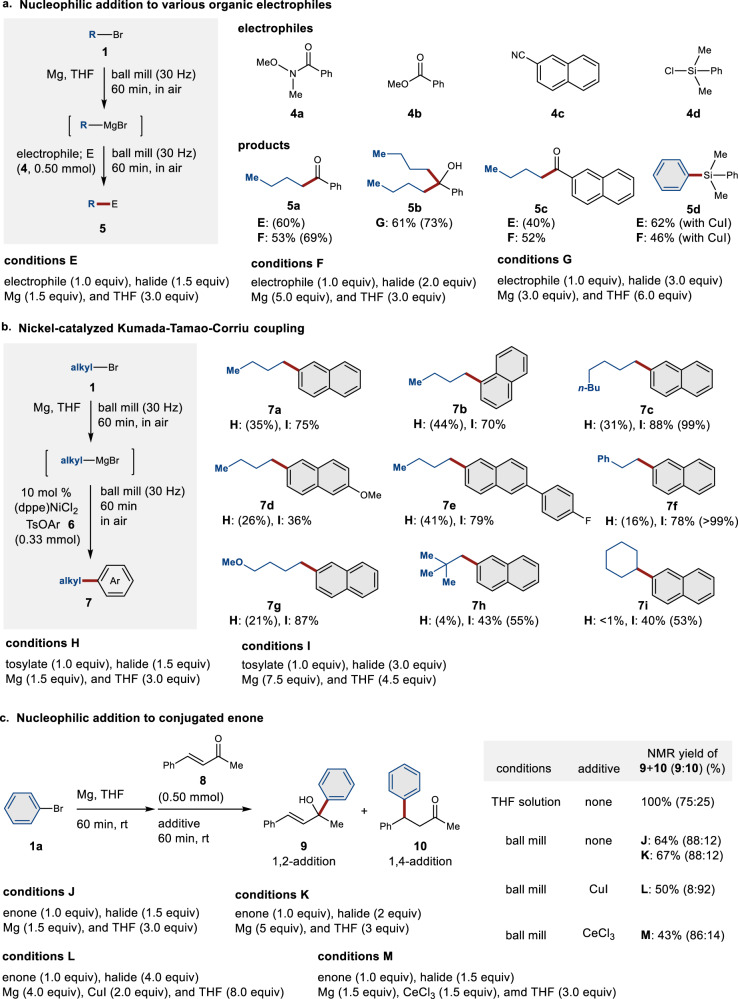
Various organic transformations using mechanochemically generated organomagnesium nucleophiles. Isolated yields are reported as percentages. Proton NMR integrated yields are shown in parentheses. **a** Nucleophilic addition to various electrophiles under mechanochemical conditions. **b** Mechanochemical nickel-catalyzed Kumada–Tamao–Corriu coupling reactions. **c** Nucleophilic addition reactions to conjugated enone **8** under mechanochemical conditions. A stainless-steel milling jar (5 mL) and a stainless-steel ball (diameter: 10 mm) were used. For details, see the Supplementary Information.

The mechanochemically synthesized organomagnesium nucleophiles were applicable to reactions with various organic electrophiles (Fig. 4a). A Weinreb amide (**4a**) and ester (**4b**) were converted to the corresponding ketone (**5a**) and tertiary alcohol (**5b**) in good yield. Nitrile (**4c**) reacted with the organomagnesium nucleophile derived from **1d** even at room temperature to provide the desired ketone (**5c**) in moderate yield. Other C−C-bond-forming reactions with carbon dioxide, epoxides, and amides afforded the corresponding products in low to good yield (for details, see the Supplementary Material). In addition, the organomagnesium reagents were applicable to not only C−C-bond-formation reactions but also to Si−C-bond-formation reactions with chlorosilane (**4d**) in the presence of a catalytic amount of copper iodide^32^.

Next, we investigated nickel-catalyzed Kumada–Tamao–Corriu coupling reactions between the mechanochemically synthesized organomagnesium nucleophiles and aryl tosylates under ball-milling conditions (Fig. 4b)^33^. After the formation of the organomagnesium reagent generated from **1d**, the jar was opened in air and 2-naphthyl tosylate (**6a**) and 10 mol% of (dppe)NiCl_2_ were added quickly. The subsequent ball-milling reaction afforded cross-coupling product **7a** in 35% NMR yield (conditions H). We found that increasing the amounts of **1d**, magnesium, and THF improved the yield of **7a** to 75% (conditions I). Under conditions I, reactions with simple naphthyl tosylates gave the desired products (**7b** and **7c**) in high yield (70% and 88%, respectively). However, **7d**, i.e., the product derived from naphthyl tosylate bearing a methoxy group (**6c**), was obtained in relatively low yield (36%), while the product bearing a fluoride atom (**7e**) was obtained in good yield (79%). A variety of primary and secondary alkyl bromides were applicable to these mechanochemical cross-coupling reactions (**7f**−**7i**). To the best of our knowledge, this is the first example of Kumada–Tamao–Corriu cross-coupling reactions under mechanochemical conditions.

Nucleophilic addition reactions of the mechanochemically synthesized organomagnesium nucleophiles to conjugated enone **8** were also examined (Fig. 4c). The reaction of the phenyl magnesium reagent with benzylidene acetone (**8**) in THF solution at room temperature afforded a mixture of the 1,2-addition product (**9**) and the 1,4-addition product (**9**/**10** = 75:25). Interestingly, the selectivity toward 1,2-addition increased under mechanochemical conditions (**9**/**10** = 88:12). Furthermore, the mechanochemical nucleophilic addition in the presence of CuI afforded the 1,4-addition product (**10**) with high selectivity (50%; **9**/**10** = 8:92), suggesting that the corresponding cuprate might be formed even under mechanochemical conditions. The addition of CeCl_3_ did not influence the selectivity under the mechanochemical conditions (43%; **9**/**10** = 86:14), while under the corresponding solution-based conditions, CeCl_3_ improved the selectivity toward the 1,2-addition product ^34, 35^.

### Confirmation of the formation of magnesium-based carbon nucleophiles under ball-milling conditions

In order to confirm the generation of magnesium-based carbon nucleophiles under ball-milling conditions, a deuterium-labeling experiment was conducted (for details, see the Supplementary Material). 3,5-Di-*tert*-butylphenyl bromide (**1h**) was treated with magnesium under the optimized conditions, the jar was opened, and CD_3_CO_2_D was added quickly. The subsequent ball-milling reaction furnished deuterium-labeled 1,3-di-*tert*-butylbenzene **11** (>99% D), which suggests that the corresponding magnesium-based carbon nucleophile is formed.

**Fig. 5:**
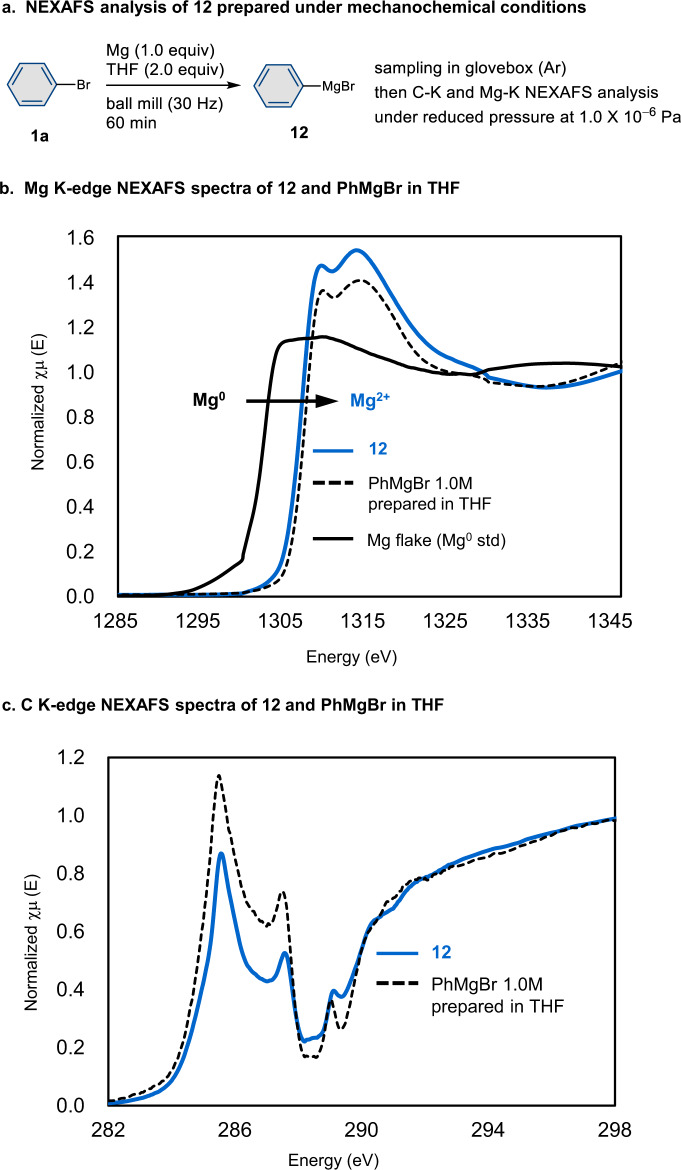
NEXAFS analysis of a magnesium-based carbon nucleophile under mechanochemical conditions. **a** Preparation of a sample of magnesium-based carbon nucleophile **12** under mechanochemical conditions. **b** Mg K-edge NEXAFS spectra of mechanochemically prepared **12**, PhMgBr prepared in solution, and a magnesium flake of a Mg^0^ standard. **c** C K-edge NEXAFS spectra of mechanochemically prepared **12** and PhMgBr prepared in solution.

Furthermore, near-edge X-ray absorption fine structure (NEXAFS) spectroscopy was used to analyze the generation of magnesium-based carbon nucleophiles under mechanochemical conditions (Fig. 5). The NEXAFS measurements were carried out at UVSOR synchrotron facility (Japan) using the magnesium-based carbon nucleophile **12**, which was prepared by ball milling and transferred into the soft X-ray optics under an argon atmosphere (Fig. 5a). The formation of the divalent cationic Mg^2+^ species was unequivocally confirmed by the high-energy shift of the Mg K-absorption edge (1307.4 eV) relative to the Mg^0^ edge (1302.6 eV) of a standard magnesium flake (Fig. 5b)^36^. The high similarity of Mg-K and C-K NEXAFS spectra of mechanochemically-prepared **12** and PhMgBr prepared in solution (Figs. 5b, c) supports the formation of similar organomagnesium species that possess a carbon–magnesium bond under both ball-milling and solution conditions in THF. The intense 1s–π* transition peaks^37^ at 285.7 and 287.7 eV in the C-K NEXAFS spectra also support the formation of a carbon–magnesium bond resulting from the transformation of the C–Br bond of the starting bromobenzene (for details, see the Supplementary Material). A preliminary theoretical study was also carried out to predict the structure of the magnesium-based carbon nucleophiles prepared by ball-milling (for details, see Supplementary Fig. 13).

## Discussion

The present mechanochemical synthesis of magnesium-based carbon nucleophiles and their reactions with various electrophiles can be carried out in air without the need for large amounts of dry and degassed solvent(s), special precautions, or synthetic techniques. In addition to these advantages, solid-state ball-milling conditions enabled the synthesis of magnesium-based carbon nucleophiles from poorly soluble aryl halides that are incompatible with conventional solution-based conditions. This can expand the utility of Grignard reagents. Direct spectroscopic evidence for the formation of magnesium-based carbon nucleophiles under mechanochemical conditions was obtained using NEXAFS spectroscopy. Given the widespread use of Grignard reagents in modern organic chemistry, we anticipate that this approach will inspire the development of attractive synthetic applications to complement existing solution-based technologies. Shortly after our submission, the Bolm group reported mechanochemical Grignard reactions with gaseous CO_2_, which afford the corresponding carboxylic acids in moderate to good yield^38^. The development of Minischi-type reactions using magnesium under ball-milling conditions, which is related to our present study, has also been reported^39^

## Methods

### Representative procedure for the solvent-less synthesis of magnesium-based carbon nucleophiles and their reactions with electrophiles

Mg turnings (0.75 mmol, 1.5 equiv) were placed in a jar (stainless steel; 5 mL) with a ball (stainless steel; 10 mm, diameter) in air. An organic bromide **1** (0.75 mmol, 1.5 equiv) and THF (123 μL, 1.5 mmol, 3.0 equiv) were added to the jar using a syringe. After the jar was closed without purging with inert gas, the jar was placed in a ball mill (Retsch MM 400, 60 min, 30 Hz). After grinding for 60 min, the jar was opened in air and charged with an electrophile **2** (0.50 mmol). The jar was then closed without purging with inert gas, and was placed in the ball mill (Retsch MM 400, 60 min, 30 Hz). After grinding for 60 min, the reaction mixture was quenched with a saturated aqueous solution of NH_4_Cl and extracted with CH_2_Cl_2_ (30 mL×3). The solution was washed with brine and dried over Na_2_SO_4_. After the removal of the solvents under reduced pressure, the crude material was purified by flash column chromatography (SiO_2_, hexane/ethyl acetate, 100:0 to 80:20) to give the corresponding product **3**.

## Supplementary information

Supporting Information

## Data Availability

For full characterization data including NMR spectra of the new compounds and experimental details, see the Supplementary Material. All relevant data underlying the results of this study are available from the corresponding authors upon request.
