# Supplementary material for: Mechanochemical synthesis of magnesium-based carbon nucleophiles in air and their use in organic synthesis
Source: Nat Commun. 2021 Nov 18;12:6691. doi: 10.1038/s41467-021-26962-w (PMC8602241; doi:10.1038/s41467-021-26962-w)
Supplement: Supplementary file 1 — Supporting Information [file 41467_2021_26962_MOESM1_ESM.pdf]

Supplementary Information for

Mechanochemical synthesis of magnesium-based carbon nucleophiles in air and their use in organic synthesis

Rina Takahashi, Anqi Hu, Yunpeng Gao, Pan Gao, Yadong Pang, Tamae Seo, Julong Jiang, Satoshi Maeda, Hikaru Takaya, Koji Kubota*, Hajime Ito*

Correspondence to: kbt@eng.hokudai.ac.jp (K.K.); hajito@eng.hokudai.ac.jp (H.I.)

Supplementary Methods

Instrumentation and Chemicals

Materials were obtained from commercial suppliers and purified using standard procedures, unless otherwise noted. Solvents were purchased from commercial suppliers and further dried over molecular sieve (MS 4Å). Magnesium turnings (≥ 99.5%, product no. 137-06041) and Mg powder (99%, product no. 135-00062) were purchased from Wako Pure Chemical Industries, Co., Ltd. All reactions were performed using grinding vessels in the Retsch MM 400 (Figure S1). Both jars and balls were made of stainless steel (Figure S2). The jars were heated using a heat gun with a temperature control function (EARTH MAN HG-1450B, TAKAGI Co., Ltd.). NMR spectra were recorded on JEOL JNM-ECX400P and JNM-ECS400 spectrometers (^{1}H: 392, 396, 400, or 401 MHz, ^{13}C: 99, 100, or 101 MHz). Tetramethylsilane (^{1}H) and $CDCl_3$ (^{13}C) were employed as external standards, respectively. Multiplicity was recorded as follows: s = singlet, brs = broad singlet, d = doublet, t = triplet, q = quartet, quint = quintet, sex = sextet, sept = septet, and m = multiplet. Dibromomethane was used as an internal standard to determine the NMR yields. Thermographic images were obtained using the InfRec Thermo GEAR (NEC Avio Infrared Technologies Co., Ltd.). Recycling preparative gel permeation chromatography (GPC) was conducted with the JAI LC-9101 using $CHCl_3$ as the eluent with the JAIGEL-1H. High-resolution mass spectra were recorded at the Global Facility Center, Hokkaido University.

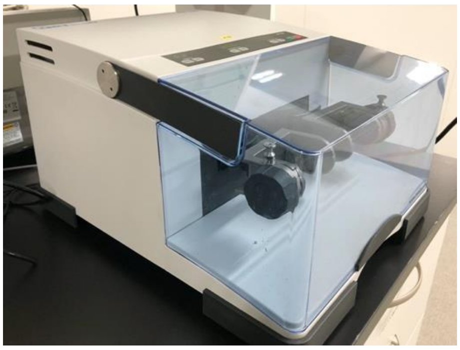

Supplementary Figure 1. Retsch MM400 used in this study.

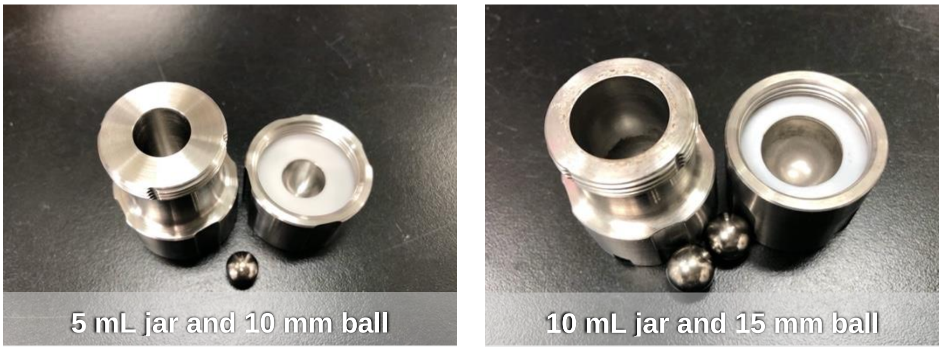

Supplementary Figure 2. Stainless jars and balls used in this study.

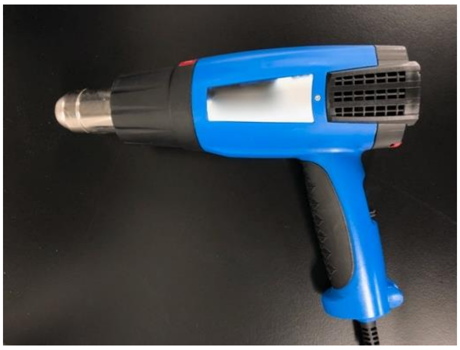

Supplementary Figure 3. Temperature-controllable heat gun used in this study.

General Procedures

Conditions A: Reactions using liquid bromides

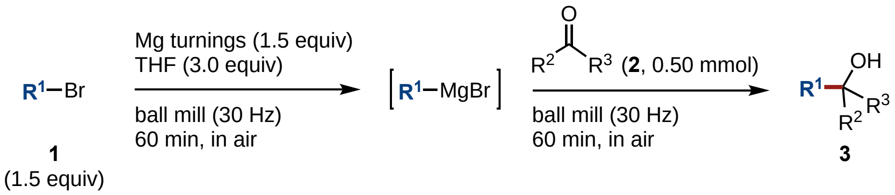

Mg turnings (0.75 mmol, 1.5 equiv) were placed in a milling jar (5 mL) with a ball (10 mm, diameter) in air. An organic bromide (**1**, 0.75 mmol, 1.5 equiv) and THF (123 µL, 1.5 mmol, 3.0 equiv) were added to the jar using a syringe. After the jar was closed without purging with inert gas, the jar was placed in the ball mill (Retsch MM 400, 1 h, 30 Hz). After grinding for 1 h, the jar was opened in air and charged with a distilled aldehyde (**2a**–**2c** and **2f**, 0.50 mmol, distilled before use) or a ketone (**2d** and **2e**, 0.50 mmol). The jar was then closed without purging with inert gas, and was placed in the ball mill (Retsch MM 400, 1 h, 30 Hz). After grinding for 1 h, the reaction mixture was quenched with a saturated aqueous solution of NH_4Cl and extracted with CH_2Cl_2 (30 mL×3). The solution was washed with brine and dried over Na_2SO_4. After the removal of the solvents under reduced pressure, the crude material was purified by flash column chromatography (SiO_2, hexane/ethyl acetate, 100:0 to 80:20) to give the corresponding product **3**.

Conditions B: Reactions using liquid bromides

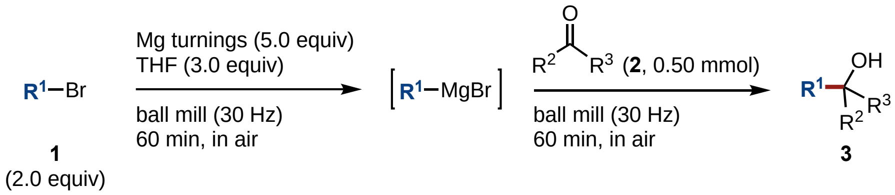

Mg turnings (2.5 mmol, 5.0 equiv) were placed in a milling jar (5 mL) with a ball (10 mm, diameter) in air. An organic bromide (**1**, 1.0 mmol, 2.0 equiv) and THF (123 µL, 1.5 mmol, 3.0 equiv) were added to the jar using a syringe. After the jar was closed without purging with inert gas, the jar was placed in a ball mill (Retsch MM 400, 1 h, 30 Hz). After grinding for 1 h, the jar was opened in air and charged with a distilled aldehyde (**2a**–**2c** and **2f**, 0.50 mmol, distilled before use) or a ketone (**2d** and **2e**, 0.50 mmol). The jar was then closed without purging with inert gas, and was placed in the ball mill (Retsch MM 400, 1 h, 30 Hz). After grinding for 1 h, the reaction mixture was quenched with a saturated aqueous solution of NH_4Cl and extracted with CH_2Cl_2 (30 mL×3). The solution was washed with brine and dried over Na_2SO_4. After the removal of the solvents under reduced pressure, the crude material was purified by flash column chromatography (SiO_2, hexane/ethyl acetate, 100:0 to 80:20) to give the corresponding product **3**. In some cases, the product **3** was further purified by recycling GPC.

Conditions C: Reactions using solid aryl bromides

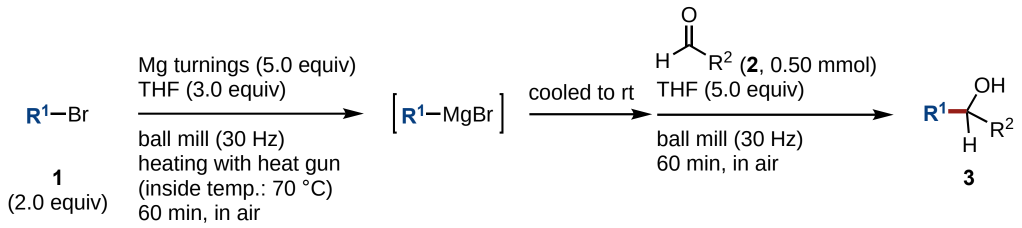

Mg turnings (2.5 mmol, 5.0 equiv) and an aryl bromide (**1**, 1.0 mmol, 2.0 equiv) were placed in a milling jar (5 mL) with a ball (10 mm, diameter) in air. THF (123 μL, 1.5 mmol, 3.0 equiv) was added to the jar using a syringe. After the jar was closed without purging with inert gas, the jar was placed in the ball mill (Retsch MM 400, 1 h, 30 Hz). A heat gun was set approximately 1 cm above the jar and was turned on (preset temperature: 110 °C, internal temperature: *ca*. 70 °C). After grinding for 1 h, the jar was cooled to room temperature, opened in air, and charged with a distilled aldehyde (**2**, 0.50 mmol) and THF (204 μL, 2.5 mmol, 5.0 equiv). The jar was then closed without purging with inert gas, and was placed in a ball mill (Retsch MM 400, 1 h, 30 Hz). After grinding for 1 h, the reaction mixture was quenched with a saturated aqueous solution of NH_4Cl and extracted with CH_2Cl_2 (30 mL×3). The solution was washed with brine and dried over Na_2SO_4. After the removal of the solvents under reduced pressure, the crude material was purified by flash column chromatography (SiO_2, hexane/ethyl acetate, 100:0 to 80:20) to give the corresponding product **3**. In some cases, the product **3** was further purified by recycling GPC.

Conditions D: Reactions using solid aryl bromides

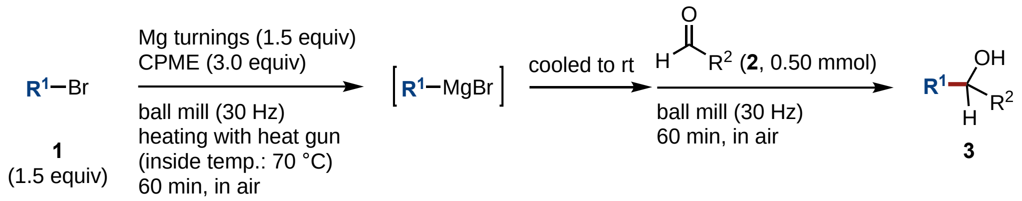

Mg turnings (0.75 mmol, 1.5 equiv) and an aryl bromide (**1**, 0.75 mmol, 1.5 equiv) were placed in a milling jar (5 mL) with a ball (10 mm, diameter) in air. CPME (175 μL, 1.5 mmol, 3.0 equiv) was added to the jar using a syringe. After the jar was closed without purging with inert gas, the jar was placed in the ball mill (Retsch MM 400, 1 h, 30 Hz). A heat gun was set in a downward direction approximately 1 cm above the jar and was turned on (preset temperature: 110 °C, internal temperature: *ca*. 70 °C). After grinding for 1 h, the jar was cooled to room temperature, opened in air, and charged with a distilled aldehyde (**2**, 0.50 mmol). The jar was then closed without purging with inert gas, and was placed in the ball mill (Retsch MM 400, 1 h, 30 Hz). After grinding for 1 h, the reaction mixture was quenched with a saturated aqueous solution of NH_4Cl and extracted with CH_2Cl_2 (30 mL×3). The solution was washed with brine and dried over Na_2SO_4. After the removal of the solvents under reduced pressure, the crude material was purified by flash column chromatography (SiO_2, hexane/ethyl acetate, 100:0 to 80:20) to give the corresponding product **3**.

Conditions E: Nucleophilic addition to various electrophiles

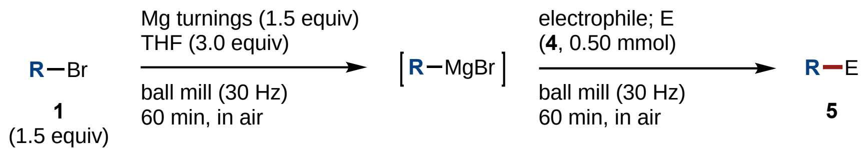

Mg turnings (0.75 mmol, 1.5 equiv) were placed in a milling jar (5 mL) with a ball (10 mm, diameter) in air. An organic bromide (**1**, 0.75 mmol, 1.5 equiv) and THF (123 μL, 1.5 mmol, 3.0 equiv) were added to the jar using a syringe. After the jar was closed without purging with inert gas, the jar was placed in the ball mill (Retsch MM 400, 1 h, 30 Hz). After grinding for 1 h, the jar was opened in air, and charged with an electrophile (**4**, 0.50 mmol). The jar was then closed without purging with inert gas, and was placed in the ball mill (Retsch MM 400, 1 h, 30 Hz). After grinding for 1 h, the reaction mixture was quenched with a saturated aqueous solution of NH_4Cl and extracted with CH_2Cl_2 (30 mL×3). The solution was washed with brine and dried over Na_2SO_4. After the removal of the solvents under reduced pressure, the crude material was purified by flash column chromatography (SiO_2, hexane/ethyl acetate, 100:0 to 80:20) to give the corresponding product **5**. In some cases, the product **5** was further purified by recycling GPC.

Conditions F: Nucleophilic addition to various electrophiles

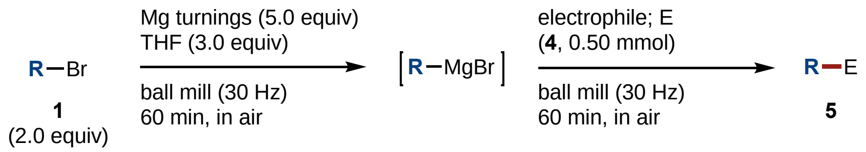

Mg turnings (2.5 mmol, 5.0 equiv) were placed in a milling jar (5 mL) with a ball (10 mm, diameter) in air. An organic bromide (**1**, 1.0 mmol, 2.0 equiv) and THF (123 μL, 1.5 mmol, 3.0 equiv) were added to the jar using a syringe. After the jar was closed without purging with inert gas, the jar was placed in the ball mill (Retsch MM 400, 1 h, 30 Hz). After grinding for 1 h, the jar was opened in air and charged with an electrophile (**4**, 0.50 mmol). The jar was then closed without purging with inert gas and was placed in the ball mill (Retsch MM 400, 1 h, 30 Hz). After grinding for 1 h, the reaction mixture was quenched with a saturated aqueous solution of NH_4Cl and extracted with CH_2Cl_2 (30 mL×3). The solution was washed with brine and dried over Na_2SO_4. After the removal of the solvents under reduced pressure, the crude material was purified by flash column chromatography (SiO_2, hexane/ethyl acetate, 100:0 to 80:20) to give the corresponding product **5**. In some cases, the product **5** was further purified by recycling GPC.

Conditions G: Nucleophilic addition to ester

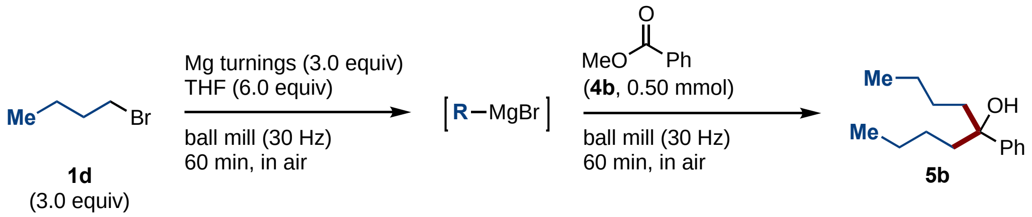

Mg turnings (1.0 mmol, 3.0 equiv) were placed in a milling jar (5 mL) with a ball (10 mm, diameter) in air. Then 1-bromobutane (**1d**, 1.0 mmol, 3.0 equiv) and THF (164 µL, 2.0 mmol, 6.0 equiv) were added to the jar using a syringe. After the jar was closed without purging with inert gas, the jar was placed in the ball mill (Retsch MM 400, 1 h, 30 Hz). After grinding for 1 h, the jar was opened in air and charged with methyl benzoate (**4b**, 0.33 mmol). The jar was then closed without purging with inert gas and was placed in the ball mill (Retsch MM 400, 1 h, 30 Hz). After grinding for 1 h, the reaction mixture was quenched with a saturated aqueous solution of NH_4Cl and extracted with CH_2Cl_2 (30 mL×3). The solution was washed with brine and dried over Na_2SO_4. After the removal of the solvents under reduced pressure, the crude material was purified by flash column chromatography (SiO_2, hexane/ethyl acetate, 100:0 to 80:20) to give the corresponding product **5b**.

Conditions H: Kumada-Tamao-Corriu coupling reactions

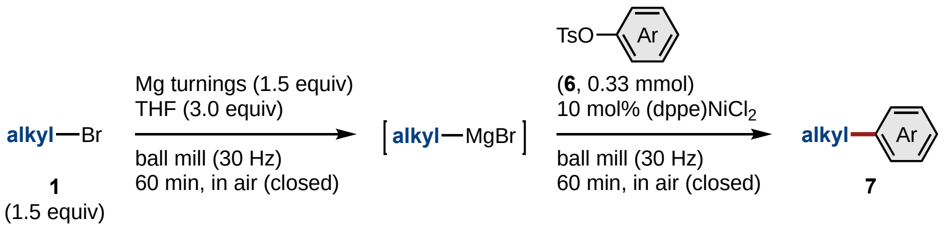

Mg turnings (0.50 mmol, 1.5 equiv) were placed in a milling jar (5 mL) with a ball (10 mm, diameter) in air. An alkyl bromide (**1**, 0.50 mmol, 1.5 equiv) and THF (82 µL, 1.0 mmol, 3.0 equiv) were added to the jar using a syringe. After the jar was closed without purging with inert gas, the jar was placed in the ball mill (Retsch MM 400, 1 h, 30 Hz). After grinding for 1 h, the jar was opened in air and was charged with (dppe)$NiCl_2$ (0.033 mmol, 0.10 equiv) and an aryl tosylate (**6**, 0.33 mmol). The jar was then closed without purging with inert gas and was placed in the ball mill (Retsch MM 400, 1 h, 30 Hz). After grinding for 1 h, the reaction mixture was quenched with 1.0 M HCl and extracted with CH_2Cl_2 (30 mL×3). The solution was dried over Na_2SO_4 and the solvents were removed under reduced pressure. The crude material was purified by flash column chromatography (SiO_2, hexane/CH_2Cl_2, 97:3) to give the corresponding product **7**. In some cases, the product **7** was further purified by recycling GPC.

Conditions I: Kumada-Tamao-Corriu coupling reactions

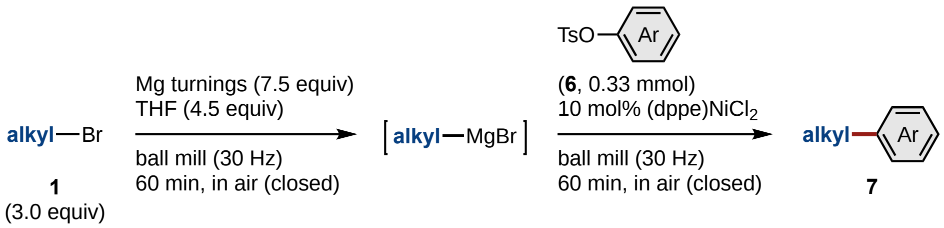

Mg turnings (2.5 mmol, 7.5 equiv) were placed in a milling jar (5 mL) with a ball (10 mm, diameter) in air. An alkyl bromide (**1**, 1.0 mmol, 3.0 equiv) and THF (123 µL, 1.5 mmol, 4.5 equiv) were added to the jar using a syringe. After the jar was closed without purging with inert gas, the jar was placed in the ball mill (Retsch MM 400, 1 h, 30 Hz). After grinding for 1 h, the jar was opened in air and was charged with (dppe)$NiCl_2$ (0.033 mmol, 0.10 equiv) and an aryl tosylate (**6**, 0.33 mmol). The jar was then closed without purging with inert gas and was placed in the ball mill (Retsch MM 400, 1 h, 30 Hz). After grinding for 1 h, the reaction mixture was quenched with 1.0 M HCl and extracted with CH_2Cl_2 (30 mL×3). The solution was dried over Na_2SO_4 and the solvents were removed under reduced pressure. The crude material was purified by flash column chromatography (SiO_2, hexane/CH_2Cl_2, 97:3) to give the corresponding product **7**. In some cases, the product **7** was further purified by recycling GPC.

Conditions J: Nucleophilic addition to enone

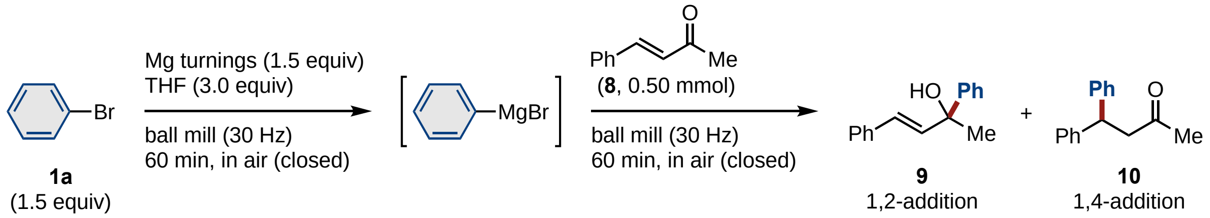

Mg turnings (0.75 mmol, 1.5 equiv) were placed in a milling jar (5 mL) with a ball (10 mm, diameter) in air. Bromobenzene (**1a**, 0.75 mmol, 1.5 equiv) and THF (123 µL, 1.5 mmol, 3.0 equiv) were added to the jar using a syringe. After the jar was closed without purging with inert gas, the jar was placed in the ball mill (Retsch MM 400, 1 h, 30 Hz). After grinding for 1 h, the jar was opened in air and was charged with (*E*)-4-phenylbut-3-en-2-one (**8**, 0.50 mmol). The jar was then closed without purging with inert gas and was placed in the ball mill (Retsch MM 400, 1 h, 30 Hz). After grinding for 1 h, the reaction mixture was quenched with a saturated aqueous solution of NH_4Cl and extracted with CH_2Cl_2 (30 mL×3). The solution was washed with brine and dried over Na_2SO_4. After the removal of the solvents under reduced pressure, ^{1}H NMR analysis of the resulting crude mixture was conducted to determine the yields of the 1,2-addition product **9** and the 1,4-addition product **10**.

Conditions K: Nucleophilic addition to enone

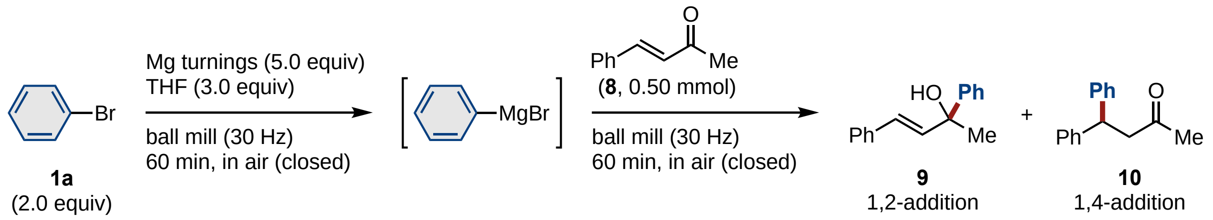

Mg turnings (2.5 mmol, 5.0 equiv) were placed in a milling jar (5 mL) with a ball (10 mm, diameter) in air. Bromobenzene (**1a**, 1.0 mmol, 2.0 equiv) and THF (123 µL, 1.5 mmol, 3.0 equiv) were added to the jar using a syringe. After the jar was closed without purging with inert gas, the jar was placed in the ball mill (Retsch MM 400, 1 h, 30 Hz). After grinding for 1 h, the jar was opened in air and was charged with (*E*)-4-phenylbut-3-en-2-one (**8**, 0.50 mmol). The jar was then closed without purging with inert gas and was placed in the ball mill (Retsch MM 400, 1 h, 30 Hz). After grinding for 1 h, the reaction mixture was quenched with a saturated aqueous solution of NH_4Cl and extracted with CH_2Cl_2 (30 mL×3). The solution was washed with brine and dried over Na_2SO_4. After the removal of the solvents under reduced pressure, ^{1}H NMR analysis of the resulting crude mixture was conducted to determine the yields of the 1,2-addition product **9** and the 1,4-addition product **10**.

Conditions L: Nucleophilic addition to enone in the presence of copper iodide

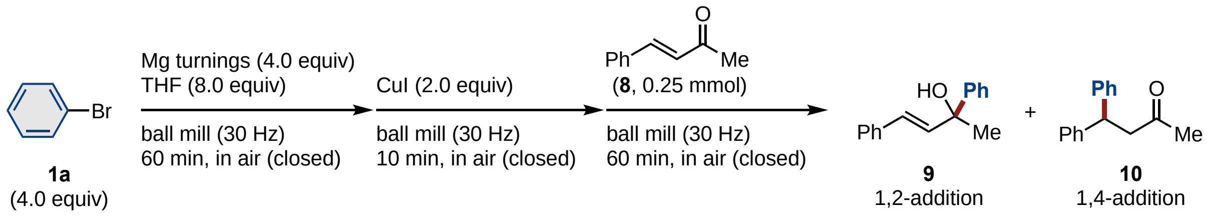

Mg turnings (1.0 mmol, 4.0 equiv) were placed in a milling jar (5 mL) with a ball (10 mm, diameter) in air. Bromobenzene (**1a**, 1.0 mmol, 4.0 equiv) and THF (164 µL, 2.0 mmol, 8.0 equiv) were added to the jar using a syringe. After the jar was closed without purging with inert gas, the jar was placed in the ball mill (Retsch MM 400, 1 h, 30 Hz). After grinding for 1 h, the jar was opened in air and was charged with copper iodide (0.50 mmol, 2.0 equiv). The jar was then closed without purging with inert gas and was placed in the ball mill (Retsch MM 400, 10 min, 30 Hz). After grinding for 10 min, the jar was opened in air and was charged with (*E*)-4-phenylbut-3-en-2-one (**8**, 0.25 mmol). The jar was then closed without purging with inert gas and was placed in the ball mill (Retsch MM 400, 1 h, 30 Hz). After grinding for 1 h, the reaction mixture was quenched with a saturated aqueous solution of NH_4Cl and extracted with CH_2Cl_2 (30 mL×3). The solution was washed with brine and dried over Na_2SO_4. After the removal of the solvents under reduced pressure, ^{1}H NMR analysis of the resulting crude mixture was conducted to determine the yields of the 1,2-addition product **9** and the 1,4-addition product **10**.

Conditions M: Nucleophilic addition to enone in the presence of cerium chloride

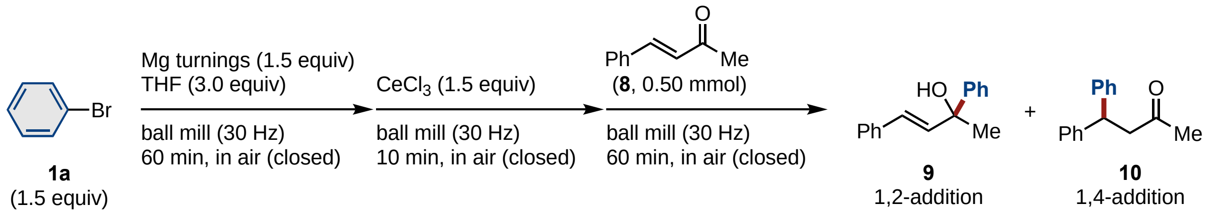

Mg turnings (0.75 mmol, 1.5 equiv) were placed in a milling jar (5 mL) with a ball (10 mm, diameter) in air. Bromobenzene (**1a**, 0.75 mmol, 1.5 equiv) and THF (123 µL, 1.5 mmol, 3.0 equiv) were added to the jar using a syringe. After the jar was closed without purging with inert gas, the jar was placed in the ball mill (Retsch MM 400, 1 h, 30 Hz). After grinding for 1 h, the jar was opened in air and was charged with cerium chloride (0.75 mmol, 1.5 equiv). The jar was then closed without purging with inert gas and was placed in the ball mill (Retsch MM 400, 10 min, 30 Hz). After grinding for 10 min, the jar was opened in air and was charged with (*E*)-4-phenylbut-3-en-2-one (**8**, 0.50 mmol). The jar was then closed without purging with inert gas and was placed in the ball mill (Retsch MM 400, 1 h, 30 Hz). After grinding for 1 h, the reaction mixture was quenched with a saturated aqueous solution of NH_4Cl and extracted with CH_2Cl_2 (30 mL×3). The solution was washed with brine and dried over Na_2SO_4. After the removal of the solvents under reduced pressure, ^{1}H NMR analysis of the resulting crude mixture was conducted to determine the yields of the 1,2-addition product **9** and the 1,4-addition product **10**.

The representative procedure is shown in below pictures (Supplementary Figures S4 and S5).

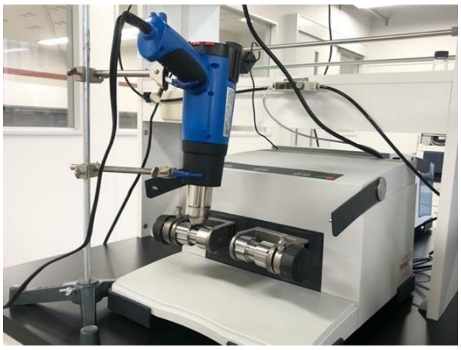

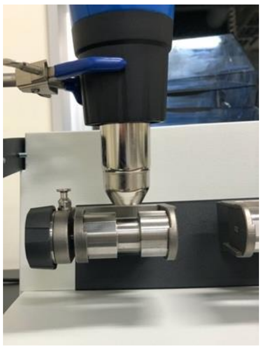

Supplementary Figure 4. Setting of the heat gun for conditions C and D. The heat gun was fixed with clamps and placed directly above the ball milling jar (distance between the heat gun and ball milling jar: *ca.* 1 cm).

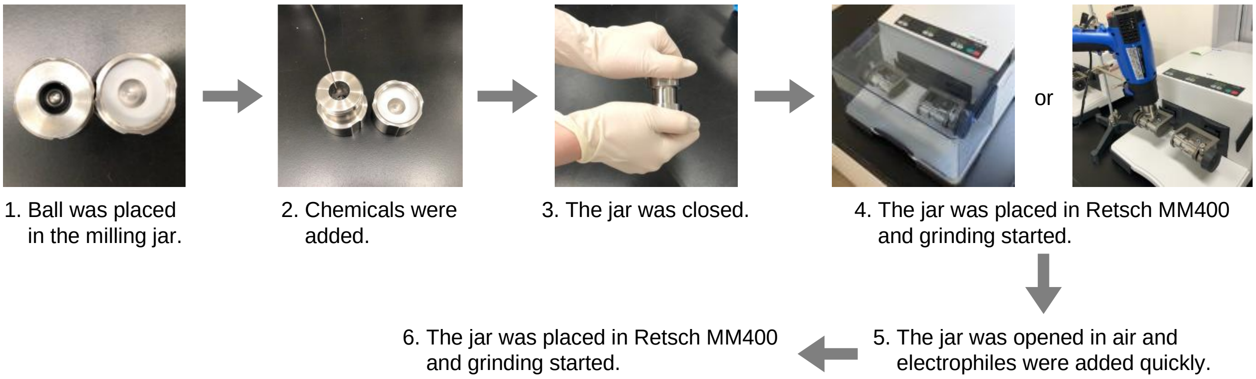

Supplementary Figure 5. Procedure of setting up the mechanochemical reactions.

Air Stability of the Grignard Reagent Prepared by Ball Mill

Electrophiles should be added as soon as possible after opening the jar. We conducted nucleophilic addition reactions after exposing the mechanochemically synthesized organomagnesium species to the air for 10–60 min, as shown below. The results showed that the yield of **3aa** decreased when the synthesized organomagnesium species were exposed to the air for 10 min or more. While benzene was detected after the exposure of the synthesized organomagnesium species to the air, byproducts derived from oxygen or carbon dioxide, such as phenol and benzoic acid, were not detected under the mechanochemical conditions. This result suggests that the decreased yield of the organomagnesium nucleophile is mainly caused by protonation by moisture during the exposure to air.

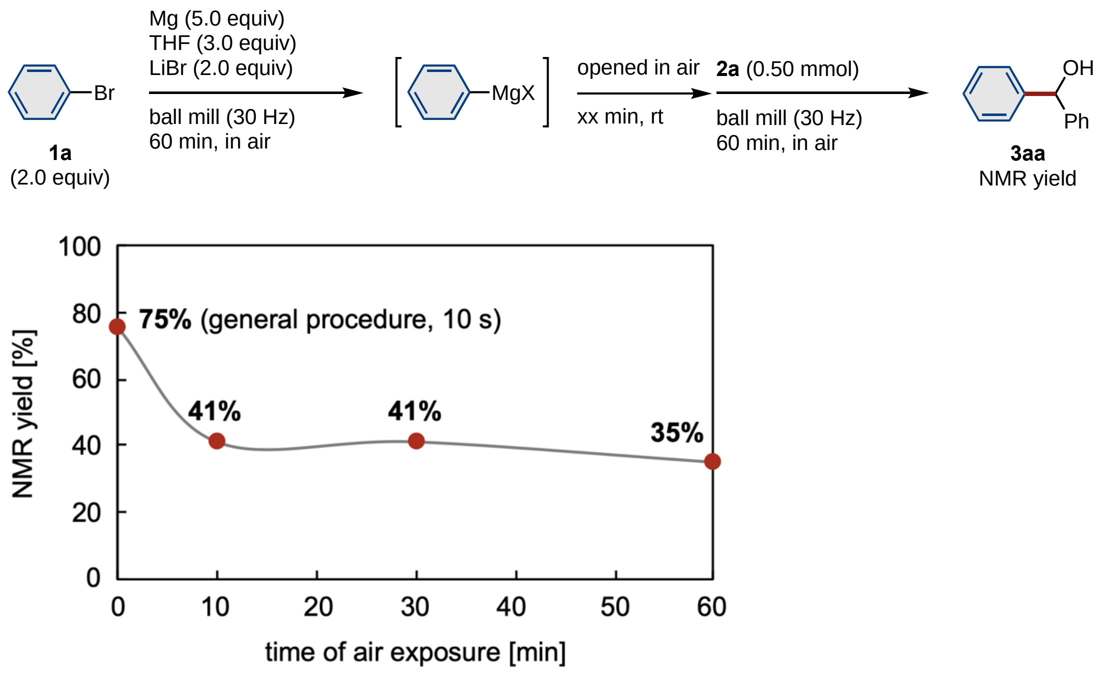

Supplementary Figure 6. Decreased yield of the nucleophilic addition product **3aa** after exposure of the mechanochemically synthesized organomagnesium species to the air.

We also examined the nucleophilic addition reactions when the mechanochemically synthesized organomagnesium species in the ball milling jar was kept closed in air at room temperature for 24 hours. This result showed that the organomagnesium reagents could be retained for several hours after their preparation if the jar was kept closed.

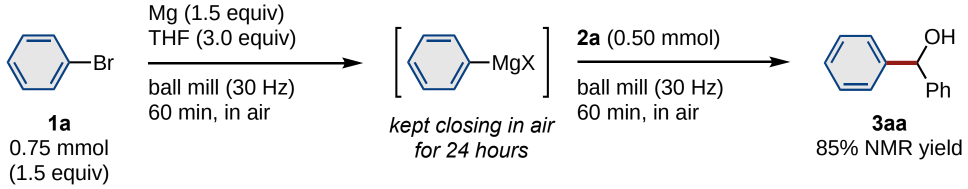

List of Substrates

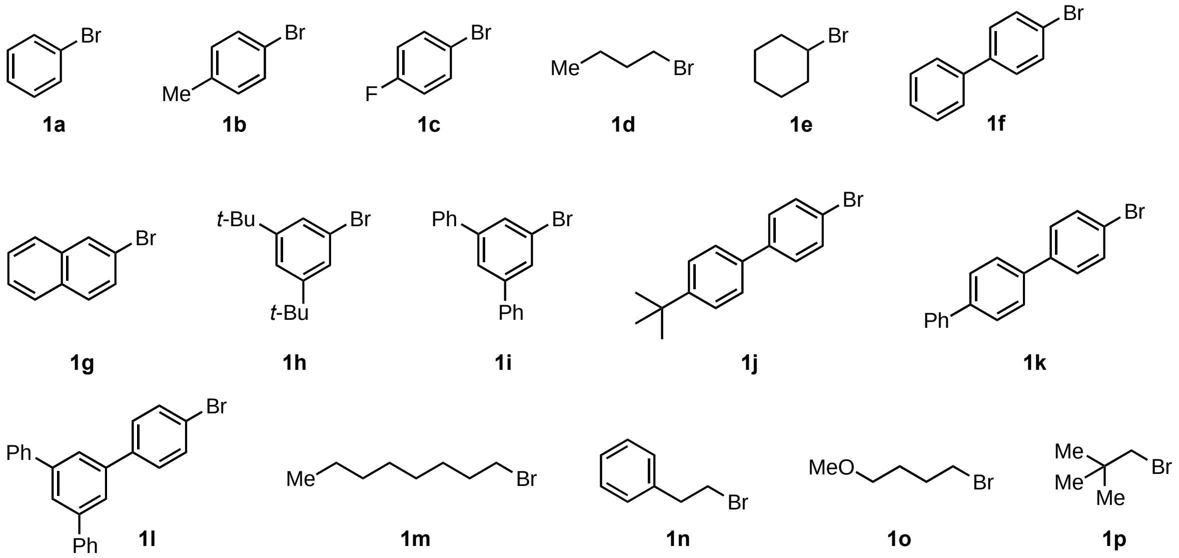

Supplementary Figure 7. List of organic bromides used in this study. All organic bromides were obtained from commercial suppliers and were used as received.

All aryl tosylates (**6a**–**6d**) were synthesized from the corresponding naphthols according to the reported procedure.[1] To a solution of naphthol (2.0 mmol) in THF (0.6 mL) was added 10wt% K_2CO_3 (3.8 mmol) or 15wt% NaOH (6.6 mmol) as an aqueous solution. After the resulting solution was cooled to 0 °C, a solution of TsCl (458 mg, 2.4 mmol for 10% K_2CO_3 or 385 mg, 2.02 mmol for 15% NaOH) in THF (1.4 mL) was slowly added over 15 min at 0 °C. After the addition of TsCl, the reaction mixture was stirred for 2 h at room temperature. EtOAc (8 mL) was added to the reaction mixture and the two-phase mixture was separated. The organic layer was washed with H_2O (3 mL) and dried over Na_2SO_4. The removal of the solvents under reduced pressure gave the corresponding pure aryl tosylate **6**. Trace amounts of TsCl were removed by washing the product with hexanes.

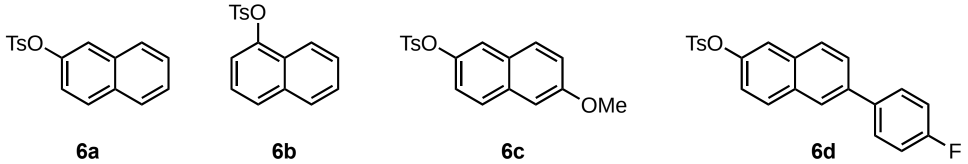

Supplementary Figure 8. List of aryl tosylates used in this study.

Thermography Measurements for Reaction Temperature inside Ball Mill Jars

The temperature inside the milling jar of the mechanochemical reaction was immediately confirmed by thermography after opening the jar. The crude mixtures were prepared under the following conditions: (A) 0.75 mmol of **1a**; 0.75 mmol of Mg turnings; 1.5 mmol of THF in a stainless-steel ball milling jar (5 mL) with a stainless-steel ball (10 mm); 30Hz; 1 h; (B) 1.0 mmol of **1a**; 2.5 mmol of Mg turnings; 1.5 mmol of THF in a stainless-steel ball milling jar (5 mL) with a stainless-steel ball (10 mm); 30Hz; 1 h; (C) 1.0 mmol of **1f**; 2.5 mmol of Mg turnings; 1.5 mmol of THF in a stainless-steel ball milling jar (5 mL) with a stainless-steel ball (10 mm); 30Hz; 1 h; preset temperature of heat gun: 110 °C. The obtained images (Fig. S9) showed that the temperatures were 33 °C, 29 °C, and 68 °C for the conditions A, B, and C, respectively.

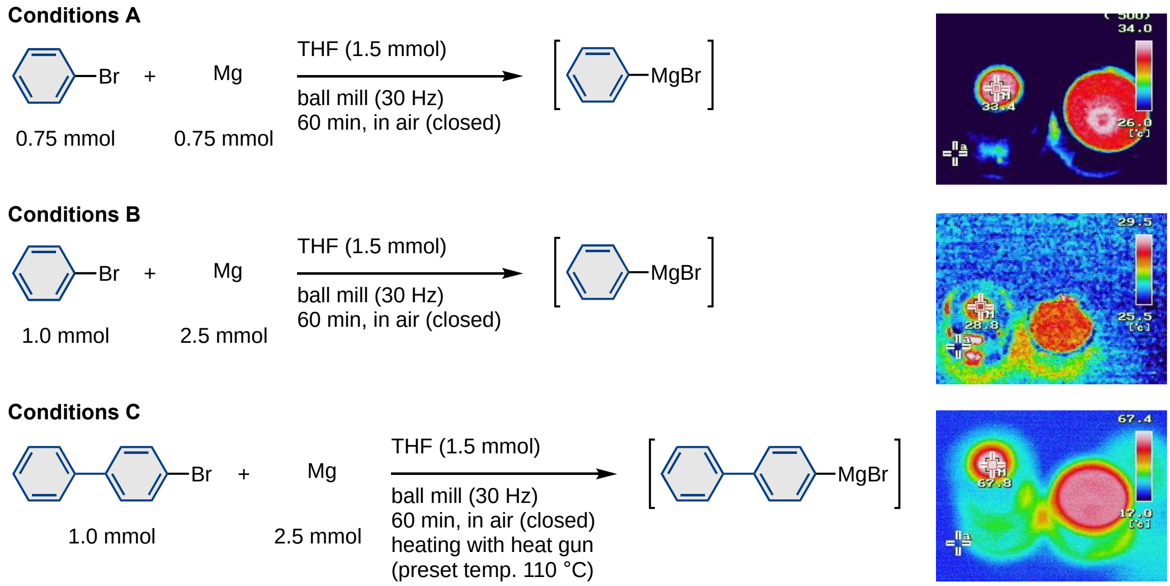

Supplementary Figure 9. Thermography measurements for temperature inside the milling jar after the reaction of organic halides and Mg.

Gram-Scale Reaction Procedures

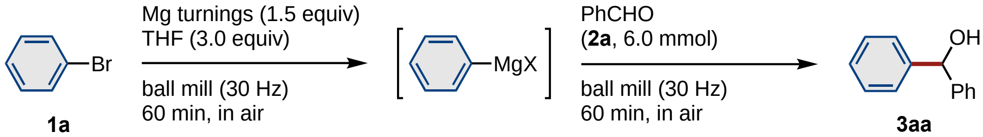

1a 9.0 mmol (1.5 equiv) → **3aa** 93% yield (1.03 g)

Mg turnings (216.1 mg, 9.0 mmol) were placed in a milling jar (10 mL) with two balls (15 mm, diameter) in air. Bromobenzene (**1a**, 1.424 g, 9.0 mmol) and THF (1.44 mL, 18 mmol) were added to the jar using a syringe. The jar was then closed without purging with inert gas, the jar was placed in the ball mill (Retsch MM 400, 60 min, 30 Hz). After grinding for 1 h, the jar was opened in air and was charged with a distilled benzaldehyde (**2a**, 636.7 mg, 6.0 mmol). The jar was then closed without purging with inert gas and was placed in the ball mill (Retsch MM 400, 60 min, 30 Hz). After grinding for 1 h, the reaction mixture was quenched with a saturated aqueous solution of NH_4Cl and extracted with CH_2Cl_2. The solution was dried over Na_2SO_4 and the solvents were removed under reduced pressure. The crude material was purified by flash column chromatography (SiO_2, hexane/ethyl acetate) to give the corresponding product **3aa** as a white powder (1.03 g, 5.6 mmol, 93% yield).

Optimization Studies

Supplementary Table 1. Optimization of the reaction conditions using aryl halides[a]

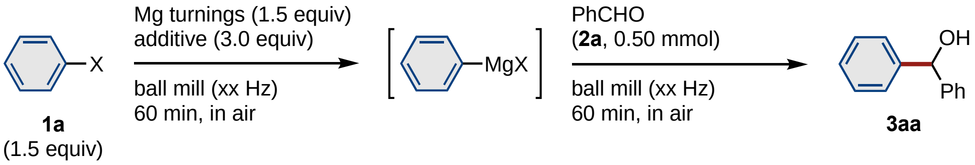

entry	halide	additive (volume)	milling frequency (Hz)	ball size (*Φ* mm)	yield (%)
1	**1a** (X = Br)	-	30	10	6
2	**1a**	THF (123 μL)	30	10	94
3	**1a**	Et_2O (155 μL)	30	10	79
4	**1a**	CPME (175 μL)	30	10	87
5	**1a**	MTBE (176 μL)	30	10	57
6	**1a**	1,4-dioxane (128 μL)	30	10	5
7	**1a**	hexane (200 μL)	30	10	<1
8	**1a**	toluene (160 μL)	30	10	1
9	**1a**	THF (61 μL)	30	10	47
10	**1a**	THF (183 μL)	30	10	90
11[b]	**1a**	THF (123 μL)	30	10	92
12	**1a**	THF (123 μL)	25	10	90
13	**1a**	THF (123 μL)	30	5	91
14[c]	**1a**	THF (123 μL)	30	5	52
15	**1a’** (X = I)	THF (123 μL)	30	10	14
16[d]	**1a’**	THF (123 μL)	30	10	74
17	**1a”** (X = Cl)	THF (123 μL)	30	10	84

[a]Reactions performed using Retsch MM400, stainless-steel milling jar (5 mL) and a stainless-steel ball. Conditions: **1a** (0.75 mmol), Mg (0.75 mmol), **2a** (0.50 mmol). Yields were determined by ^{1}H NMR analysis with dibromomethane as an internal standard. [b]Undistilled THF (purchased from FUJIFILM Wako Chemicals; product no. 206-05106; BHT is included as a stabilizer) was used. [c]Reactions performed using Retsch MM400, stainless-steel milling jar (1.5 mL) and a stainless-steel ball (diameter: 5 mm). [d]Conditions: **1a’** (1.0 mmol), Mg (2.5 mmol), **2a** (0.50 mmol).

Supplementary Table 2. Optimization of reaction using secondly alkyl bromide[a]

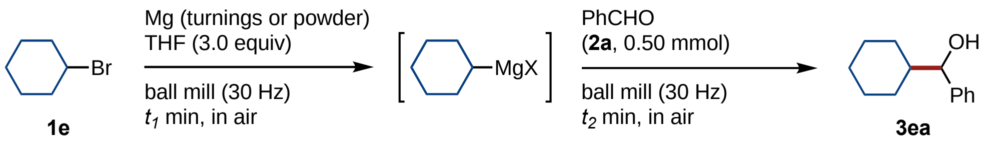

entry	**1e** (equiv)	Mg (equiv)	reaction time (min) first step	reaction time (min) second step	yield (%)
1	1.5	1.5 (turnings)	60	60	24
2	1.5	1.5 (powder)	60	60	30
3	1.5	1.5 (turnings)	60	60	26
4	1.8	2.5 (turnings)	60	60	23
5	1.8	2.5 (turnings)	90	60	53
6	1.8	2.5 (turnings)	120	60	43
7	2.0	2.5 (turnings)	90	60	74
8	2.0	2.5 (powder)	60	60	62
9	2.0	2.5 (powder)	90	60	56

[a]Reactions performed using Retsch MM400, stainless-steel milling jar (5 mL) and a stainless-steel ball (10 mm, diameter). Yields were determined by ^{1}H NMR analysis with dibromomethane as an internal standard.

Supplementary Table 3. Effect of halides[a]

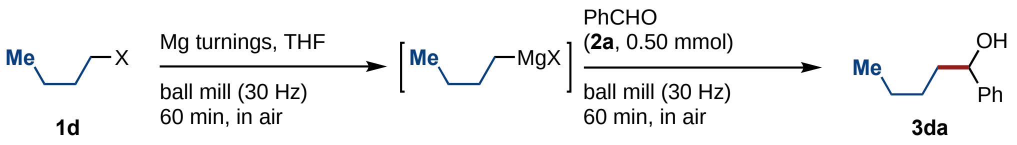

Conditions A
1d (0.75 mmol)
Mg (0.75 mmol)
THF (1.5 mmol)

Conditions B
1d (1.0 mmol)
Mg (2.5 mmol)
THF (1.5 mmol)

entry	halide	conditions	yield (%)
1	**1d** (X = Br)	A	90
2	**1d**	B	92
3	**1d'** (X = I)	A	45
4	**1d'**	B	68
5	**1d"** (X = Cl)	A	82
6	**1d"**	B	84

[a]Reactions performed using Retsch MM400, stainless-steel milling jar (5 mL) and a stainless-steel ball (10 mm, diameter). Conditions A: **1d** (0.75 mmol), Mg (0.75 mmol), THF (1.5 mmol), **2d** (0.50 mmol). Conditions B: **1d** (1.0 mmol), Mg (2.5 mmol), THF (1.5 mmol), **2d** (0.50 mmol). Yields were determined by ^{1}H NMR analysis with dibromomethane as an internal standard.

Supplementary Table 4. Effect of the addition of lithium salts[a]

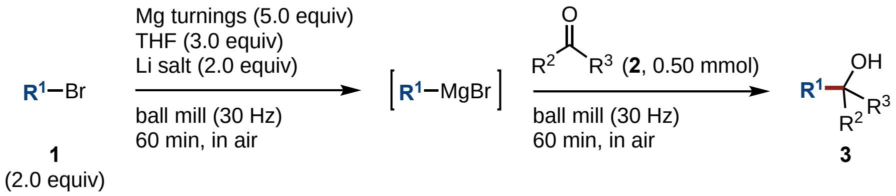

entry	halide	electrophile	Li salt	yield (%)
1	**1a**	**2e**	-	68
2	**1a**	**2e**	LiBr	82
3	**1b**	**2a**	-	70
4	**1b**	**2a**	LiBr	65
5	**1c**	**2a**	LiBr	40
6	**1c**	**2a**	LiCl	81
7	**1d**	**2a**	-	92
8	**1d**	**2a**	LiBr	28

[a]Reactions performed using Retsch MM400, stainless-steel milling jar (5 mL) and a stainless-steel ball (10 mm, diameter). Lithium salts were dried under reduced pressure at 150 °C for 3 hours before use. Yields were determined by ^{1}H NMR analysis with dibromomethane as an internal standard.

Reactions Under the Solvent-less Conditions in a Test Tube

Mg turnings (18.2 mg, 0.75 mmol) were placed in an oven-dried reaction vial. After being sealed with a screw cap containing a Teflon-coated rubber septum, the vial was connected to a nitrogen line through a needle. An organic bromide (**1**, 0.75 mmol) and THF (123 μL, 1.5 mmol) were added to the vial and then the reaction mixture was stirred at room temperature. After 1 h, a distilled aldehyde (**2a** or **2f**, 0.50 mmol) was added and the reaction mixture was stirred for 1 h at room temperature. The reaction mixture was quenched with a saturated aqueous solution of NH_4Cl and extracted with CH_2Cl_2 (30 mL×3). The solution was washed with brine and dried over Na_2SO_4. After the removal of the solvents under reduced pressure, the resulting crude mixture was analyzed by ^{1}H NMR with dibromomethane as an internal standard to determine the NMR yield of the product **3**.

Even though the reaction mixtures are liquid, we confirmed that the organomagnesium nucleophiles were not generated efficiently in a test tube using magnetic stirring under the optimized conditions shown in Figure S10. These results suggest that activation of magnesium metal by strong mechanical agitation in a ball mill seems to be essential for the efficient formation of the organomagnesium species.

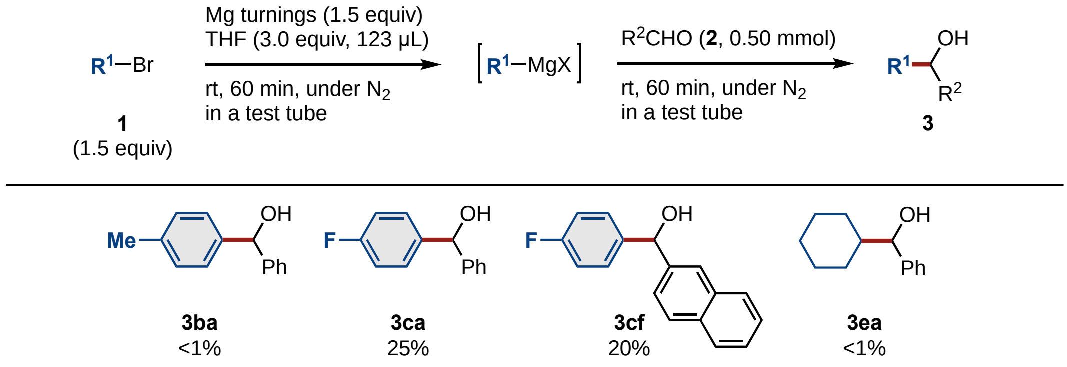

Supplementary Figure 10. Reactions under solvent-less conditions without strong mechanical agitation.

Scope of organic halides and electrophiles

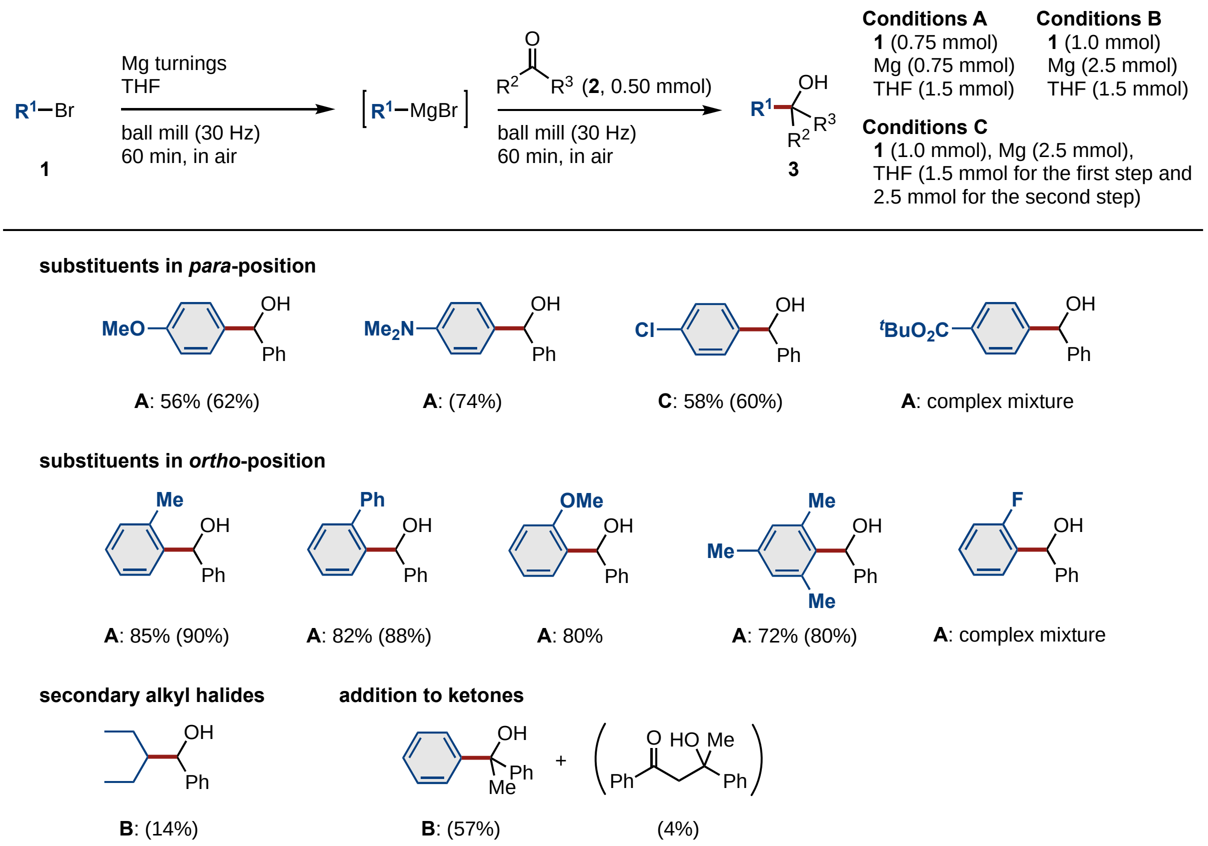

Supplementary Figure 11. Scope of the mechanochemical synthesis of organomagnesium nucleophiles from various organic bromides and their nucleophilic addition to aldehydes and ketones. Isolated yields are reported as percentages. Proton NMR integrated yields are shown in parentheses.

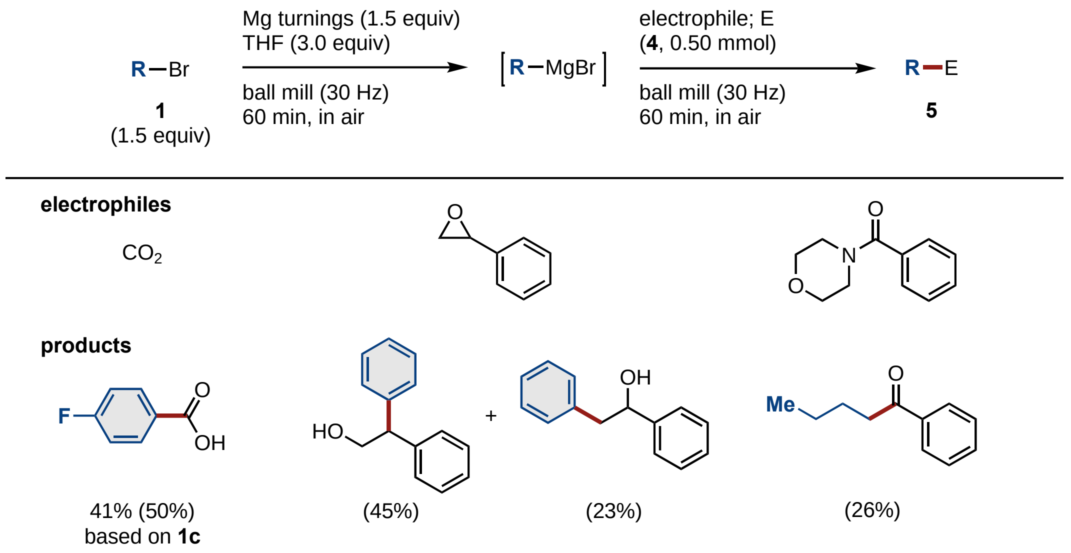

Supplementary Figure 12. Nucleophilic addition to various electrophiles under the optimized conditions E. Isolated yields are reported as percentages. Proton NMR integrated yields are shown in parentheses.

Radical clock experiments

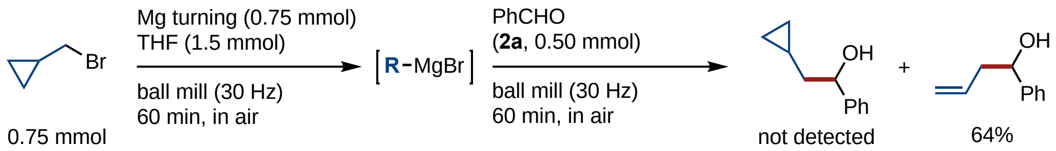

Mg turnings (18.3 mg, 0.75 mmol) and (bromomethyl)cyclopropane (100.5 mg, 0.75 mmol) were placed in a milling jar (5 mL) with a ball (diameter: 10 mm) in air. THF (123 μL, 1.5 mmol) was added to the jar using a syringe. After the jar was closed without purging with inert gas, the jar was placed in the ball mill (Retsch MM 400, 60 min, 30 Hz). After grinding for 1 h, the jar was opened in air, and charged with benzaldehyde (53.2 mg, 0.50 mmol). The jar was then closed without purging with inert gas and placed in the ball mill (Retsch MM 400, 60 min, 30 Hz). After grinding for 60 min, the reaction mixture was quenched with a saturated aqueous solution of NH_4Cl and extracted with CH_2Cl_2 (3×30 mL). The solution was washed with brine and dried over Na_2SO_4. After the removal of the solvents under reduced pressure, the resulting crude mixture was analyzed by ^{1}H NMR spectroscopy with 4-dimethylaminopyridine as an internal standard to determine the NMR yield.

Deuteration Experiments

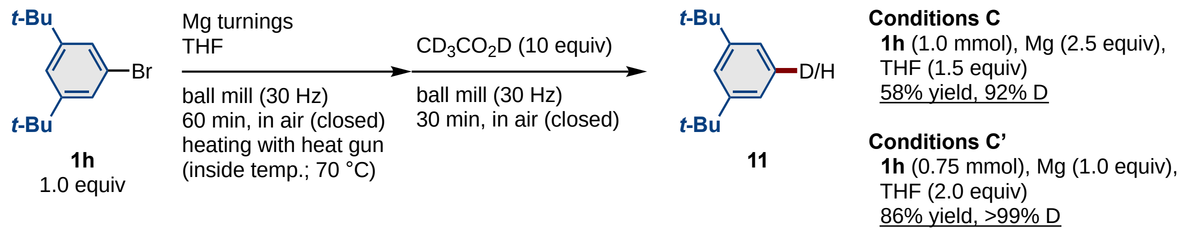

Mg turnings and 1-bromo-3,5-di-*tert*-butylbenzene (**1h**) were placed in a milling jar (5 mL) with a ball (diameter: 10 mm) in air. THF was the added to the jar using a syringe. After the jar was closed without purging with inert gas, the jar was placed in the ball mill (Retsch MM 400, 60 min, 30 Hz). A heat gun was set approximately 1 cm above the jar and was turned on (preset temperature: 110 °C, internal temperature: *ca*. 70 °C). After grinding for 1 h, the jar was cooled to room temperature for 20 minutes, opened in air, and charged with acetic acid-d_4 (10 equiv). The jar was then closed without purging with inert gas and was placed in the ball mill (Retsch MM 400, 30 min, 30 Hz). After grinding for 30 min, the reaction mixture was quenched with a saturated aqueous solution of $NaHCO_3$ and extracted with CH_2Cl_2 (30 mL×3). The solution was washed with brine and dried over Na_2SO_4. After the removal of the solvents under reduced pressure, the resulting crude mixture was analyzed by ^{1}H NMR with dibromomethane as an internal standard to determine the NMR yield of 1,3-di-*tert*-butylbenzene (**11**).

X-Ray Absorption Fine Structure (XAFS) Analysis of 12

Mg K-edge NEXAFS measurements were performed at the soft X-ray beamline BL2A of UVSOR-III synchrotron.[2] The mechanochemically-prepared gummy organomagnesium sample of **12** was pasted on high purity indium foil and fixed onto a copper sample holder. For the sample preparation of solution-phase-prepared **12**, 1.0 M of THF solution of **12**[3] without $MgBr_2$ precipitation dropped and dried on high purity indium foil. The sample holder was fixed on a linear and rotatable manipulator and then installed into a vacuum chamber, which was evacuated to a pressure of less than 1×10^{-6} Pa. The sample preparation and install processes were carefully performed under argon or nitrogen atmospheres. The Mg K-edge NEXAFS spectra (1250−1400 eV) were taken in total electron yields (TEYs) by measuring a sample drain current. The energy resolution of the incident soft X-rays at the Mg K-edge is set to 0.2 eV in the range from 1300 to 1330 eV and 1.0 eV in the range from 1250 to 1300 eV and from 1330 to 1400 eV.

C K-edge NEXAFS measurements were performed at the soft X-ray beamline BL3U of the UVSOR-III synchrotron.[4] All the organomagnesium samples were fixed onto a stainless sample holder and installed into a vacuum chamber 1×10^{-6} Pa in the same manner as for the Mg K-edge NEXAFS measurement. The C K-edge NEXASF spectra (280−300 eV) were taken in total electron yields (TEYs) by measuring a sample drain current. The energy resolution of the incident soft X-rays at the C K-edge is set to 0.05 eV in the range from 283 to 293 eV and 0.2 eV in the range from 280 to 283 eV and from 293 to 300 eV.

The data processing for the subtraction of background of indium foil, baseline correction, and normalization of the resulting spectra were performed by using Athena program.[5]

Theoretical Study to Predict Structures of Magnesium-Based Carbon Nucleophiles

Experimentally, 2.0 equivalents of THF for aryl halides was added to the reaction system as an additive. Though the ligation of solvent molecule to Mg atom is known for the Grignard reagent in solution,[6–8] how does the existence of THF affect the structure of the Grignard reagent in solid-state remains unclear so far. To understand the impact of the THF addition, we performed a theoretical study using model complexes $(RMgBr)_4$-nTHF (of which R = Phenyl (Ph) and Ethyl (Et) groups and n = 0, 2, 4, 6 and 8). To locate the most stable structures of these complexes, minimum-only sampling calculations were conducted using the SC-AFIR method[9, 10] at the GFN-xTB[11, 12] level of theory under the ORCA environment,[13, 14] where the model collision energy parameter Gamma of the AFIR method was set as 200 kJ/mol. As the results, 1623 and 1420 local minimum structures were obtained for $(PhMgBr)_4$ and $(EtMgBr)_4$, respectively. The most stable structures were then selected for the second round of sampling calculations by adding 2, 4, 6 and 8 THF molecules, respectively. For $(PhMgBr)_4$-nTHF, 1511, 1426, 948 and 750 local minimum structures were eventually provided for n = 2, 4, 6, and 8 respectively. In the case of $(EtMgBr)_4$-nTHF, 1786, 1501, 1065 and 837 local minimum structures were located. Stable structures acquired in the sampling calculations were subsequently fully re-optimized at B3LYP-D3/Def2SVP level of theory[15–20] using the RIJCOSX approximation[21, 22] (as implemented in the ORCA package). An auxiliary basis set, named def2/J,[20] was employed in the RIJCOSX approximation. Frequency calculations were also carried out at the same level of theory to confirm that all the optimized structures were minima. Since the reaction was conducted in the solid state, no solvation model is adopted in this work. All the Gibbs energies quoted below were evaluated at 298.15 K and 1 atm.

As shown in Figure S11, the most stable $(PhMgBr)_4$ and $(EtMgBr)_4$ isomers were found to have cubic structures. In both R = Ph and Et cases, the additional THF molecules coordinated to the Mg atoms, and such a coordination eventually induced the scission of Mg-Br bonds in the cubic structure. In other words, the coordination of THF molecules transformed the cubic structures into the more opened ones. Given that, it is supposed that one of the impacts of the THF addition is to transform the $(RMgBr)_m$ species from an unreactive, closed form to the more reactive and open form. Further exploration of the stable structures of $(RMgBr)_m$-nTHF (for m = 6 and more) are currently under progress in our laboratory, aiming at providing a better understanding of the structures of the Grignard reagents in solid-state through the comparisons between the theoretical study and the experimental NEXAFS spectra.

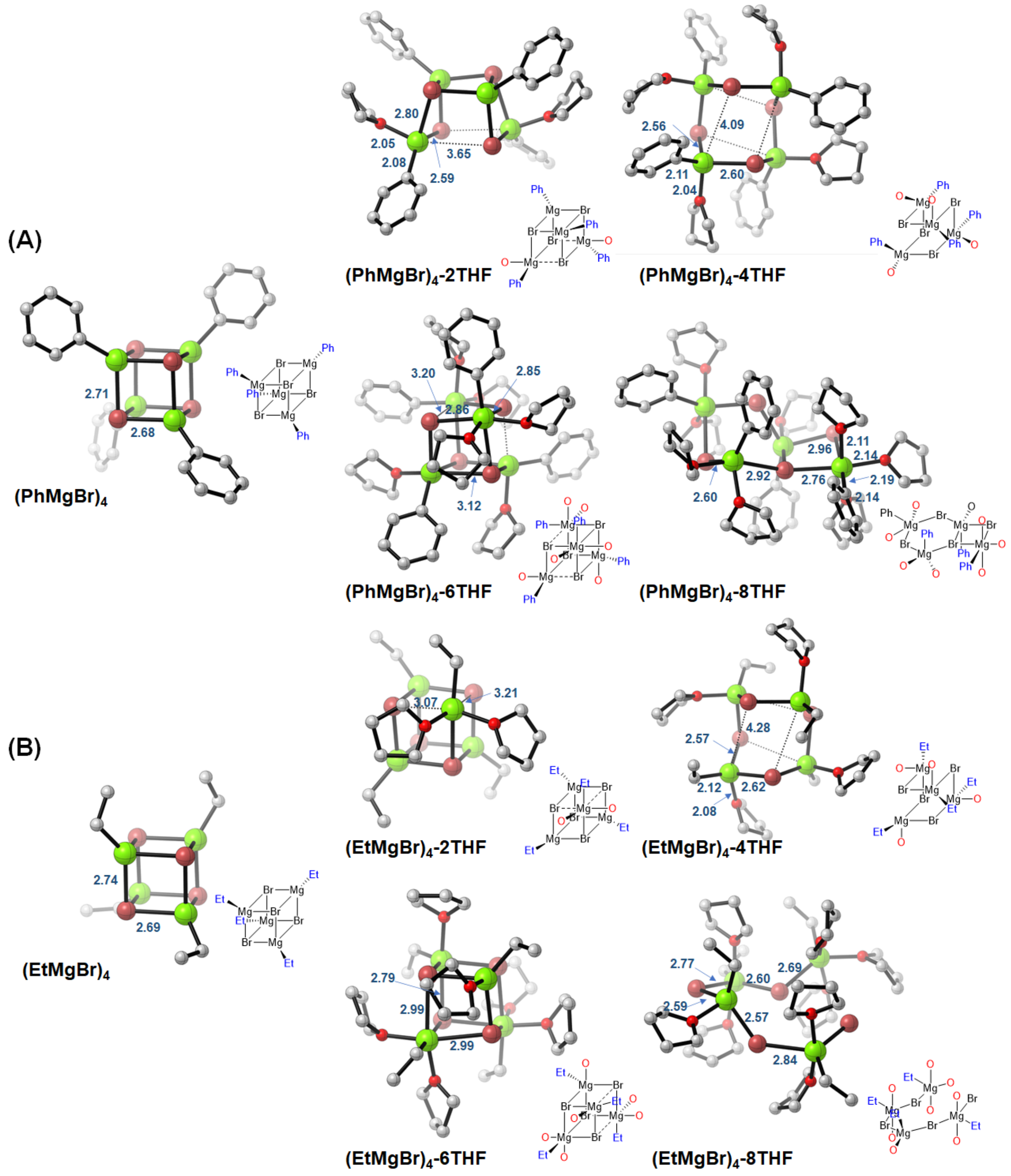

Supplementary Figure 13. Optimized structures of (PhMgBr)$_4$-nTHF (A) and (EtMgBr)$_4$-nTHF (B) at the B3LYP-D3/Def2SVP level, where n = 0, 2, 4, 6 and 8 (note that all H atoms are omitted for clarity). The addition of THF molecules transformed the cubic structures obtained at n = 0 to the more opened structures by the coordination to Mg atoms and the Mg-Br bond scission associated.

Product Characterizations

Diphenylmethanol (3aa).

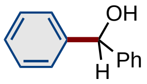

3aa

Conditions A: The reaction was conducted with **1a** (79 µL, 0.75 mmol) and **2a** (53.7 mg, 0.50 mmol). The product **3aa** was obtained in 84% yield (77.9 mg, 0.42 mmol) as a white powder.

Conditions B: The reaction was conducted with **1a** (105 µL, 1.0 mmol), lithium bromide (86.9 mg, 1.0 mmol), and **2a** (53.2 mg, 0.50 mmol). The product **3aa** was obtained in 64% yield (59.1 mg, 0.32 mmol) as a white powder. ^{1}H and ^{13}C NMR of the product **3aa** were in agreement with the literature.[23]

^{1}H NMR (400 MHz, $CDCl_3$, δ): 2.22 (d, J = 3.6 Hz, 1H), 5.86 (d, J = 3.6 Hz, 1H), 7.27 (tt, J = 1.7, 7.1 Hz, 2H), 7.31–7.41 (m, 8H). ^{13}C NMR (101 MHz, $CDCl_3$, δ): 76.1 (*C*H), 126.5 (*C*H), 127.5 (*C*H), 128.4 (*C*H), 143.7 (*C*). HRMS-EI (m/z): [M]$^+$ calcd for $C_{13}H_{12}O$, 184.0888; found, 184.0885.

1,3-Diphenylpropan-1-ol (3ab).

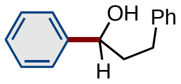

3ab

Conditions A: The reaction was conducted with **1a** (79 µL, 0.75 mmol) and **2b** (68.9 mg, 0.51 mmol). The product **3ab** was obtained in 70% yield (76.5 mg, 0.36 mmol) as a colorless oil.

Conditions B: The reaction was conducted with **1a** (105 µL, 1.0 mmol), lithium bromide (86.9 mg, 1.0 mmol for the first step), and **2b** (66.9 mg, 0.50 mmol). The product **3ab** was obtained in 63% yield (66.8 mg, 0.32 mmol) as a colorless oil. ^{1}H and ^{13}C NMR of the product **3ab** were in agreement with the literature.[23]

^{1}H NMR (400 MHz, $CDCl_3$, δ): 1.83 (d, J = 3.2 Hz, 1H), 1.99–2.20 (m, 2H), 2.62–2.81 (m, 2H), 4.68–4.72 (m, 1H), 7.17–7.32 (m, 6H), 7.36 (d, J = 4.4 Hz, 4H). ^{13}C NMR (100 MHz, $CDCl_3$, δ): 32.0 (*C*H_2), 40.4 (*C*H_2), 73.8 (*C*H), 125.8 (*C*H), 125.9 (*C*H), 127.6 (*C*H), 128.3 (*C*H), 128.39 (*C*H), 128.45 (*C*H), 141.7 (*C*), 144.5 (*C*). HRMS-EI (m/z): [M]$^+$ calcd for $C_{15}H_{16}O$, 212.1201; found, 212.1204.

Cyclohexyl(phenyl)methanol (3ac).

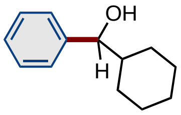

3ac

Conditions A: The reaction was conducted with **1a** (79 µL, 0.75 mmol) and **2c** (56.8 mg, 0.51 mmol). The product **3ac** was obtained in 73% yield (70.6 mg, 0.37 mmol) as a white powder.

Conditions B: The reaction was conducted with **1a** (105 µL, 1.0 mmol), lithium chloride (42.6 mg, 1.0 mmol for the first step), and **2c** (56.3 mg, 0.50 mmol). The product **3ac** was obtained in 58% yield (55.7 mg, 0.29 mmol) as a white powder. ^{1}H and ^{13}C NMR of the product **3ac** were in agreement with the literature.[23]

^{1}H NMR (392 MHz, $CDCl_3$, δ): 0.87–1.29 (m, 5H), 1.34–1.41 (m, 1H), 1.55–1.70 (m, 3H), 1.73–1.80 (m, 1H), 1.81 (d, J = 3.1 Hz, 1H), 1.95–2.03 (m, 1H), 4.37 (dd, J = 2.7, 7.1 Hz, 1H), 7.24–7.37 (m, 5H). ^{13}C NMR (101 MHz, $CDCl_3$, δ): 25.96 (CH_2), 26.04 (CH_2), 26.4 (CH_2), 28.8 (CH_2), 29.2 (CH_2), 44.9 (CH), 79.3 (CH), 126.6 (CH), 127.4 (CH), 128.1 (CH), 143.6 (C). HRMS-EI (m/z): $[M]^+$ calcd for $C_{13}H_{18}O$, 190.1358; found, 190.1360.

1,1-Diphenylpropan-1-ol (3ad).

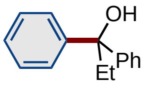

3ad

Conditions A: The reaction was conducted with **1a** (79 µL, 0.75 mmol) and **2d** (67.3 mg, 0.50 mmol). The product **3ad** was obtained in 82% yield (87.7 mg, 0.41 mmol) as a white powder.

Conditions B: The reaction was conducted with **1a** (105 µL, 1.0 mmol), lithium bromide (87.0 mg, 1.0 mmol for the first step), and **2d** (66.8 mg, 0.50 mmol). The product **3ad** was obtained in 45% yield (47.8 mg, 0.23 mmol) as a white powder. ^{1}H and ^{13}C NMR of the product **3ad** were in agreement with the literature.[24]

^{1}H NMR (400 MHz, $CDCl_3$, δ): 0.89 (t, J = 7.4 Hz, 3H), 2.06 (s, 1H), 2.33 (q, J = 7.3 Hz, 2H), 7.22 (tt, J = 1.5, 7.3 Hz, 2H), 7.31 (t, J = 7.4 Hz, 4H), 7.40–7.44 (m, 4H). ^{13}C NMR (101 MHz, $CDCl_3$, δ): 8.1 (CH_3), 34.4 (CH_2), 78.4 (C), 126.0 (CH), 126.7 (CH), 128.1 (CH), 146.8 (C). HRMS-ESI (m/z): $[M{-}H]^+$ calcd for $C_{15}H_{15}O$, 211.1128; found, 211.1126.

2,4-Diphenylbutan-2-ol (3ae).

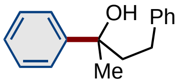

3ae

Conditions A: The reaction was conducted with **1a** (79 µL, 0.75 mmol) and **2e** (74.1 mg, 0.50 mmol). The product **3ae** was obtained in 79% yield (89.5 mg, 0.40 mmol) as a white powder.

Conditions B: The reaction was conducted with **1a** (105 µL, 1.0 mmol), lithium bromide (86.8 mg, 1.0 mmol for the first step), and **2e** (74.2 mg, 0.50 mmol). The product **3ae** was obtained in 69% yield (77.7 mg, 0.34 mmol) as a white powder. ^{1}H and ^{13}C NMR of the product **3ae** were in agreement with the literature.[25]

^{1}H NMR (401 MHz, $CDCl_3$, δ): 1.62 (s, 3H), 1.73 (s, 1H), 2.07–2.20 (m, 2H), 2.40–2.49 (m, 1H), 2.58–2.67 (m, 1H), 7.12 (d, J = 7.2 Hz, 2H), 7.16 (dt, J = 1.7, 7.3 Hz, 1H), 7.22–7.29 (m, 3H), 7.34–7.40 (m, 2H), 7.45–7.51 (m, 2H). ^{13}C NMR (101 MHz, $CDCl_3$, δ): 30.38 (CH_2), 30.43 (CH_3), 45.9 (CH_2), 74.6 (C), 124.7 (CH), 125.7 (CH), 126.6 (CH), 128.19 (CH), 128.24 (CH), 128.3 (CH), 142.2 (C), 147.5 (C). HRMS-EI (m/z): $[M]^+$ calcd for $C_{16}H_{18}O$, 226.1358; found, 226.1358.

Naphthalen-2-yl(phenyl)methanol (3af).

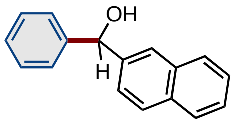

3af

Conditions A: The reaction was conducted with **1a** (79 μL, 0.75 mmol) and **2f** (78.1 mg, 0.50 mmol). The product **3af** was obtained in 73% yield (86.0 mg, 0.37 mmol) as a white powder.

Conditions B: The reaction was conducted with **1a** (157.4 mg, 105 μL, 1.0 mmol) and **2f** (78.1 mg, 0.50 mmol). The product **3af** was obtained in 73% yield (85.1 mg, 0.36 mmol) as a white powder. ^{1}H and ^{13}C NMR of the product **3af** were in agreement with the literature.[23]

^{1}H NMR (400 MHz, $CDCl_3$, δ): 2.54 (d, *J* = 3.2 Hz, 1H), 5.90 (d, *J* = 2.8 Hz, 1H), 7.23 (tt, *J* = 1.9, 7.1 Hz, 1H), 7.27–7.33 (m, 2H), 7.34–7.40 (m, 3H), 7.41–7.49 (m, 2H), 7.74 (d, *J* = 8.8 Hz, 1H), 7.75–7.81 (m, 2H), 7.83 (s, 1H). ^{13}C NMR (101 MHz, $CDCl_3$, δ): 76.2 (*C*H), 124.7 (*C*H), 124.9 (*C*H), 125.9 (*C*H), 126.1 (*C*H), 126.6 (*C*H), 127.59 (*C*H), 127.61 (*C*H), 128.0 (*C*H), 128.2 (*C*H), 128.5 (*C*H), 132.8 (*C*), 133.2 (*C*), 141.0 (*C*), 143.5 (*C*). HRMS-EI (m/z): $[M]^+$ calcd for $C_{17}H_{14}O$, 234.1045; found, 234.1037.

Phenyl(*p*-tolyl)methanol (3ba).

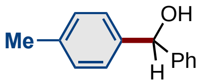

3ba

Conditions A: The reaction was conducted with **1b** (128.3 mg, 0.75 mmol) and **2a** (53.5 mg, 0.50 mmol). The product **3ba** was obtained in 86% yield (86.1 mg, 0.43 mmol) as a white powder.

Conditions B: The reaction was conducted with **1b** (170.5 mg, 1.0 mmol), THF (1.5 mmol for the first step, 0.5 mmol for the second step), and **2a** (53.2 mg, 0.50 mmol). The product **3ba** was obtained in 56% yield (55.4 mg, 0.28 mmol) as a white powder. ^{1}H and ^{13}C NMR of the product **3ba** were in agreement with the literature.[23]

^{1}H NMR (400 MHz, $CDCl_3$, δ): 2.15 (d, *J* = 3.6 Hz, 1H), 2.33 (s, 3H), 5.83 (d, *J* = 3.6 Hz, 1H), 7.15 (d, *J* = 7.6 Hz, 2H), 7.23–7.29 (m, 3H), 7.31–7.40 (m, 4H). ^{13}C NMR (100 MHz, $CDCl_3$, δ): 21.1 (CH_3), 76.0 (*C*H), 126.4 (*C*H), 126.5 (*C*H), 127.4 (*C*H), 128.4 (*C*H), 129.1 (*C*H), 137.2 (*C*), 140.9 (*C*), 143.9 (*C*). HRMS-EI (m/z): $[M]^+$ calcd for $C_{14}H_{14}O$, 198.1045; found, 198.1048.

3-Phenyl-1-(*p*-tolyl)propan-1-ol (3bb).

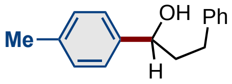

3bb

Conditions A: The reaction was conducted with **1b** (128.3 mg, 0.75 mmol) and **2b** (67.8 mg, 0.51 mmol). The product **3bb** was obtained in 70% yield (80.5 mg, 0.36 mmol) as a colorless oil.

Conditions B: The reaction was conducted with **1b** (171.0 mg, 1.0 mmol) and **2b** (67.3 mg, 0.50 mmol). The product **3bb** was obtained in 42% yield (48.1 mg, 0.21 mmol) as a colorless oil. ^{1}H and ^{13}C NMR of the

product **3bb** were in agreement with the literature.[26]

^{1}H NMR (392 MHz, $CDCl_3$, δ): 1.77 (d, *J* = 3.1 Hz, 1H), 1.97–2.19 (m, 2H), 2.35 (s, 3H), 2.61–2.79 (m, 2H), 4.66 (sept, *J* = 2.7 Hz, 1H), 7.14–7.31 (m, 9H). ^{13}C NMR (99 MHz, $CDCl_3$, δ): 21.1 (CH_3), 32.0 (CH_2), 40.3 (CH_2), 73.7 (*C*H), 125.8 (*C*H), 125.9 (*C*H), 128.3 (*C*H), 128.4 (*C*H), 129.1 (*C*H), 137.3 (*C*), 141.5 (*C*), 141.8 (*C*). HRMS-EI (m/z): $[M]^+$ calcd for $C_{16}H_{18}O$, 226.1358; found, 226.1361.

Cyclohexyl(*p*-tolyl)methanol (3bc).

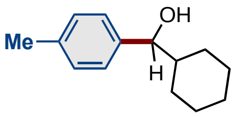

3bc

Conditions A: The reaction was conducted with **1b** (128.3 mg, 0.75 mmol) and **2c** (56.4 mg, 0.50 mmol). The product **3bc** was obtained in 62% yield (64.0 mg, 0.31 mmol) as a white powder.

Conditions B: The reaction was conducted with **1b** (172.0 mg, 1.0 mmol), THF (1.5 mmol for the first step, 0.5 mmol for the second step), and **2c** (56.3 mg, 0.50 mmol). The product **3bc** was obtained in 55% yield (56.8 mg, 0.28 mmol) as a white powder. ^{1}H and ^{13}C NMR of the product **3bc** were in agreement with the literature.[27]

^{1}H NMR (400 MHz, $CDCl_3$, δ): 0.86–1.28 (m, 5H), 1.33–1.40 (m, 1H), 1.57–1.69 (m, 3H), 1.73–1.81 (m, 2H), 1.97–2.03 (m, 1H), 2.34 (s, 3H), 4.33 (dd, *J* = 3.0, 7.0 Hz, 1H), 7.13–7.21 (m, 4H). ^{13}C NMR (100 MHz, $CDCl_3$, δ): 21.1 (CH_3), 26.0 (CH_2), 26.1 (CH_2), 26.4 (CH_2), 28.9 (CH_2), 29.3 (CH_2), 44.8 (*C*H), 79.2 (*C*H), 126.5 (*C*H), 128.8 (*C*H), 136.9 (*C*), 140.6 (*C*). HRMS-EI (m/z): $[M]^+$ calcd for $C_{14}H_{20}O$, 204.1514; found, 204.1513.

1-Phenyl-1-(*p*-tolyl)propan-1-ol (3bd).

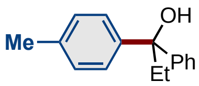

3bd

Conditions A: The reaction was conducted with **1b** (128.3 mg, 0.75 mmol) and **2d** (67.5 mg, 0.50 mmol). The product **3bd** was obtained in 74% yield (84.0 mg, 0.37 mmol) as a colorless oil.

Conditions B: The reaction was conducted with **1b** (171.2 mg, 1.0 mmol) and **2d** (67.0 mg, 0.50 mmol). The product **3bd** was obtained in 64% yield (72.3 mg, 0.32 mmol) as a colorless oil. ^{1}H and ^{13}C NMR of the product **3bd** were in agreement with the literature.[24]

^{1}H NMR (400 MHz, $CDCl_3$, δ): 0.88 (t, *J* = 7.6 Hz, 3H), 2.03 (s, 1H), 2.31 (q, *J* = 7.3 Hz, 2H), 2.32 (s, 3H), 7.12 (d, *J* = 8.4 Hz, 2H), 7.21 (t, *J* = 7.2 Hz, 1H), 7.28–7.33 (m, 4H), 7.41 (d, *J* = 7.2 Hz, 2H). ^{13}C NMR (101 MHz, $CDCl_3$, δ): 8.1 (CH_3), 20.9 (CH_3), 34.4 (CH_2), 78.3 (*C*), 126.0 (*C*H), 126.6 (*C*H), 128.0 (*C*H), 128.7 (*C*H), 136.3 (*C*), 144.0 (*C*), 147.0 (*C*). HRMS-ESI (m/z): $[M–H]^+$ calcd for $C_{16}H_{17}O$, 225.1285; found, 225.1284.

4-Phenyl-2-(*p*-tolyl)butan-2-ol (3be).

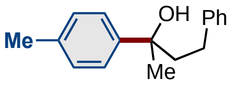

3be

Conditions A: The reaction was conducted with **1b** (128.3 mg, 0.75 mmol) and **2e** (74.4 mg, 0.50 mmol). The product **3be** was obtained in 69% yield (83.7 mg, 0.35 mmol) as a colorless oil.

Conditions B: The reaction was conducted with **1b** (170.1 mg, 1.0 mmol) and **2e** (73.9 mg, 0.50 mmol). The product **3be** was obtained in 60% yield (71.9 mg, 0.30 mmol) as a colorless oil.

^{1}H NMR (400 MHz, $CDCl_3$, δ): 1.60 (s, 3H), 1.72 (s, 1H), 2.04–2.18 (m, 2H), 2.36 (s, 3H), 2.41–2.49 (m, 1H), 2.56–2.66 (m, 1H), 7.10–7.20 (m, 5H), 7.24 (d, *J* = 7.2 Hz, 2H), 7.36 (dt, *J* = 2.1, 8.5 Hz, 2H). ^{13}C NMR (101 MHz, $CDCl_3$, δ): 20.9 (CH_3), 30.4 (CH_3), 30.4 (CH_2), 45.8 (CH_2), 74.6 (*C*), 124.6 (*C*H), 125.7 (*C*H), 128.26 (*C*H), 128.29 (*C*H), 128.9 (*C*H), 136.1 (*C*), 142.3 (*C*), 144.5 (*C*). HRMS-EI (m/z): $[M]^+$ calcd for $C_{17}H_{20}O$, 240.1514; found, 240.1515.

Naphthalen-2-yl(*p*-tolyl)methanol (3bf).

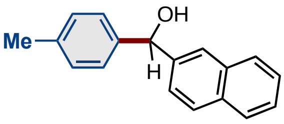

3bf

Conditions A: The reaction was conducted with **1b** (128.3 mg, 0.75 mmol) and **2f** (78.1 mg, 0.50 mmol). The product **3bf** was obtained in 72% yield (89.8 mg, 0.36 mmol) as a white powder.

Conditions B: The reaction was conducted with **1b** (173.4 mg, 1.0 mmol) and **2f** (78.2 mg, 0.50 mmol). The product **3bf** was obtained in 68% yield (84.0 mg, 0.34 mmol) as a white powder. ^{1}H and ^{13}C NMR of the product **3bf** were in agreement with the literature.[28]

^{1}H NMR (400 MHz, $CDCl_3$, δ): 2.30 (s, 3H), 2.43 (s, 1H), 5.90 (s, 1H), 7.11 (d, *J* = 8.0 Hz, 2H), 7.26 (d, *J* = 8.0 Hz, 2H), 7.38 (d, *J* = 8.4 Hz, 1H), 7.40–7.50 (m, 2H), 7.74 (d, *J* = 8.8 Hz, 1H), 7.76–7.82 (m, 2H), 7.85 (s, 1H). ^{13}C NMR (101 MHz, $CDCl_3$, δ): 21.1 (CH_3), 76.1 (*C*H), 124.7 (*C*H), 124.8 (*C*H), 125.8 (*C*H), 126.1 (*C*H), 126.6 (*C*H), 127.6 (*C*H), 128.0 (*C*H), 128.2 (*C*H), 129.2 (*C*H), 132.8 (*C*), 133.2 (*C*), 137.3 (*C*), 140.7 (*C*), 141.2 (*C*). HRMS-EI (m/z): $[M]^+$ calcd for $C_{18}H_{16}O$, 248.1201; found, 248.1198.

(4-Fluorophenyl)(phenyl)methanol (3ca).

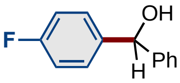

3ca

Conditions A: The reaction was conducted with **1c** (82 μL, 0.75 mmol) and **2a** (53.5 mg, 0.50 mmol). The product **3ca** was obtained in 77% yield (73.8 mg, 0.36 mmol) as a white powder.

Conditions B: The reaction was conducted with **1c** (109 μL, 1.0 mmol), lithium chloride (42.4 mg, 1.0 mmol), and **2a** (53.1 mg, 0.50 mmol). The product **3ca** was obtained in 67% yield (68.0 mg, 0.34 mmol) as a white powder. ^{1}H and ^{13}C NMR of the product **3ca** were in agreement with the literature.[23]

^{1}H NMR (401 MHz, $CDCl_3$, δ): 2.19 (d, *J* = 3.2 Hz, 1H), 5.84 (d, *J* = 3.6 Hz, 1H), 7.02 (tt, *J* = 2.2, 8.9 Hz,

2H), 7.27–7.39 (m, 7H). ^{13}C NMR (101 MHz, $CDCl_3$, δ): 75.5 (*C*H), 115.2 (d, $J_{C\text{-}F}$ = 22.1 Hz, *C*H), 126.4 (*C*H), 127.7 (*C*H), 128.2 (d, $J_{C\text{-}F}$ = 8.0 Hz, *C*H), 128.5 (*C*H), 139.5 (d, $J_{C\text{-}F}$ = 3.0 Hz, *C*), 143.6 (*C*), 162.1 (d, $J_{C\text{-}F}$ = 245.7 Hz, *C*). HRMS-ESI (m/z): $[M–H]^+$ calcd for $C_{13}H_{10}FO$, 201.0721; found, 201.0717.

1-(4-Fluorophenyl)-3-phenylpropan-1-ol (3cb).

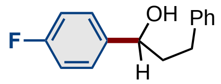

3cb

Conditions A: The reaction was conducted with **1c** (82 µL, 0.75 mmol) and **2b** (67.0 mg, 0.50 mmol). The product **3cb** was obtained in 70% yield (80.2 mg, 0.35 mmol) as a colorless oil.

Conditions B: The reaction was conducted with **1c** (109 µL, 1.0 mmol), lithium chloride (42.4 mg, 1.0 mmol) and **2b** (66.9 mg, 0.50 mmol). The product **3cb** was obtained in 61% yield (70.3 mg, 0.31 mmol) as a colorless oil. ^{1}H and ^{13}C NMR of the product **3cb** were in agreement with the literature.[26]

^{1}H NMR (392 MHz, $CDCl_3$, δ): 1.82 (d, *J* = 3.1 Hz, 1H), 1.95–2.18 (m, 2H), 2.61–2.79 (m, 2H), 4.68 (sept, *J* = 2.7 Hz, 1H), 7.04 (tt, *J* = 2.5, 9.1 Hz, 2H), 7.19 (t, *J* = 6.3 Hz, 3H), 7.26–7.36 (m, 4H). ^{13}C NMR (101 MHz, $CDCl_3$, δ): 31.9 (*C*H_2), 40.5 (*C*H_2), 73.2 (*C*H), 115.3 (d, $J_{C\text{-}F}$ = 22.1 Hz, *C*H), 125.9 (*C*H), 127.5 (d, $J_{C\text{-}F}$ = 7.7 Hz, *C*H), 128.37 (*C*H), 128.40 (*C*H), 140.2 (*C*), 141.5 (*C*), 162.2 (d, $J_{C\text{-}F}$ = 245.7 Hz, *C*). HRMS-ESI (m/z): $[M–H]^+$ calcd for $C_{15}H_{14}FO$, 229.1034; found, 229.1032.

Cyclohexyl(4-fluorophenyl)methanol (3cc).

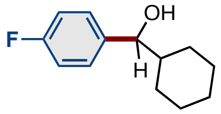

3cc

Conditions A: The reaction was conducted with **1c** (82 µL, 0.75 mmol) and **2c** (55.9 mg, 0.50 mmol). The product **3cc** was obtained in 62% yield (64.8 mg, 0.31 mmol) as a yellow oil.

Conditions B: The reaction was conducted with **1c** (109 µL, 1.0 mmol), lithium chloride (42.4 mg, 1.0 mmol) and **2c** (55.4 mg, 0.50 mmol). The product **3cc** was obtained in 55% yield (56.9 mg, 0.27 mmol) as a yellow oil. ^{1}H and ^{13}C NMR of the product **3cc** were in agreement with the literature.[29]

^{1}H NMR (400 MHz, $CDCl_3$, δ): 0.84–1.29 (m, 5H), 1.32–1.39 (m, 1H), 1.52–1.71 (m, 3H), 1.74–1.82 (m, 2H), 1.93–2.00 (m, 1H), 4.36 (dd, *J* = 3.0, 7.0 Hz, 1H), 7.02 (tt, *J* = 2.3, 9.0 Hz, 2H), 7.24–7.29 (m, 2H). ^{13}C NMR (101 MHz, $CDCl_3$, δ): 25.9 (*C*H_2), 26.0 (*C*H_2), 26.3 (*C*H_2), 28.8 (*C*H_2), 29.1 (*C*H_2), 45.0 (*C*H), 78.6 (*C*H), 114.9 (d, $J_{C\text{-}F}$ = 21.2 Hz, *C*H), 128.1 (d, $J_{C\text{-}F}$ = 7.7 Hz, *C*H), 139.2 (d, $J_{C\text{-}F}$ = 2.8 Hz, *C*), 162.0 (d, $J_{C\text{-}F}$ = 244.7 Hz, *C*). HRMS-EI (m/z): $[M]^+$ calcd for $C_{13}H_{17}FO$, 208.1263; found, 208.1263.

1-(4-Fluorophenyl)-1-phenylpropan-1-ol (3cd).

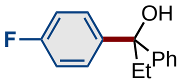

3cd

Conditions A: The reaction was conducted with **1c** (82 µL, 0.75 mmol) and **2d** (67.5 mg, 0.50 mmol). The product **3cd** was obtained in 71% yield (82.0 mg, 0.36 mmol) as a colorless oil.

Conditions B: The reaction was conducted with **1c** (175.0 mg, 1.0 mmol), lithium chloride (42.4 mg, 1.0 mmol) and **2d** (67.2 mg, 0.50 mmol). The product **3cd** was obtained in 61% yield (70.1 mg, 0.30 mmol) as a colorless oil. ^{1}H and ^{13}C NMR of the product **3cd** were in agreement with the literature.[24]

^{1}H NMR (400 MHz, $CDCl_3$, δ): 0.88 (t, J = 7.4 Hz, 3H), 2.04 (s, 1H), 2.30 (q, J = 7.2 Hz, 2H), 6.98 (tt, J = 2.3, 9.0 Hz, 2H), 7.23 (tt, J = 1.5, 7.1 Hz, 1H), 7.29–7.42 (m, 6H). ^{13}C NMR (101 MHz, $CDCl_3$, δ): 8.1 (CH_3), 34.5 (CH_2), 78.1 (C), 114.7 (d, $J_{C\text{-}F}$ = 21.2 Hz, CH), 126.0 (CH), 126.9 (CH), 127.8 (d, $J_{C\text{-}F}$ = 7.7 Hz, CH), 128.2 (CH), 142.7 (d, $J_{C\text{-}F}$ = 2.9 Hz, C), 146.7 (C), 161.5 (d, $J_{C\text{-}F}$ = 245.6 Hz, C). HRMS-ESI (m/z): $[M{-}H]^+$ calcd for $C_{15}H_{14}FO$, 229.1034; found, 229.1031.

2-(4-Fluorophenyl)-4-phenylbutan-2-ol (3ce).

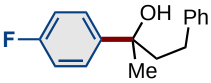

3ce

Conditions A: The reaction was conducted with **1c** (82 µL, 0.75 mmol) and **2e** (74.4 mg, 0.50 mmol). The product **3ce** was obtained in 54% NMR yield.

Conditions B: The reaction was conducted with **1c** (109 µL, 1.0 mmol), lithium chloride (42.4 mg, 1.0 mmol), and **2e** (73.9 mg, 0.50 mmol). The product **3ce** was obtained in 72% yield (87.9 mg, 0.36 mmol) as a white powder.

^{1}H NMR (392 MHz, $CDCl_3$, δ): 1.60 (s, 3H), 1.71 (s, 1H), 2.06–2.16 (m, 2H), 2.37–2.49 (m, 1H), 2.55–2.66 (m, 1H), 7.04 (tt, J = 2.4, 9.2 Hz, 2H), 7.11 (d, J = 6.7 Hz, 2H), 7.16 (tt, J = 1.6, 7.4 Hz, 1H), 7.22–7.28 (m, 2H), 7.40–7.48 (m, 2H). ^{13}C NMR (101 MHz, $CDCl_3$, δ): 30.4 (CH_2), 30.5 (CH_3), 46.0 (CH_2), 74.4 (C), 114.9 (d, $J_{C\text{-}F}$ = 21.2 Hz, CH), 125.8 (CH), 126.4 (CH), 126.5 (CH), 128.3 (d, $J_{C\text{-}F}$ = 13.5 Hz, CH), 142.0 (C), 143.2 (d, $J_{C\text{-}F}$ = 2.9 Hz, C), 161.6 (d, $J_{C\text{-}F}$ = 245.6 Hz, C). HRMS-EI (m/z): $[M]^+$ calcd for $C_{16}H_{17}FO$, 224.1263; found, 224.1267.

(4-Fluorophenyl)(naphthalen-2-yl)methanol (3cf).

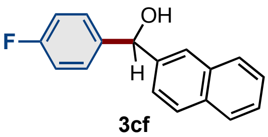

3cf

Conditions A: The reaction was conducted with **1c** (82 µL, 0.75 mmol) and **2f** (78.1 mg, 0.50 mmol). The product **3cf** was obtained in 48% NMR yield.

Conditions B: The reaction was conducted with **1c** (174.4 mg, 109 µL, 1.0 mmol) and **2f** (78.1 mg, 0.50 mmol). The product **3cf** was obtained in 65% yield (82.5 mg, 0.33 mmol) as pale-yellow oil. ^{1}H and ^{13}C

NMR of the product **3cf** were in agreement with the literature.[28]

^{1}H NMR (400 MHz, $CDCl_3$, δ): 2.55–2.64 (m, 1H), 5.88 (s, 1H), 6.97 (tt, J = 2.3, 9.1 Hz, 2H), 7.27–7.36 (m, 3H), 7.42–7.50 (m, 2H), 7.75 (d, J = 8.4 Hz, 1H), 7.77–7.84 (m, 3H). ^{13}C NMR (101 MHz, $CDCl_3$, δ): 75.6 (*C*H), 115.3 (d, $J_{C\text{-}F}$ = 21.2 Hz, *C*H), 124.5 (*C*H), 124.9 (*C*H), 126.1 (*C*H), 126.3 (*C*H), 127.6 (*C*H), 128.0 (*C*H), 128.3 (d, $J_{C\text{-}F}$ = 7.6 Hz, *C*H), 128.4 (*C*H), 132.8 (*C*), 133.1 (*C*), 139.3 (d, $J_{C\text{-}F}$ = 2.8 Hz, *C*), 140.9 (*C*), 162.1 (d, $J_{C\text{-}F}$ = 245.6 Hz, *C*). HRMS-EI (m/z): [M]$^+$ calcd for $C_{17}H_{13}FO$, 252.0950; found, 252.0946.

1-Phenylpentan-1-ol (3da).

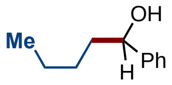

3da

Conditions A: The reaction was conducted with **1d** (81 µL, 0.75 mmol) and **2a** (53.4 mg, 0.50 mmol). The product **3da** was obtained in 73% yield (60.2 mg, 0.37 mmol) as a colorless oil.

Conditions B: The reaction was conducted with **1d** (107 µL, 1.0 mmol) and **2a** (53.3 mg, 0.50 mmol). The product **3da** was obtained in 77% yield (63.7 mg, 0.39 mmol) as a colorless oil. ^{1}H and ^{13}C NMR of the product **3da** were in agreement with the literature.[30]

^{1}H NMR (400 MHz, $CDCl_3$, δ): 0.89 (t, J = 7.0 Hz, 3H), 1.21–1.45 (m, 4H), 1.66–1.88 (m, 2H), 1.79 (d, J = 3.2 Hz, 1H), 4.64–4.70 (m, 1H), 7.27–7.31 (m, 1H), 7.35–7.36 (m, 4H). ^{13}C NMR (101 MHz, $CDCl_3$, δ): 14.0 (*C*H_3), 22.5 (*C*H_2), 27.9 (*C*H_2), 38.7 (*C*H_2), 74.6 (*C*H), 125.8 (*C*H), 127.4 (*C*H), 128.3 (*C*H), 144.9 (*C*). HRMS-EI (m/z): [M]$^+$ calcd for $C_{11}H_{16}O$, 164.1201; found, 164.1200.

1-Phenylheptan-3-ol (3db).

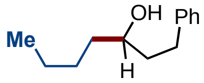

3db

Conditions A: The reaction was conducted with **1d** (81 µL, 0.75 mmol) and **2b** (67.0 mg, 0.50 mmol). The product **3db** was obtained in 46% yield (44.6 mg, 0.23 mmol) as pale-yellow oil.

Conditions B: The reaction was conducted with **1d** (136.0 mg, 1.0 mmol) and **2b** (67.2 mg, 0.50 mmol). The product **3db** was obtained in 62% yield (60.1 mg, 0.31 mmol) as pale-yellow oil. ^{1}H and ^{13}C NMR of the product **3db** were in agreement with the literature.[26]

^{1}H NMR (400 MHz, $CDCl_3$, δ): 0.90 (t, J = 6.8 Hz, 3H), 1.21–1.61 (m, 7H), 1.66–1.87 (m, 2H), 2.59–2.72 (m, 1H), 2.74–2.86 (m, 1H), 3.61 (sept, J = 4.0 Hz, 1H), 7.14–7.23 (m, 3H), 7.28 (t, J = 7.6 Hz, 2H). ^{13}C NMR (101 MHz, $CDCl_3$, δ): 14.0 (*C*H_3), 22.7 (*C*H_2), 27.8 (*C*H_2), 32.0 (*C*H_2), 37.2 (*C*H_2), 39.0 (*C*H_2), 71.3 (*C*H), 125.7 (*C*H), 128.3 (*C*H), 128.4 (*C*H), 142.2 (*C*). HRMS-EI (m/z): [M]$^+$ calcd for $C_{13}H_{20}O$, 192.1514; found, 192.1511.

1-Cyclohexylpentan-1-ol (3dc).

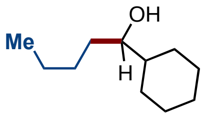

3dc

Conditions A: The reaction was conducted with **1d** (81 μL, 0.75 mmol) and **2c** (55.8 mg, 0.50 mmol). The product **3dc** was obtained in 67% yield (57.0 mg, 0.33 mmol) as a pale-yellow oil.

Conditions B: The reaction was conducted with **1d** (137.2 mg, 1.0 mmol) and **2c** (56.6 mg, 0.50 mmol). The product **3dc** was obtained in 55% yield (47.2 mg, 0.28 mmol) as a pale-yellow oil. ^{1}H and ^{13}C NMR of the product **3dc** were in agreement with the literature.[31]

^{1}H NMR (400 MHz, $CDCl_3$, δ): 0.91 (t, *J* = 6.8 Hz, 3H), 0.97–1.57 (m, 13H), 1.60–1.70 (m, 2H), 1.72–1.88 (m, 3H), 3.29–3.45 (m, 1H). ^{13}C NMR (101 MHz, $CDCl_3$, δ): 14.1 (CH_3), 22.8 (CH_2), 26.2 (CH_2), 26.4 (CH_2), 26.5 (CH_2), 27.6 (CH_2), 28.1 (CH_2), 29.2 (CH_2), 33.8 (CH_2), 43.5 (CH), 76.2 (CH). HRMS-ESI (m/z): [M–H]$^+$ calcd for $C_{11}H_{21}O$, 169.1598; found, 169.1594.

3-Phenylheptan-3-ol (3dd).

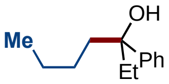

3dd

Conditions A: The reaction was conducted with **1d** (80 μL, 0.75 mmol) and **2d** (67.4 mg, 0.50 mmol). The product **3dd** was obtained in 58% NMR yield.

Conditions B: The reaction was conducted with **1d** (107 μL, 1.0 mmol) and **2d** (73.4 mg, 0.55 mmol). The product **3dd** was obtained in 60% yield (62.6 mg, 0.32 mmol) as a pale-yellow oil. ^{1}H and ^{13}C NMR of the product **3dd** were in agreement with the literature.[32]

^{1}H NMR (400 MHz, $CDCl_3$, δ): 0.75 (t, *J* = 7.4 Hz, 3H), 0.83 (t, *J* = 7.4 Hz, 3H), 0.94–1.11 (m, 1H), 1.16–1.34 (m, 3H), 1.69 (s, 1H), 1.72–1.92 (m, 4H), 7.22 (tt, *J* = 1.8, 7.0 Hz, 1H), 7.30–7.40 (m, 4H). ^{13}C NMR (101 MHz, $CDCl_3$, δ): 7.8 (CH_3), 14.0 (CH_3), 23.1 (CH_2), 25.6 (CH_2), 35.4 (CH_2), 42.3 (CH_2), 77.2 (C), 125.3 (CH), 126.2 (CH), 127.9 (CH), 146.1 (C). HRMS-ESI (m/z): [M+Na]$^+$ calcd for $C_{13}H_{20}ONa$, 215.1412; found, 215.1409.

3-Methyl-1-phenylheptan-3-ol (3de).

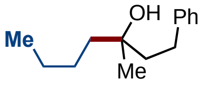

3de

Conditions A: The reaction was conducted with **1d** (80 μL, 0.75 mmol) and **2e** (74.0 mg, 0.50 mmol). The product **3de** was obtained in 66% NMR yield.

Conditions B: The reaction was conducted with **1d** (137.0 mg, 1.0 mmol) and **2e** (74.8 mg, 0.50 mmol). The product was **3de** obtained in 63% yield (65.6 mg, 0.32 mmol) as a yellow oil. ^{1}H and ^{13}C NMR of the product **3de** were in agreement with the literature.[33]

^{1}H NMR (400 MHz, $CDCl_3$, δ): 0.92 (t, J = 7.0 Hz, 3H), 1.23 (s, 3H), 1.28–1.41 (m, 5H), 1.45–1.61 (m, 2H), 1.69–1.82 (m, 2H), 2.61–2.72 (m, 2H), 7.12–7.22 (m, 3H), 7.28 (t, J = 7.4 Hz, 2H). ^{13}C NMR (101 MHz, $CDCl_3$, δ): 14.1 (CH_3), 23.2 (CH_2), 26.1 (CH_2), 26.9 (CH_3), 30.3 (CH_2), 41.7 (CH_2), 43.7 (CH_2), 72.6 (C), 125.7 (CH), 128.27 (CH), 128.34 (CH), 142.6 (C). HRMS-ESI (m/z): $[M–H]^+$ calcd for $C_{14}H_{21}O$, 205.1598; found, 205.1595.

1-(Naphthalen-2-yl)pentan-1-ol (3df).

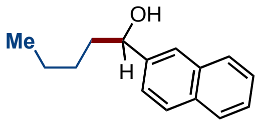

3df

Conditions A: The reaction was conducted with **1d** (80 µL, 0.75 mmol) and **2f** (78.1 mg, 0.50 mmol). The product **3df** was obtained in 68% yield (73.1 mg, 0.34 mmol) as a white solid.

Conditions B: The reaction was conducted with **1d** (137.0 mg, 1.0 mmol) and **2f** (78.2 mg, 0.50 mmol). The product **3df** was obtained in 99% yield (107.3 mg, 0.50 mmol) as a white solid. ^{1}H and ^{13}C NMR of the product **3df** were in agreement with the literature.[30]

^{1}H NMR (400 MHz, $CDCl_3$, δ): 0.86 (t, J = 7.0 Hz, 3H), 1.17–1.45 (m, 4H), 1.69–1.90 (m, 2H), 2.21 (s, 1H), 4.76 (t, J = 6.6 Hz, 1H), 7.39–7.50 (m, 3H), 7.72 (s, 1H), 7.76–7.84 (m, 3H). ^{13}C NMR (101 MHz, $CDCl_3$, δ): 14.0 (CH_3), 22.6 (CH_2), 27.9 (CH_2), 38.6 (CH_2), 74.6 (CH), 124.1 (CH), 124.5 (CH), 125.7 (CH), 126.0 (CH), 127.6 (CH), 127.8 (CH), 128.1 (CH), 132.8 (C), 133.2 (C), 142.2 (C). HRMS-EI (m/z): $[M]^+$ calcd for $C_{15}H_{18}O$, 214.1358; found, 214.1355.

Cyclohexyl(phenyl)methanol (3ea).

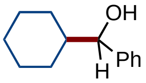

3ea

Conditions A: The reaction was conducted with **1e** (92 µL, 0.75 mmol) and **2a** (53.5 mg, 0.50 mmol). The product **3ea** was obtained in 24% NMR yield.

Conditions B: The reaction was conducted with **1e** (123 µL, 1.0 mmol) and **2a** (52.9 mg, 0.50 mmol). The reaction time of the first step was extended to 90 minutes. The product **3ea** was obtained in 58% yield (55.2 mg, 0.29 mmol) as a colorless oil. ^{1}H and ^{13}C NMR of the product **3ea** were in agreement with the literature.[23]

^{1}H NMR (392 MHz, $CDCl_3$, δ): 0.87–1.29 (m, 5H), 1.34–1.42 (m, 1H), 1.57–1.71 (m, 3H), 1.73–1.82 (m, 1H), 1.80 (d, J = 3.6 Hz, 1H), 1.95–2.03 (m, 1H), 4.37 (dd, J = 2.8, 7.6 Hz, 1H), 7.24–7.37 (m, 5H). ^{13}C NMR (101 MHz, $CDCl_3$, δ): 25.95 (CH_2), 26.04 (CH_2), 26.4 (CH_2), 28.8 (CH_2), 29.2 (CH_2), 44.9 (CH), 79.3 (CH), 126.6 (CH), 127.3 (CH), 128.1 (CH), 143.6 (C). HRMS-EI (m/z): $[M]^+$ calcd for $C_{13}H_{18}O$, 190.1358; found, 190.1351.

1-Cyclohexyl-3-phenylpropan-1-ol (3eb).

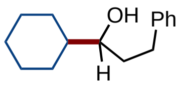

3eb

Conditions A: The reaction was conducted with **1e** (92 μL, 0.75 mmol) and **2b** (67.1 mg, 0.50 mmol). The product **3eb** was obtained in 40% yield (43.8 mg, 0.20 mmol) as a white solid.

Conditions B: The reaction was conducted with **1e** (162.8 mg, 1.0 mmol) and **2b** (67.2 mg, 0.50 mmol). The reaction time of the first step was extended to 90 minutes. The product **3eb** was obtained in 42% yield (45.9 mg, 0.21 mmol) as a white solid. ^{1}H and ^{13}C NMR of the product **3eb** were in agreement with the literature.[26]

^{1}H NMR (400 MHz, $CDCl_3$, δ): 0.94–1.39 (m, 6H), 1.42 (s, 1H), 1.60–1.87 (m, 7H), 2.58–2.72 (m, 1H), 2.78–2.89 (m, 1H), 3.34–3.43 (m, 1H), 7.14–7.23 (m, 3H), 7.24–7.32 (m, 2H). ^{13}C NMR (101 MHz, $CDCl_3$, δ): 26.1 (CH_2), 26.3 (CH_2), 26.5 (CH_2), 27.8 (CH_2), 29.1 (CH_2), 32.3 (CH_2), 35.9 (CH_2), 43.7 (CH), 75.6 (CH), 125.7 (CH), 128.3 (CH), 128.4 (CH), 142.4 (C). HRMS-ESI (m/z): $[M+Na]^+$ calcd for $C_{15}H_{22}ONa$, 241.1563; found, 241.1562.

Dicyclohexylmethanol (3ec).

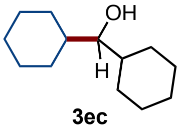

3ec

Conditions A: The reaction was conducted with **1e** (92 μL, 0.75 mmol) and **2c** (56.5 mg, 0.50 mmol). The product **3ec** was obtained in 26% NMR yield.

Conditions B: The reaction was conducted with **1e** (123 μL, 1.0 mmol) and **2c** (56.2 mg, 0.50 mmol). The reaction time of the first step was extended to 90 minutes. The product **3ec** was obtained in 43% yield (42.0 mg, 0.21 mmol) as a white solid. ^{1}H and ^{13}C NMR of the product **3ec** were in agreement with the literature.[34]

^{1}H NMR (400 MHz, $CDCl_3$, δ): 0.94–1.32 (m, 11H), 1.37–1.49 (m, 2H), 1.52–1.60 (m, 2H), 1.62–1.70 (m, 2H), 1.71–1.87 (m, 6H), 2.99–3.10 (m, 1H). ^{13}C NMR (101 MHz, $CDCl_3$, δ): 26.1 (CH_2), 26.46 (CH_2), 26.52 (CH_2), 27.3 (CH_2), 30.0 (CH_2), 39.8 (CH), 80.4 (CH). HRMS-ESI (m/z): $[M+Na]^+$ calcd for $C_{13}H_{24}ONa$, 219.1725; found, 219.1729.

1-Cyclohexyl-1-phenylpropan-1-ol (3ed).

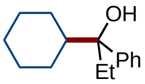

3ed

Conditions A: The reaction was conducted with **1e** (97 μL, 0.79 mmol) and **2d** (67.5 mg, 0.50 mmol). The product **3ed** was obtained in 33% NMR yield.

Conditions B: The reaction was conducted with **1e** (163.7 mg, 1.0 mmol) and **2d** (67.4 mg, 0.50 mmol). The reaction time of the first step was extended to 90 minutes. The product **3ed** was obtained in 30% yield (33.3 mg, 0.15 mmol) as a colorless oil. ^{1}H and ^{13}C NMR of the product **3ed** were in agreement with the literature.[35]

^{1}H NMR (400 MHz, $CDCl_3$, δ): 0.69 (t, *J* = 7.4 Hz, 3H), 0.87–1.29 (m, 5H), 1.38–1.46 (m, 1H), 1.56–1.70 (m, 4H), 1.73–1.81 (m, 1H), 1.89 (q, *J* = 7.3 Hz, 2H), 1.92–1.95 (m, 1H), 7.19–7.24 (m, 1H), 7.29–7.38 (m, 4H). ^{13}C NMR (101 MHz, $CDCl_3$, δ): 7.8 (CH_3), 26.4 (CH_2), 26.6 (CH_2), 26.67 (CH_2), 26.69 (CH_2), 27.4 (CH_2), 31.6 (CH_2), 47.9 (*C*H), 79.2 (*C*), 125.9 (*C*H), 126.0 (*C*H), 127.7 (*C*H), 145.1 (*C*). HRMS-ESI (m/z): $[M-H]^+$ calcd for $C_{15}H_{21}O$, 217.1598; found, 217.1598.

2-Cyclohexyl-4-phenylbutan-2-ol (3ee).

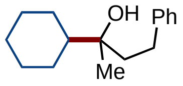

3ee

Conditions A: The reaction was conducted with **1e** (97 µL, 0.79 mmol) and **2e** (75.1 mg, 0.51 mmol). The product **3ee** was obtained in 15% yield (17.1 mg, 0.073 mmol) as a pale-yellow oil.

Conditions B: The reaction was conducted with **1e** (163.7 mg, 1.0 mmol) and **2e** (73.2 mg, 0.49 mmol). The reaction time of the first step was extended to 90 minutes. The product **3ee** was obtained in 22% yield (25.7 mg, 0.11 mmol) as a pale-yellow oil. ^{1}H and ^{13}C NMR of the product **3ee** were in agreement with the literature.[36]

^{1}H NMR (401 MHz, $CDCl_3$, δ): 0.95–1.31 (m, 9H), 1.39 (tt, *J* = 2.4, 11.8 Hz, 1H), 1.61–1.90 (m, 7H), 2.68 (q, *J* = 7.7 Hz, 2H), 7.14–7.23 (m, 3H), 7.24–7.32 (m, 2H). ^{13}C NMR (101 MHz, $CDCl_3$, δ): 23.8 (CH_3), 26.5 (CH_2), 26.7 (CH_2), 26.8 (CH_2), 26.9 (CH_2), 27.6 (CH_2), 29.8 (CH_2), 41.9 (CH_2). 47.6 (*C*H), 74.4 (*C*), 125.7 (*C*H), 128.3 (*C*H), 128.4 (*C*H), 142.9 (*C*). HRMS-ESI (m/z): $[M+Na]^+$ calcd for $C_{16}H_{24}ONa$, 255.1719; found, 255.1718.

Cyclohexyl(naphthalen-2-yl)methanol (3ef).

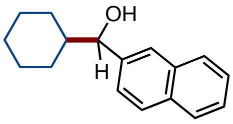

3ef

Conditions A: The reaction was conducted with **1e** (97 µL, 0.79 mmol) and **2f** (78.1 mg, 0.50 mmol). The product **3ef** was obtained in 19% NMR yield.

Conditions B: The reaction was conducted with **1e** (130 µL, 1.1 mmol), Mg (48.6 mg, 2.0 mmol), and **2f** (78.1 mg, 0.50 mmol). The product **3ef** was obtained in 50% yield (59.9 mg, 0.25 mmol) as a white solid. ^{1}H and ^{13}C NMR of the product **3ef** were in agreement with the literature.[30]

^{1}H NMR (400 MHz, $CDCl_3$, δ): 0.98 (dquint, *J* = 3.4, 10.1 Hz, 1H), 1.05–1.31 (m, 4H), 1.34–1.45 (m, 1H), 1.53–1.82 (m, 4H), 1.98 (s, 1H), 2.02 (d, *J* = 12.8 Hz, 1H), 4.53 (d, *J* = 7.2 Hz, 1H), 7.40–7.51 (m, 3H), 7.72 (s, 1H), 7.78–7.87 (m, 3H). ^{13}C NMR (101 MHz, $CDCl_3$, δ): 26.0 (CH_2), 26.1 (CH_2), 26.4 (CH_2), 28.8 (CH_2), 29.4 (CH_2), 44.9 (*C*H), 79.5 (*C*H), 124.7 (*C*H), 125.5 (*C*H), 125.7 (*C*H), 126.0 (*C*H), 127.6 (*C*H), 127.88 (*C*H), 127.92 (*C*H), 132.9 (*C*), 133.1 (*C*), 141.0 (*C*). HRMS-EI (m/z): $[M]^+$ calcd for $C_{17}H_{20}O$, 240.1514; found, 240.1510.

(1,1'-Biphenyl)-4-yl(phenyl)methanol (3fa).

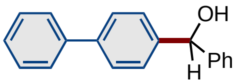

3fa

Conditions C: The reaction was conducted with **1f** (233.9 mg, 1.0 mmol), THF (1.5 mmol for the first step, 2.5 mmol for the second step) and **2a** (54.1 mg, 0.51 mmol). The product **3fa** was obtained in 75% yield (99.3 mg, 0.38 mmol) as a white solid.

Conditions D: The reaction was conducted with **1f** (174.9 mg, 0.75 mmol) and **2a** (53.3 mg, 0.50 mmol). The product **3fa** was obtained in 76% yield (99.8 mg, 0.38 mmol) as a white solid. ^{1}H and ^{13}C NMR of the product **3fa** were in agreement with the literature.[23]

^{1}H NMR (400 MHz, $CDCl_3$, δ): 2.47 (s, 1H), 5.81 (s, 1H), 7.25 (tt, *J* = 1.8, 7.2 Hz, 1H), 7.29–7.35 (m, 3H), 7.36–7.43 (m, 6H), 7.50–7.57 (m, 4H). ^{13}C NMR (101 MHz, $CDCl_3$, δ): 75.9 (*C*H), 126.5 (*C*H), 126.9 (*C*H), 127.0 (*C*H), 127.17 (*C*H), 127.23 (*C*H), 127.6 (*C*H), 128.5 (*C*H), 128.7 (*C*H), 140.4 (*C*), 140.7 (*C*), 142.7 (*C*), 143.7 (*C*). HRMS-EI (m/z): [M]$^+$ calcd for $C_{19}H_{16}O$, 260.1201; found, 260.1205.

(1,1'-Biphenyl)-4-yl(naphthalen-2-yl)methanol (3ff).

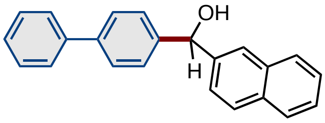

3ff

Conditions C: The reaction was conducted with **1f** (233.7 mg, 1.0 mmol), THF (1.5 mmol for the first step, 2.5 mmol for the second step) and **2f** (78.3 mg, 0.50 mmol). The product **3ff** was obtained in 76% yield (118.4 mg, 0.38 mmol) as a white solid.

Conditions D: The reaction was conducted with **1f** (174.4 mg, 0.75 mmol) and **2f** (78.0 mg, 0.50 mmol). The product **3ff** was obtained in 60% yield (92.4 mg, 0.30 mmol) as a white solid. ^{1}H and ^{13}C NMR of the product **3ff** were in agreement with the literature.[37]

^{1}H NMR (400 MHz, $CDCl_3$, δ): 2.43 (d, *J* = 3.6 Hz, 1H), 6.01 (d, *J* = 2.4 Hz, 1H), 7.32 (tt, *J* = 1.5, 7.4 Hz, 1H), 7.38–7.50 (m, 7H), 7.52–7.58 (m, 4H), 7.77–7.87 (m, 3H), 7.91 (s, 1H). ^{13}C NMR (101 MHz, $CDCl_3$, δ): 76.1 (*C*H), 124.7 (*C*H), 125.0 (*C*H), 126.0 (*C*H), 126.2 (*C*H), 127.06 (*C*H), 127.11 (*C*H), 127.3 (*C*H), 127.7 (*C*H), 128.1 (*C*H), 128.4 (*C*H), 128.7 (*C*H), 132.9 (*C*), 133.2 (*C*), 140.5 (*C*), 140.7 (*C*), 141.0 (*C*), 142.6 (*C*). HRMS-EI (m/z): [M]$^+$ calcd for $C_{23}H_{18}O$, 310.1358; found, 310.1350.

Naphthalen-2-yl(phenyl)methanol (3ga).

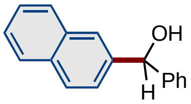

3ga

Conditions C: The reaction was conducted with **1g** (207.2 mg, 1.0 mmol), THF (1.5 mmol for the first step, 2.5 mmol for the second step) and **2a** (53.9 mg, 0.51 mmol). The product **3ga** was obtained in 77% yield (91.7 mg, 0.39 mmol) as a white solid. ^{1}H and ^{13}C NMR of the product **3ga** were in agreement with the

literature.[23]

Conditions D: The reaction was conducted with **1g** (156.0 mg, 0.75 mmol) and **2a** (52.6 mg, 0.50 mmol). The product **3ga** was obtained in 61% NMR yield.

^{1}H NMR (400 MHz, $CDCl_3$, δ): 2.32 (d, *J* = 3.6 Hz, 1H), 6.01 (d, *J* = 3.6 Hz, 1H), 7.25−7.30 (m, 1H), 7.34 (tt, *J* = 1.7, 7.3 Hz, 2H), 7.40–7.45 (m, 3H), 7.47 (quint, *J* = 3.1 Hz, 2H), 7.77–7.86 (m, 3H), 7.90 (s, 1H). ^{13}C NMR (101 MHz, $CDCl_3$, δ): 76.3 (*C*H), 124.7 (*C*H), 125.0 (*C*H), 125.9 (*C*H), 126.2 (*C*H), 126.7 (*C*H), 127.6 (*C*H), 128.0 (*C*H), 128.3 (*C*H), 128.5 (*C*H), 132.8 (*C*), 133.2 (*C*), 141.0 (*C*), 143.6 (*C*). HRMS-EI (m/z): $[M]^+$ calcd for $C_{17}H_{14}O$, 234.1045; found, 234.1046.

(3,5-Di-*tert*-butylphenyl)(phenyl)methanol (3ha).

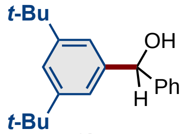

3ha

Conditions C: The reaction was conducted with **1h** (269.8 mg, 1.0 mmol), THF (1.5 mmol for the first step, 2.5 mmol for the second step) and **2a** (53.8 mg, 0.51 mmol). The product **3ha** was obtained in 75% yield (112.4 mg, 0.38 mmol) as a white solid.

Conditions D: The reaction was conducted with **1h** (201.9 mg, 0.75 mmol) and **2a** (52.3 mg, 0.49 mmol). The product **3ha** was obtained in 35% NMR yield.

^{1}H NMR (400 MHz, $CDCl_3$, δ): 1.30 (s, 18H), 2.19 (d, *J* = 3.2 Hz, 1H), 5.84 (d, *J* = 3.6 Hz, 1H), 7.23 (d, *J* = 1.2 Hz, 2H), 7.24–7.29 (m, 1H), 7.31–7.37 (m, 3H), 7.38–7.43 (m, 2H). ^{13}C NMR (101 MHz, $CDCl_3$, δ): 31.4 (CH_3), 34.9 (*C*), 76.9 (*C*H), 120.8 (*C*H), 121.6 (*C*H), 126.5 (*C*H), 127.3 (*C*H), 128.3 (*C*H), 142.9 (*C*), 143.9 (*C*), 150.8 (*C*). HRMS-EI (m/z): $[M]^+$ calcd for $C_{21}H_{28}O$, 296.2140; found, 296.2135.

(1,1':3',1''-Terphenyl)-5'-yl(phenyl)methanol (3ia).

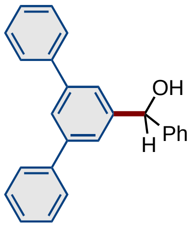

3ia

Conditions C: The reaction was conducted with **1i** (309.2 mg, 1.0 mmol), THF (1.5 mmol for the first step, 2.5 mmol for the second step) and **2a** (53.8 mg, 0.51 mmol). The product **3ia** was obtained in 82% yield (139.4 mg, 0.41 mmol) as a white solid.

Conditions D: The reaction was conducted with **1i** (231.6 mg, 0.75 mmol) and **2a** (53.6 mg, 0.50 mmol). The product **3ia** was obtained in 61% NMR yield.

^{1}H NMR (400 MHz, $CDCl_3$, δ): 2.43 (s, 1H), 5.92 (s, 1H), 7.25 (tt, *J* = 1.7, 7.3 Hz, 1H), 7.30−7.36 (m, 4H), 7.39–7.45 (m, 6H), 7.56–7.60 (m, 4H), 7.61 (t, *J* = 1.8 Hz, 2H), 7.69 (t, *J* = 1.6 Hz, 1H). ^{13}C NMR (101 MHz, $CDCl_3$, δ): 76.3 (*C*H), 124.3 (*C*H), 125.4 (*C*H), 126.5 (*C*H), 127.3 (*C*H), 127.5 (*C*H), 127.7 (*C*H), 128.6 (*C*H), 128.7 (*C*H), 140.9 (*C*), 142.0 (*C*), 143.6 (*C*), 144.8 (*C*). HRMS-EI (m/z): $[M]^+$ calcd for $C_{25}H_{20}O$,

336.1514; found, 336.1503.

[4'-(*tert*-Butyl)-(1,1'-biphenyl)-4-yl](phenyl)methanol (3ja).

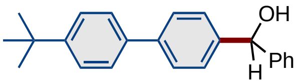

3ja

Conditions C: The reaction was conducted with **1j** (289.5 mg, 1.0 mmol), THF (1.5 mmol for the first step, 2.5 mmol for the second step) and **2a** (52.7 mg, 0.50 mmol). The product was obtained in 79% yield (124.8 mg, 0.39 mmol) as a white solid.

Conditions D: The reaction was conducted with **1j** (217.1 mg, 0.75 mmol) and **2a** (53.5 mg, 0.50 mmol). The product **3ja** was not detected by ^{1}H NMR.

^{1}H NMR (400 MHz, $CDCl_3$, δ): 1.35 (s, 9H), 2.29 (s, 1H), 5.87 (s, 1H), 7.27 (tt, *J* = 1.7, 7.2 Hz, 1H), 7.32–7.38 (m, 2H), 7.39–7.47 (m, 6H), 7.51 (dt, *J* = 2.0, 8.7 Hz, 2H), 7.55 (dt, *J* = 1.8, 8.1 Hz, 2H). ^{13}C NMR (101 MHz, $CDCl_3$, δ): 31.3 (CH_3), 34.5 (*C*), 76.0 (*C*H), 125.7 (*C*H), 126.5 (*C*H), 126.7 (*C*H), 126.9 (*C*H), 127.1 (*C*H), 127.6 (*C*H), 128.5 (*C*H), 137.8 (*C*), 140.3 (*C*), 142.5 (*C*), 143.7 (*C*), 150.3 (*C*). HRMS-EI (m/z): $[M]^+$ calcd for $C_{23}H_{24}O$, 316.1827; found, 316.1816.

(1,1':3',1''-Terphenyl)-5'-yl(phenyl)methanol (3kb).

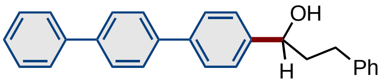

3kb

Conditions C: The reaction was conducted with **1k** (309.6 mg, 1.0 mmol), THF (1.5 mmol for the first step, 2.5 mmol for the second step) and **2b** (68.7 mg, 0.51 mmol). The product **3kb** was obtained in 42% yield (72.4 mg, 0.20 mmol) as a white solid.

^{1}H NMR (392 MHz, $CDCl_3$, δ): 1.87 (d, *J* = 3.5 Hz, 1H), 2.03–2.25 (m, 2H), 2.67–2.85 (m, 2H), 4.73–4.80 (m, 1H), 7.17–7.24 (m, 3H), 7.27–7.32 (m, 2H), 7.34–7.39 (m, 1H), 7.43–7.49 (m, 4H), 7.62–7.66 (m, 4H), 7.68 (s, 4H). ^{13}C NMR (99 MHz, DMSO-d_6, 70 °C, δ): 31.3 (CH_2), 40.5 (CH_2), 71.2 (*C*H), 125.2 (*C*H), 125.9 (*C*H), 126.19 (*C*H), 126.21 (*C*H), 126.7 (*C*H), 126.8 (*C*H), 127.1 (*C*H), 127.9 (*C*H), 128.6 (*C*H), 137.8 (*C*), 138.7 (*C*), 138.9 (*C*), 139.5 (*C*), 141.8 (*C*), 145.2 (*C*). HRMS-EI (m/z): $[M]^+$ calcd for $C_{27}H_{24}O$, 364.1827; found, 364.1825.

3-Phenyl-1-[5'-phenyl-(1,1':3',1''-terphenyl)-4-yl]propan-1-ol (3lb)

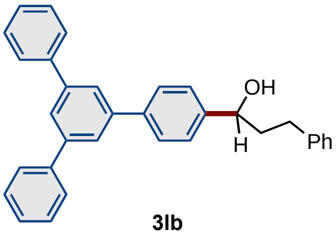

3lb

Conditions C: The reaction was conducted with **1l** (384.7 mg, 1.0 mmol), THF (1.5 mmol for the first step, 2.5 mmol for the second step) and **2b** (66.9 mg, 0.50 mmol). The product **3lb** was obtained in 39% yield

(85.9 mg, 0.19 mmol) as a white solid.

^{1}H NMR (392 MHz, $CDCl_3$, δ): 1.89 (d, J = 3.5 Hz, 1H), 2.04–2.28 (m, 2H), 2.67–2.87 (m, 2H), 4.74–4.81 (m, 1H), 7.18–7.33 (m, 5H), 7.37–7.43 (m, 2H), 7.45–7.52 (m, 6H), 7.68–7.73 (m, 6H), 7.79 (s, 3H). ^{13}C NMR (99 MHz, $CDCl_3$, δ): 32.0 (CH$_2$), 40.4 (CH$_2$), 73.5 (CH), 125.0 (CH), 125.1 (CH), 125.8 (CH), 126.4 (CH), 127.3 (CH), 127.4 (CH), 127.5 (CH), 128.36 (CH), 128.41 (CH), 128.8 (CH), 140.3 (C), 141.0 (C), 141.7 (C), 141.8 (C), 142.3 (C), 143.8 (C). HRMS-EI (m/z): [M+Na]$^+$ calcd for $C_{33}H_{28}ONa$, 463.2032; found, 463.2033.

1-Phenylpentan-1-one (5a).

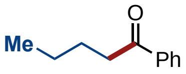

5a

Conditions E: The reaction was conducted with **1d** (102.3 mg, 0.75 mmol) and **4a** (86.8 mg, 0.53 mmol). The product **5a** was obtained in 60% NMR yield and 44% isolated yield (37.7 mg, 0.23 mmol) as a pale-yellow oil.

Conditions F: The reaction was conducted with **1d** (107 μL, 1.0 mmol) and **4a** (82.5 mg, 0.50 mmol). The product **5a** was obtained in 53% yield (43.3 mg, 0.27 mmol) as a pale-yellow oil. ^{1}H and ^{13}C NMR of the product **5a** were in agreement with the literature.[38]

^{1}H NMR (400 MHz, $CDCl_3$, δ): 0.96 (t, J = 7.4 Hz, 3H), 1.42 (sex, J = 7.4 Hz, 2H), 1.73 (quint, J = 7.4 Hz, 2H), 2.97 (t, J = 7.6 Hz, 2H), 7.46 (t, J = 7.4 Hz, 2H), 7.56 (tt, J = 1.7, 7.3 Hz, 1H), 7.95–7.99 (m, 2H). ^{13}C NMR (100 MHz, $CDCl_3$, δ): 13.9 (CH$_3$), 22.5 (CH$_2$), 26.4 (CH$_2$), 38.3 (CH$_2$), 128.0 (CH), 128.5 (CH), 132.8 (CH), 137.0 (C), 200.6 (C). HRMS-EI (m/z): [M]$^+$ calcd for $C_{11}H_{14}O$, 162.1045; found, 162.1044.

5-Phenylnonan-5-ol (5b).

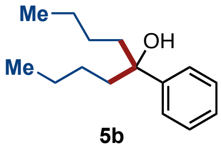

5b

Conditions G: The reaction was conducted with **1d** (136.1 mg, 1.0 mmol) and **4b** (45.5 mg, 0.33 mmol). The product **5b** was obtained in 61% yield (44.7 mg, 0.20 mmol) as a colorless oil. ^{1}H and ^{13}C NMR of the product **5b** were in agreement with the literature.[32]

^{1}H NMR (400 MHz, $CDCl_3$, δ): 0.83 (t, J = 7.2 Hz, 6H), 0.96–1.08 (m, 2H), 1.18–1.33 (m, 6H), 1.67 (s, 1H), 1.72–1.88 (m, 4H), 7.22 (t, J = 7.2 Hz, 1H), 7.31–7.40 (m, 4H). ^{13}C NMR (100 MHz, $CDCl_3$, δ): 14.0 (CH$_3$), 23.1 (CH$_2$), 25.6 (CH$_2$), 42.7 (CH$_2$), 125.2 (CH), 126.2 (CH), 128.0 (CH), 146.5 (C). HRMS-ESI (m/z): [M–H]$^+$ calcd for $C_{15}H_{23}O$, 219.1754; found, 219.1752.

1-(Naphthalen-2-yl)pentan-1-one (5c).

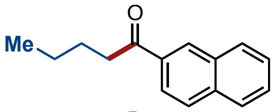

5c

Conditions E: The reaction was conducted with **1d** (102.4 mg, 0.75 mmol) and **4c** (76.6 mg, 0.50 mmol). The product **5c** was obtained in 40% NMR yield.

Conditions F: The reaction was conducted with **1d** (136.8 mg, 1.0 mmol) and **4c** (76.8 mg, 0.50 mmol). The product **5c** was obtained in 52% yield (55.5 mg, 0.26 mmol) as a yellow solid. ^{1}H and ^{13}C NMR of the product **5c** were in agreement with the literature.[38]

^{1}H NMR (400 MHz, $CDCl_3$, δ): 0.99 (t, *J* = 7.2 Hz, 3H), 1.46 (sex, *J* = 7.4 Hz, 2H), 1.79 (quint, *J* = 7.5 Hz, 2H), 3.11 (t, *J* = 7.2 Hz, 2H), 7.53–7.63 (m, 2H), 7.89 (t, *J* = 7.8 Hz, 2H), 7.98 (d, *J* = 8.0 Hz, 1H), 8.04 (dd, *J* = 1.8, 8.6 Hz, 1H), 8.48 (s, 1H). ^{13}C NMR (100 MHz, $CDCl_3$, δ): 14.0 (CH_3), 22.5 (CH_2), 26.6 (CH_2), 38.4 (CH_2), 123.9 (*C*H), 126.6 (*C*H), 127.7 (*C*H), 128.26 (*C*H), 128.32 (*C*H), 129.47 (*C*H), 129.54 (*C*H), 132.5 (*C*), 134.3 (*C*), 135.4 (*C*), 200.5 (*C*). HRMS-EI (m/z): $[M]^+$ calcd for $C_{15}H_{16}O$, 212.1201; found, 212.1201.

Dimethyldiphenylsilane (5d).

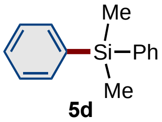

5d

Conditions E: The reaction was conducted with **1a** (118.4 mg, 0.75 mmol), **4d** (85 mg, 0.50 mmol), and CuI (23.6 mg, 0.13 mmol). The product **5d** was obtained in 62% yield (66.0 mg, 0.31 mmol) as a colorless oil.

Conditions F: The reaction was conducted with **1a** (157.0 mg, 1.0 mmol), **4d** (85.5 mg, 0.50 mmol), and CuI (23.8 mg, 0.13 mmol). The product **5d** was obtained in 46% yield (49.4 mg, 0.23 mmol) as a colorless oil. ^{1}H and ^{13}C NMR of the product **5d** were in agreement with the literature.[39]

^{1}H NMR (400 MHz, $CDCl_3$, δ): 0.55 (s, 6H), 7.32–7.41 (m, 6H), 7.48–7.55 (m, 4H). ^{13}C NMR (100 MHz, $CDCl_3$, δ): –2.4 (CH_3), 127.8 (*C*H), 129.1 (*C*H), 134.2 (*C*H), 138.2 (*C*). HRMS-EI (m/z): $[M]^+$ calcd for $C_{14}H_{16}Si$, 212.1021; found, 212.1018.

2-Butylnaphthalene (7a).

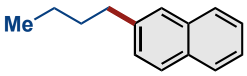

7a

Conditions H: The reaction was conducted with **1d** (69.8 mg, 0.50 mmol) and **6a** (98.8 mg, 0.33 mmol). The product **7a** was obtained in 35% NMR yield.

Conditions I: The reaction was conducted with **1d** (136.8 mg, 1.0 mmol) and **6a** (98.6 mg, 0.33 mmol). The reaction time of the second step was shorten to 30 minutes. The product **7a** was obtained in 75% yield (45.6 mg, 0.25 mmol) as a colorless oil. ^{1}H and ^{13}C NMR of the product **7a** were in agreement with the literature.[40]

^{1}H NMR (400 MHz, $CDCl_3$, δ): 0.95 (t, *J* = 7.4 Hz, 3H), 1.40 (sex, *J* = 7.4 Hz, 2H), 1.65–1.73 (m, 2H), 2.78

(t, J = 7.6 Hz, 2H), 7.34 (dd, J = 1.8, 8.4 Hz, 1H), 7.42 (dquint, J = 1.6, 7.3 Hz, 2H), 7.61 (s, 1H), 7.73–7.82 (m, 3H). ^{13}C NMR (99 MHz, $CDCl_3$, δ): 14.0 (CH_3), 22.4 (CH_2), 33.5 (CH_2), 35.8 (CH_2), 124.9 (*C*H), 125.8 (*C*H), 126.3 (*C*H), 127.36 (*C*H), 127.44 (*C*H), 127.6 (*C*H), 127.7 (*C*H), 131.9 (*C*), 133.6 (*C*), 140.4 (*C*). HRMS-EI (m/z): $[M]^+$ calcd for $C_{14}H_{16}$, 184.1252; found, 184.1250.

1-Butylnaphthalene (7b).

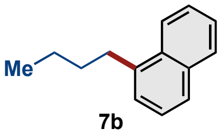

7b

Conditions H: The reaction was conducted with **1d** (69.1 mg, 0.50 mmol) and **6b** (98.6 mg, 0.33 mmol). The product **7b** was obtained in 44% NMR yield.

Conditions I: The reaction was conducted with **1d** (136.5 mg, 1.0 mmol) and **6b** (98.6 mg, 0.33 mmol). The reaction time of the second step was shorten to 30 minutes. The product **7b** was obtained in 70% yield (42.6 mg, 0.23 mmol) as a colorless oil. ^{1}H and ^{13}C NMR of the product **7b** were in agreement with the literature.[40]

^{1}H NMR (400 MHz, $CDCl_3$, δ): 0.97 (t, J = 7.4 Hz, 3H), 1.46 (sex, J = 7.4 Hz, 2H), 1.51–1.70 (m, 2H), 3.07 (t, J = 8.0 Hz, 2H), 7.32 (d, J = 6.8 Hz, 1H), 7.39 (t, J = 7.6 Hz, 1H), 7.44–7.53 (m, 2H), 7.70 (d, J = 8.0 Hz, 1H), 7.85 (d, J = 7.6 Hz, 1H), 8.05 (d, J = 8.4 Hz, 1H). ^{13}C NMR (100 MHz, $CDCl_3$, δ): 14.0 (CH_3), 22.9 (CH_2), 32.8 (CH_2), 33.0 (CH_2), 123.9 (*C*H), 125.3 (*C*H), 125.50 (*C*H), 125.57 (*C*H), 125.8 (*C*H), 126.3 (*C*H), 128.7 (*C*H), 131.9 (*C*), 133.9 (*C*), 139.0 (*C*). HRMS-EI (m/z): $[M]^+$ calcd for $C_{14}H_{16}$, 184.1252; found, 184.1252.

2-Octylnaphthalene (7c).

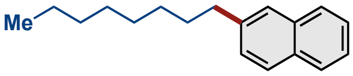

7c

Conditions H: The reaction was conducted with **1m** (96.0 mg, 0.50 mmol) and **6a** (98.8 mg, 0.33 mmol). The product **7c** was obtained in 31% NMR yield.

Conditions I: The reaction was conducted with **1m** (193.7 mg, 1.0 mmol) and **6a** (98.5 mg, 0.33 mmol). The product **7c** was obtained in 88% yield (69.7 mg, 0.29 mmol) as a colorless oil. ^{1}H and ^{13}C NMR of the product **7c** were in agreement with the literature.[40]

^{1}H NMR (400 MHz, $CDCl_3$, δ): 0.88 (t, J = 6.8 Hz, 3H), 1.18–1.42 (m, 10H), 1.70 (quint, J = 7.5 Hz, 2H), 2.76 (t, J = 7.6 Hz, 2H), 7.33 (dd, J = 1.8, 8.6 Hz, 1H), 7.42 (dquint, J = 1.4, 6.8 Hz, 2H), 7.61 (s, 1H), 7.73–7.83 (m, 3H). ^{13}C NMR (101 MHz, $CDCl_3$, δ): 14.1 (CH_3), 22.7 (CH_2), 29.3 (CH_2), 29.4 (CH_2), 29.5 (CH_2), 31.4 (CH_2), 31.9 (CH_2), 36.1 (CH_2), 124.9 (*C*H), 125.8 (*C*H), 126.3 (*C*H), 127.37 (*C*H), 127.43 (*C*H), 127.6 (*C*H), 127.7 (*C*H), 131.9 (*C*), 133.6 (*C*), 140.4 (*C*). HRMS-EI (m/z): $[M]^+$ calcd for $C_{18}H_{24}$, 240.1878; found, 240.1876.

2-Butyl-6-methoxynaphthalene (7d).

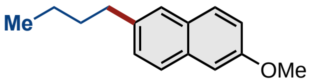

7d

Conditions H: The reaction was conducted with **1d** (67.7 mg, 0.50 mmol) and **6c** (108.4 mg, 0.33 mmol). The product **7d** was obtained in 26% NMR yield.

Conditions I: The reaction was conducted with **1d** (107 μL, 1.0 mmol) and **6c** (108.2 mg, 0.33 mmol). The product **7d** was obtained in 36% yield (25.7 mg, 0.12 mmol) as a white solid. ^{1}H and ^{13}C NMR of the product **7d** were in agreement with the literature.[41]

^{1}H NMR (400 MHz, $CDCl_3$, δ): 0.94 (t, *J* = 7.2 Hz, 3H), 1.33–1.44 (m, 2H), 1.63–1.72 (m, 2H), 2.74 (t, *J* = 7.6 Hz, 2H), 3.91 (s, 3H), 7.10–7.13 (m, 2H), 7.30 (dd, *J* = 2.0, 8.6 Hz, 1H), 7.54 (s, 1H), 7.64–7.69 (m, 2H). ^{13}C NMR (99 MHz, $CDCl_3$, δ): 14.0 (CH_3), 22.4 (CH_2), 33.6 (CH_2), 35.6 (CH_2), 55.2 (CH_3), 105.6 (*C*H), 118.5 (*C*H), 126.1 (*C*H), 126.6 (*C*H), 127.9 (*C*H), 128.8 (*C*H), 129.1 (*C*), 132.8 (*C*), 138.1 (*C*), 157.0 (*C*). HRMS-EI (m/z): $[M]^+$ calcd for $C_{15}H_{18}O$, 214.1358; found, 214.1358.

2-Butyl-6-(4-fluorophenyl)naphthalene (7e).

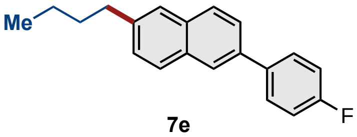

7e

Conditions H: The reaction was conducted with **1d** (69.2 mg, 0.50 mmol) and **6d** (129.2 mg, 0.33 mmol). The product **7d** was obtained in 41% NMR yield.

Conditions I: The reaction was conducted with **1d** (137.0 mg, 1.0 mmol) and **6d** (129.7 mg, 0.33 mmol). The product **7d** was obtained in 79% yield (72.4 mg, 0.26 mmol) as a white solid.

^{1}H NMR (396 MHz, $CDCl_3$, δ): 0.96 (t, *J* = 7.3 Hz, 3H), 1.41 (sex, *J* = 7.4 Hz, 2H), 1.65–1.75 (m, 2H), 2.79 (t, *J* = 7.7 Hz, 2H), 7.16 (tt, *J* = 2.5, 9.1 Hz, 2H), 7.37 (dd, *J* = 1.4, 8.5 Hz, 1H), 7.61–7.69 (m, 4H), 7.81 (d, *J* = 8.3 Hz, 1H), 7.84 (d, *J* = 8.3 Hz, 1H), 7.94 (s, 1H). ^{13}C NMR (100 MHz, $CDCl_3$, δ): 14.0 (CH_3), 22.4 (CH_2), 33.5 (CH_2), 35.8 (CH_2), 115.6 (d, *J* = 21.0 Hz, *C*H), 125.3 (*C*H), 125.4 (*C*H), 126.0 (*C*H), 127.9 (*C*H), 127.99 (*C*H), 128.01 (*C*H), 128.8 (d, *J* = 7.6 Hz, *C*H), 132.1 (*C*), 132.7 (*C*), 136.7 (*C*), 137.4 (d, *J* = 2.9 Hz, *C*),140.7 (*C*), 162.4 (d, *J* = 247.0 Hz, *C*H). HRMS-EI (m/z): $[M]^+$ calcd for $C_{20}H_{19}F$, 278.1471; found, 278.1465.

2-Phenethylnaphthalene (7f).

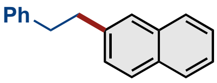

7f

Conditions H: The reaction was conducted with **1n** (93.2 mg, 0.50 mmol) and **6a** (98.4 mg, 0.33 mmol). The product **7f** was obtained in 16% NMR yield.

Conditions I: The reaction was conducted with **1n** (185.5 mg, 1.0 mmol) and **6a** (98.6 mg, 0.33 mmol). The product **7f** was obtained in 78% yield (59.6 mg, 0.26 mmol) as a white solid. ^{1}H and ^{13}C NMR of the product **7f** were in agreement with the literature.[42]

^{1}H NMR (400 MHz, $CDCl_3$, δ): 2.96–3.03 (m, 2H), 3.04–3.12 (m, 2H), 7.16–7.23 (m, 3H), 7.24–7.30 (m, 2H), 7.32 (dd, *J* = 1.6, 8.4 Hz, 1H), 7.37–7.47 (m, 2H), 7.60 (s, 1H), 7.72–7.84 (m, 3H). ^{13}C NMR (101 MHz, $CDCl_3$, δ): 37.8 (CH_2), 38.1 (CH_2), 125.1 (*C*H), 125.85 (*C*H), 125.92 (*C*H), 126.4 (*C*H), 127.3 (*C*H), 127.4 (*C*H), 127.6 (*C*H), 127.8 (*C*H), 128.3 (*C*H), 128.5 (*C*H), 132.0 (*C*), 133.6 (*C*), 139.2 (*C*), 141.7 (*C*). HRMS-EI (m/z): $[M]^+$ calcd for $C_{18}H_{16}$, 232.1252; found, 232.1244.

2-(4-methoxybutyl)naphthalene (7g).

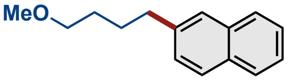

7g

Conditions H: The reaction was conducted with **1o** (83.8 mg, 0.50 mmol) and **6a** (98.4 mg, 0.33 mmol). The product **7g** was obtained in 21% NMR yield.

Conditions I: The reaction was conducted with **1o** (167.7 mg, 1.0 mmol) and **6a** (98.6 mg, 0.33 mmol). The product **7g** was obtained in 87% yield (61.8 mg, 0.29 mmol) as a colorless oil. ^{1}H and ^{13}C NMR of the product **7g** were in agreement with the literature.[42]

^{1}H NMR (400 MHz, $CDCl_3$, δ): 1.61–1.70 (m, 2H), 1.73–1.83 (m, 2H), 2.80 (t, *J* = 7.4 Hz, 2H), 3.33 (s, 3H), 3.41 (t, *J* = 6.5 Hz, 2H), 7.34 (dd, *J* = 1.6, 8.2 Hz, 1H), 7.43 (dquint, *J* = 1.2, 6.8 Hz, 2H), 7.62 (s, 1H), 7.75–7.82 (m, 3H). ^{13}C NMR (99 MHz, $CDCl_3$, δ): 27.8 (CH_2), 29.3 (CH_2), 35.8 (CH_2), 58.5 (CH_3), 72.6 (CH_2), 125.0 (*C*H), 125.8 (*C*H), 126.3 (*C*H), 127.31 (*C*H), 127.35 (*C*H), 127.5 (*C*H), 127.7 (*C*H), 131.9 (*C*), 133.6 (*C*), 139.9 (*C*). HRMS-EI (m/z): $[M]^+$ calcd for $C_{15}H_{18}O$, 214.1358; found, 214.1357.

2-Neopentylnaphthalene (7h).

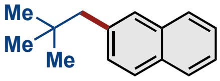

7h

Conditions H: The reaction was conducted with **1p** (75.5 mg, 0.50 mmol) and **6a** (98.3 mg, 0.33 mmol). The product **7h** was obtained in 4% NMR yield.

Conditions I: The reaction was conducted with **1p** (151.5 mg, 1.0 mmol) and **6a** (98.4 mg, 0.33 mmol). The reaction time of the second step was 30 minutes. The product **7h** was obtained in 43% yield (27.9 mg, 0.25 mmol) as a white solid. ^{1}H and ^{13}C NMR of the product **7h** were in agreement with the literature.[43]

^{1}H NMR (400 MHz, $CDCl_3$, δ): 0.95 (s, 9H), 2.65 (s, 2H), 7.28 (d, *J* = 8.4 Hz, 1H), 7.42 (quint, *J* = 6.9 Hz, 2H), 7.56 (s, 1H), 7.73 (d, *J* = 8.4 Hz, 1H), 7.79 (t, *J* = 8.2 Hz, 2H). ^{13}C NMR (101 MHz, $CDCl_3$, δ): 29.5 (CH_3), 32.1 (*C*), 50.3 (CH_2), 125.0 (*C*H), 125.7 (*C*H), 126.8 (*C*H), 127.50 (*C*H), 127.52 (*C*H), 128.6 (*C*H), 129.5 (*C*H), 131.9 (*C*), 133.2 (*C*), 137.4 (*C*). HRMS-EI (m/z): $[M]^+$ calcd for $C_{15}H_{18}$, 198.1409; found, 198.1412.

2-Cyclohexylnaphthalene (7i).

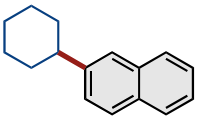

7i

Conditions H: The reaction was conducted with **1e** (82.7 mg, 0.50 mmol) and **6a** (98.4 mg, 0.33 mmol). The product **7i** was not obtained.

Conditions I: The reaction was conducted with **1e** (164.0 mg, 1.0 mmol) and **6a** (98.5 mg, 0.33 mmol). The reaction time was 90 minutes for the first step and 30 minutes for the second step. The product **7i** was obtained in 40% yield (27.5 mg, 0.13 mmol) as a pale orange oil. ^{1}H and ^{13}C NMR of the product **7i** were in agreement with the literature.[40]

^{1}H NMR (400 MHz, $CDCl_3$, δ): 1.29 (tq, *J* = 4.0, 12.5 Hz, 1H), 1.38–1.60 (m, 4H), 1.74–1.82 (m, 1H), 1.84–1.92 (m, 2H), 1.93–2.01 (m, 2H), 2.66 (tt, *J* = 3.3, 11.5 Hz, 1H), 7.35–7.48 (m, 3H), 7.63 (s, 1H), 7.74–7.83 (m, 3H). ^{13}C NMR (101 MHz, $CDCl_3$, δ): 26.2 (CH_2), 26.9 (CH_2), 34.4 (CH_2), 44.6 (*C*H), 124.5 (*C*H), 125.0 (*C*H), 125.7 (*C*H), 126.2 (*C*H), 127.51 (*C*H), 127.55 (*C*H), 127.7 (*C*H), 132.1 (*C*), 133.6 (*C*), 145.5 (*C*). HRMS-EI (m/z): [M] calcd for $C_{16}H_{18}$, 210.1409; found, 210.1403.

NMR spectra

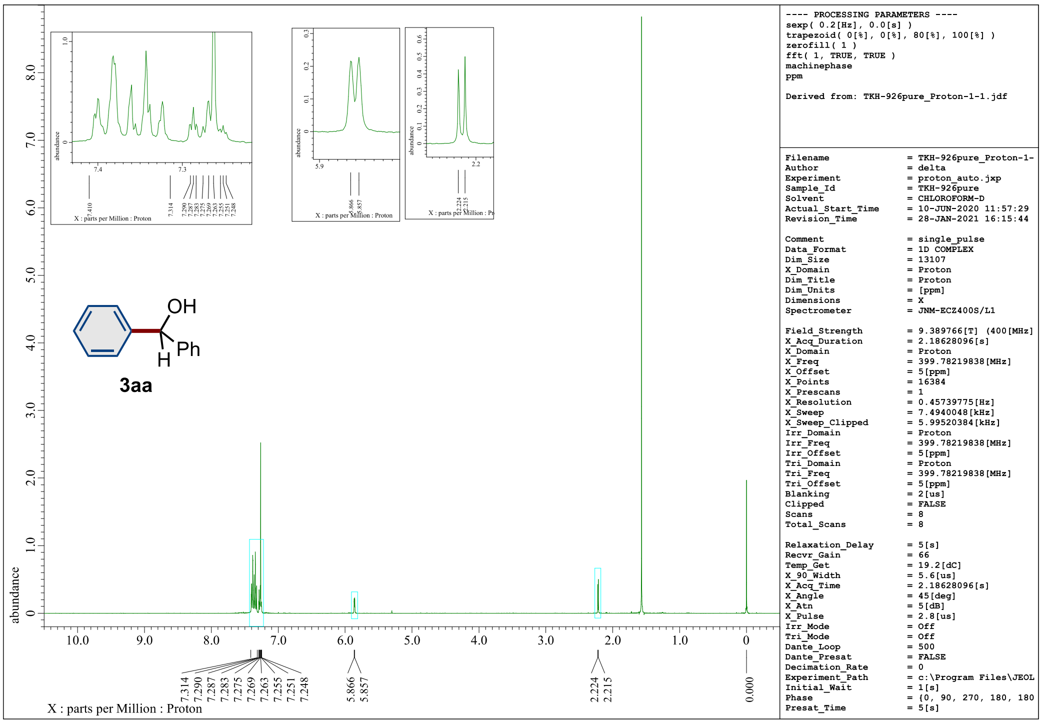

Supplementary Figure 14. ^{1}H NMR spectrum of **3aa**.

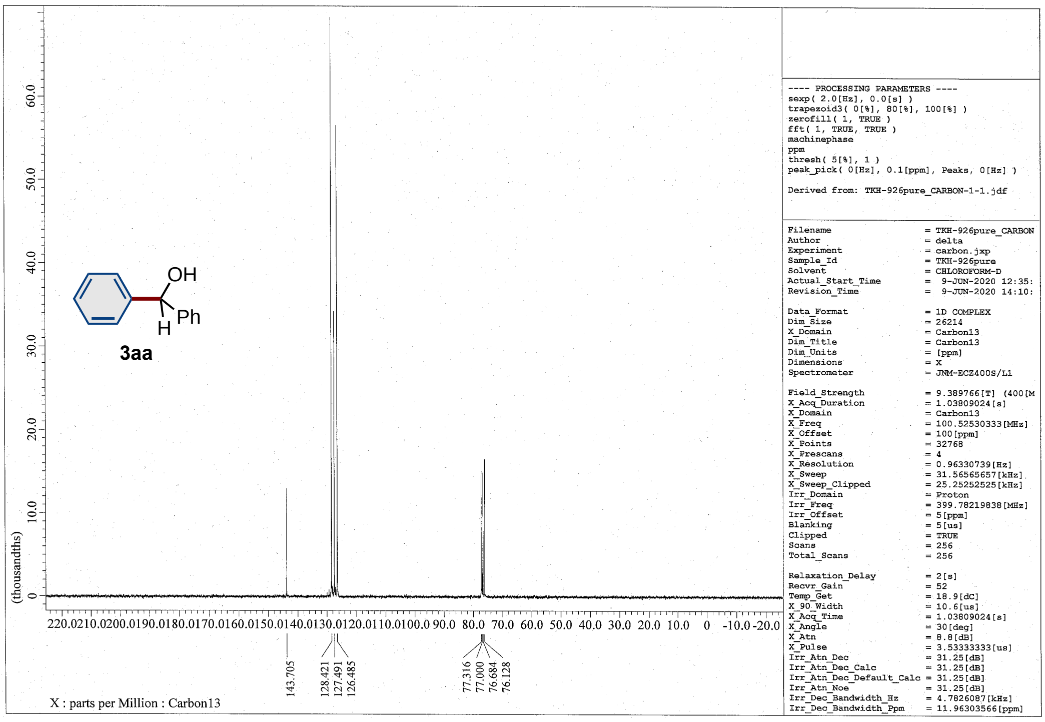

Supplementary Figure 15. ^{13}C NMR spectrum of **3aa**.

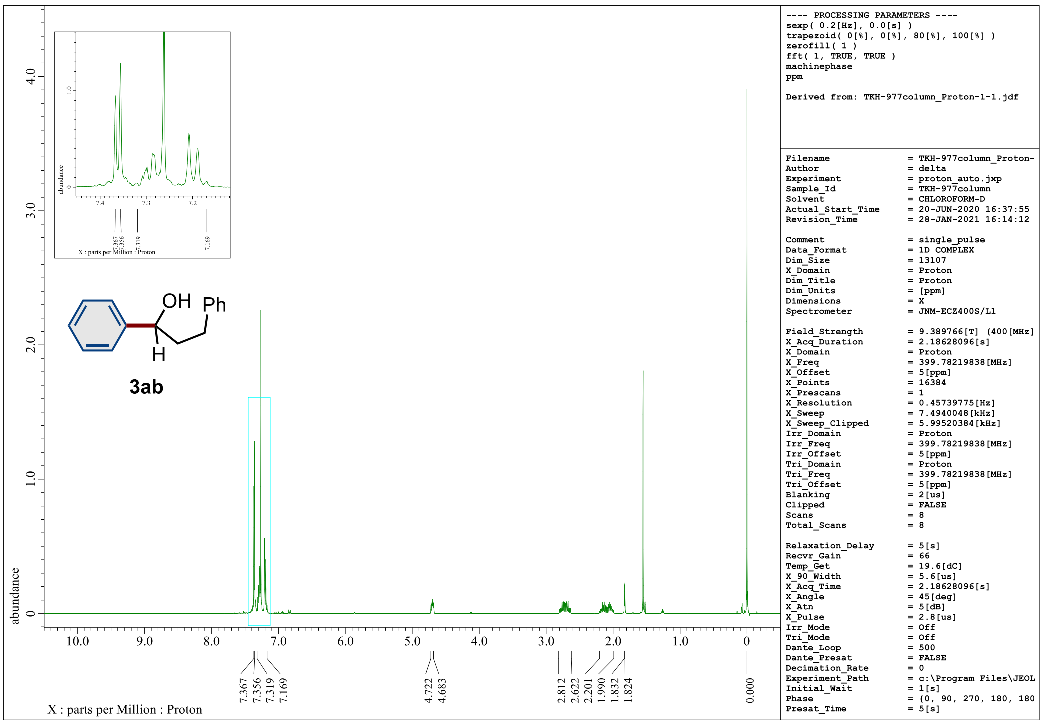

Supplementary Figure 16. ^{1}H NMR spectrum of **3ab**.

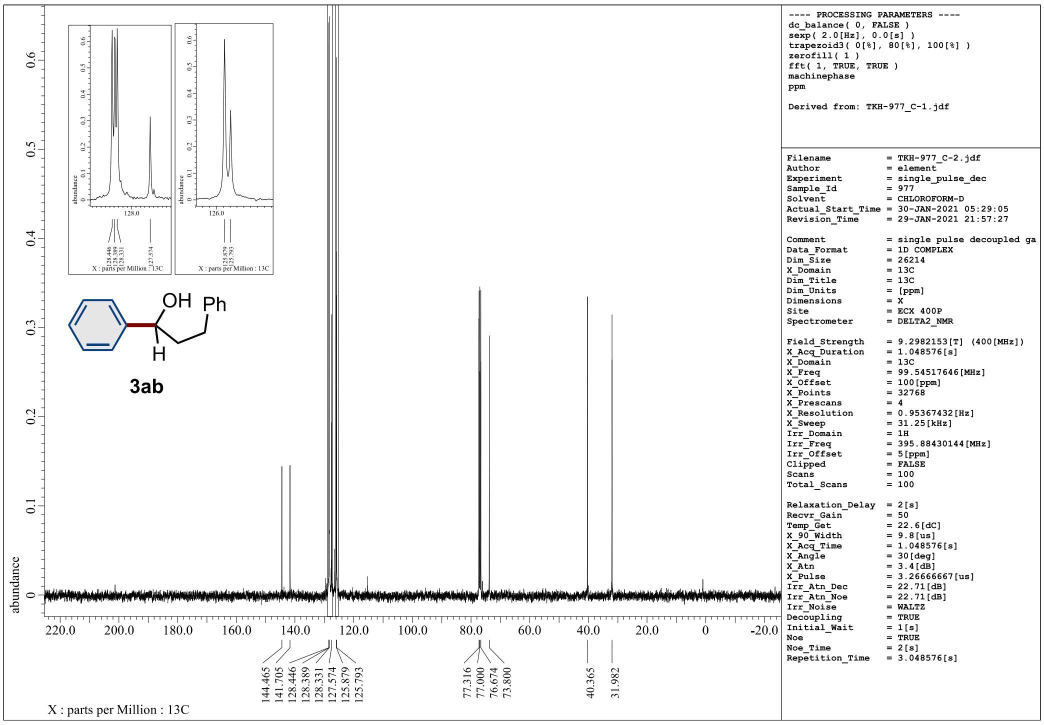

Supplementary Figure 17. ^{13}C NMR spectrum of **3ab**.

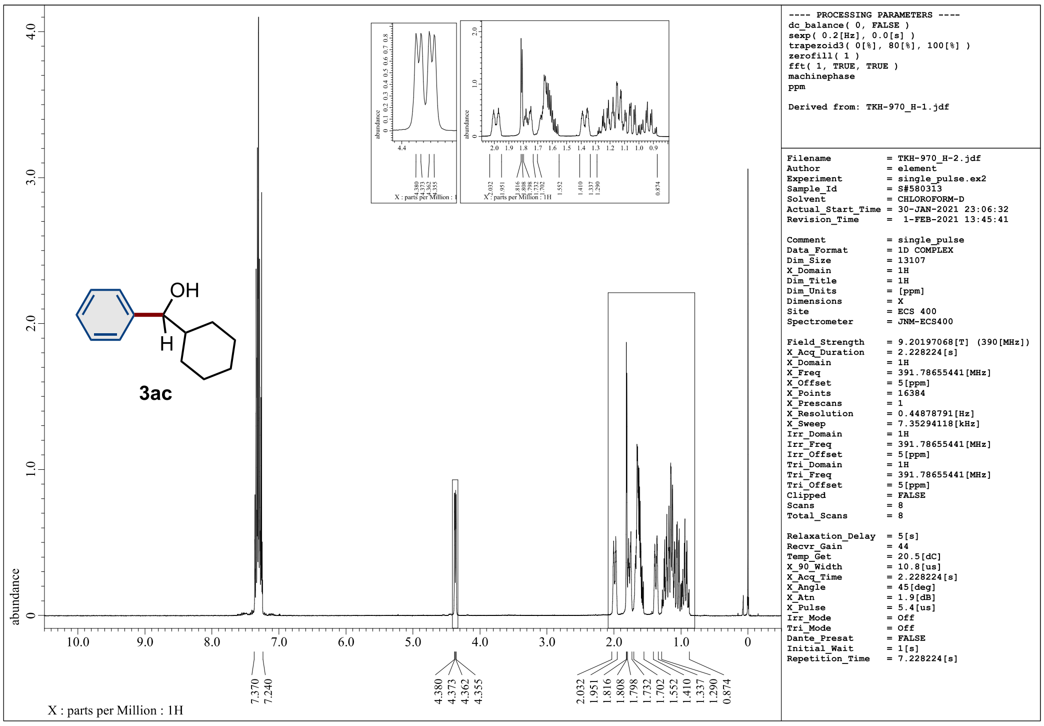

Supplementary Figure 18. ^{1}H NMR spectrum of **3ac**.

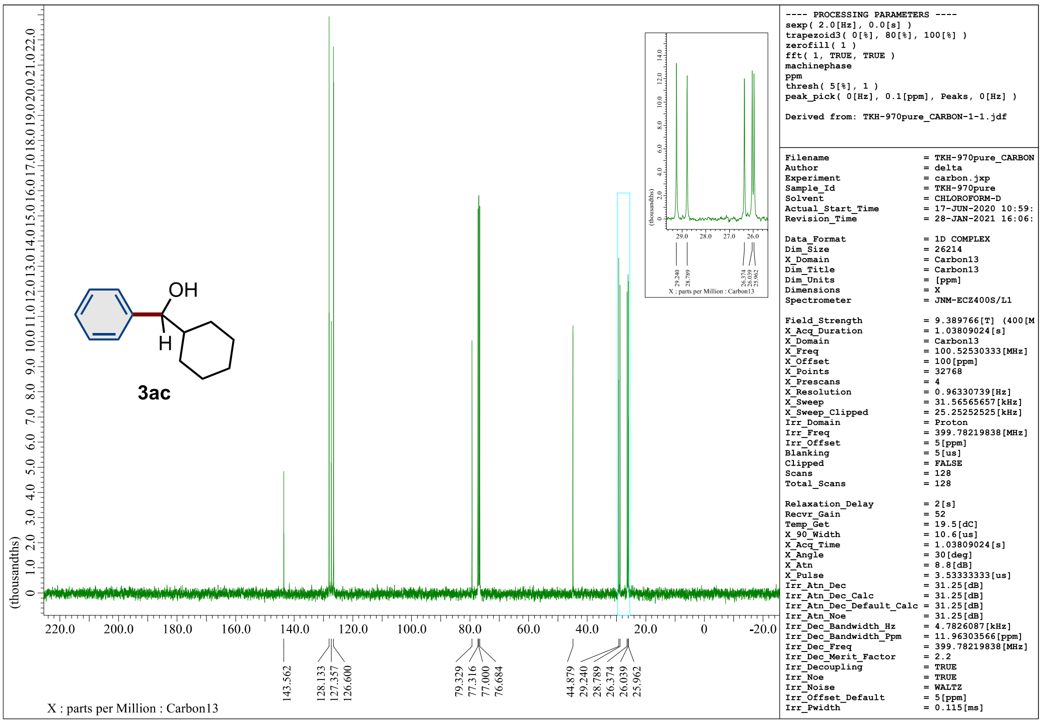

Supplementary Figure 19. ^{13}C NMR spectrum of **3ac**.

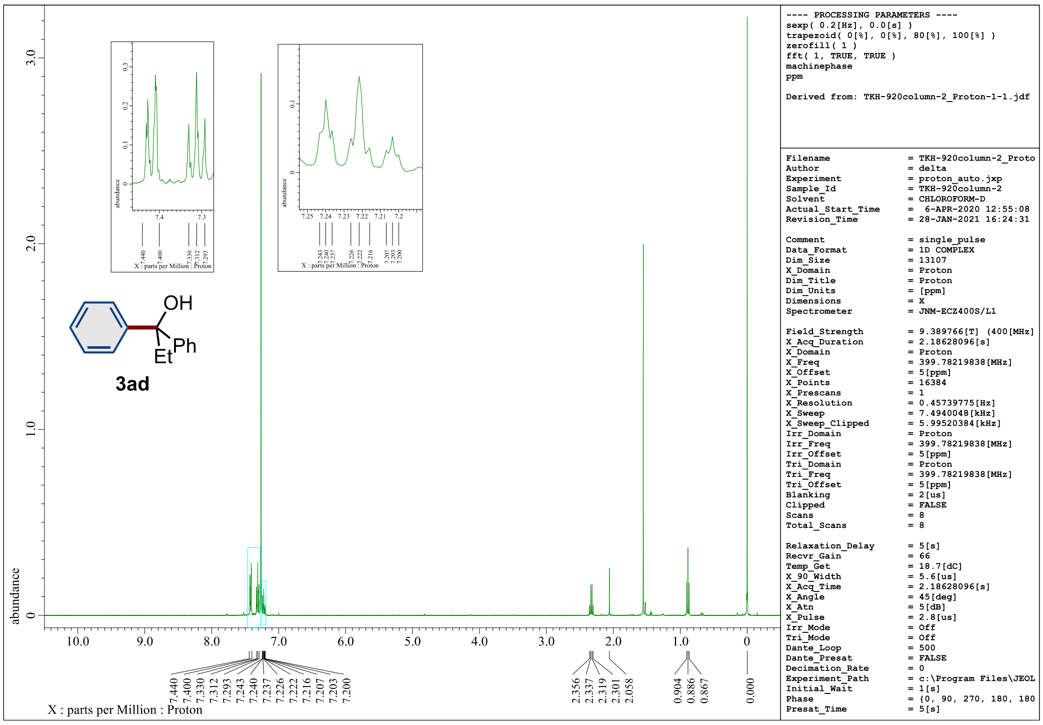

Supplementary Figure 20. ^{1}H NMR spectrum of **3ad**.

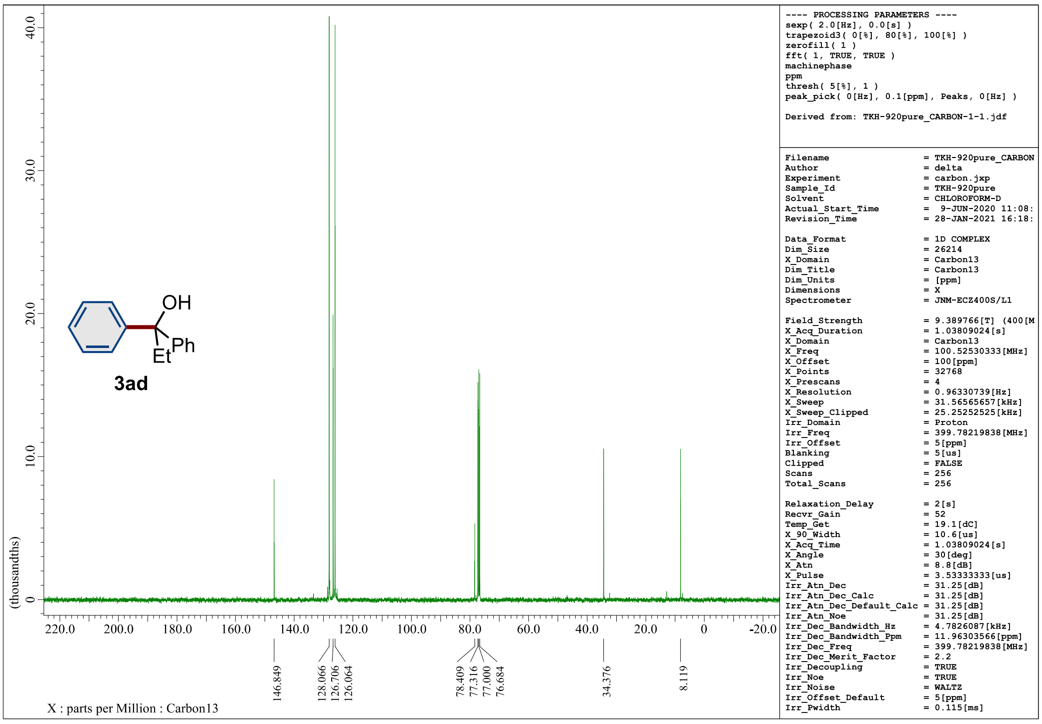

Supplementary Figure 21. ^{13}C NMR spectrum of **3ad**.

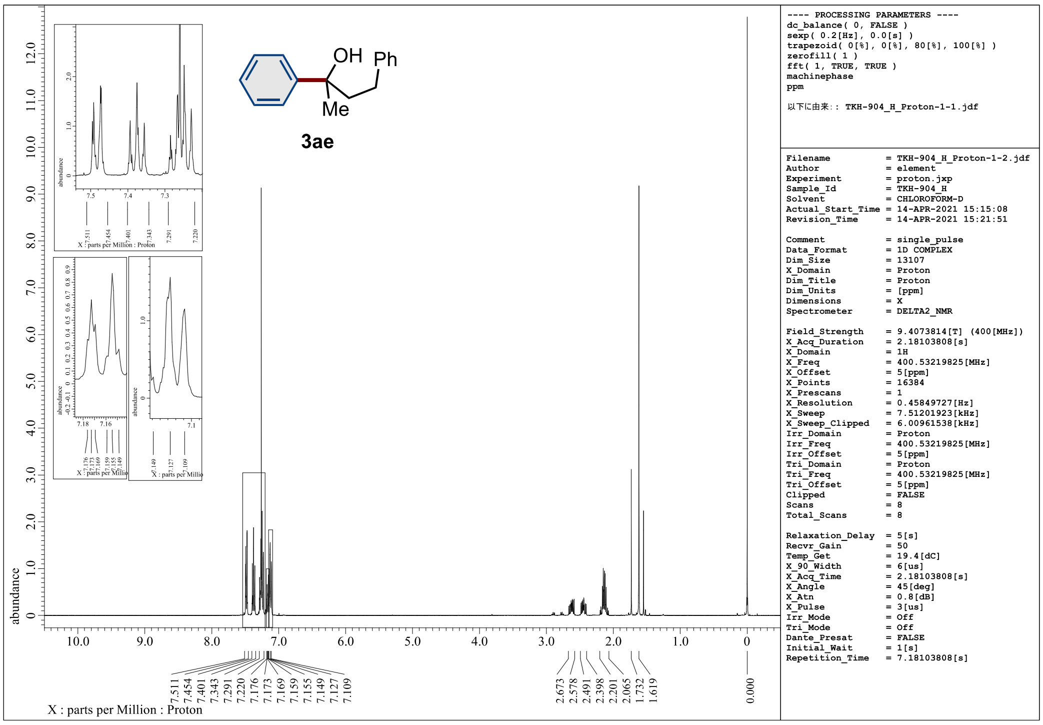

Supplementary Figure 22. ^{1}H NMR spectrum of **3ae**.

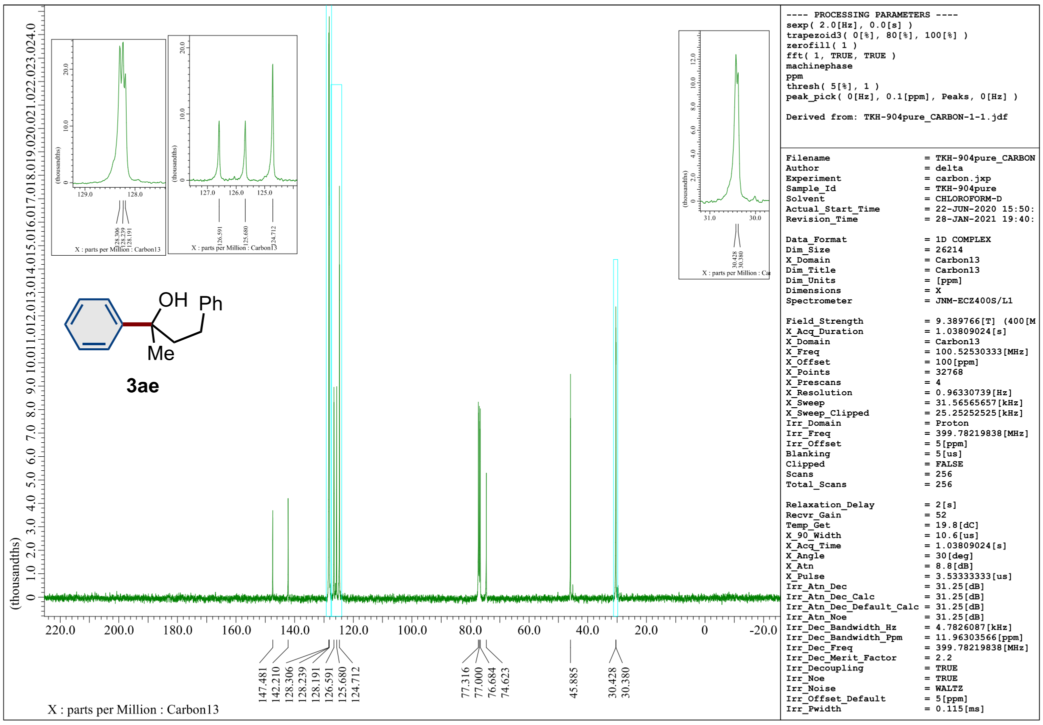

Supplementary Figure 23. ^{13}C NMR spectrum of **3ae**.

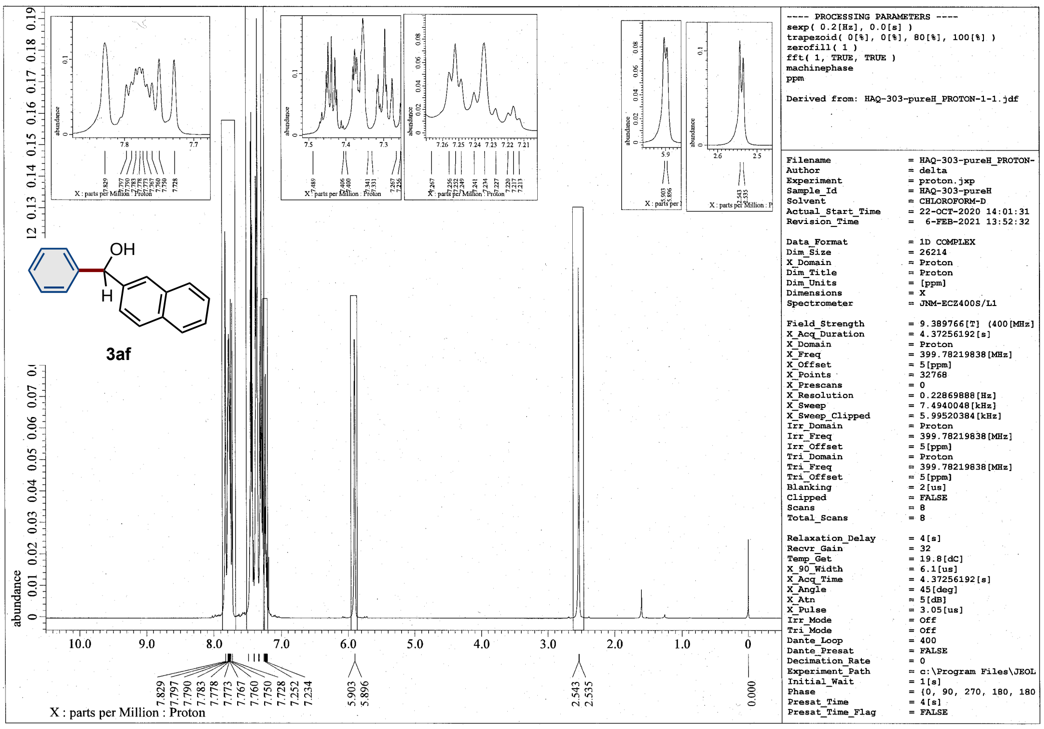

Supplementary Figure 24. ^{1}H NMR spectrum of **3af**.

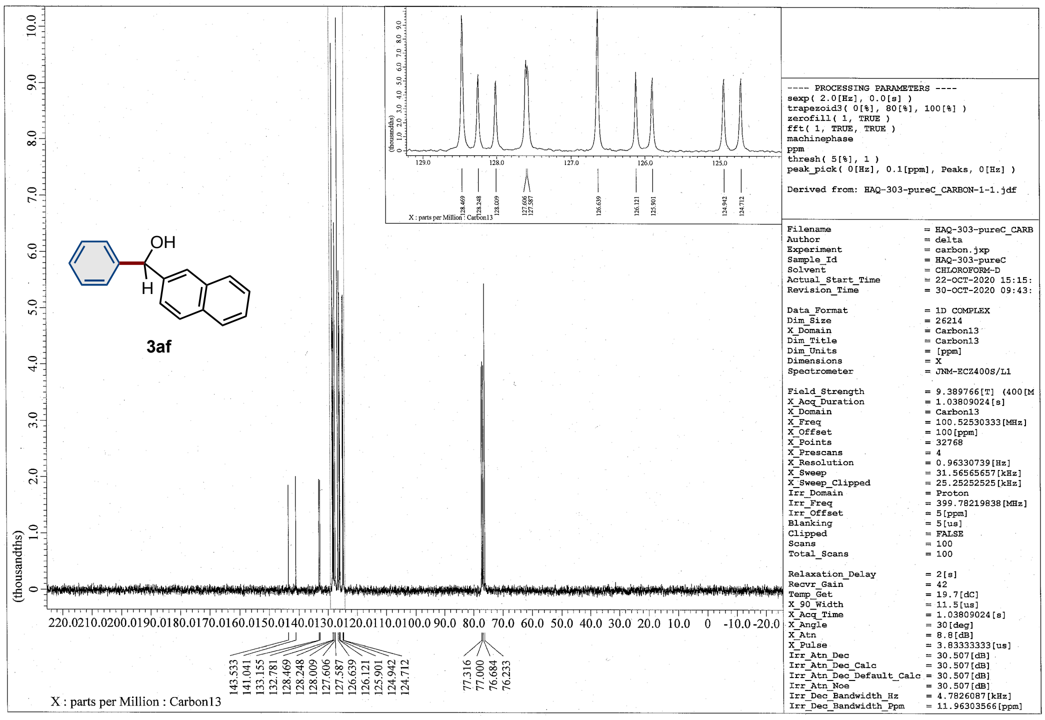

Supplementary Figure 25. ^{13}C NMR spectrum of **3af**.

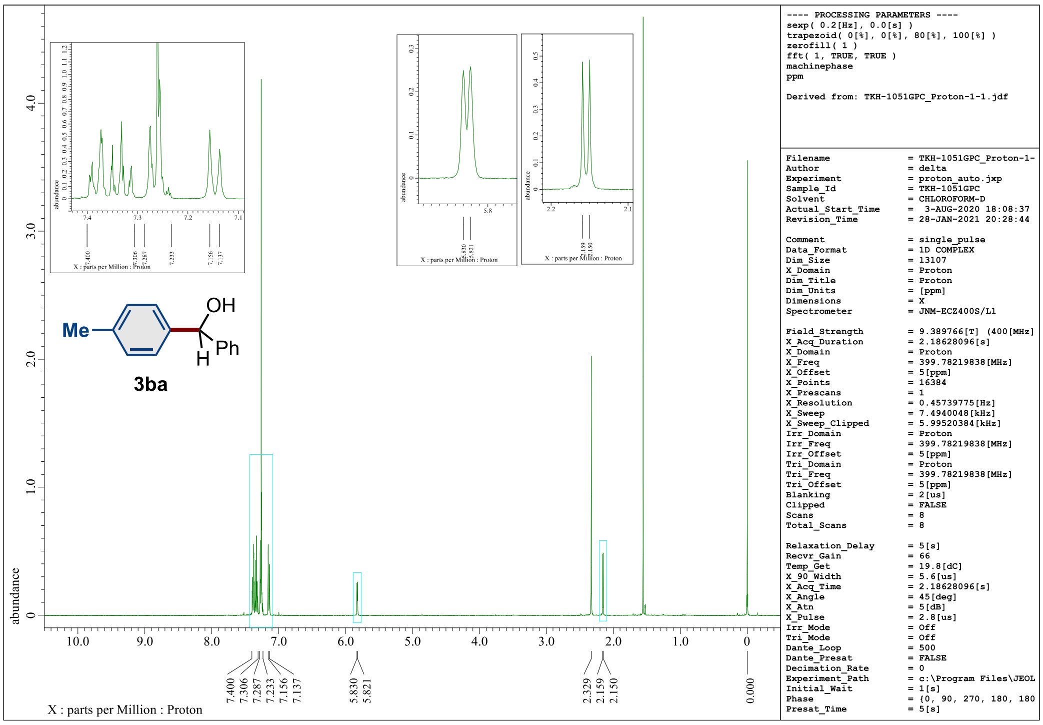

Supplementary Figure 26. ^{1}H NMR spectrum of **3ba**.

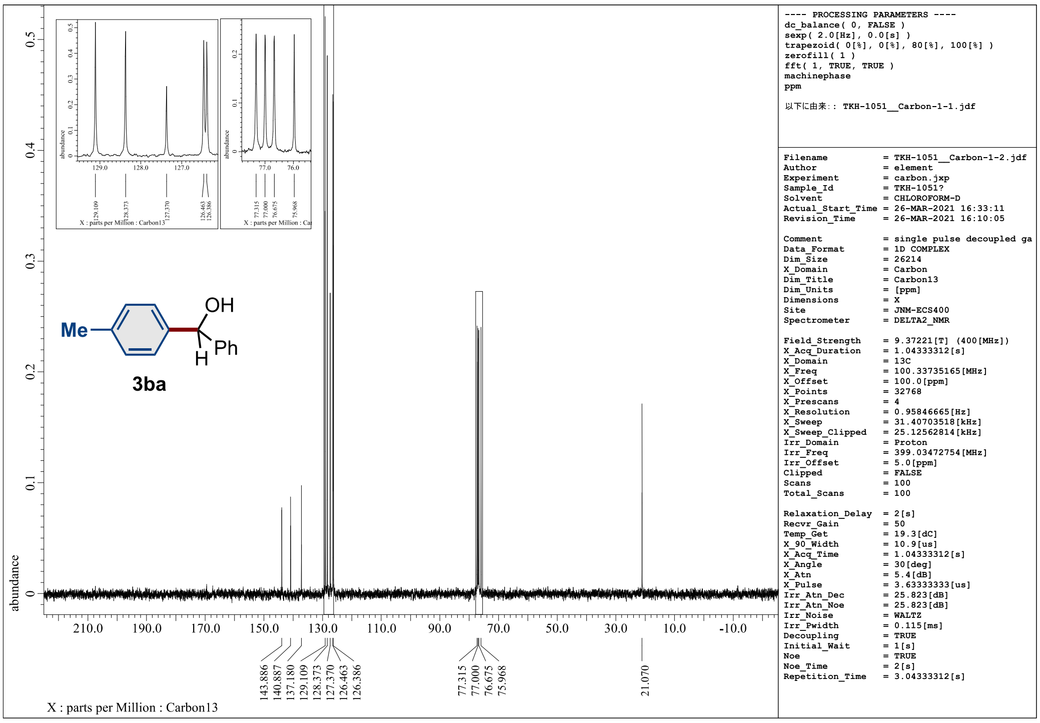

Supplementary Figure 27. ^{13}C NMR spectrum of **3ba**.

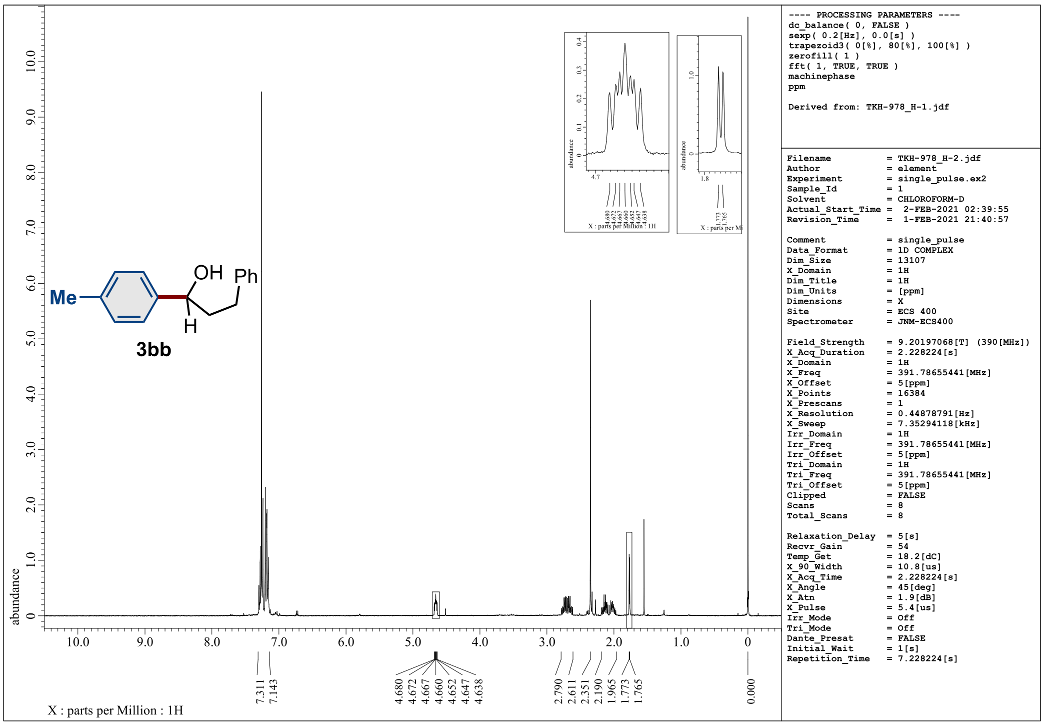

Supplementary Figure 28. ^{1}H NMR spectrum of **3bb**.

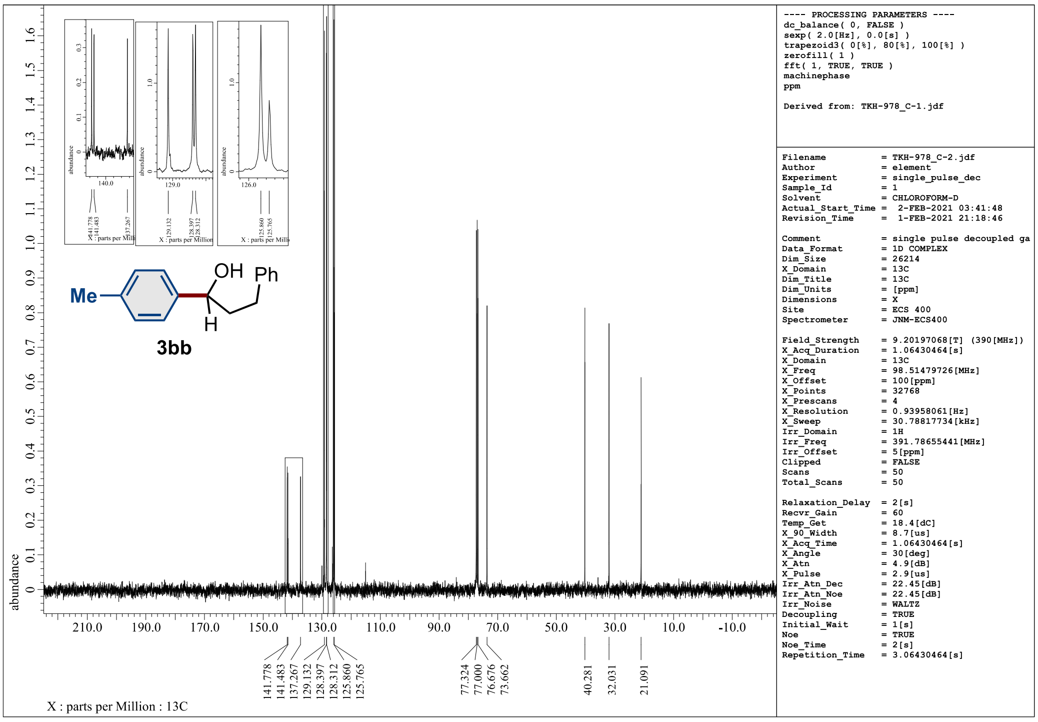

Supplementary Figure 29. ^{13}C NMR spectrum of **3bb**.

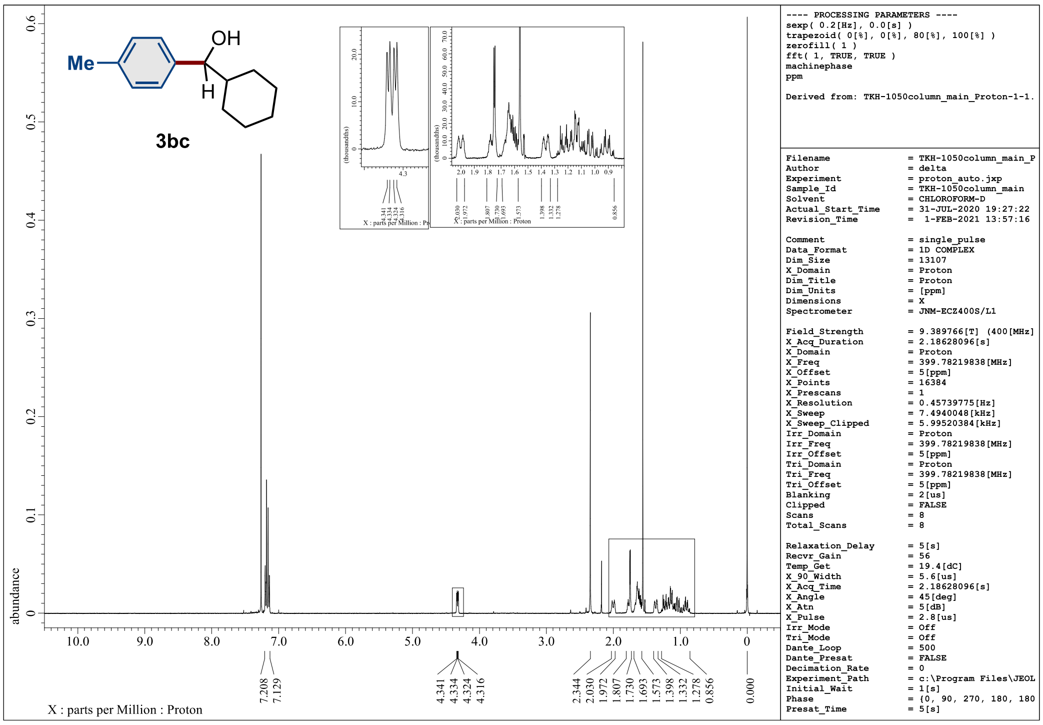

Supplementary Figure 30. ^{1}H NMR spectrum of **3bc**.

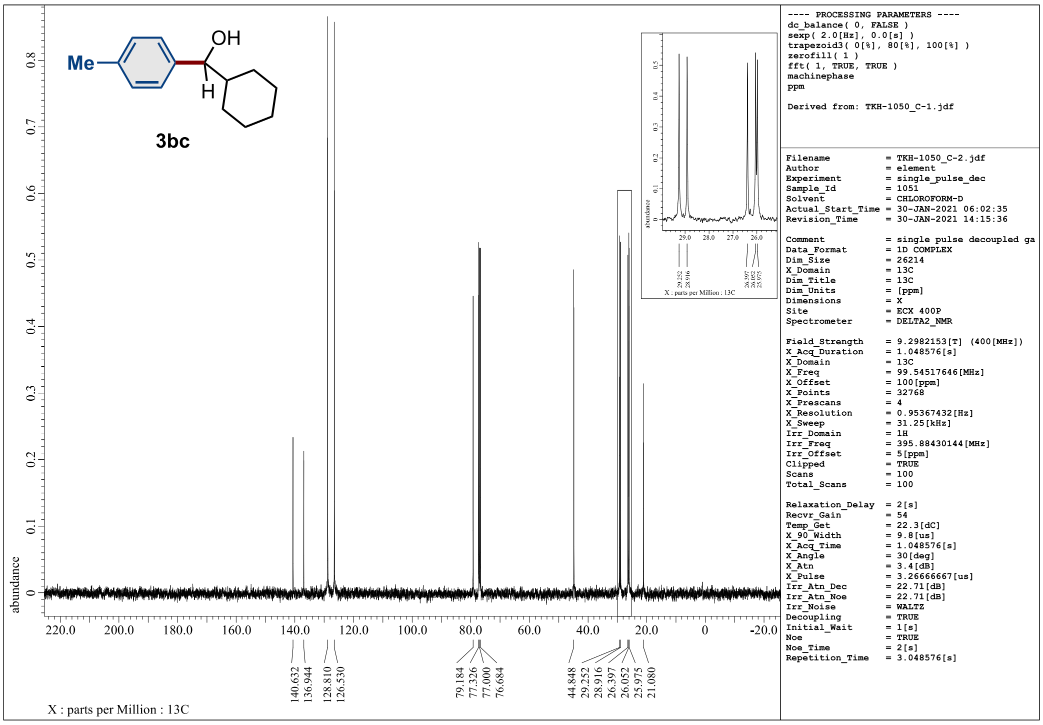

Supplementary Figure 31. ^{13}C NMR spectrum of **3bc**.

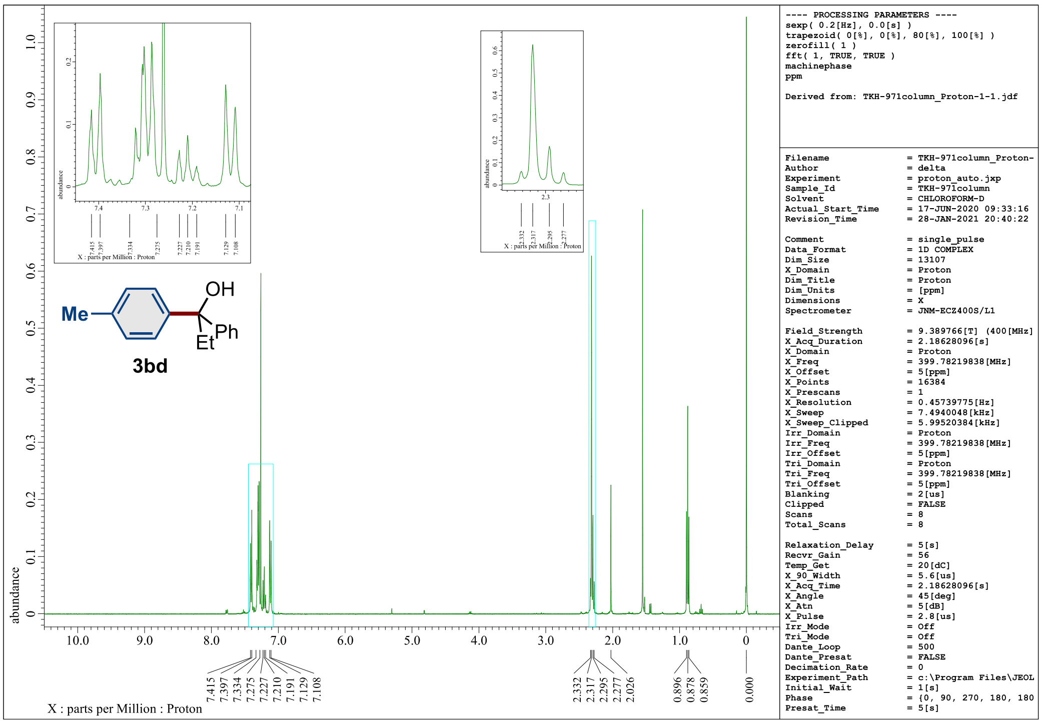

Supplementary Figure 32. ^{1}H NMR spectrum of **3bd**.

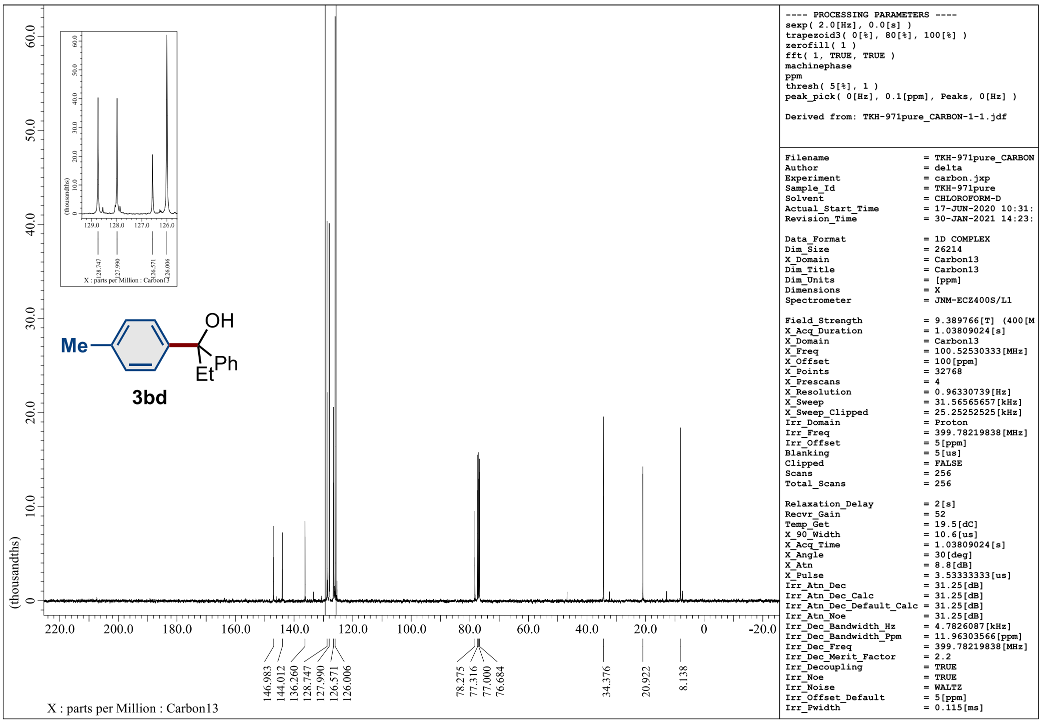

Supplementary Figure 33. ^{13}C NMR spectrum of **3bd**.

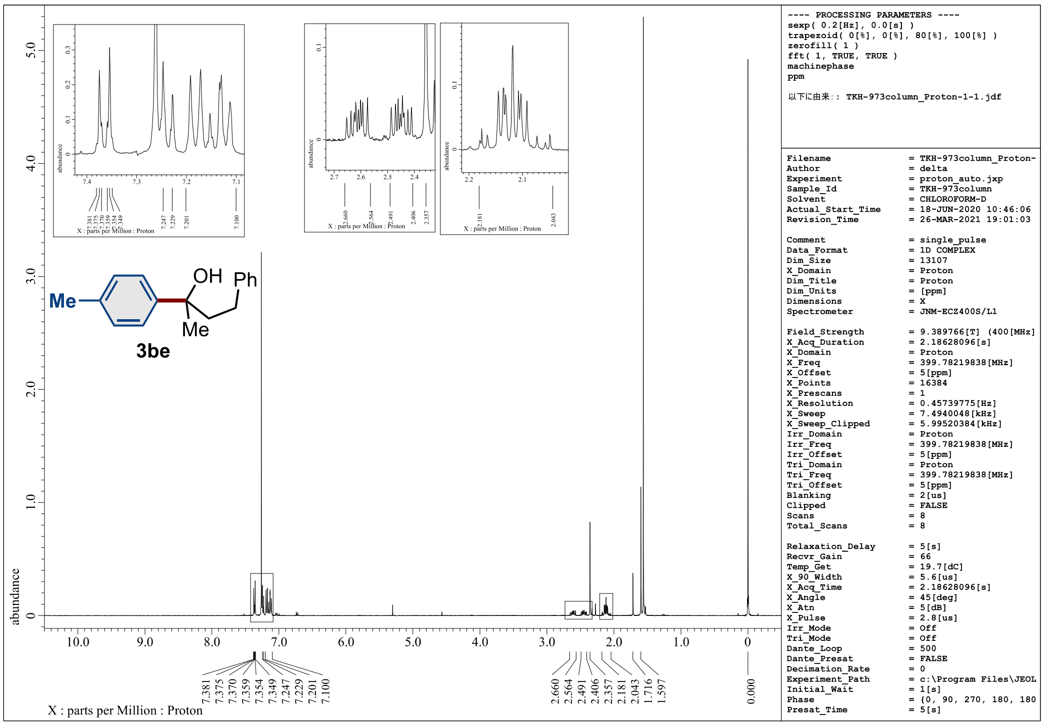

Supplementary Figure 34. ^{1}H NMR spectrum of **3be**.

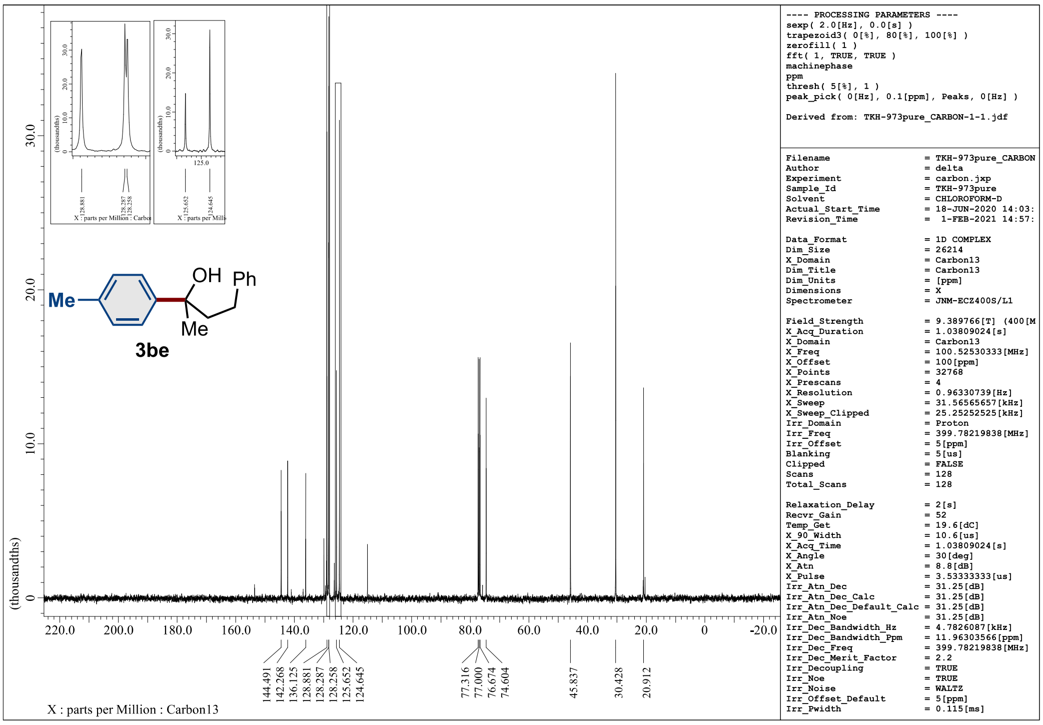

Supplementary Figure 35. ^{13}C NMR spectrum of **3be**.

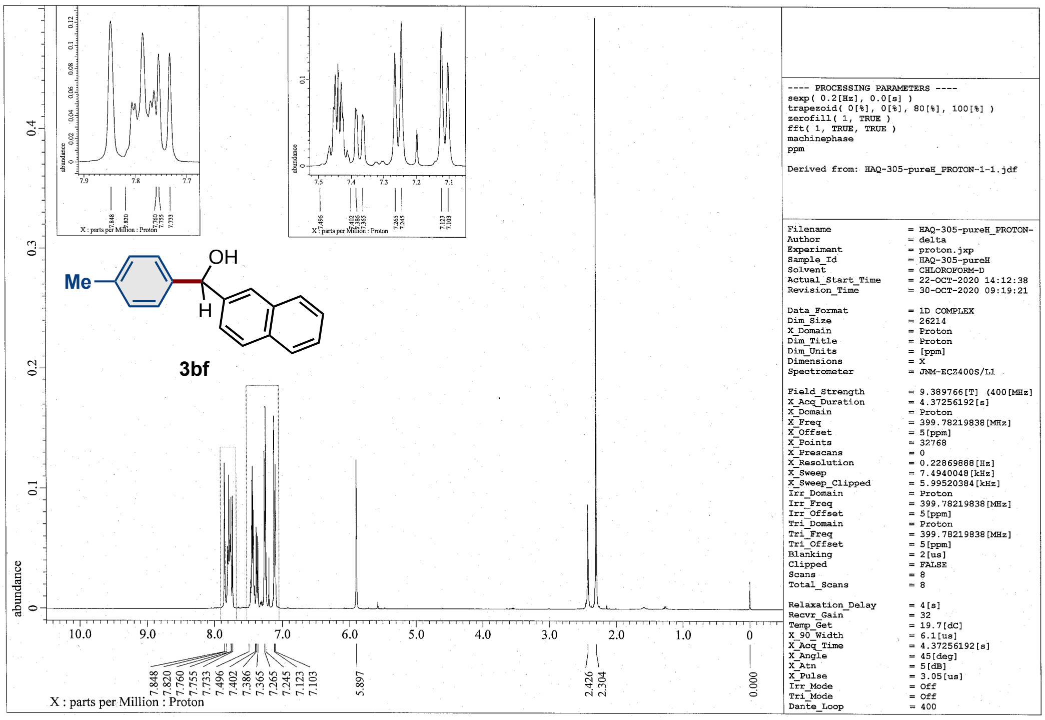

Supplementary Figure 36. ^{1}H NMR spectrum of **3bf**.

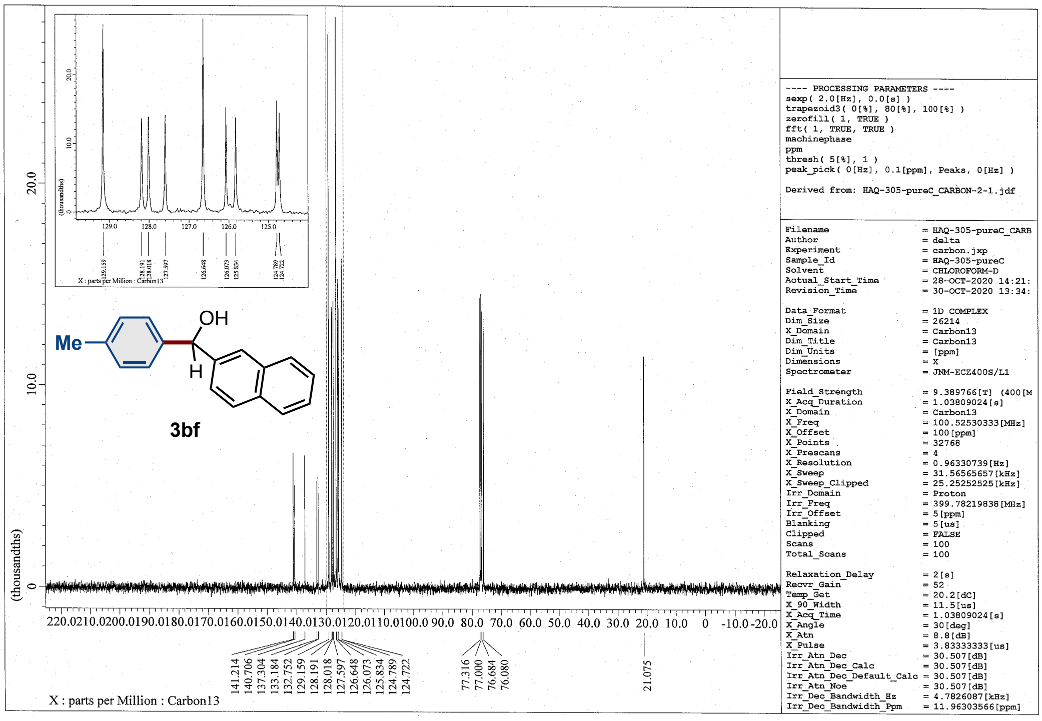

Supplementary Figure 37. ^{13}C NMR spectrum of **3bf**.

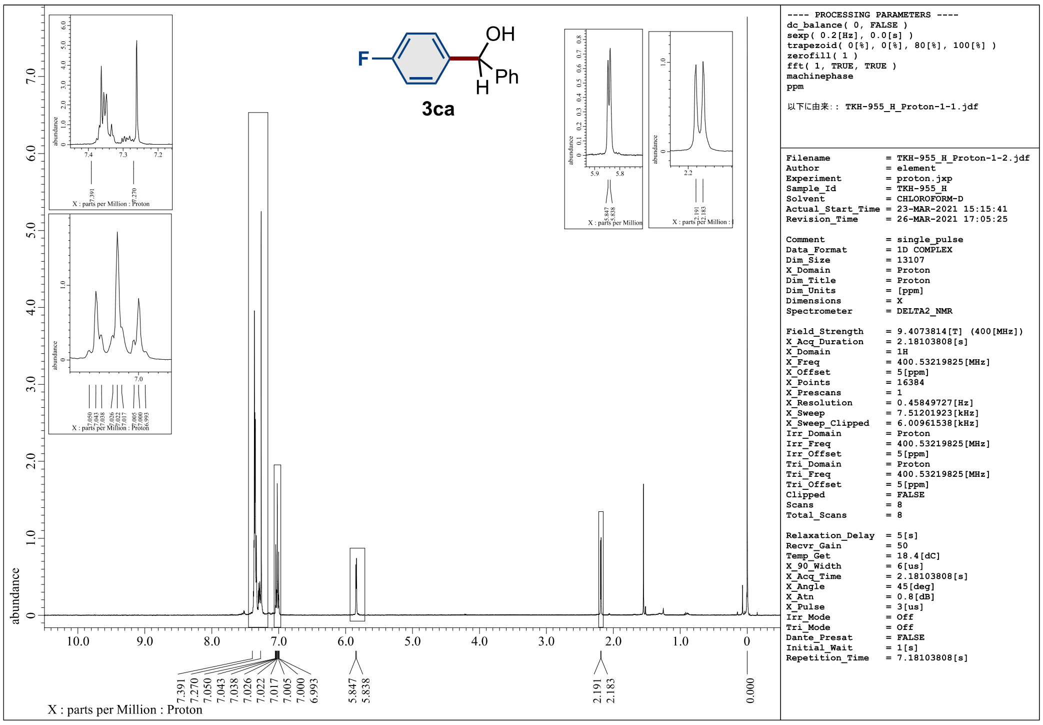

Supplementary Figure 38. ^{1}H NMR spectrum of **3ca**.

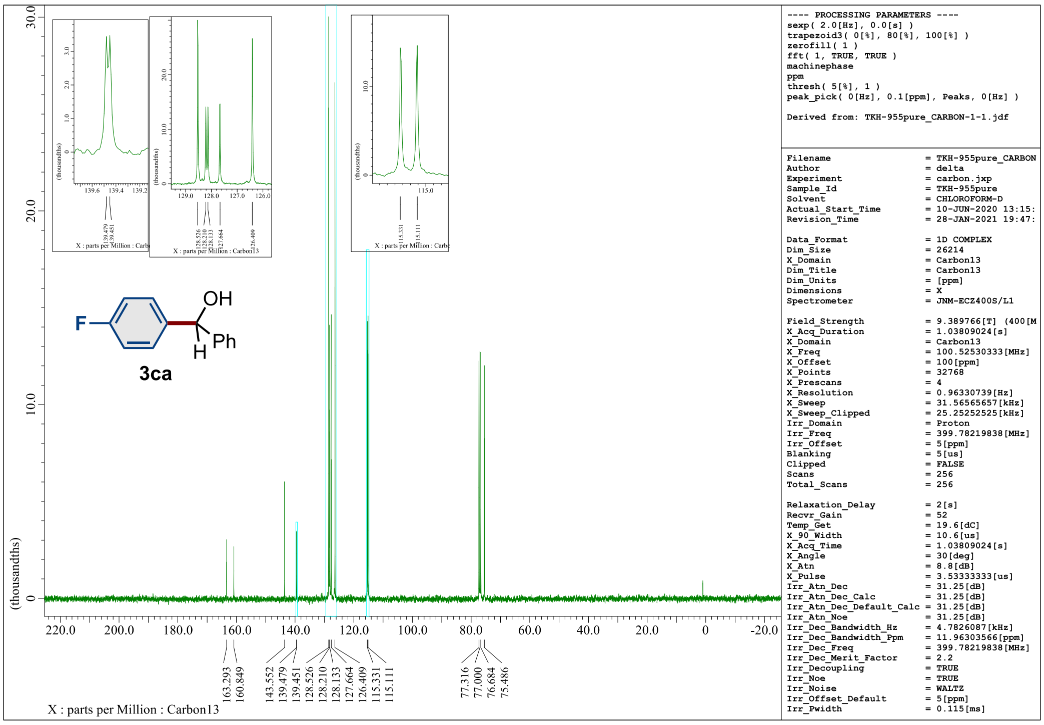

Supplementary Figure 39. ^{13}C NMR spectrum of **3ca**.

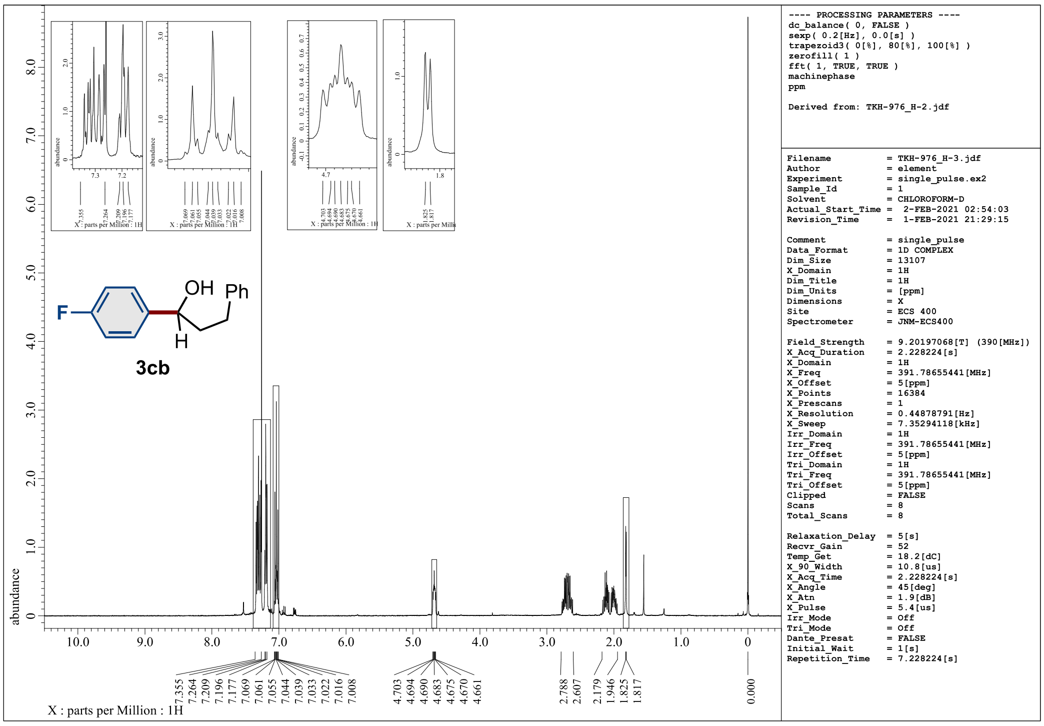

Supplementary Figure 40. ^{1}H NMR spectrum of **3cb**.

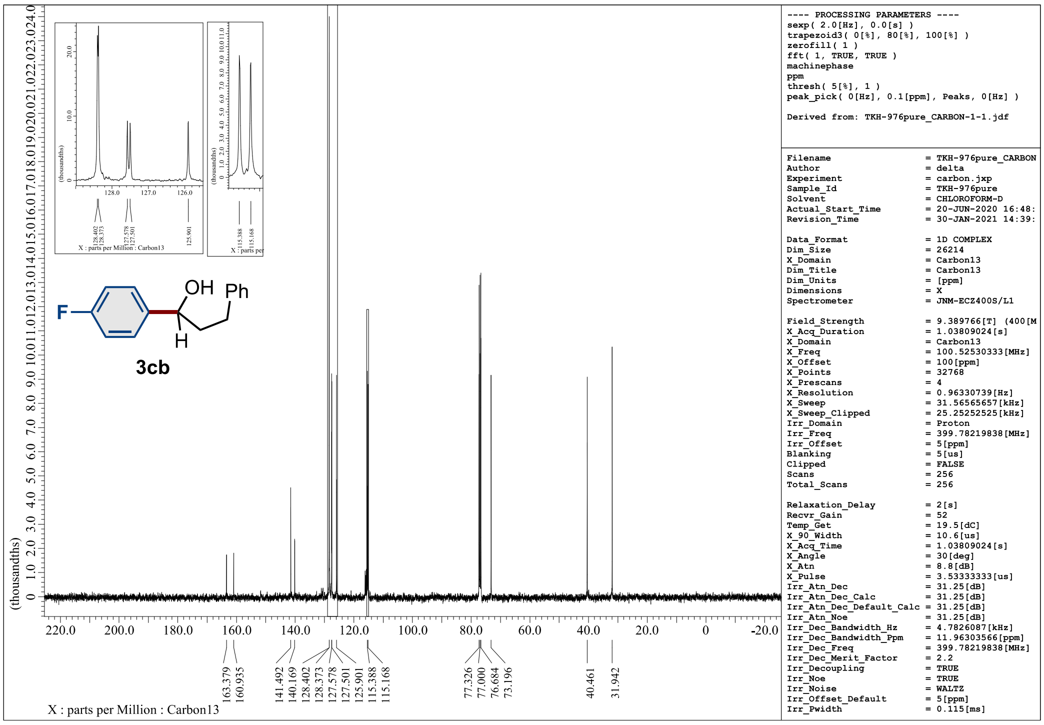

Supplementary Figure 41. ^{13}C NMR spectrum of **3cb**.

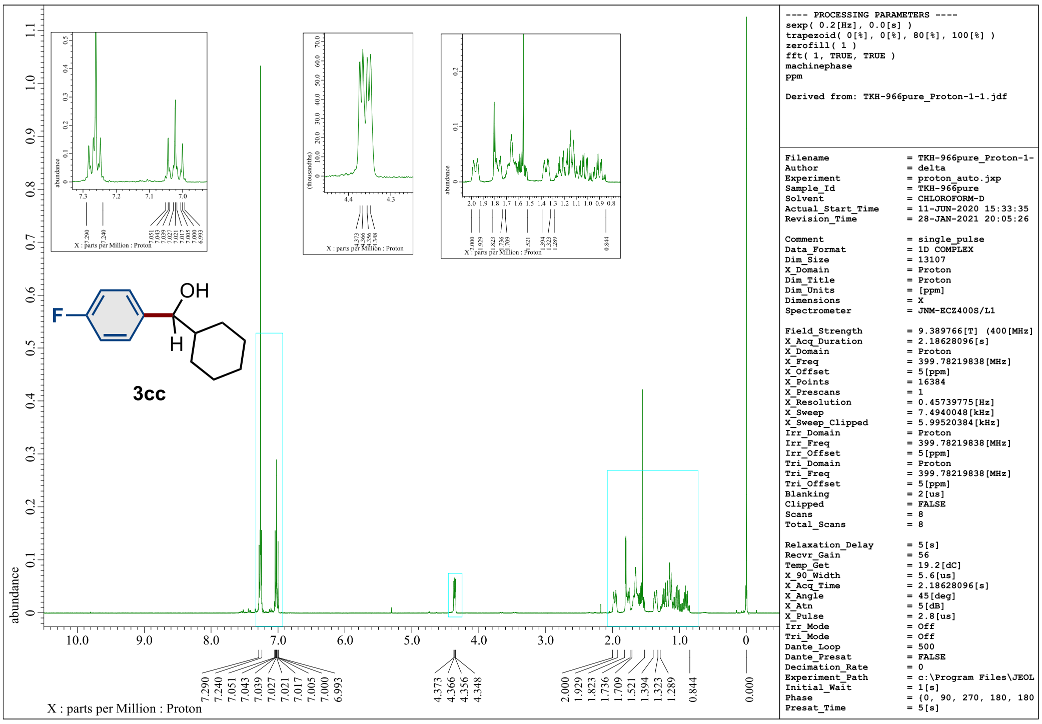

Supplementary Figure 42. ^{1}H NMR spectrum of **3cc**.

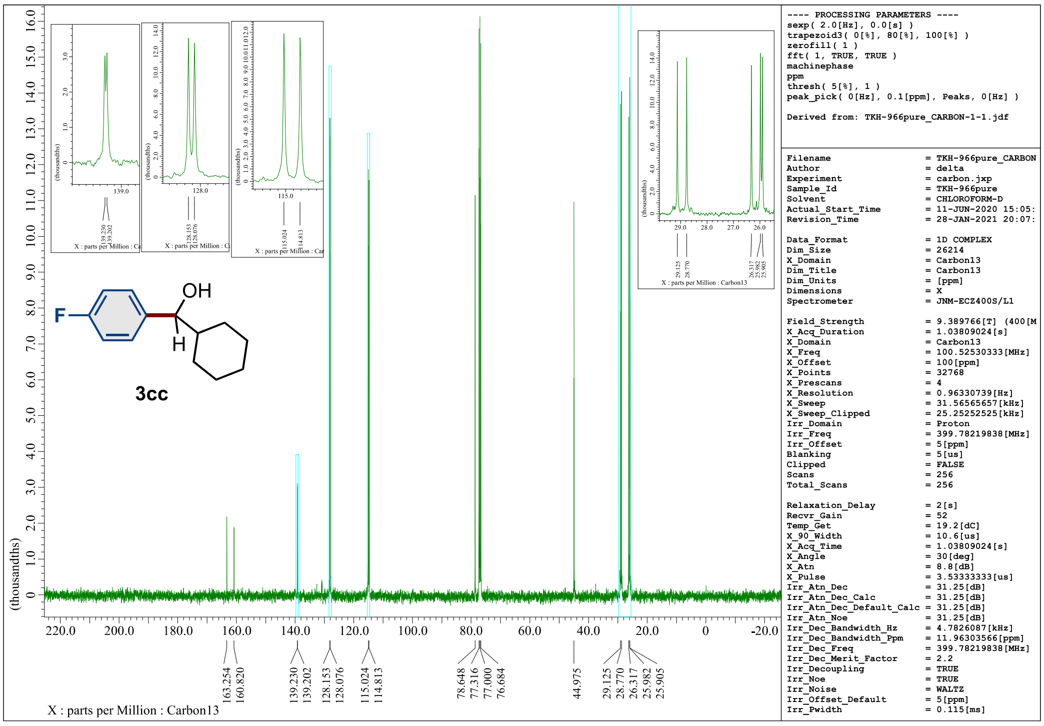

Supplementary Figure 43. ^{13}C NMR spectrum of **3cc**.

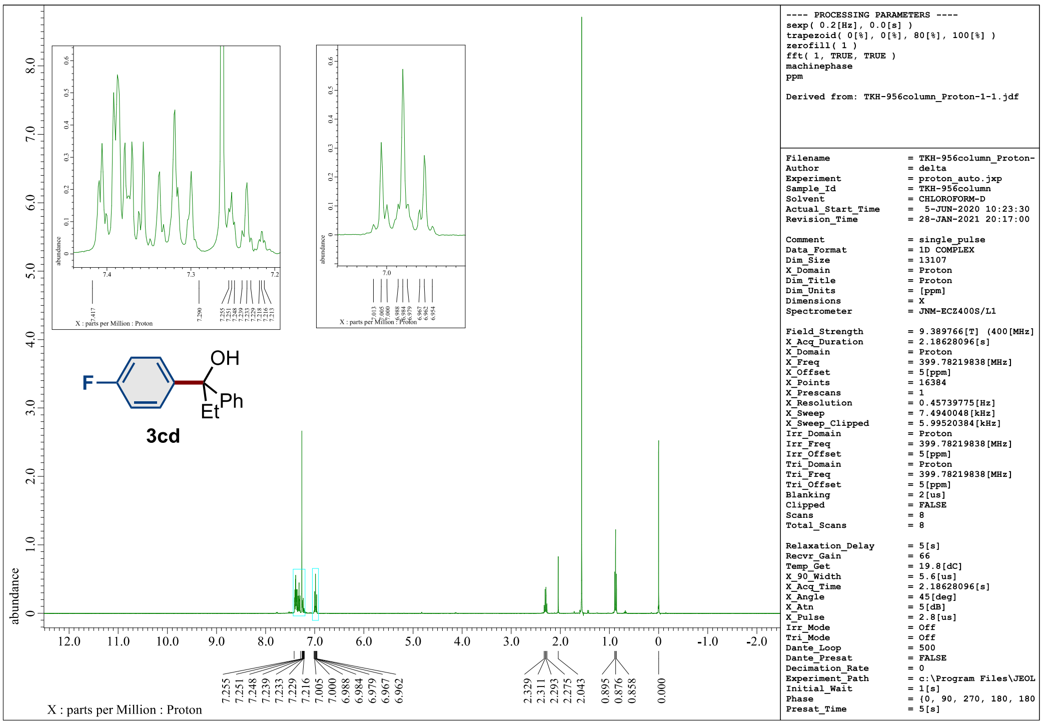

Supplementary Figure 44. ^{1}H NMR spectrum of **3cd**.

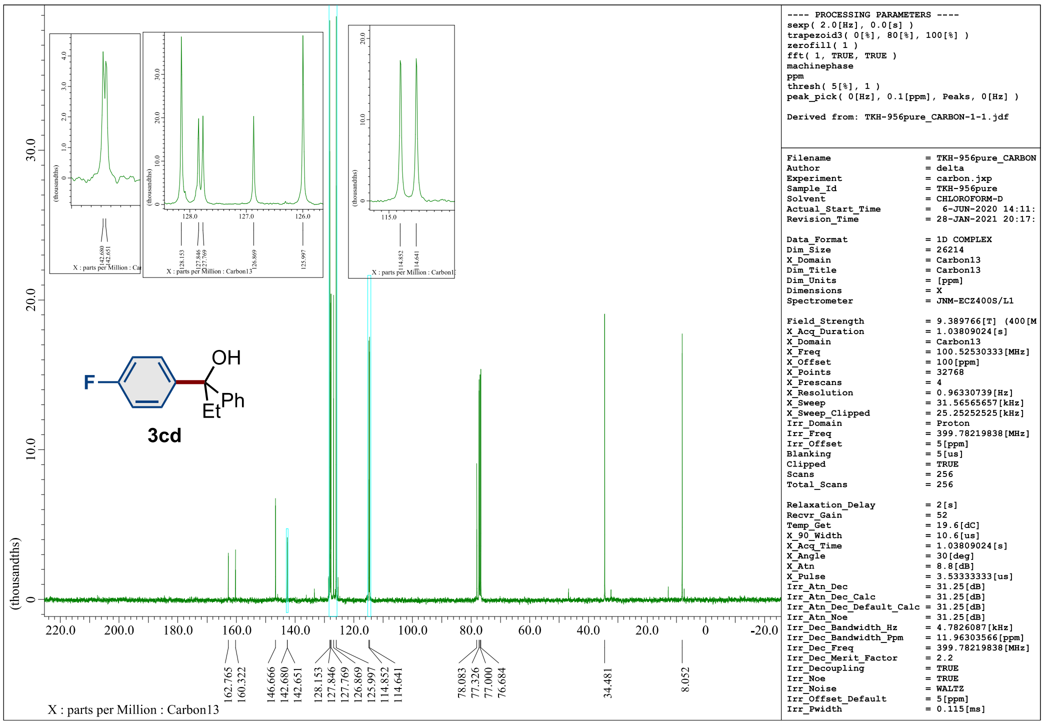

Supplementary Figure 45. ^{13}C NMR spectrum of **3cd**.

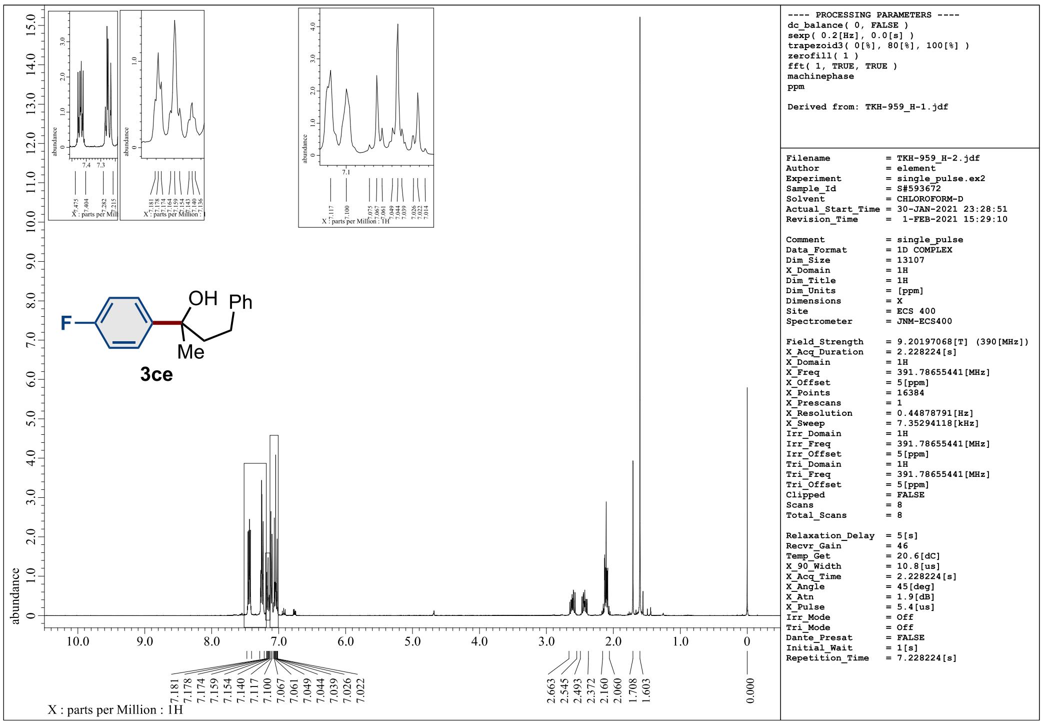

Supplementary Figure 46. ^{1}H NMR spectrum of **3ce**.

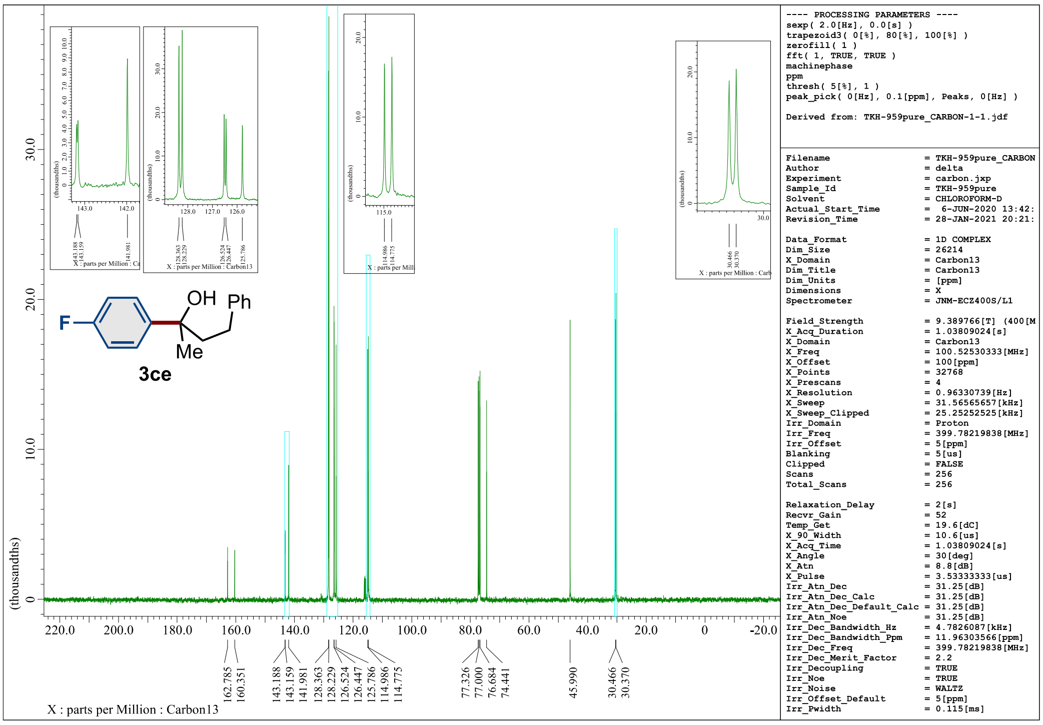

Supplementary Figure 47. ^{13}C NMR spectrum of **3ce**.

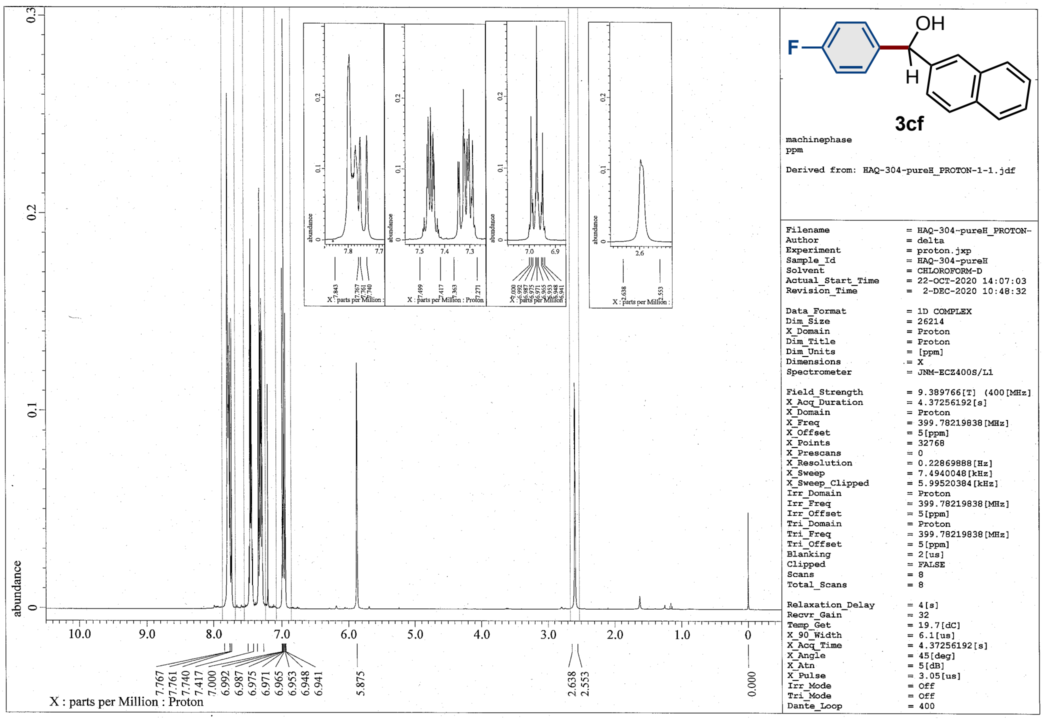

Supplementary Figure 48. ^{1}H NMR spectrum of **3cf**.

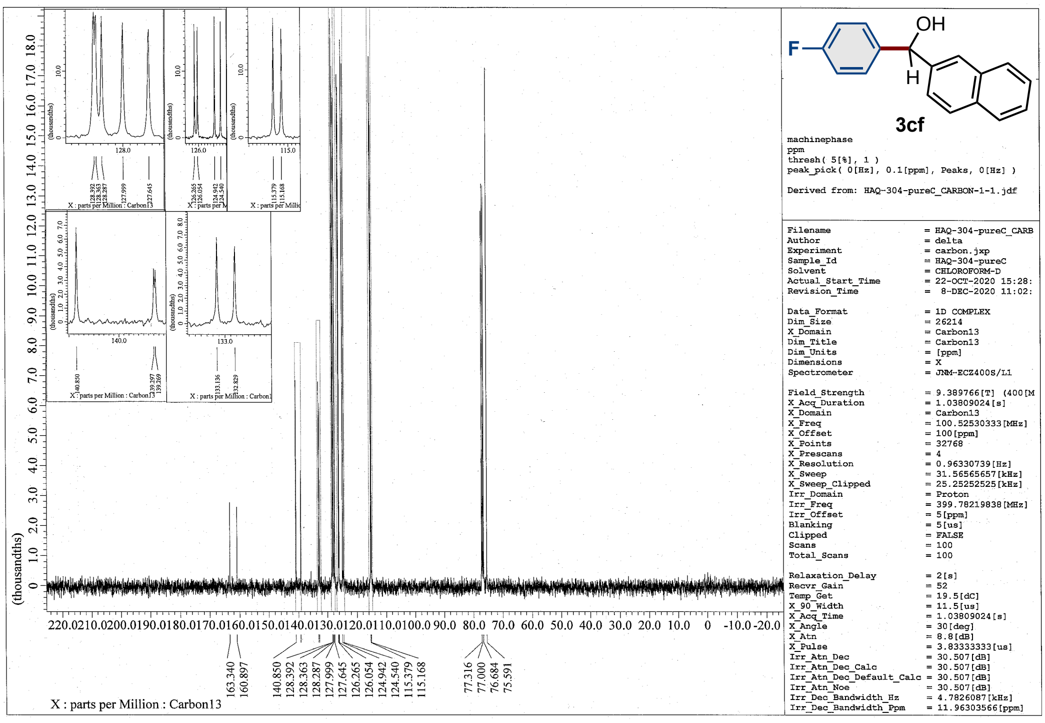

Supplementary Figure 49. ^{13}C NMR spectrum of **3cf**.

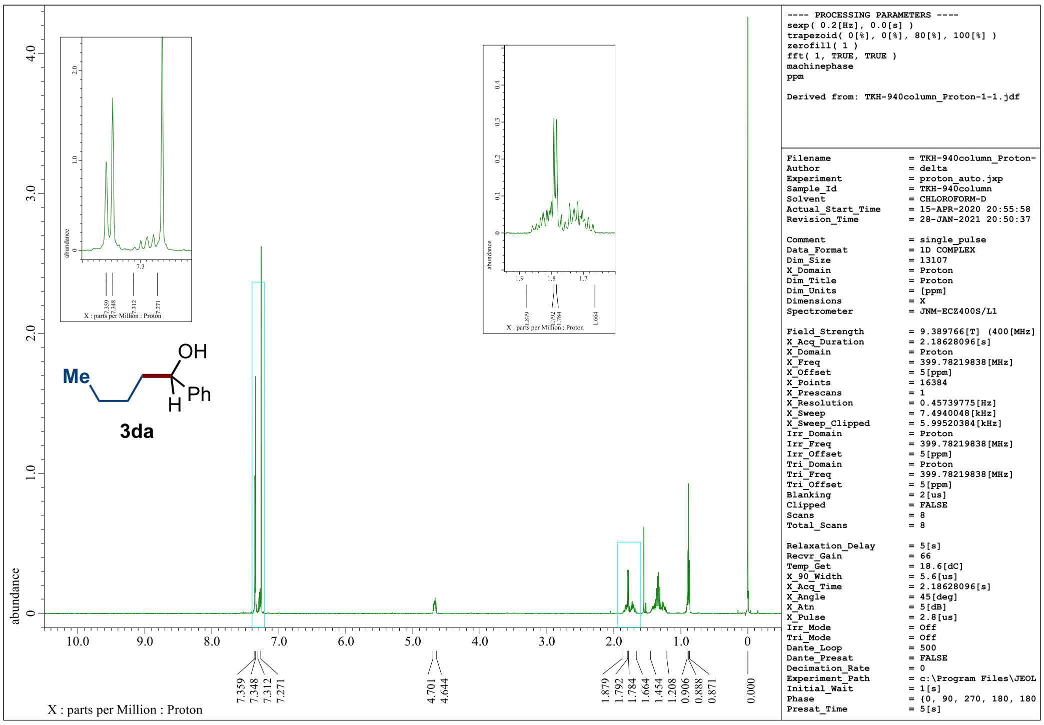

Supplementary Figure 50. ^{1}H NMR spectrum of **3da**.

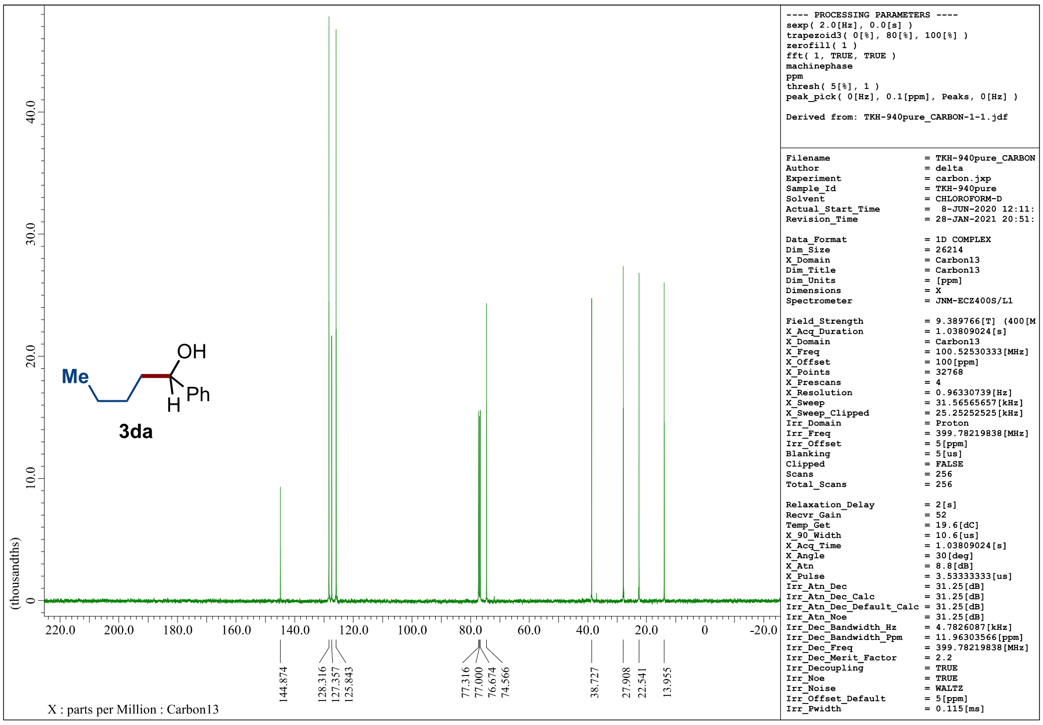

Supplementary Figure 51. ^{13}C NMR spectrum of **3da**.

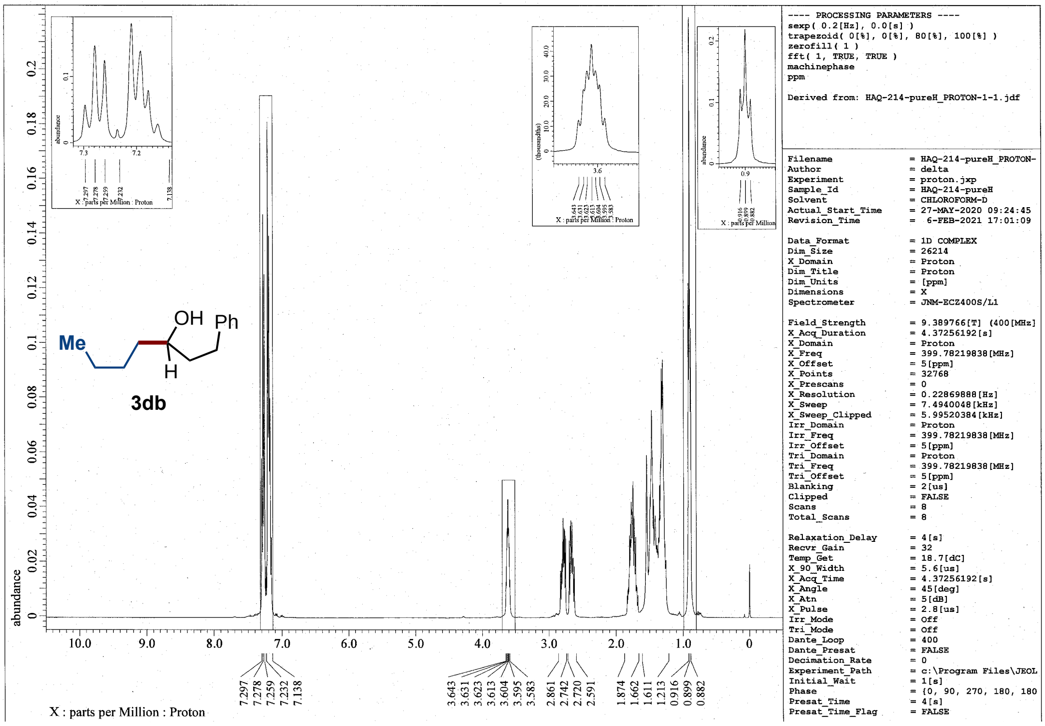

Supplementary Figure 52. ^{1}H NMR spectrum of **3db**.

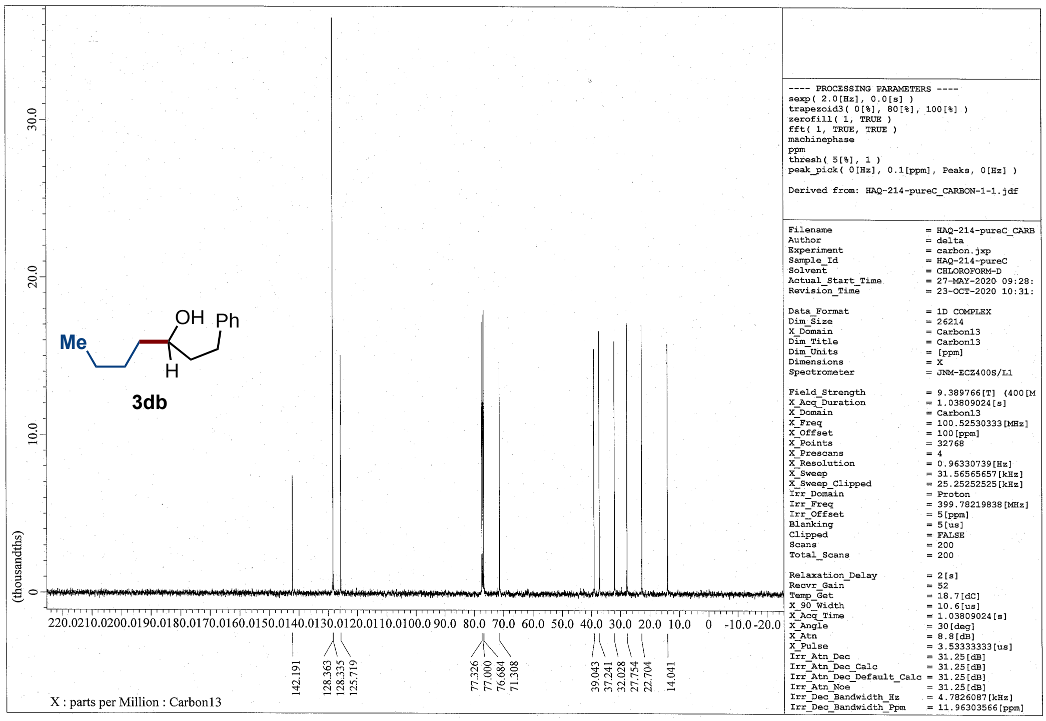

Supplementary Figure 53. ^{13}C NMR spectrum of **3db**.

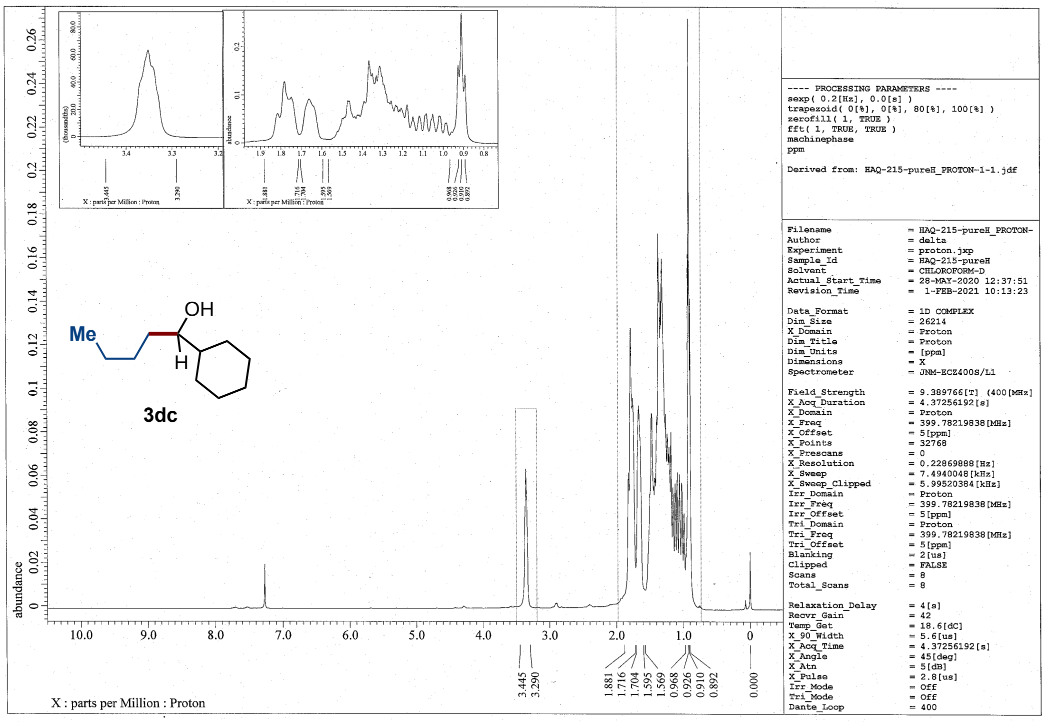

Supplementary Figure 54. ^{1}H NMR spectrum of **3dc**.

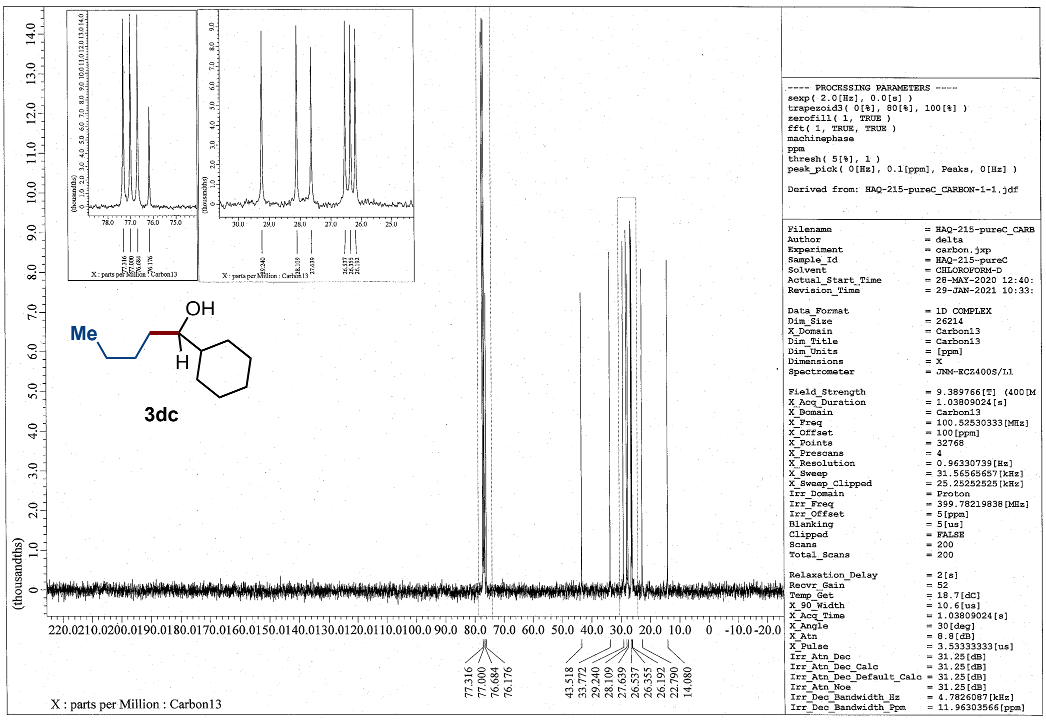

Supplementary Figure 55. ^{13}C NMR spectrum of **3dc**.

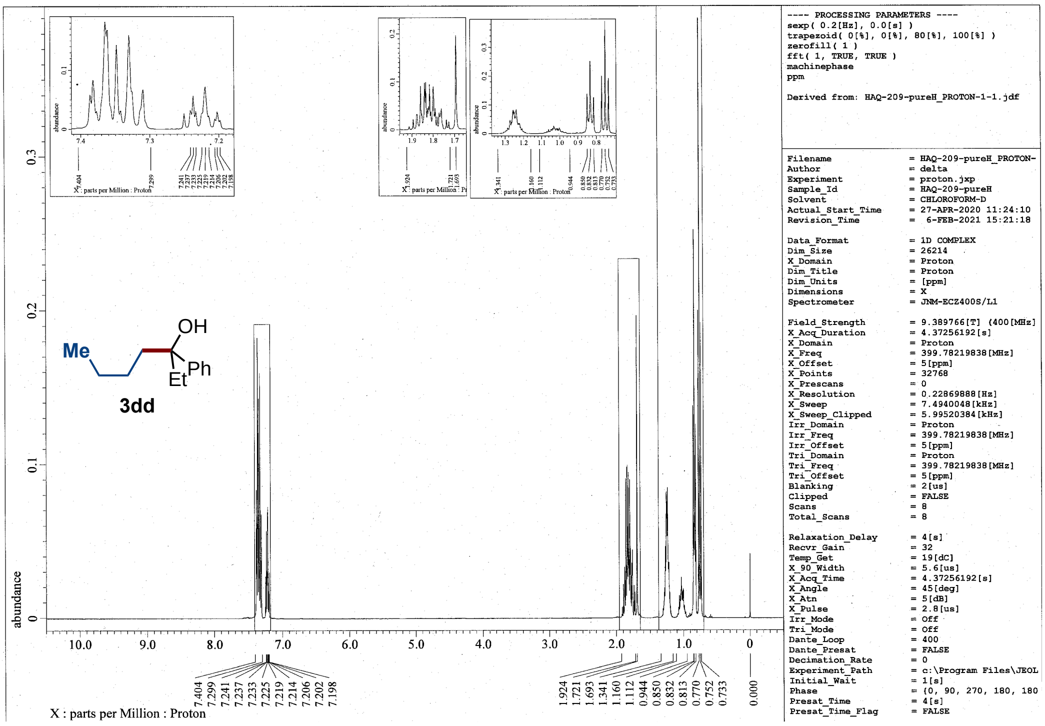

Supplementary Figure 56. ^{1}H NMR spectrum of **3dd**.

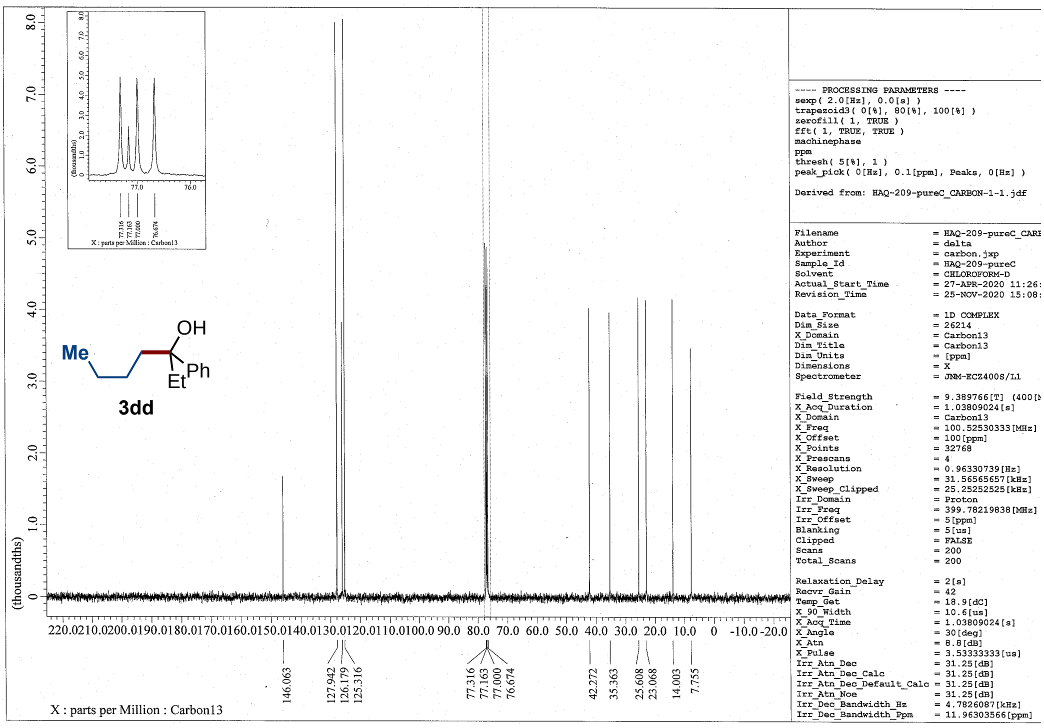

Supplementary Figure 57. ^{13}C NMR spectrum of **3dd**.

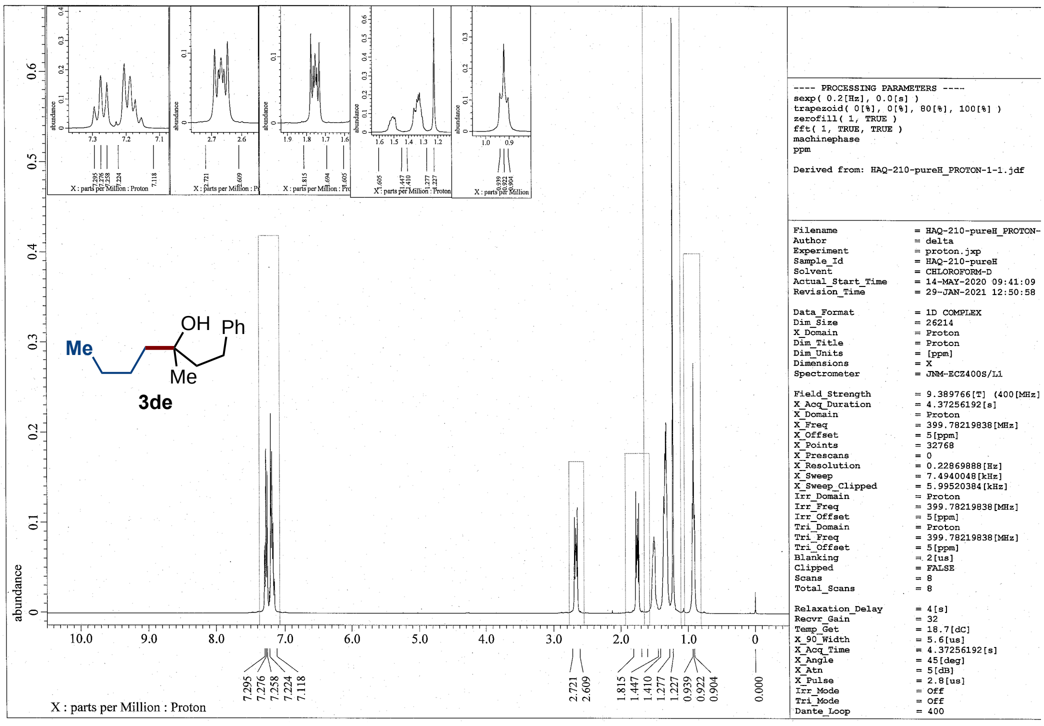

Supplementary Figure 58. ^{1}H NMR spectrum of **3de**.

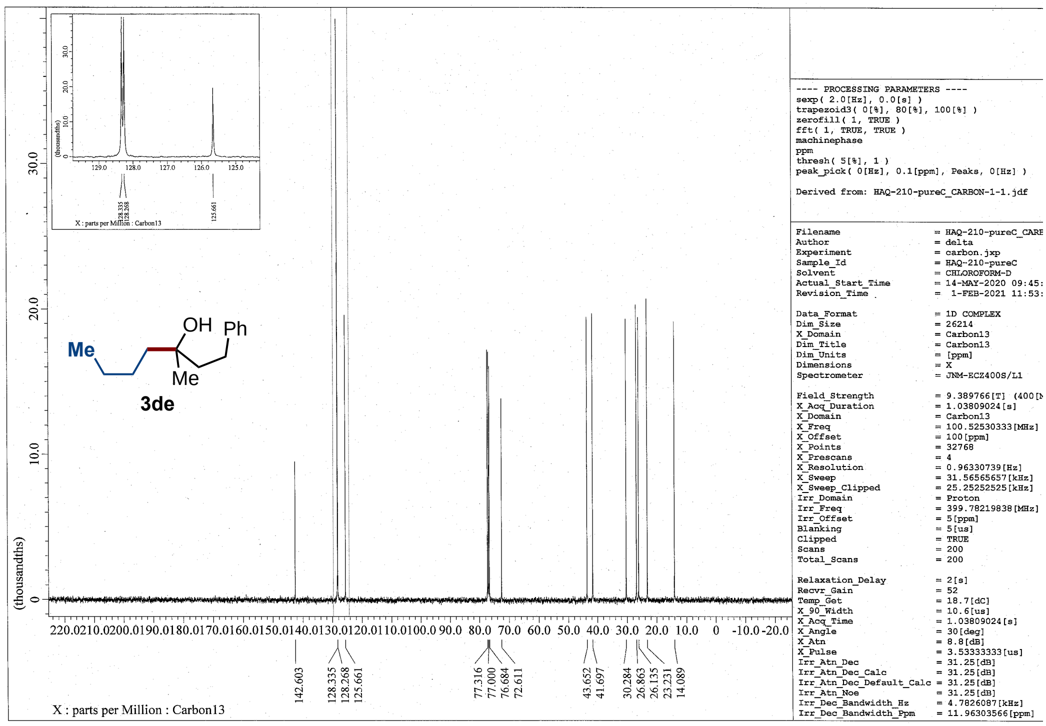

Supplementary Figure 59. ^{13}C NMR spectrum of **3de**.

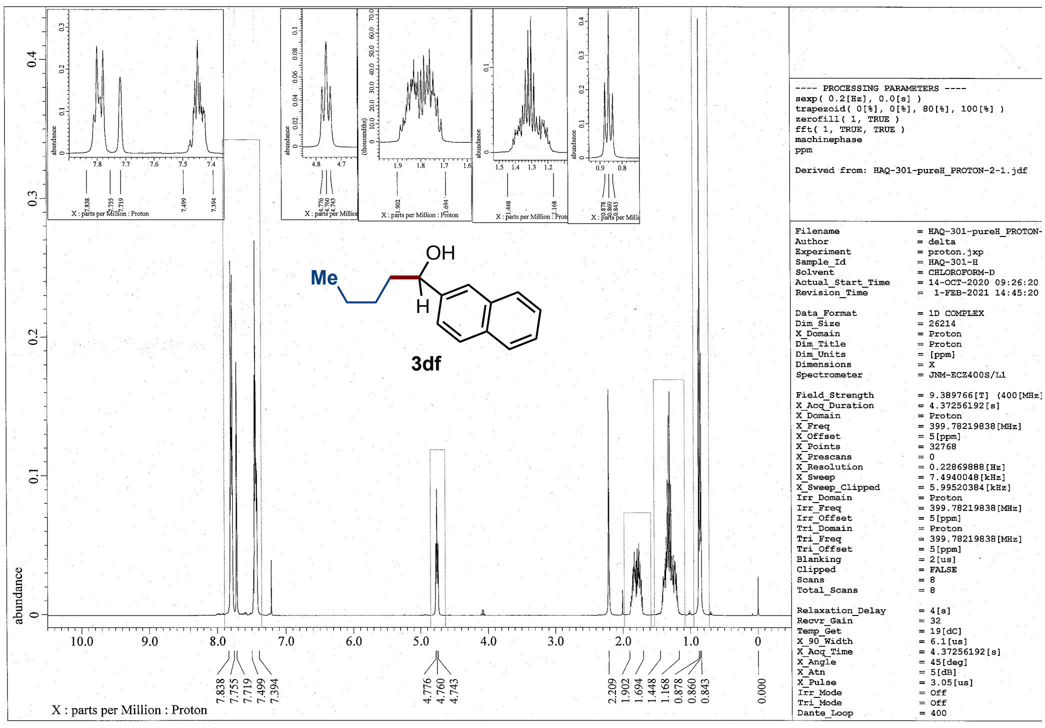

Supplementary Figure 60. ^{1}H NMR spectrum of **3df**.

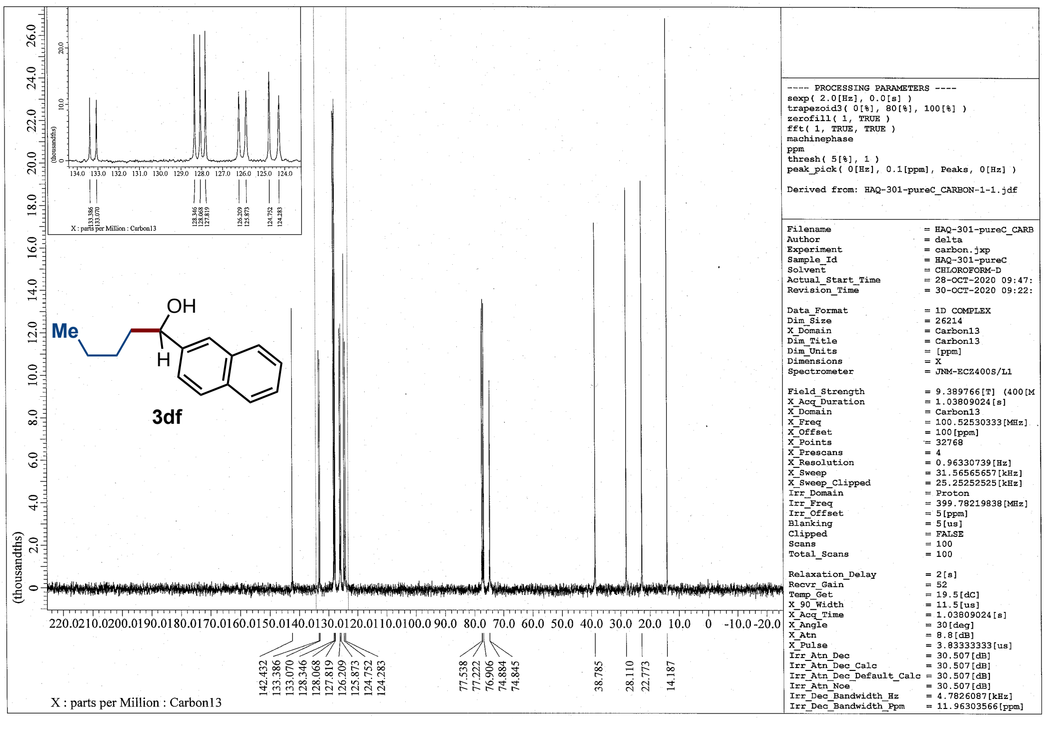

Supplementary Figure 61. ^{13}C NMR spectrum of **3df**.

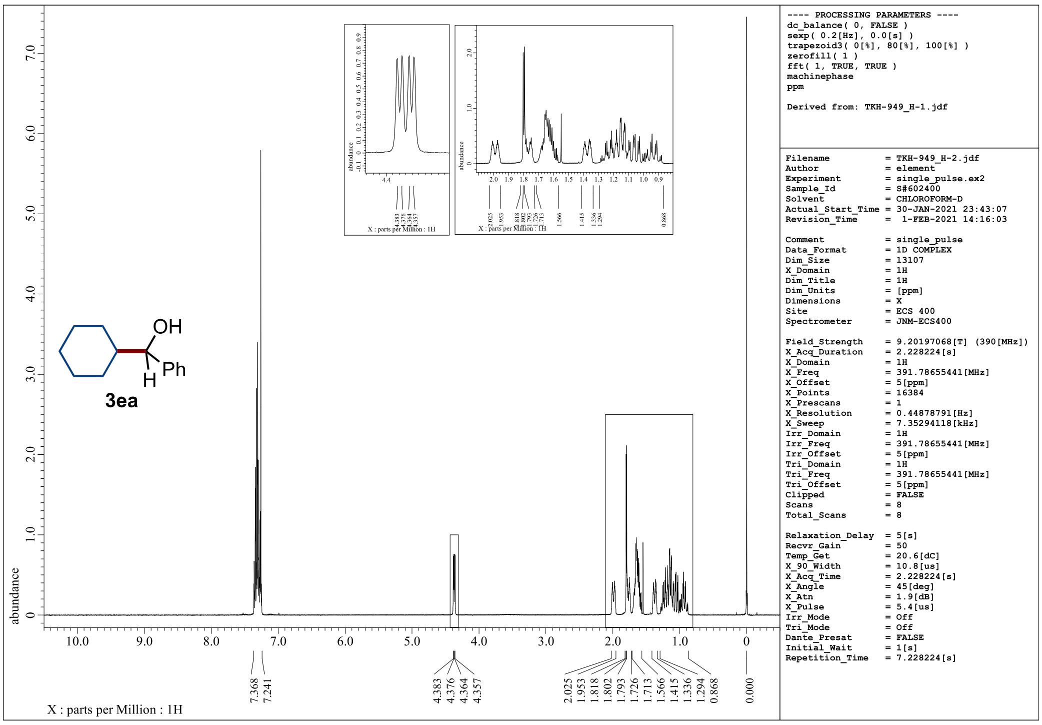

Supplementary Figure 62. ^{1}H NMR spectrum of **3ea**.

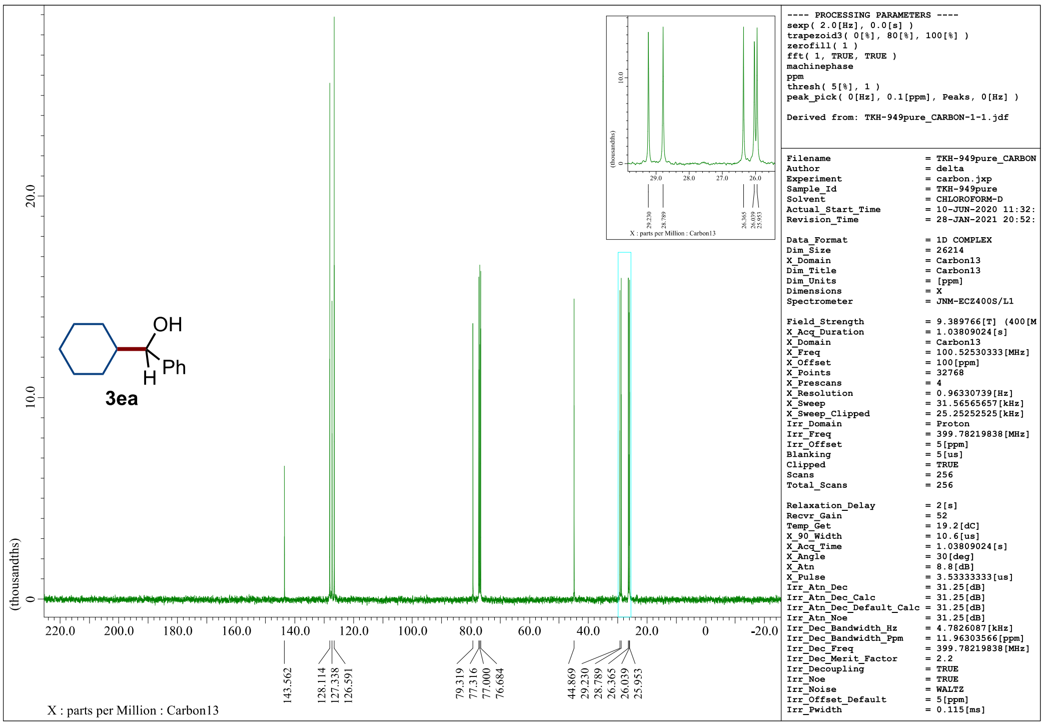

Supplementary Figure 63. ^{13}C NMR spectrum of **3ea**.

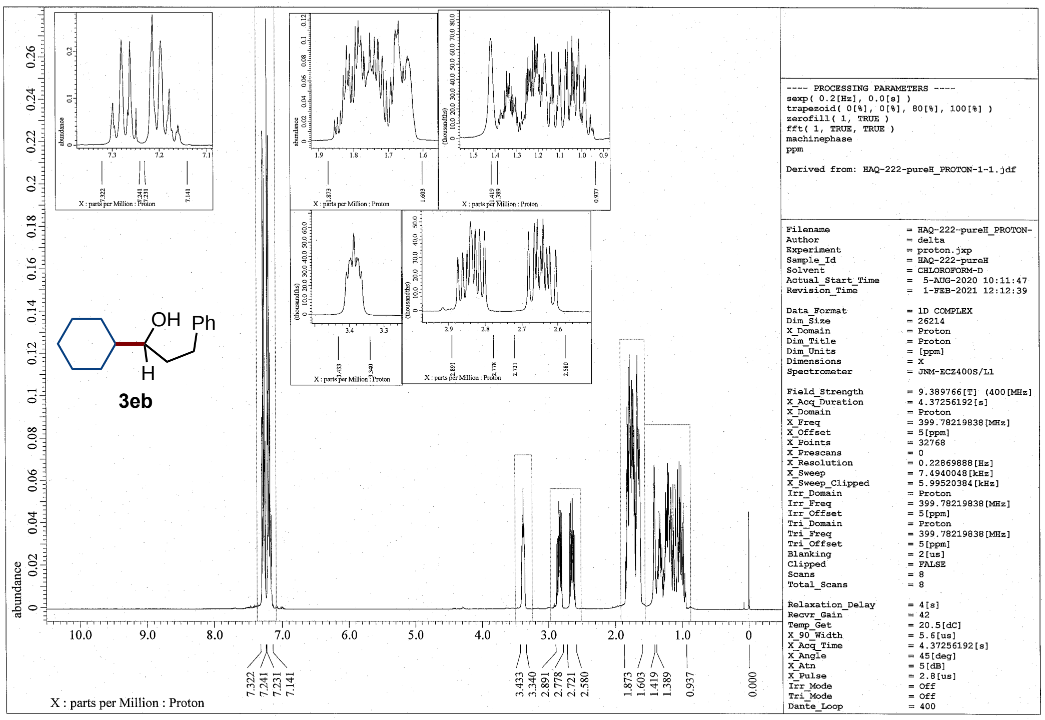

Supplementary Figure 64. ^{1}H NMR spectrum of **3eb**.

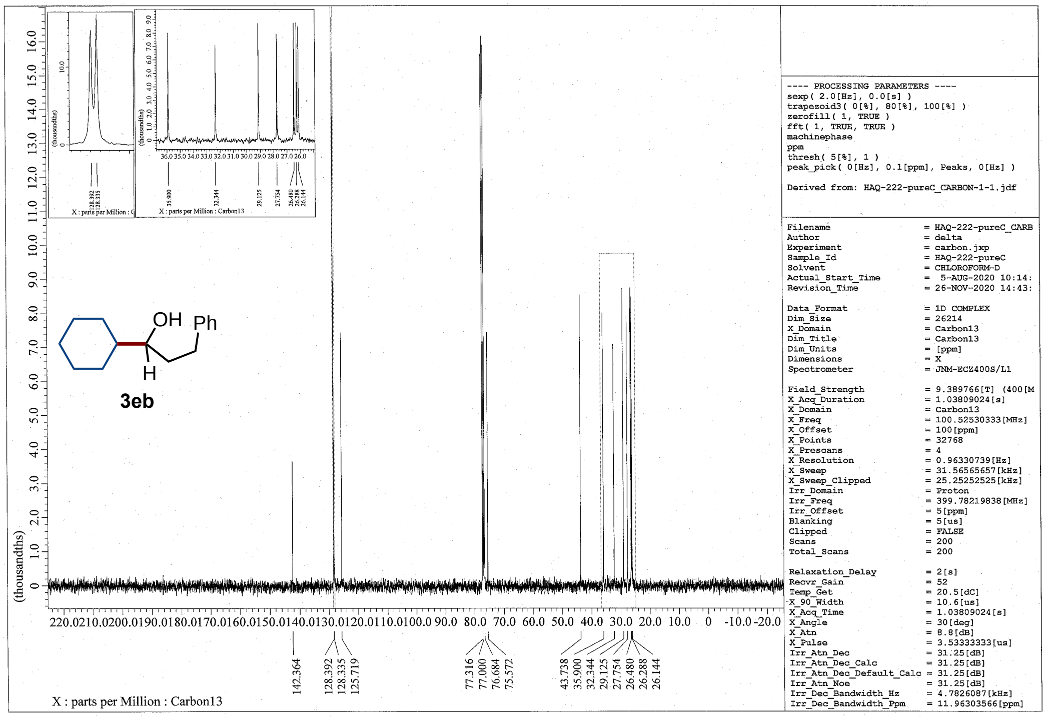

Supplementary Figure 65. ^{13}C NMR spectrum of **3eb**.

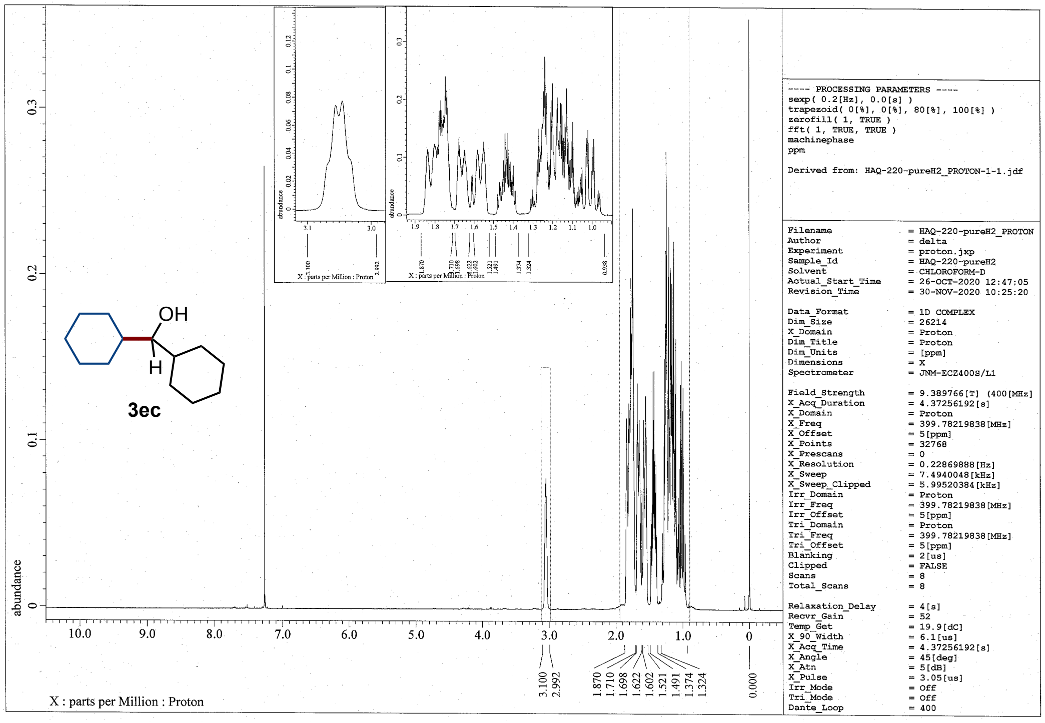

Supplementary Figure 66. ^{1}H NMR spectrum of **3ec**.

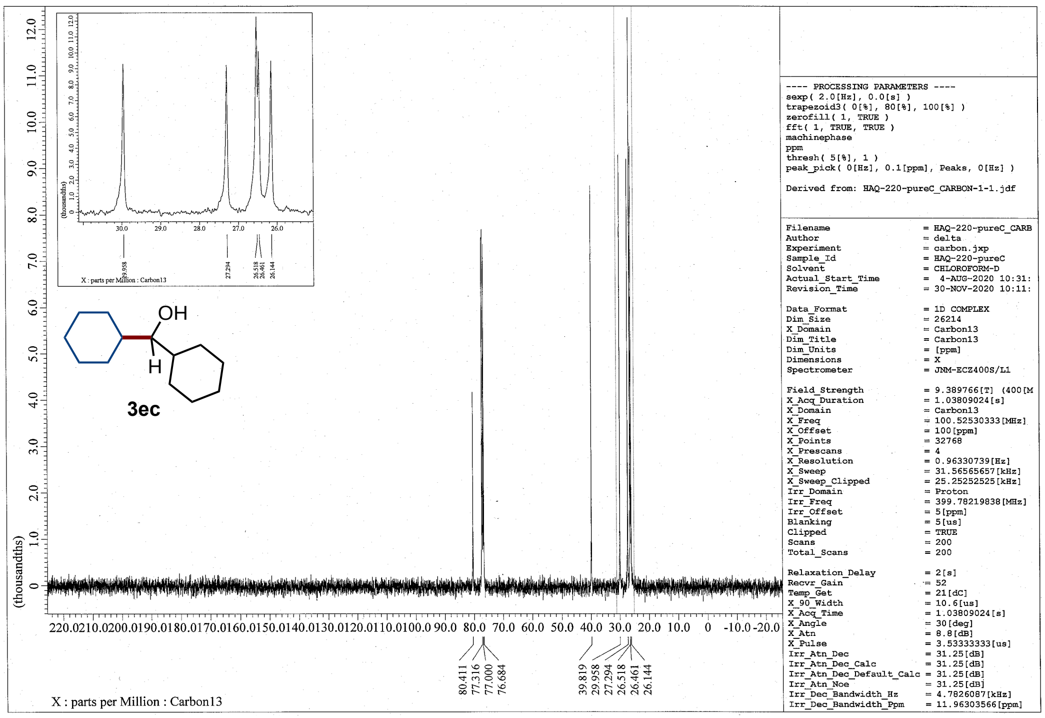

Supplementary Figure 67. ^{13}C NMR spectrum of **3ec**.

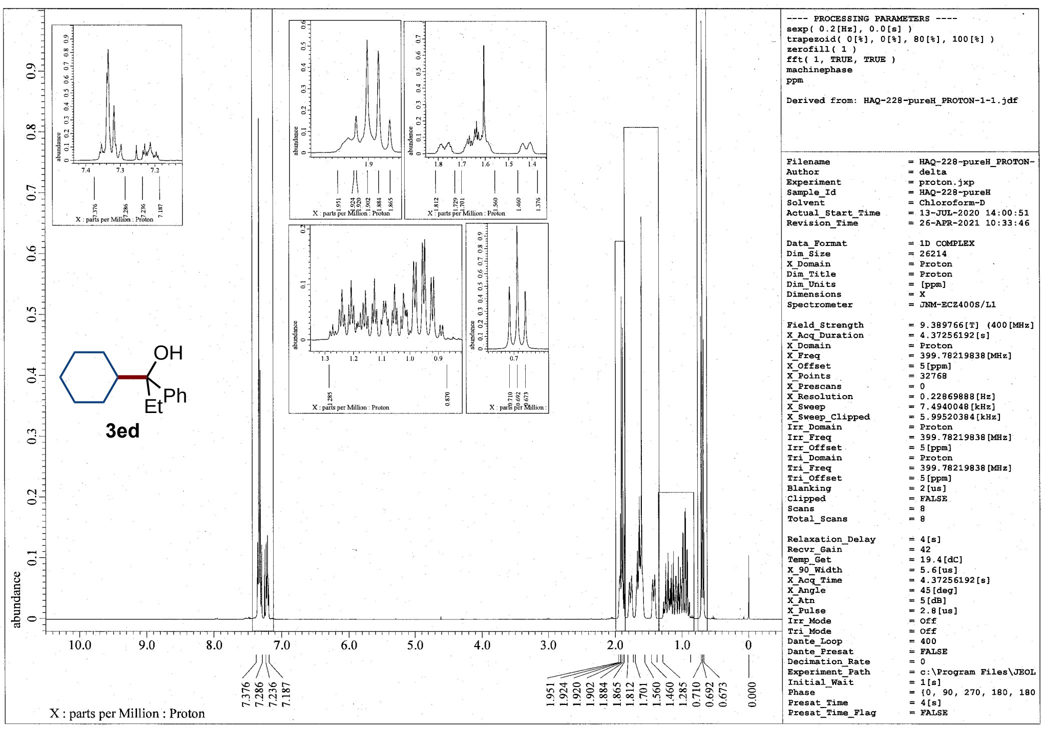

Supplementary Figure 68. ^{1}H NMR spectrum of **3ed**.

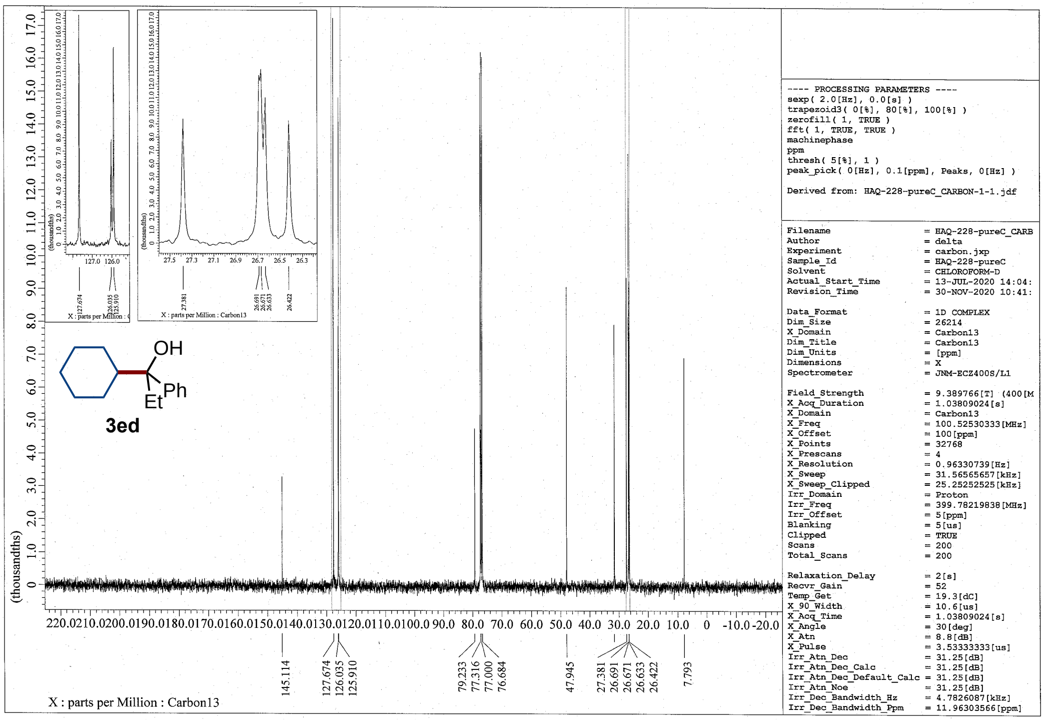

Supplementary Figure 69. ^{13}C NMR spectrum of **3ed**.

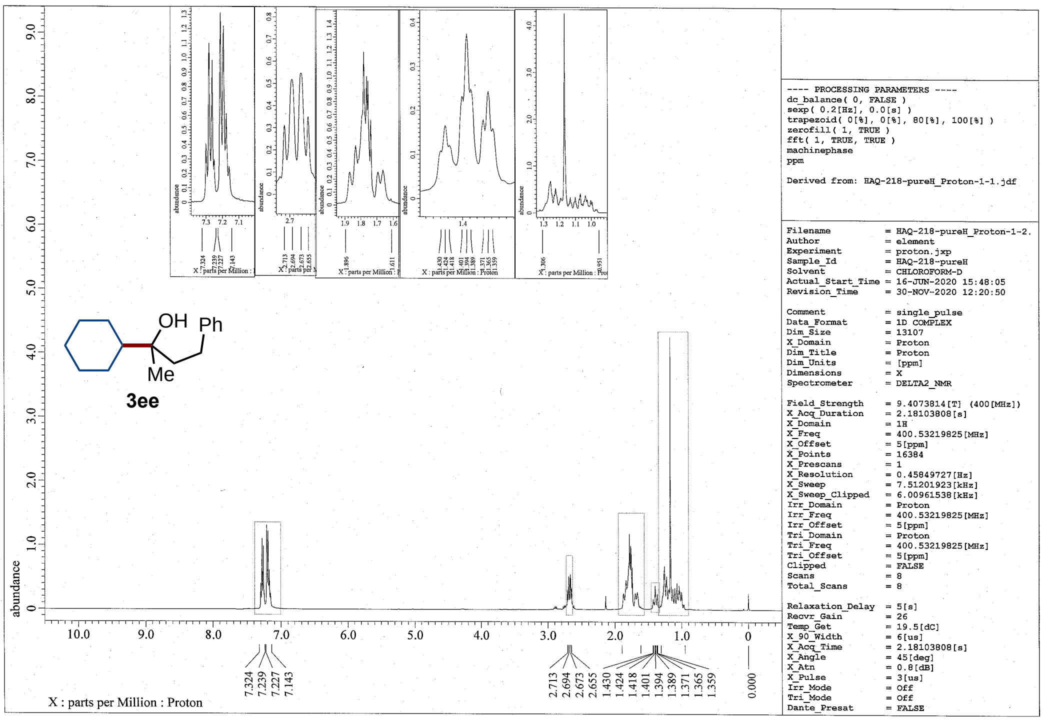

Supplementary Figure 70. ^{1}H NMR spectrum of **3ee**.

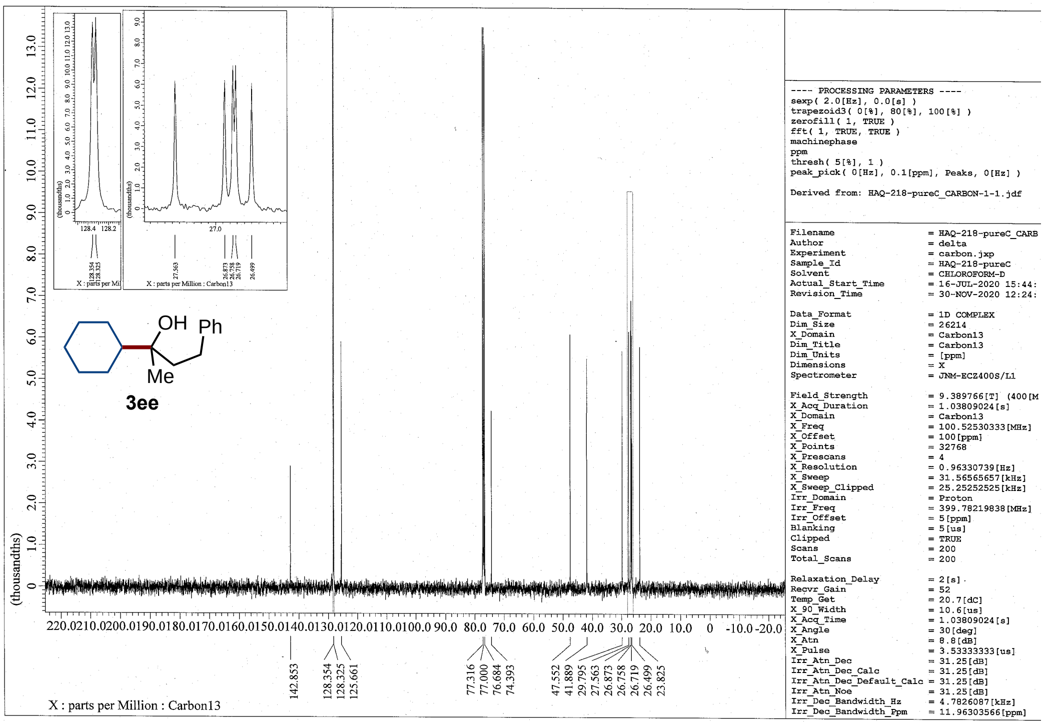

Supplementary Figure 71. ^{13}C NMR spectrum of **3ee**.

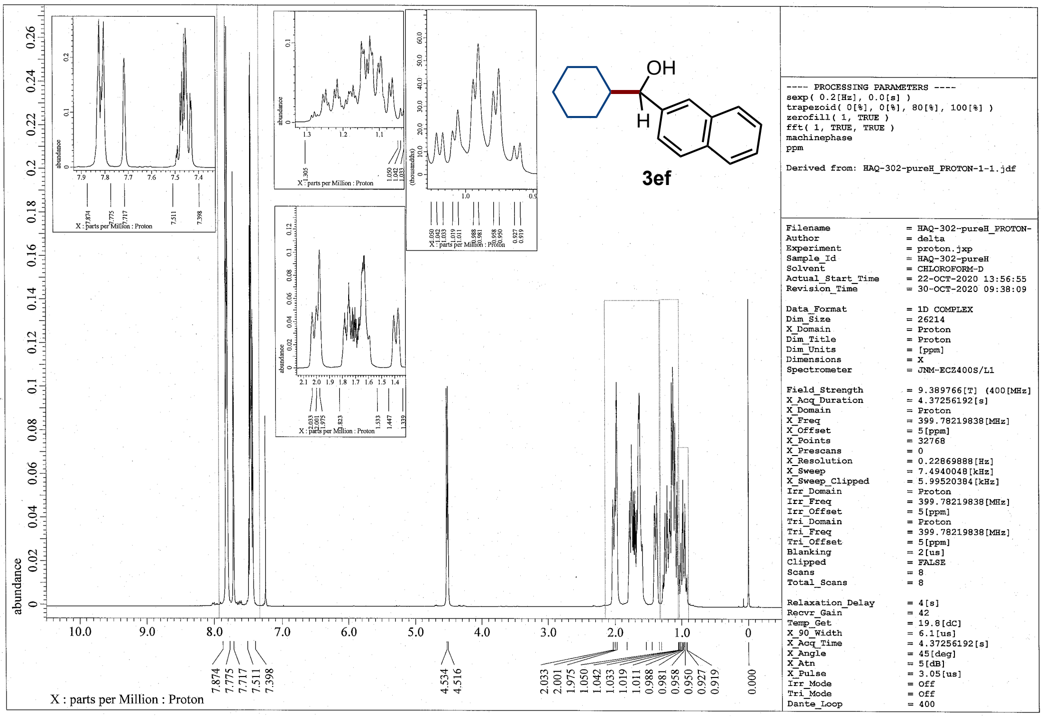

Supplementary Figure 72. ^{1}H NMR spectrum of **3ef**.

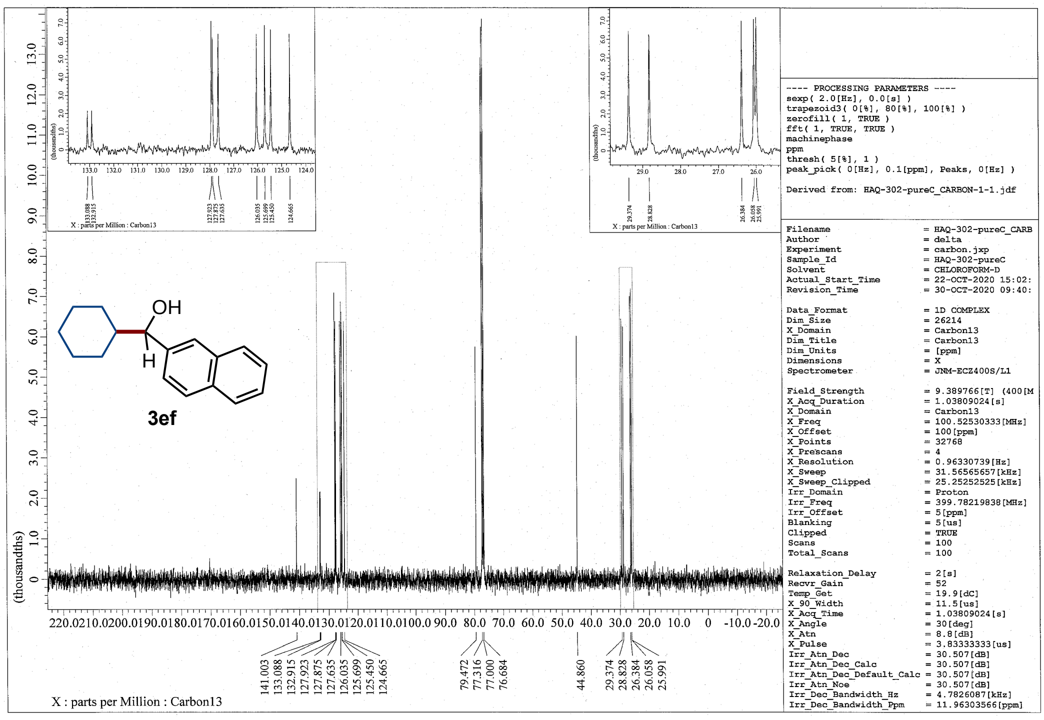

Supplementary Figure 73. ^{13}C NMR spectrum of **3ef**.

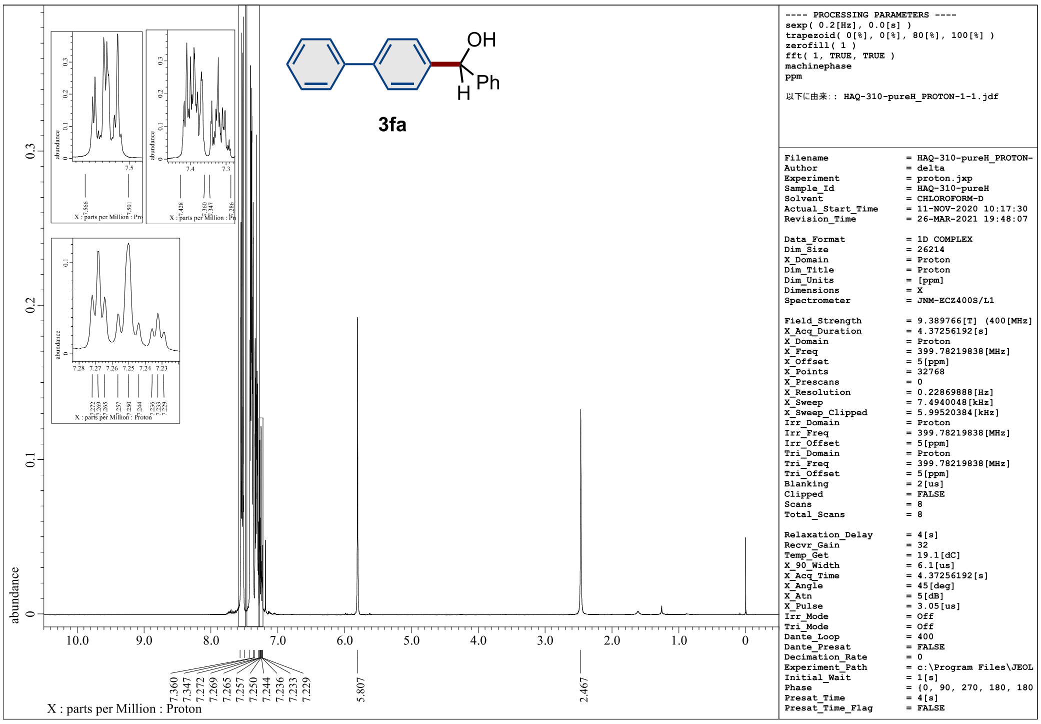

Supplementary Figure 74. ^{1}H NMR spectrum of **3fa**.

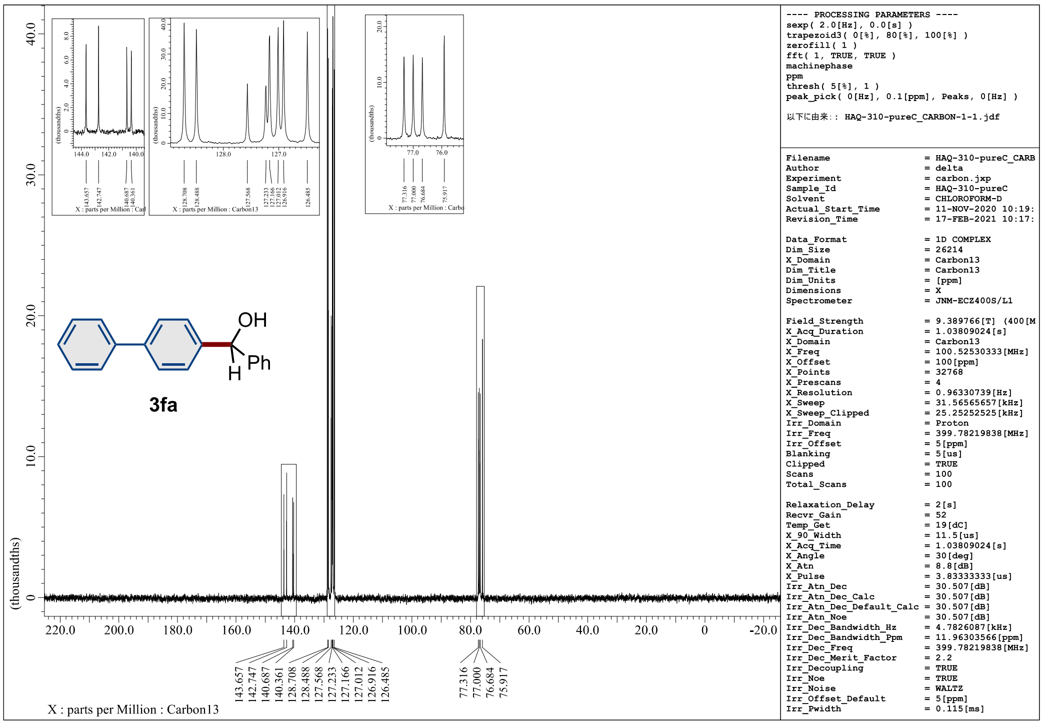

Supplementary Figure 75. ^{13}C NMR spectrum of **3fa**.

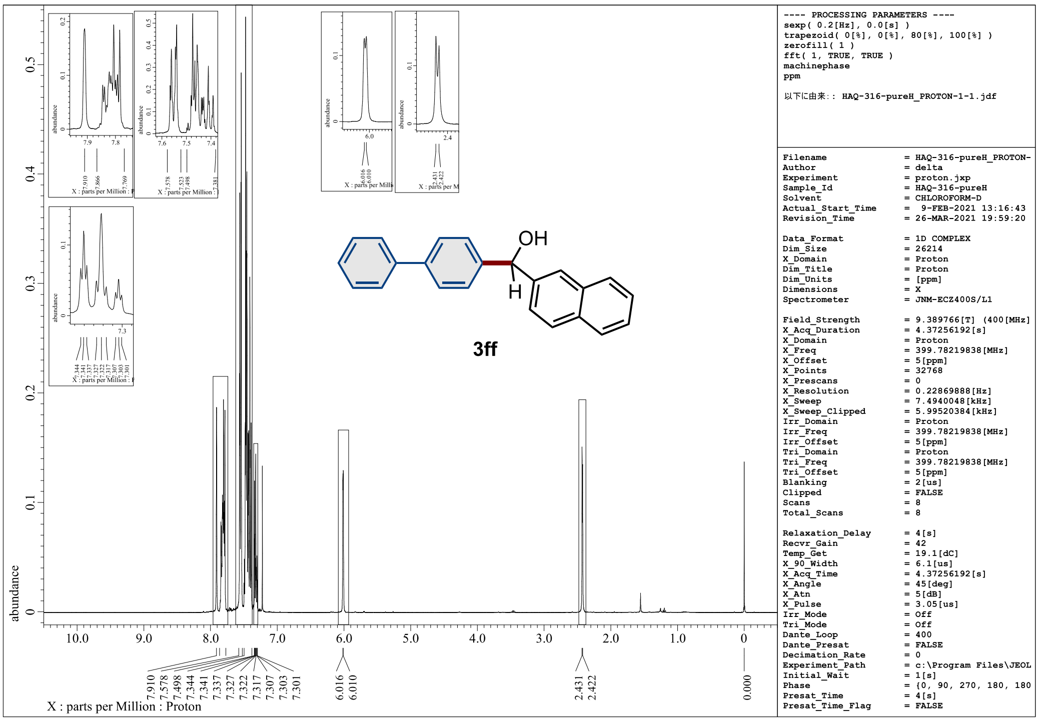

Supplementary Figure 76. ^{1}H NMR spectrum of **3ff**.

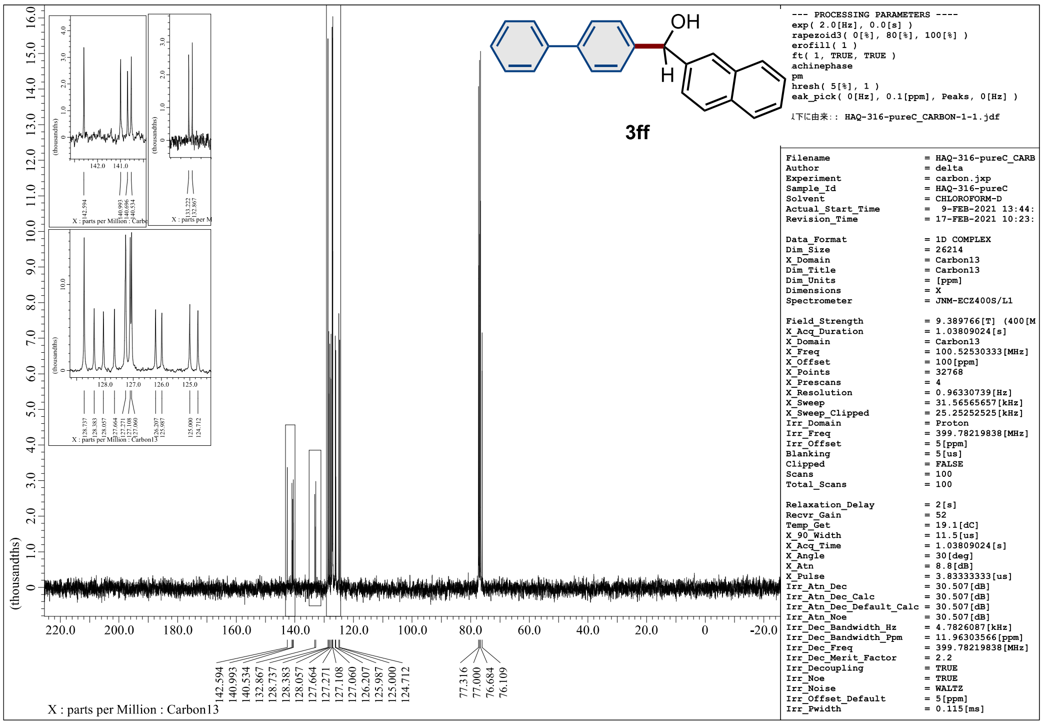

Supplementary Figure 77. ^{13}C NMR spectrum of **3ff**.

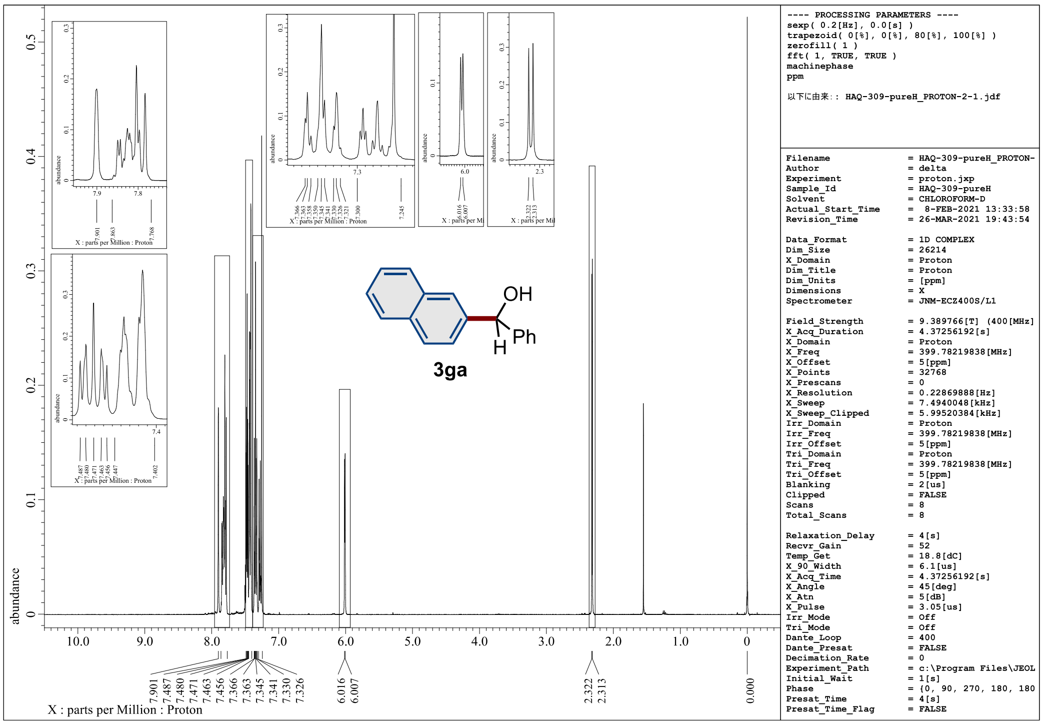

Supplementary Figure 78. ^{1}H NMR spectrum of **3ga**.

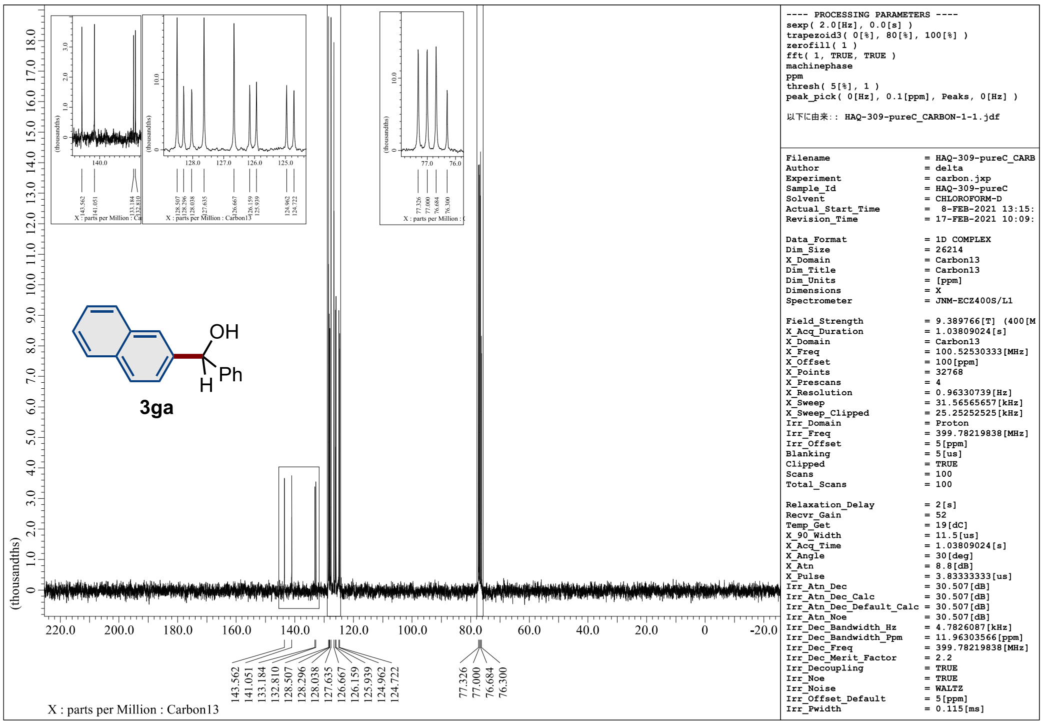

Supplementary Figure 79. ^{13}C NMR spectrum of **3ga**.

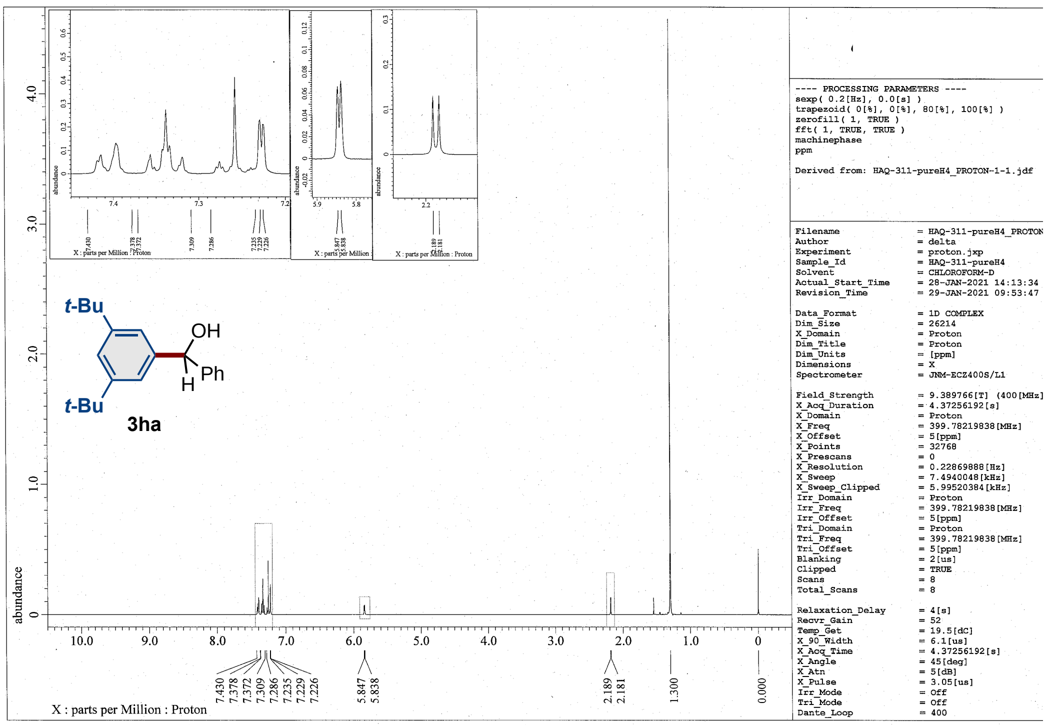

Supplementary Figure 80. ^{1}H NMR spectrum of **3ha**.

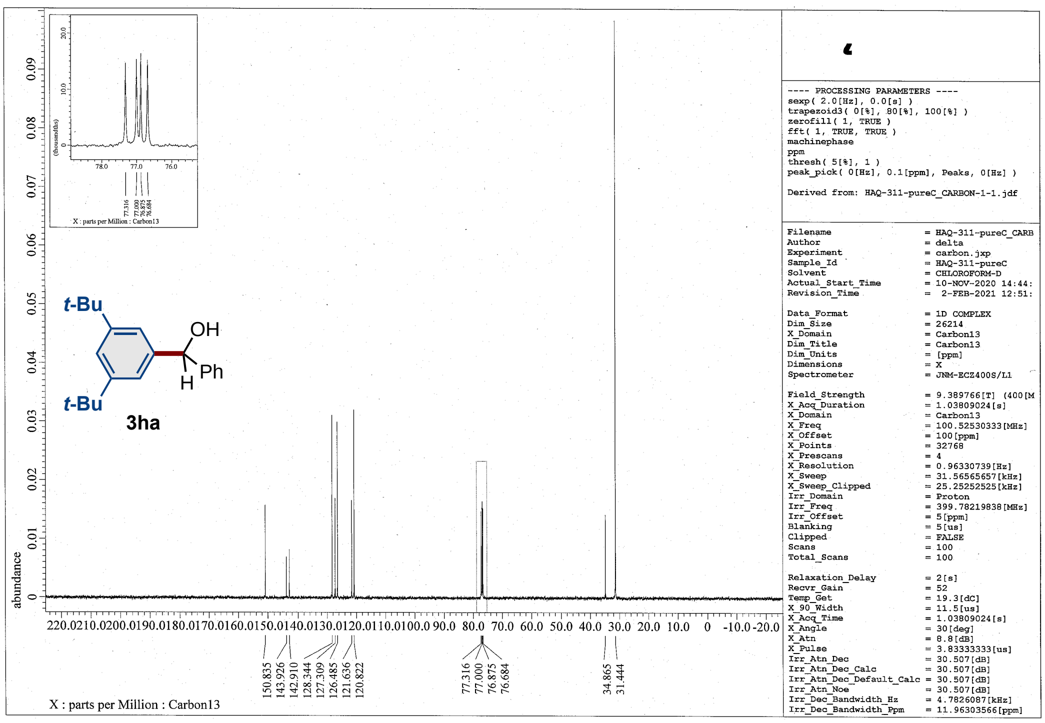

Supplementary Figure 81. ^{13}C NMR spectrum of **3ha**.

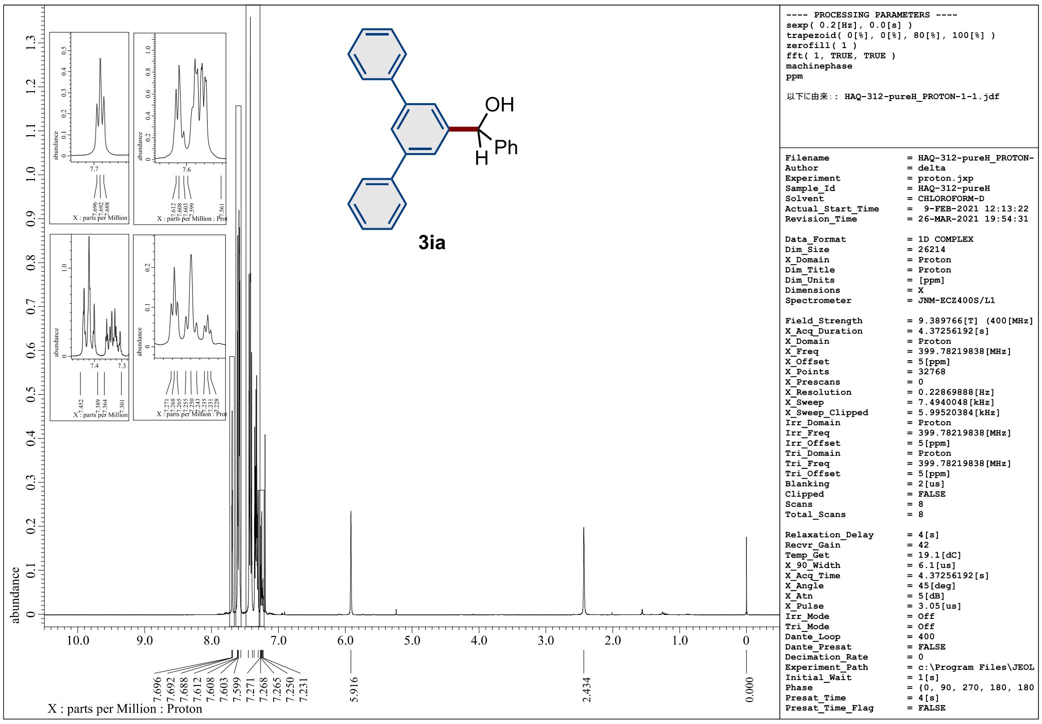

Supplementary Figure 82. ^{1}H NMR spectrum of **3ia**.

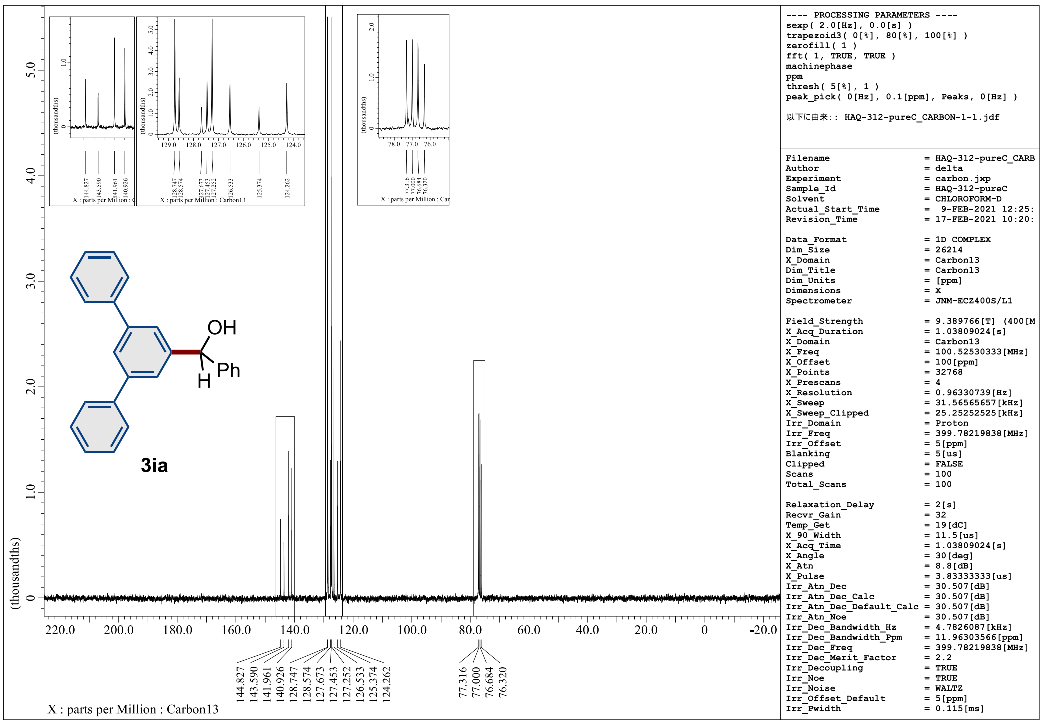

Supplementary Figure 83. ^{13}C NMR spectrum of **3ia**.

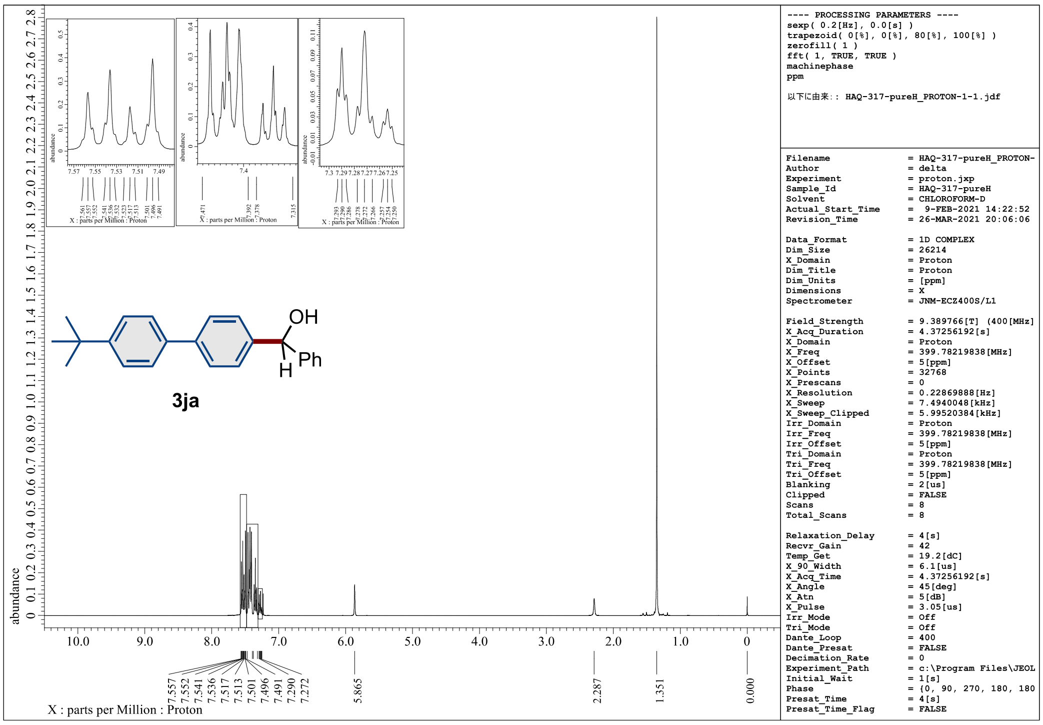

Supplementary Figure 84. ^{1}H NMR spectrum of **3ja**.

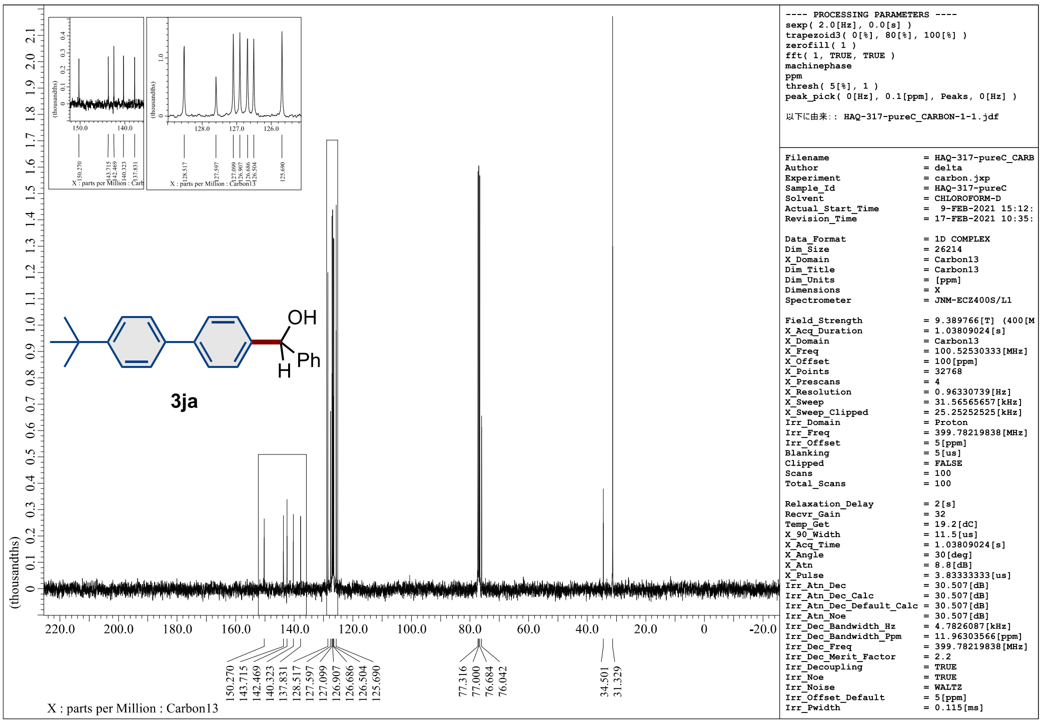

Supplementary Figure 85. ^{13}C NMR spectrum of **3ja**.

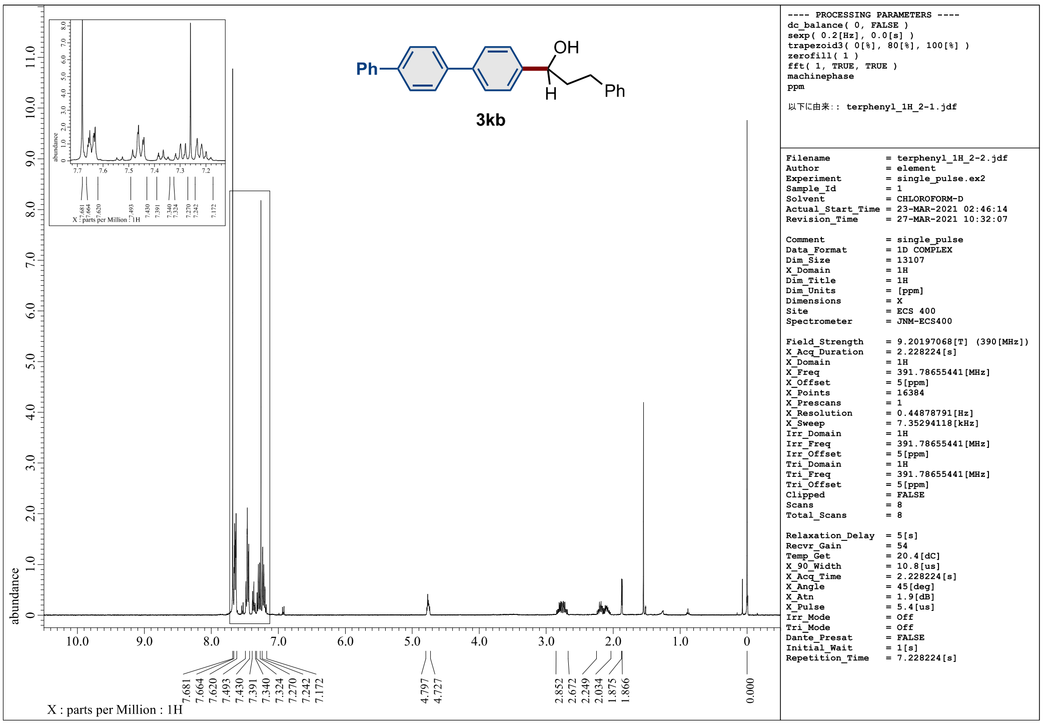

Supplementary Figure 86. ^{1}H NMR spectrum of **3kb**.

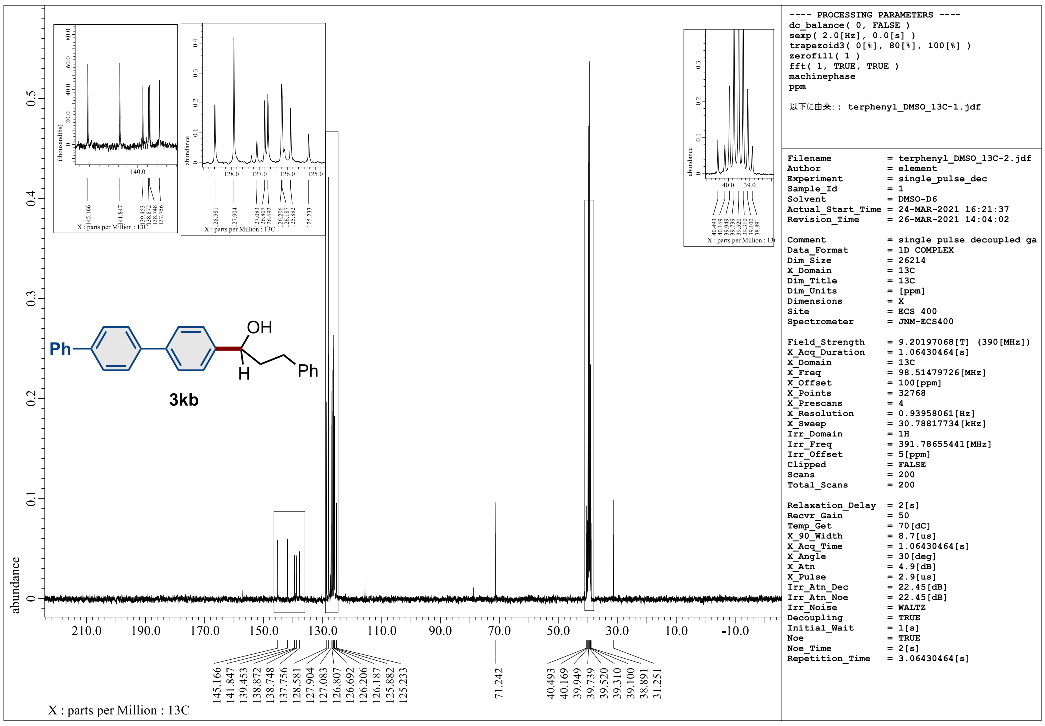

Supplementary Figure 87. ^{13}C NMR spectrum of **3kb**.

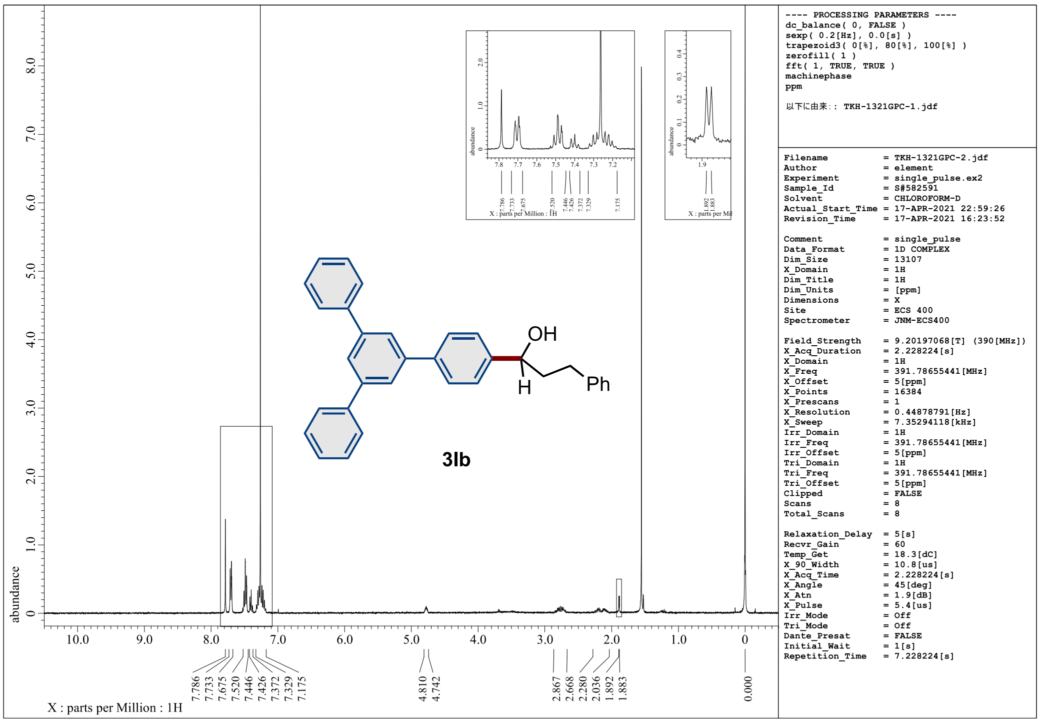

Supplementary Figure 88. ^{1}H NMR spectrum of **3lb**.

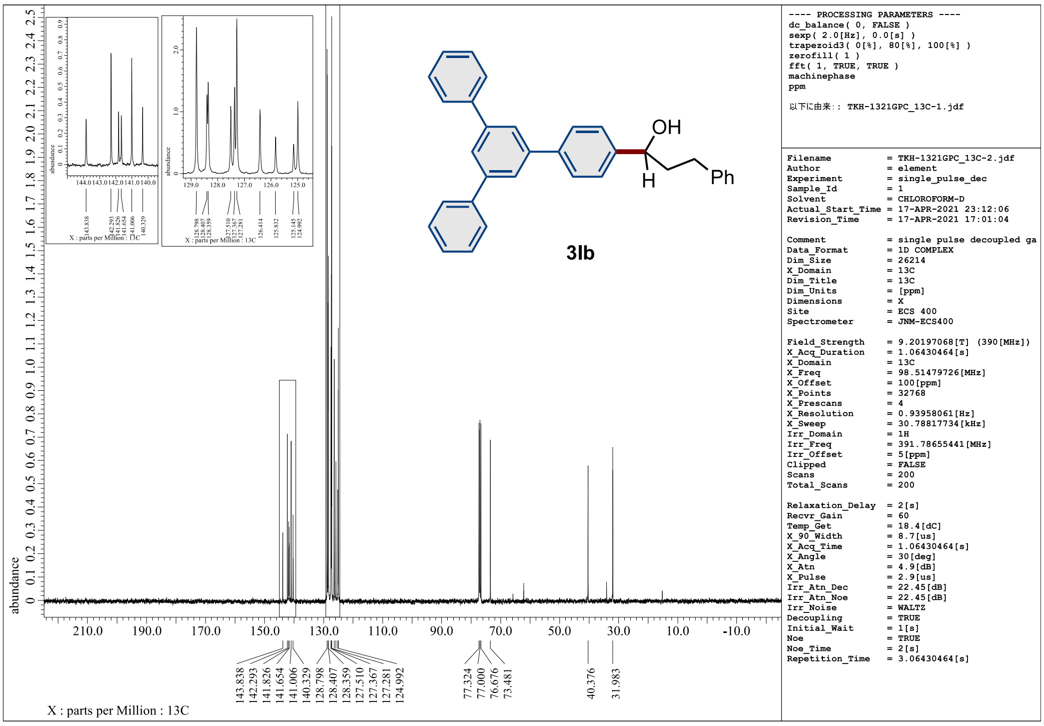

Supplementary Figure 89. ^{13}C NMR spectrum of **3lb**.

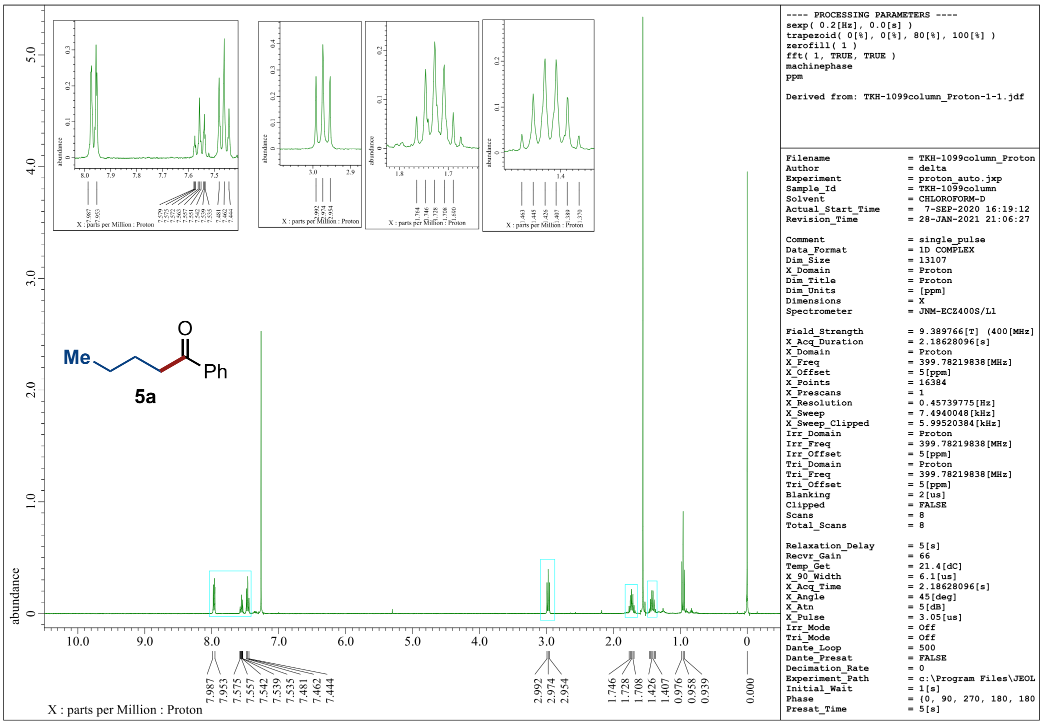

Supplementary Figure 90. ^{1}H NMR spectrum of **5a**.

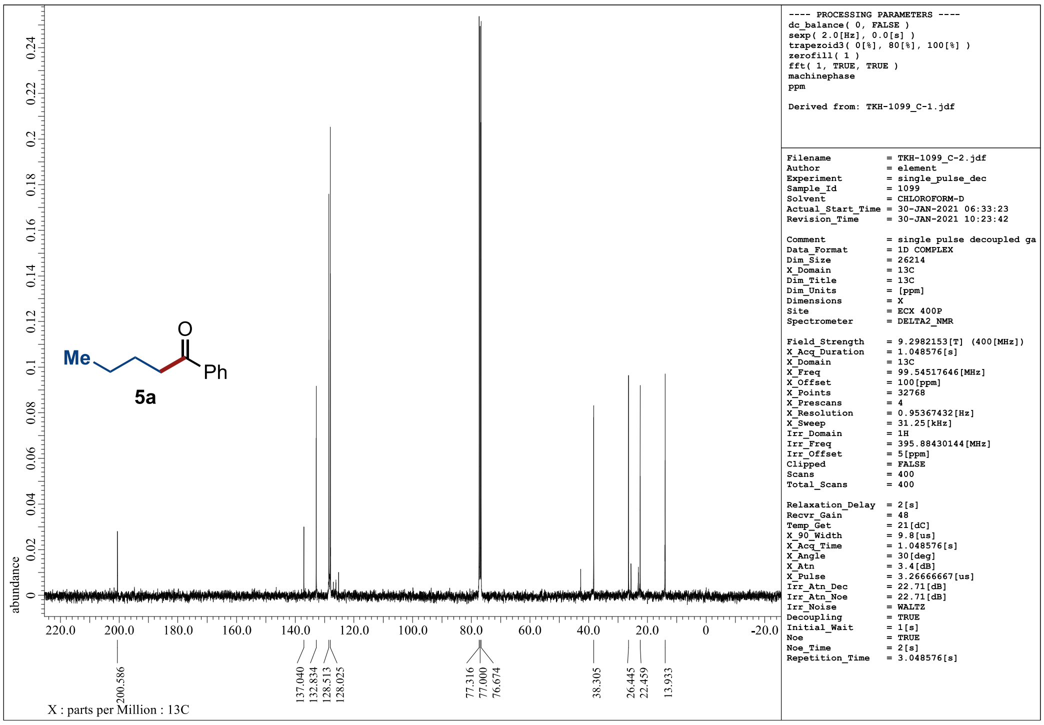

Supplementary Figure 91. ^{13}C NMR spectrum of **5a**.

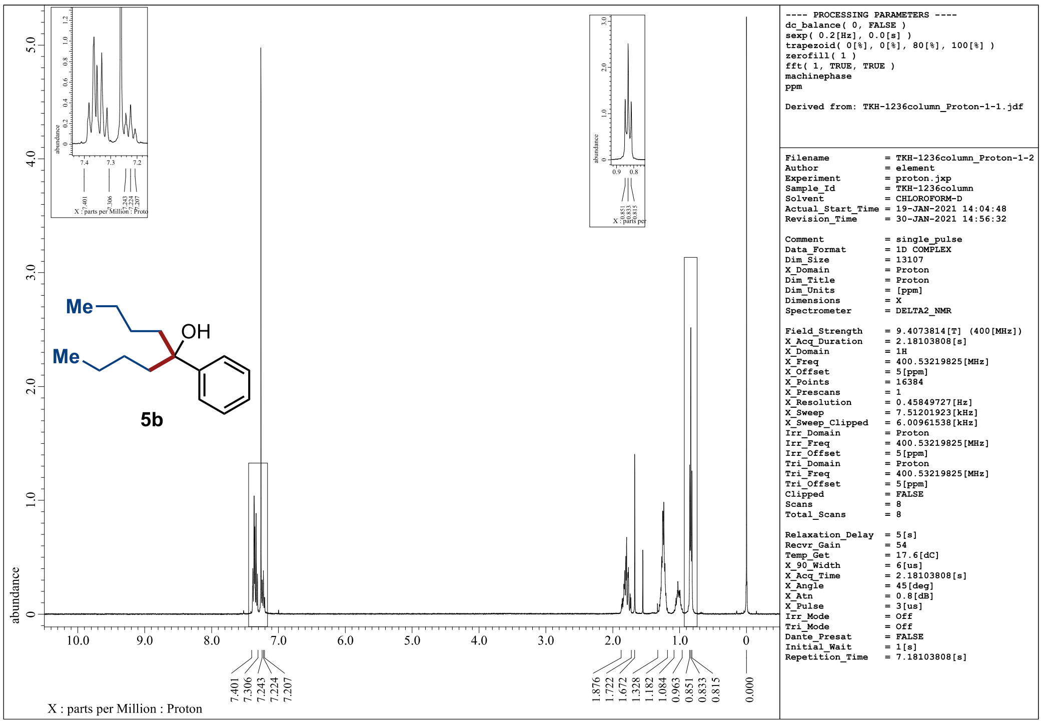

Supplementary Figure 92. ^{1}H NMR spectrum of **5b**.

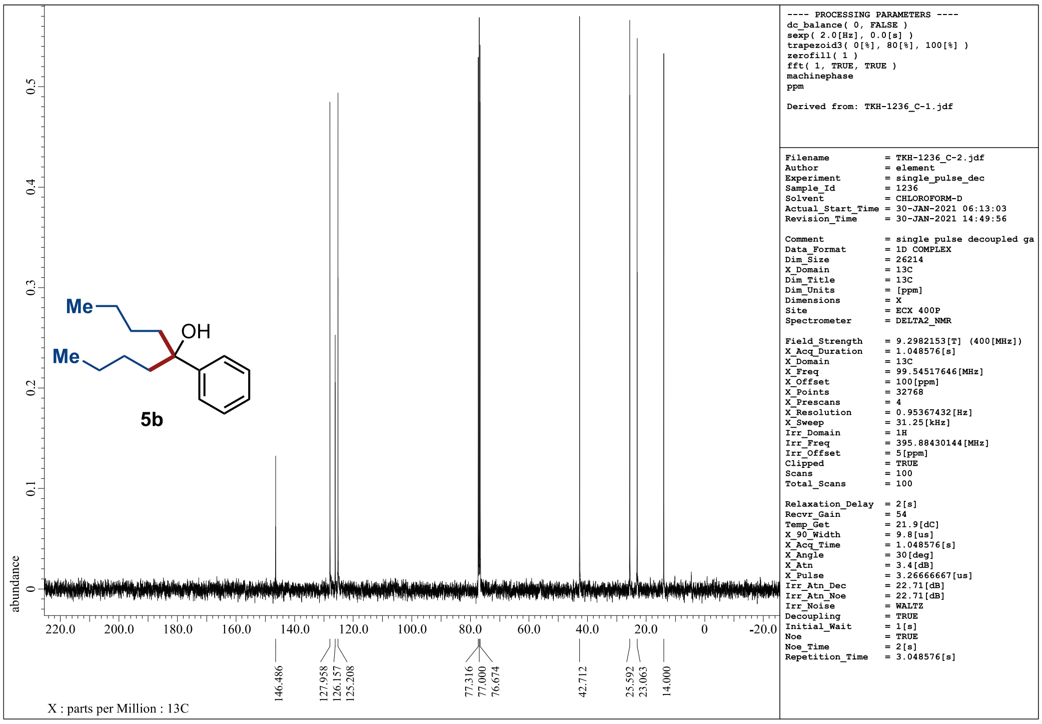

Supplementary Figure 93. ^{13}C NMR spectrum of **5b**.

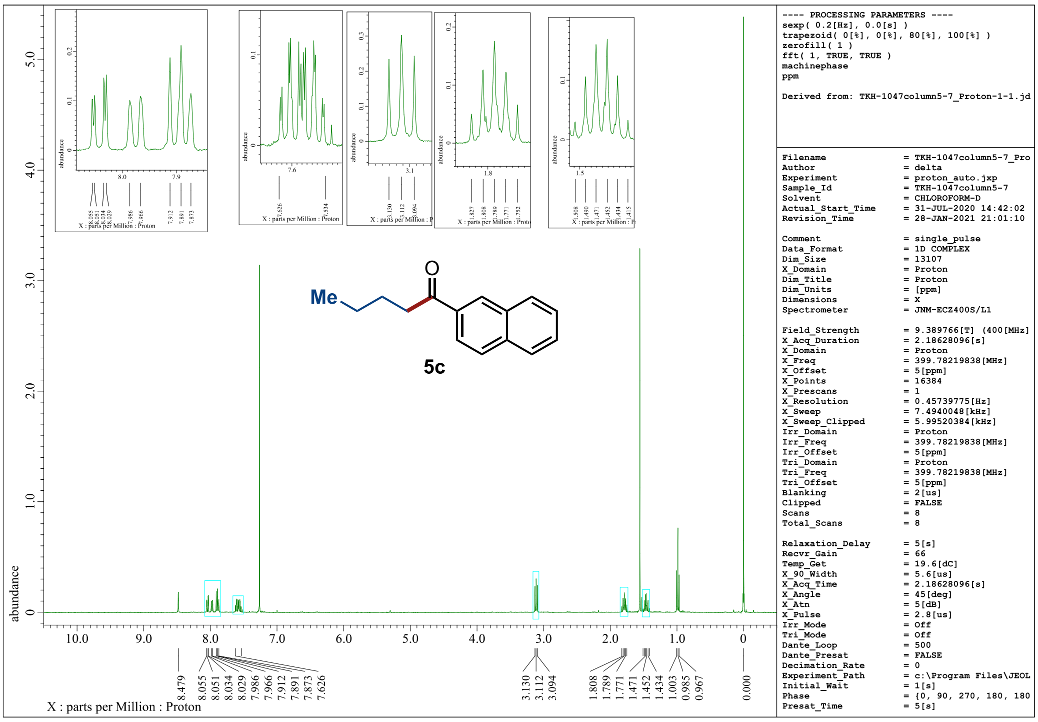

Supplementary Figure 94. ^{1}H NMR spectrum of **5c**.

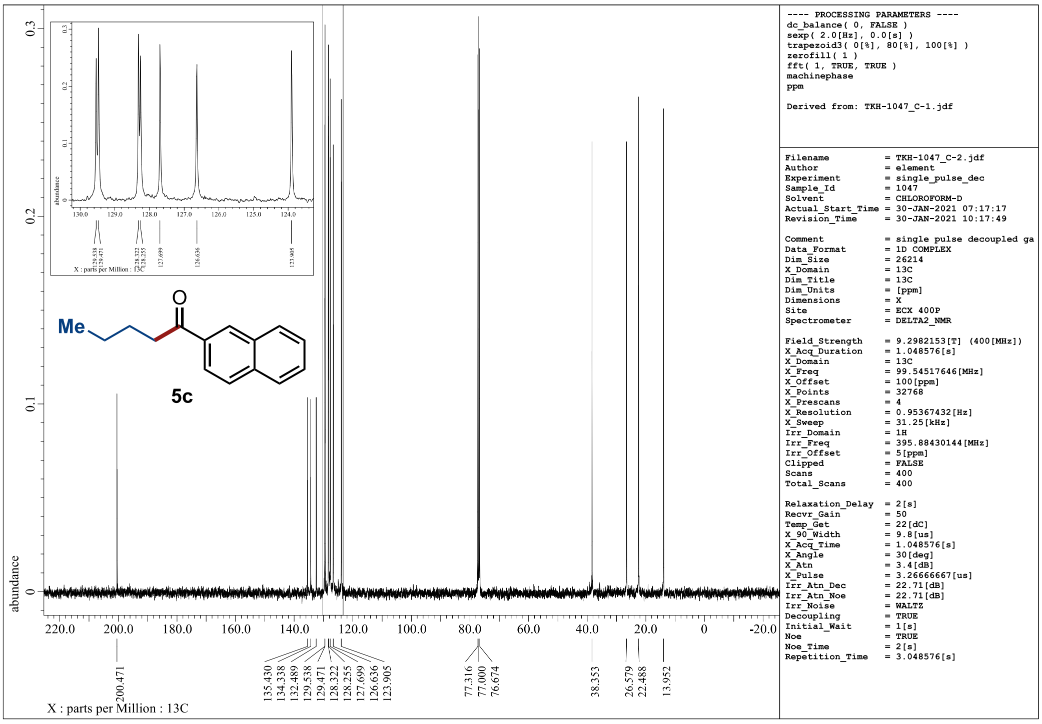

Supplementary Figure 95. ^{13}C NMR spectrum of **5c**.

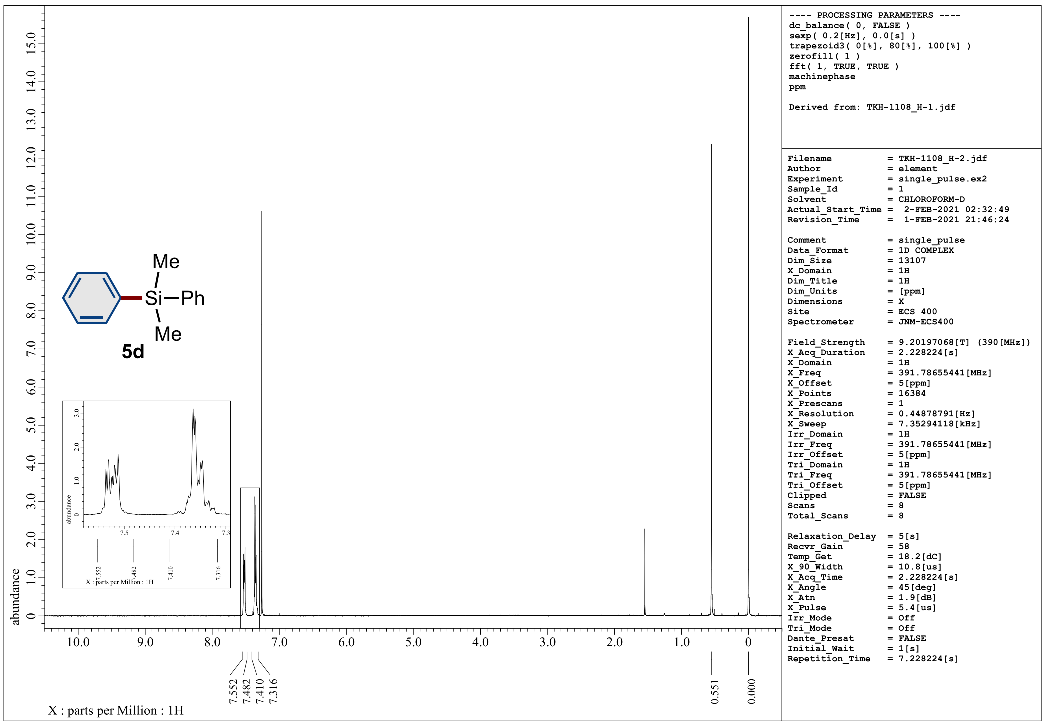

Supplementary Figure 96. ^{1}H NMR spectrum of **5d**.

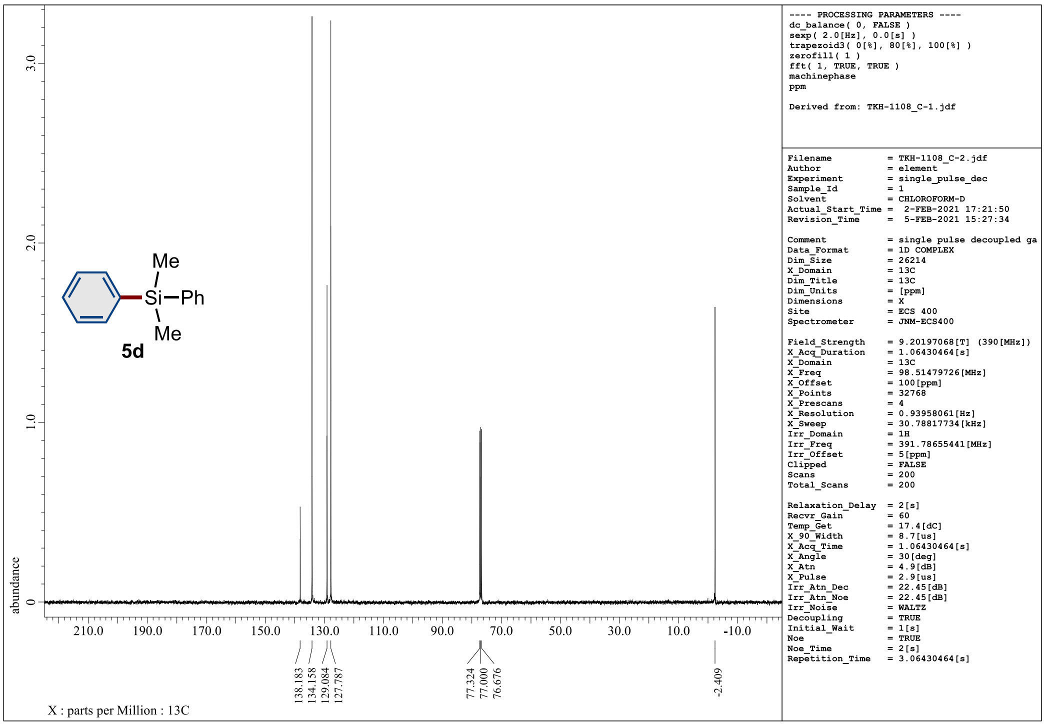

Supplementary Figure 97. ^{13}C NMR spectrum of **5d**.

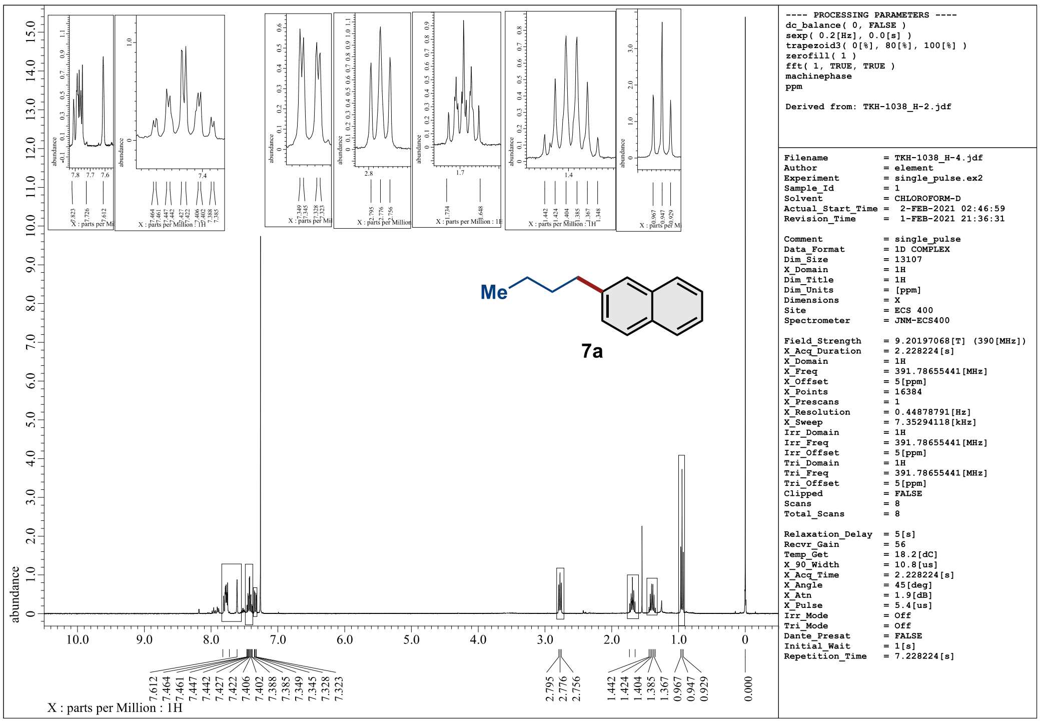

Supplementary Figure 98. ^{1}H NMR spectrum of **7a**.

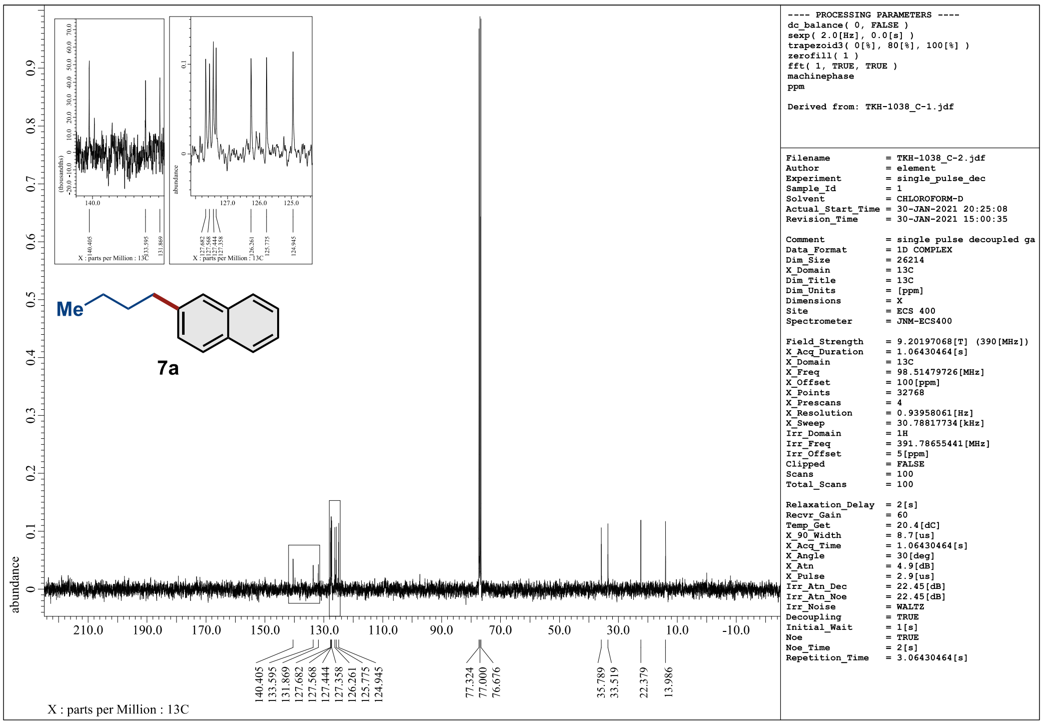

Supplementary Figure 99. ^{13}C NMR spectrum of **7a**.

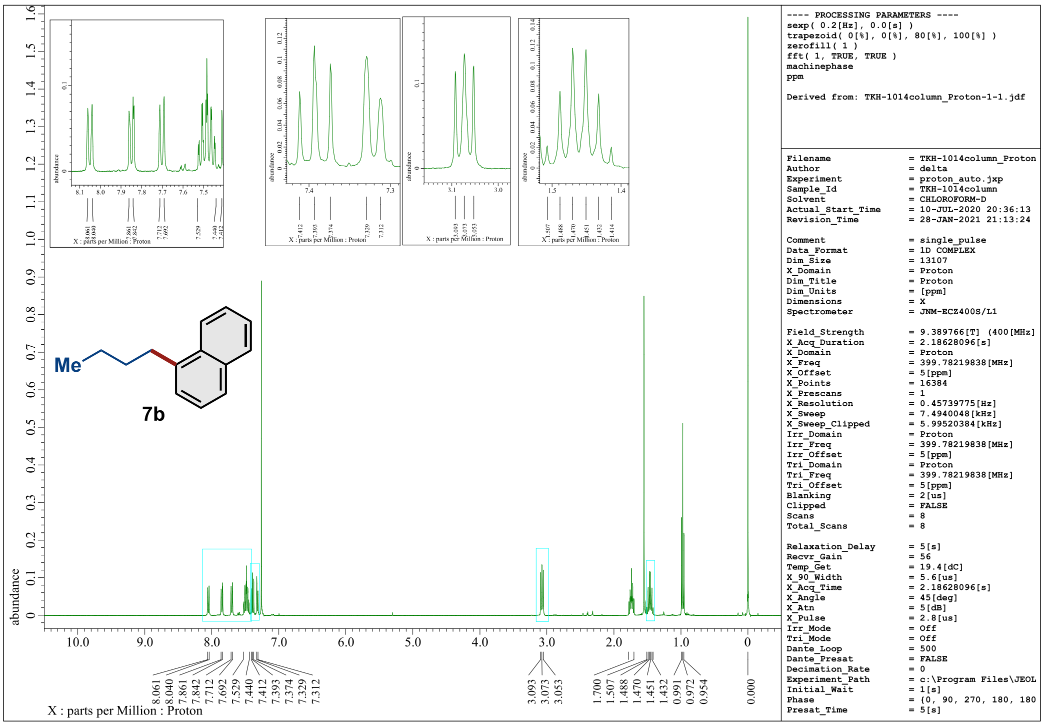

Supplementary Figure 100. ^{1}H NMR spectrum of **7b**.

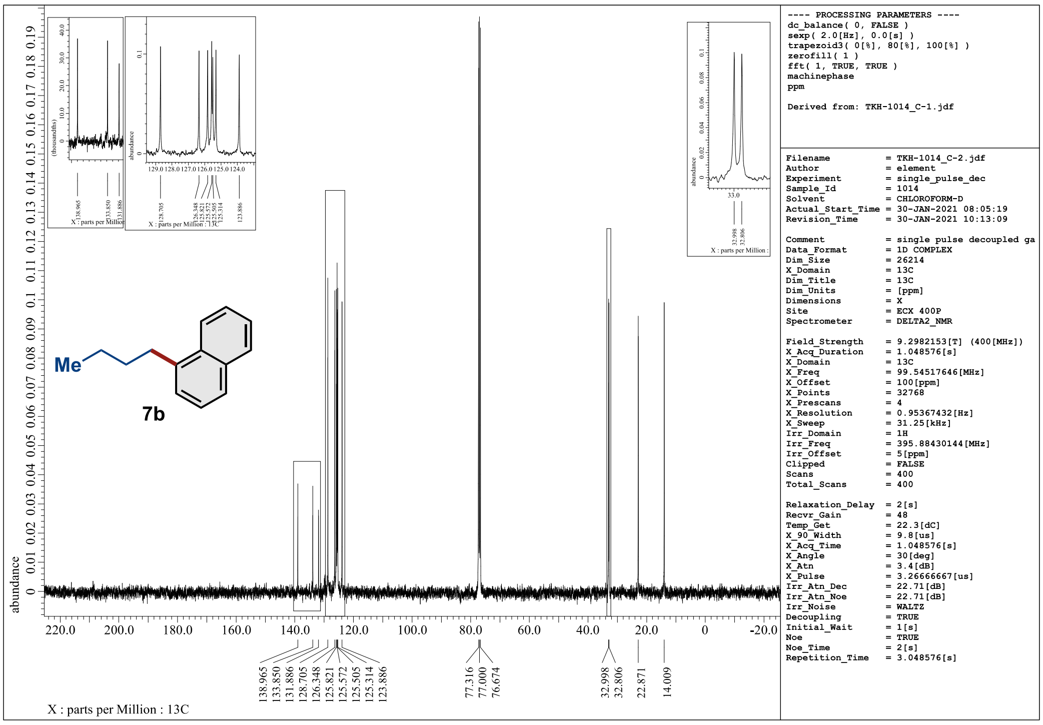

Supplementary Figure 101. ^{13}C NMR spectrum of **7b**.

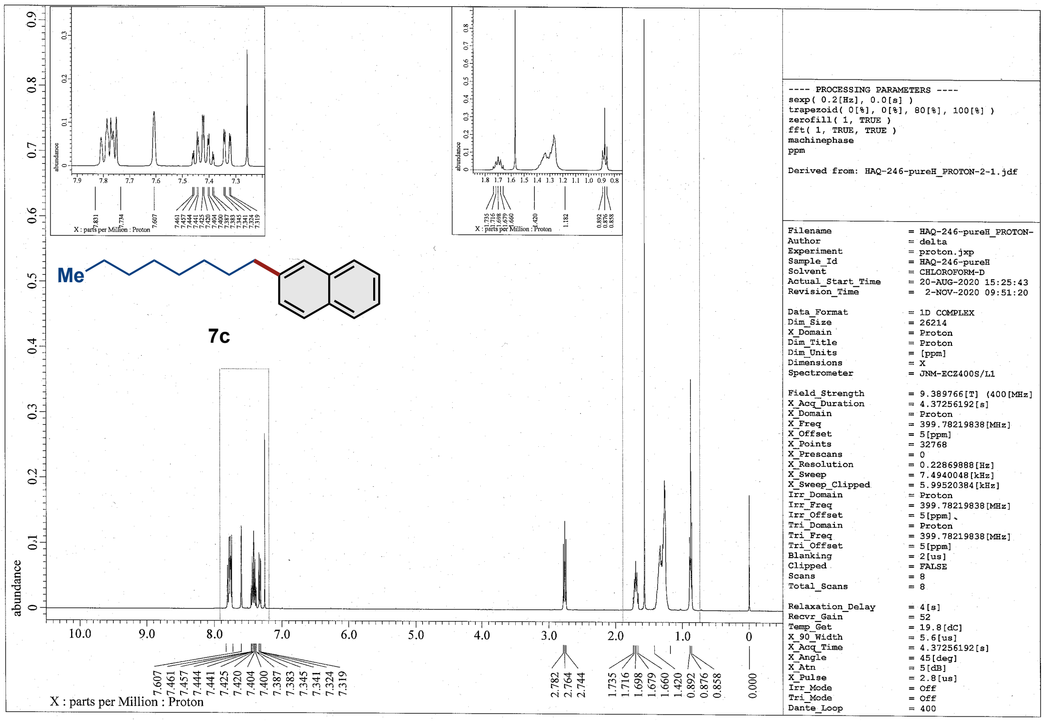

Supplementary Figure 102. ^{1}H NMR spectrum of **7c**.

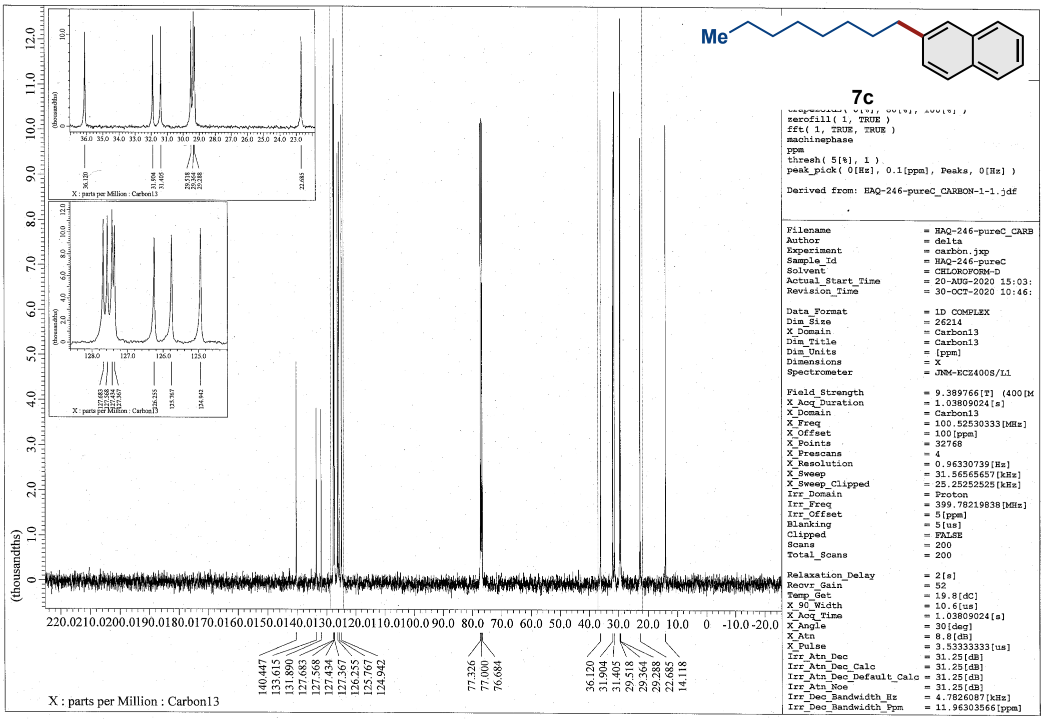

Supplementary Figure 103. ^{13}C NMR spectrum of **7c**.

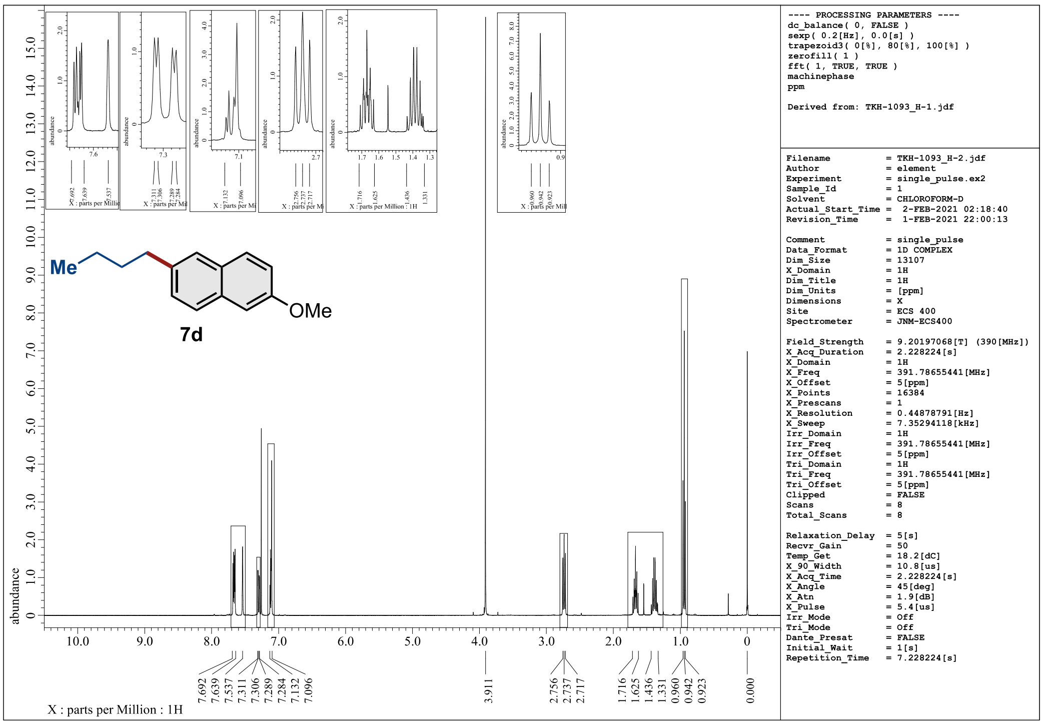

Supplementary Figure 104. ^{1}H NMR spectrum of **7d**.

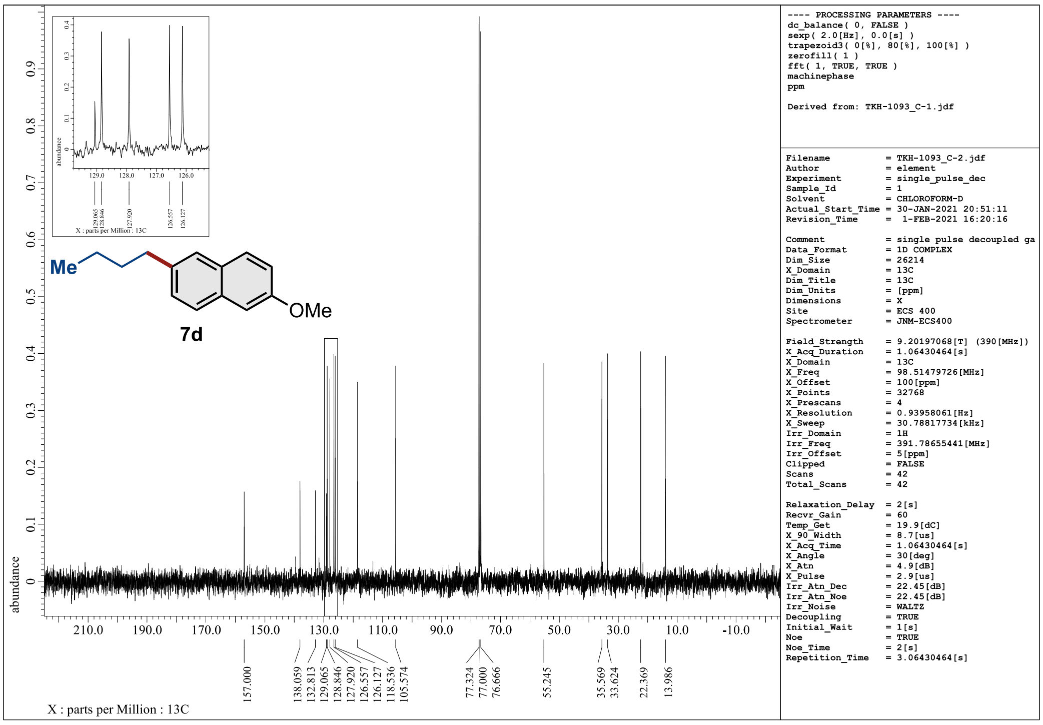

Supplementary Figure 105. ^{13}C NMR spectrum of **7d**.

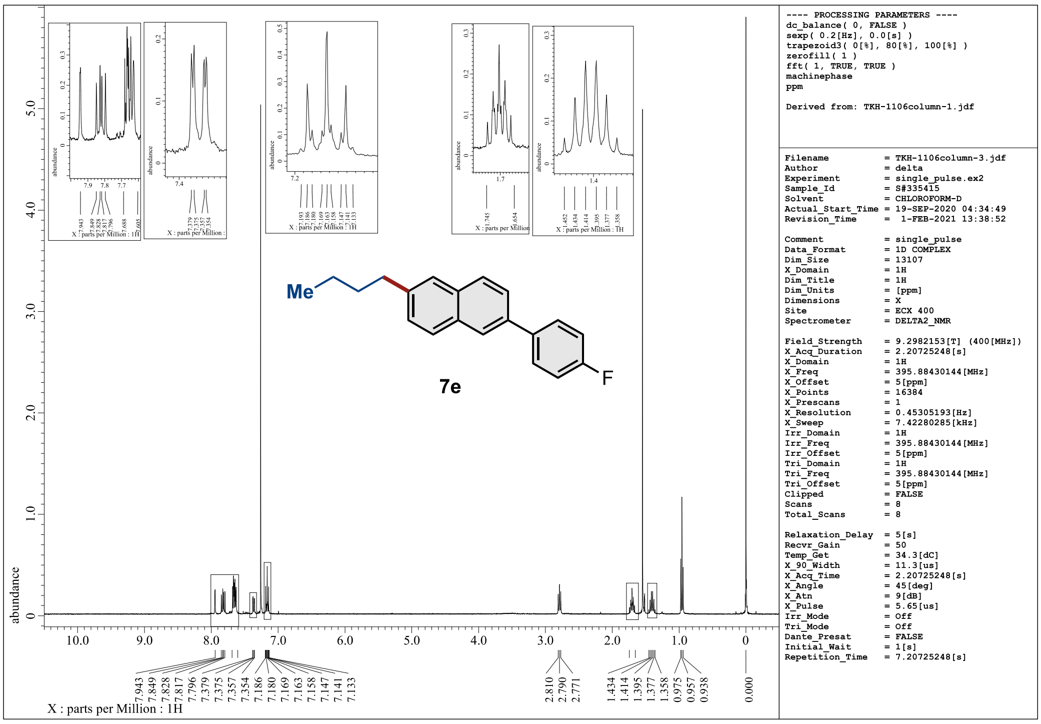

Supplementary Figure 106. ^{1}H NMR spectrum of **7e**.

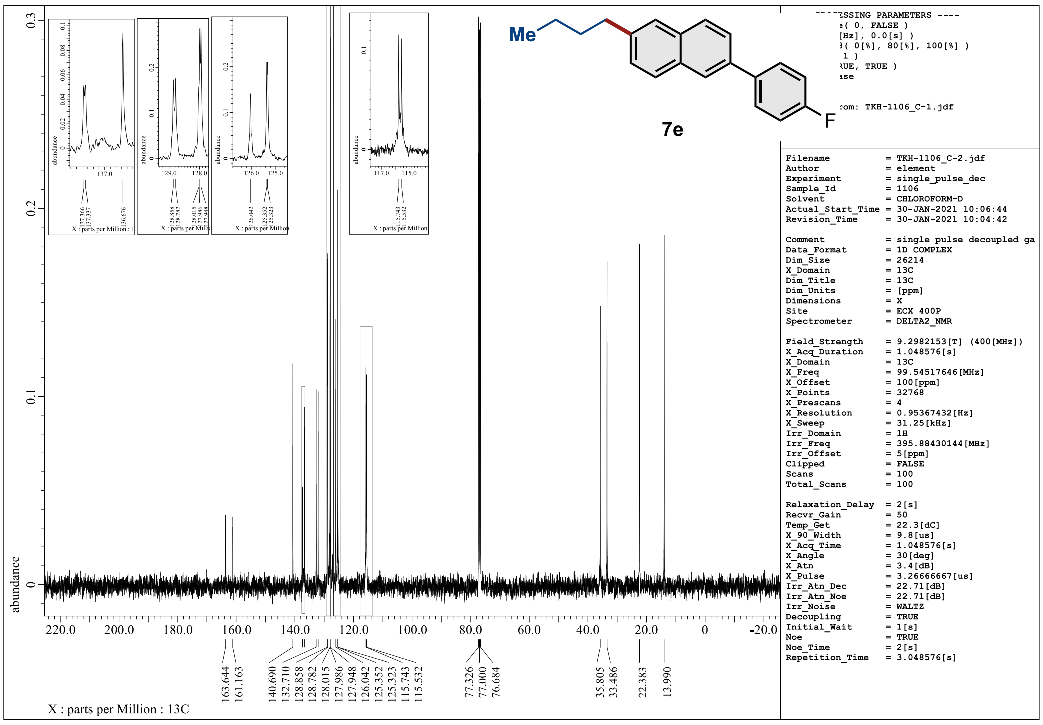

Supplementary Figure 107. ^{13}C NMR spectrum of **7e**.

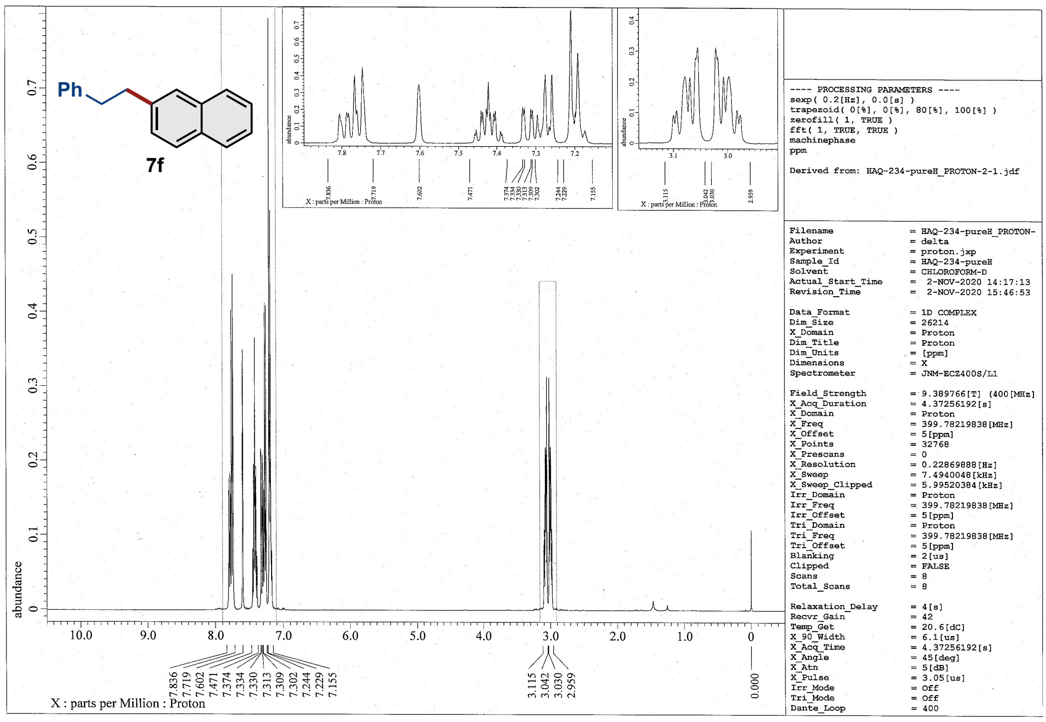

Supplementary Figure 108. ^{1}H NMR spectrum of **7f**.

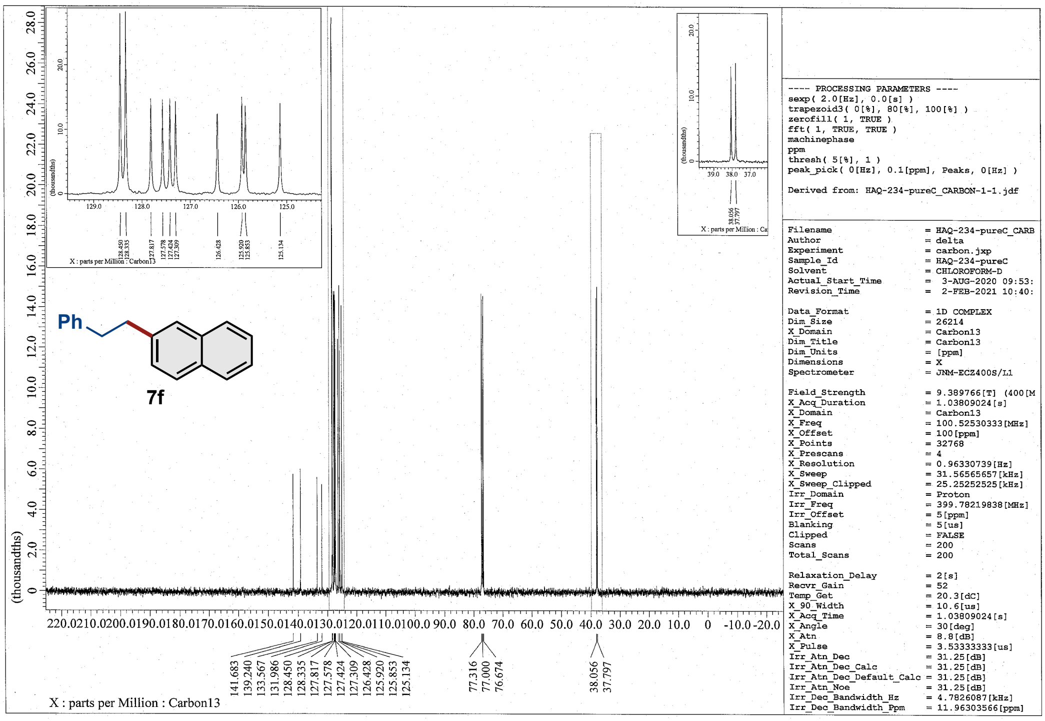

Supplementary Figure 109. ^{13}C NMR spectrum of **7f**.

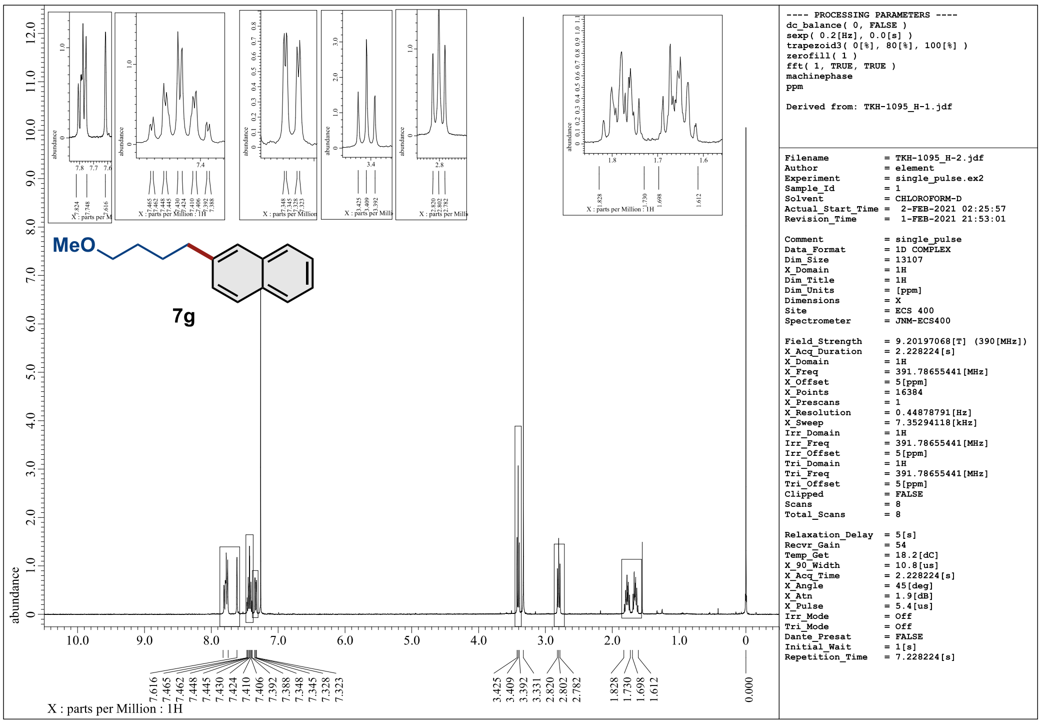

Supplementary Figure 110. ^{1}H NMR spectrum of **7g**.

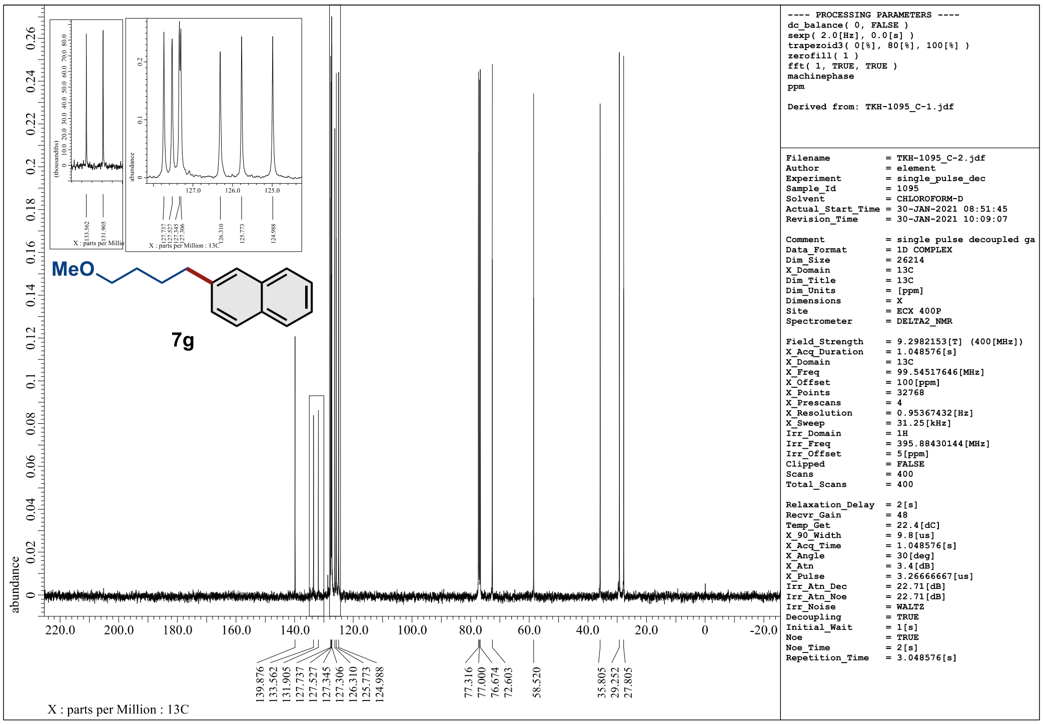

Supplementary Figure 111. ^{13}C NMR spectrum of **7g**.

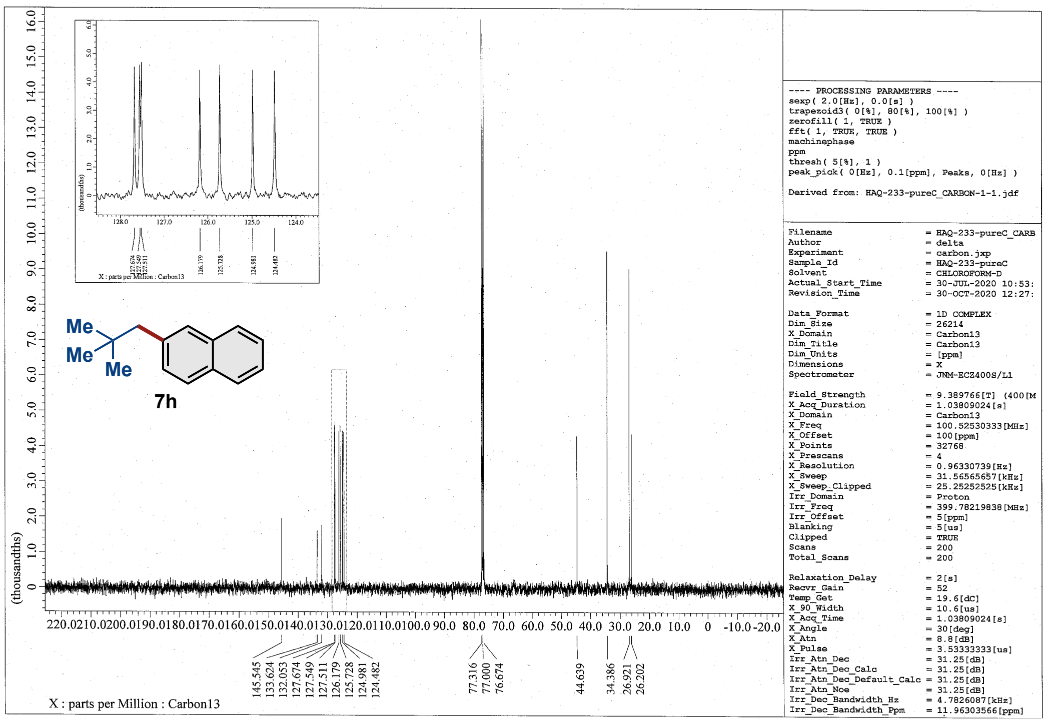

Supplementary Figure 112. ^{1}H NMR spectrum of **7h**.

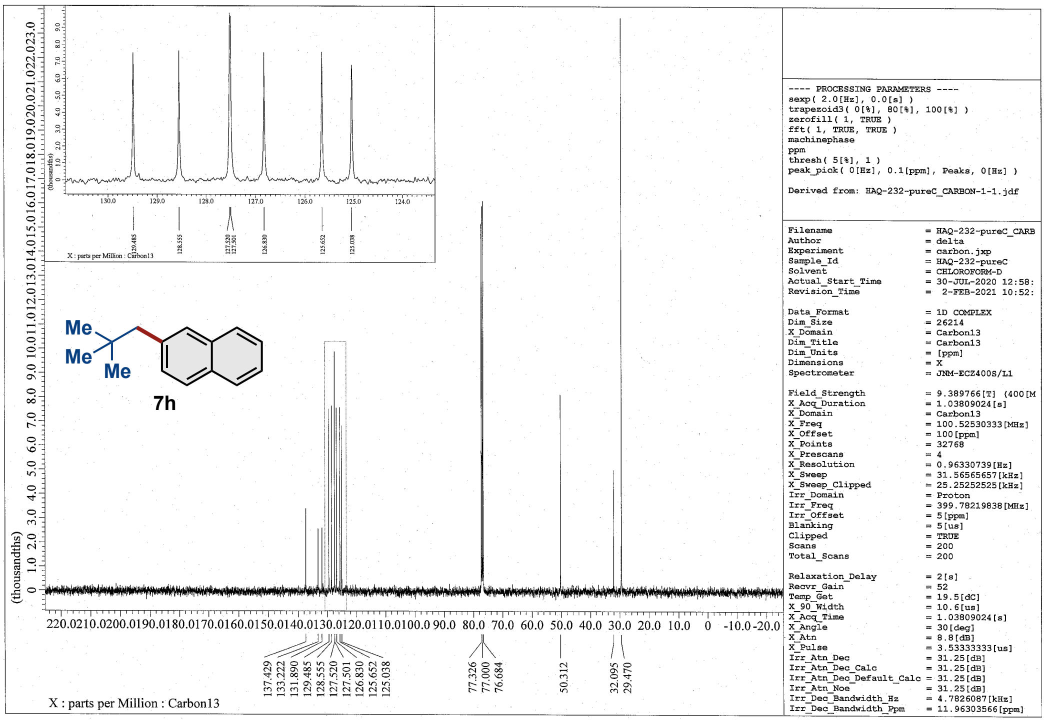

Supplementary Figure 113. ^{13}C NMR spectrum of **7h**.

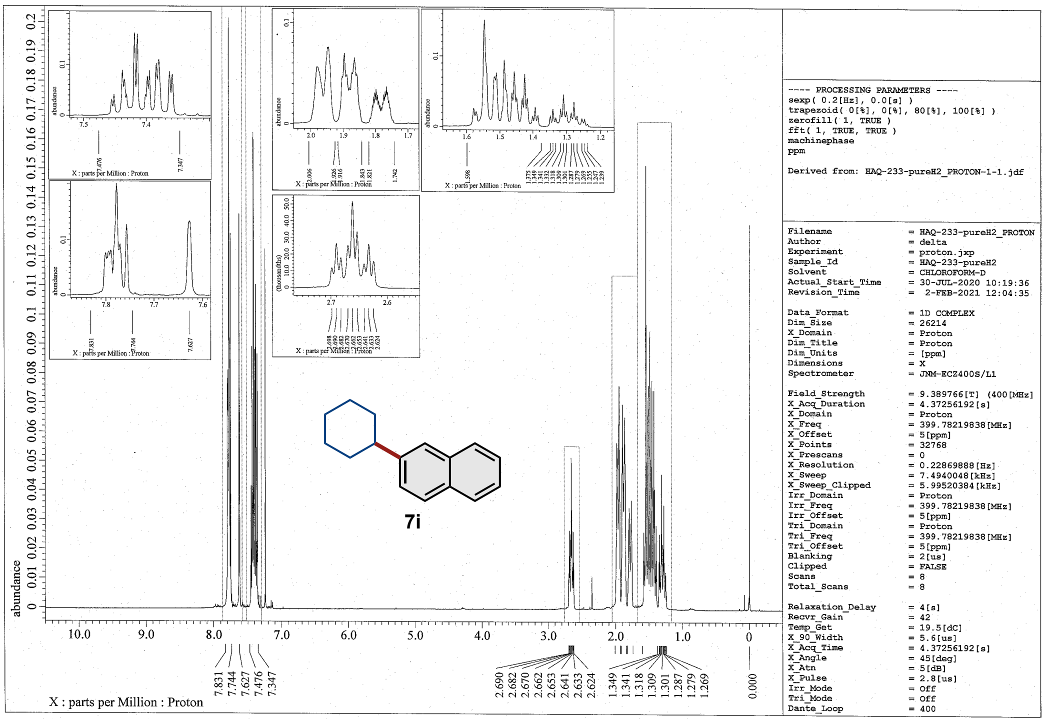

Supplementary Figure 114. ^{1}H NMR spectrum of **7i**.

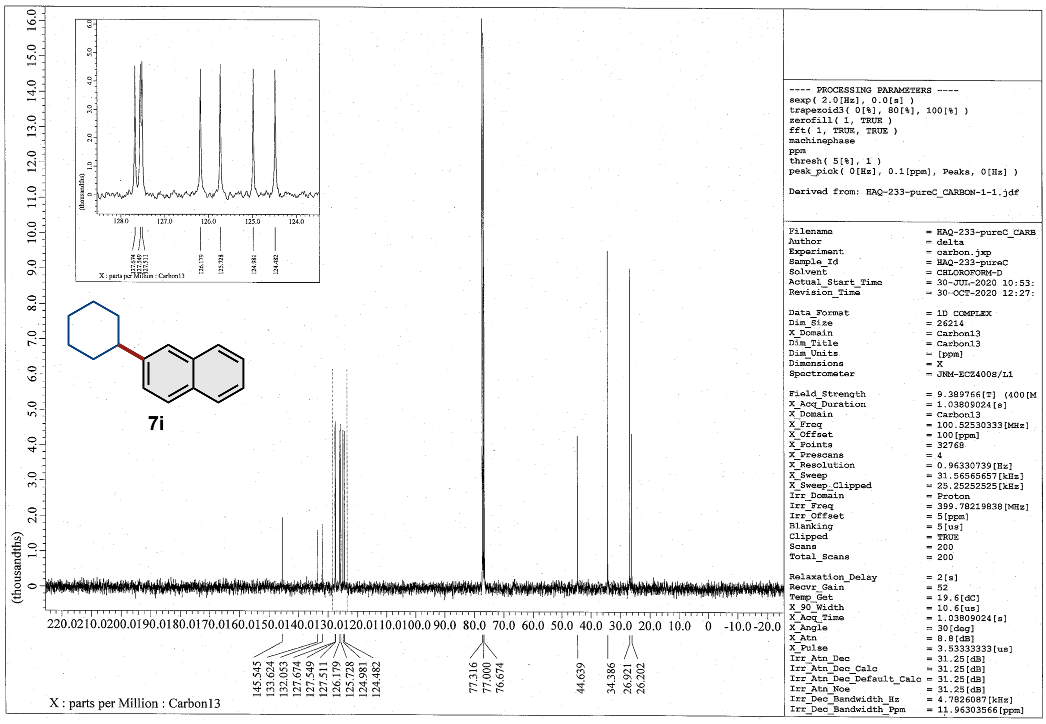

Supplementary Figure 115. ^{13}C NMR spectrum of **7i**.

Supplementary References

1. X. Lei, A. Jalla, M. A. A. Shama, J. M. Stafford, B. Cao, *Synthesis* **2015**, *47*, 2578–2585.
2. A. Hiraya, M. Watanabe, T. K. Sham, *Rev. Sci. Instrum.* **1995**, *66*, 1528–1530.
3. A 1.0 M THF solution of phenylmagnesium bromide (Cat. No. 32141-25) was purchase from TCI and used after sonication to completely dissolve the precipitation of magnesium halide.
4. T. Hatsui, E. Shigemasa, N. Kosugi, *AIP Conf. Proc.* **2004**, *705*, 921–924.
5. B. Ravel and M. Newville, *J. Synchrotron Radiat*. **2005**, *12*, 537–541.
6. T. Mori, S. Kato, *J. Phys. Chem. A* **2009**, *113*, 6158–6165.
7. R. M. Peltzer, O. Eisenstein, A. Nova, M. Cascella, *J. Phys. Chem. B* **2017**, *121*, 4226–4237.
8. R. M. Peltzer, J. Gauss, O. Eisenstein, M. Cascella, *J. Am. Chem. Soc.* **2020**, *142*, 2984–2994.
9. S. Maeda, Y. Harabuchi, M. Takagi, T. Taketsugu, K. Morokuma, *Chem. Rec.* **2016**, *16*, 2232–2248.
10. S. Maeda, Y. Harabuchi, M. Takagi, K. Saita, K. Suzuki, T. Ichino, T. Sumiya, K. Sugiyama, Y. Ono, *J. Comput. Chem.* **2018**, *39*, 233–251.
11. S. Grimme, C. Bannwarth, P. Shushkov, *J. Chem. Theory Comput.* **2017**, *13*, 1989–2009.
12. M. Bursch, H. Neugebauer, S. Grimme, *Angew. Chem. Int. Ed.* **2019**, *58*, 11078–11087.
13. F. Neese, *WIREs Comput. Mol. Sci.* **2012**, *2*, 73–78.
14. F. Neese, *WIREs Comput. Mol. Sci.* **2018**, *8*, e1327.
15. A. D. Becke, *Phys. Rev. A* **1988**, *38*, 3098–3100.
16. A. D. Becke, *J. Chem. Phys*. **1933**, *98*, 1372–1377.
17. C. Lee, W. Yang, R. G. Parr, *Phys. Rev. B* **1988**, *37*, 785–789.
18. S. Grimme, J. Antony, S. Ehrlich, H. Krieg, *J. Chem. Phys.* **2010**, *132*, 154104.
19. F. Weigend, R. Ahlrichs, *Phys. Chem. Chem. Phys.* **2005**, *7*, 3297–3305.
20. F. Weigend, *Phys. Chem. Chem. Phys.* **2006**, *8*, 1057–1065.
21. F. Neese, F. Wennmohs, A. Hansen, U. Becker, *Chem. Phys.* **2009**, *356*, 98–109.
22. R. Izsák, F. Neese, *J. Chem. Phys.* **2011**, *135*, 144105.
23. R. N. Gaykar, A. Bhunia, A. T. Biju, *J. Org. Chem.* **2018**, *83*, 11333–11340.
24. Y.-X. Liao, C.-H. Xing, Q.-S. Hu, *Org. Lett.* **2012**, *14*, 1544–1547.
25. Q. Zhu, Z. He, L. Wang, Y. Hu, C. Xia, C. Liu, *Chem. Commun*. **2019**, *55*, 11884–11887.
26. T. W. Ng, G. Liao, K. K. Lau, H.-J. Pan, Y. Zhao, *Angew. Chem., Int. Ed.* **2020**, *59*, 11384–11389.
27. Z.-L. Shen, Y.-L. Yeo, T.-P. Loh, *J. Org. Chem.* **2008**, *73*, 3922–3924.
28. T. Yamamoto, T. Ohta, Y. Ito, *Org. Lett.* **2005**, *7*, 4153–4155.
29. K. Polidano, B. G. Reed-Berendt, A. Basset, A. J. A. Watson, J. M. J. Williams, L. C. Morrill, *Org. Lett.* **2017**, *19*, 6716–6719.
30. X. Wu, X. Li, W. Huang, Y. Wang, H. Xu, L. Cai, J. Qu, Y. Chen, *Org. Lett.* **2019**, *21*, 2453–2458.
31. E. Fernµndez-Mateos, B. Maciµ, M. Yus, *Adv. Synth. Catal.* **2013**, *355*, 1249–1254.
32. D. Chen, Y. Zhang, X. Pan, F. Wang, S. Huang, *Adv. Synth. Catal.* **2018**, *360*, 3607–3612.
33. P. C. Too, Y. L. Tnay, S. Chiba, *Beilstein J. Org. Chem.* **2013**, *9*, 1217–1225.

34. S. R. Tamang, M. Findlater, *J. Org. Chem.* **2017**, *82*, 12857–12862.
35. V. Bagutski, R. M. French, V. K. Aggarwal, *Angew. Chem., Int. Ed.* **2010**, *49*, 5142–5145.
36. B. Bieszczad, D. G. Gilheany, *Angew. Chem., Int. Ed.* **2017**, *56*, 4272–4276.
37. K. Li, N. Hu, R. Luo, W. Yuan, W. Tang, *J. Org. Chem.* **2013**, *78*, 6350–6355.
38. Y. Xia, J. Wang, G. Dong, *J. Am. Chem. Soc.* **2018**, *140*, 5347–5351.
39. E. Morita, K. Murakami, M. Iwasaki, K. Hirano, H. Yorimitsu, K. Oshima, *Bull. Chem. Soc. Jpn.* **2009**, *82*, 1012–1014.
40. Y. Sumida, T. Sumida, T. Hosoya, *Synthesis* **2017**, *49*, 3590–3601.
41. T. Agrawal, S. P. Cook, *Org. Lett*. **2013**, *15*, 96–99.
42. M. Tobisu, T. Takahira, T. Morioka, N. Chatani, *J. Am. Chem. Soc*. **2016**, *138*, 6711–6714.
43. M. Tobisu, T. Takahira, N. Chatani, *Org. Lett.* **2015**, *17*, 4352–4355.
